# Modular Access to Diverse Chemiluminescent Dioxetane‐Luminophores through Convergent Synthesis

**DOI:** 10.1002/anie.202202187

**Published:** 2022-03-29

**Authors:** Samer Gnaim, Sachin Popat Gholap, Liang Ge, Sayantan Das, Sara Gutkin, Ori Green, Omri Shelef, Nir Hananya, Phil S. Baran, Doron Shabat

**Affiliations:** ^1^ School of Chemistry Raymond and Beverly Sackler Faculty of Exact Sciences 69978 Tel Aviv Israel; ^2^ Department of Chemistry Scripps Research 10550 North Torrey Pines Road La Jolla CA 92037 USA

**Keywords:** Chemiluminescence, Cross-Coupling, Dioxetanes, Palladium, Synthetic Methods

## Abstract

Adamantyl‐dioxetane luminophores are an important class of chemiluminescent molecular probes for diagnostics and imaging. We have developed a new efficient synthetic route for preparation of adamantyl‐enolether as precursors for dioxetane chemiluminescent luminophores. The synthesis is convergent, using an unusual Stille cross‐coupling reaction employing a stannane‐enolether, to directly afford adamantyl‐enolether. In a following simple step, the dioxetane is obtained by oxidation of the enolether precursor with singlet‐oxygen. The scope of this synthetic route is broad since a large number of haloaryl substrates are either commercially available or easily accessible. Such a late‐stage derivatization strategy simplifies the rapid exploration of novel luminogenic molecular structures in a library format and simplifies the synthesis of known dioxetane luminophores. We expect that this new synthetic strategy will be particularly useful in the design and synthesis of yet unexplored dioxetane chemiluminescent luminophores.

## Graphical abstract

A new efficient synthetic route for preparation of adamantyl‐enolether as precursors for dioxetane chemiluminescent luminophores was developed. The synthesis is convergent, using an unusual Stille cross‐coupling reaction employing a stannane‐enolether, to directly afford adamantyl‐enolether. The scope of this synthetic route is broad since a large number of haloaryl substrates are either commercially available or easily accessible.

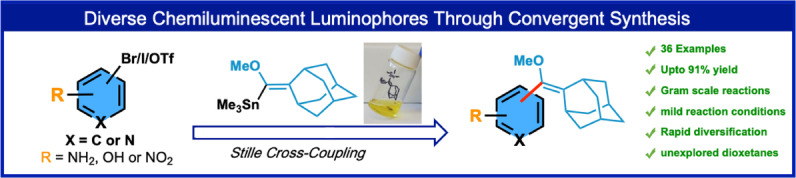

## Introduction

**Figure 1:**
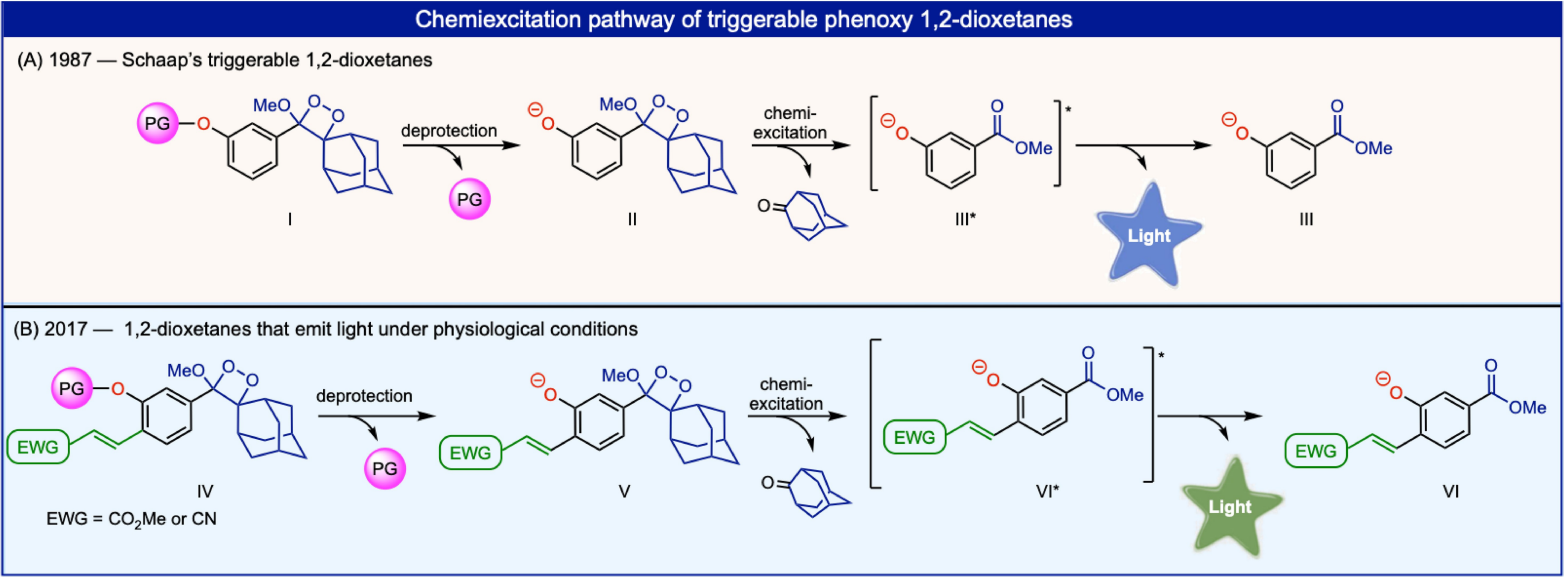
The general structure and chemiexcitation pathway of A) Schaap's dioxetane‐based probes, B) acrylate or acrylo‐nitrile phenoxy 1,2‐dioxetane probes (PG, protecting group).

The thermal and mechanical decompositions of 1,2‐dioxetanes have been extensively explored in the past, as these highly energetic molecules possess chemiluminescence light‐emission properties.[^1^, ^2^, ^3^] A ground‐breaking milestone in the chemistry of such compounds was achieved, more than 30 years ago, with the discovery of triggerable dioxetanes by the Schaap group.^[4]^ In such compounds, chemiexcitation is initiated as a consequence of phenolate formation, which occurs following deprotection of phenols (Figure 1A). Thus, upon removal of a phenol‐protecting group, by a specific analyte or enzyme, light emission can be produced. However, Schaap's dioxetanes suffer from an inherent limitation: The chemiluminescence emission of these luminophores is extremely weak under aqueous conditions. Therefore, such dioxetane luminophores cannot be used in water as a sole component, and additives are used to enhance the light emission intensity. During the last several years, our group has explored new approaches for amplifying the chemiluminescence light emission under physiological conditions.[^5^, ^6^, ^7^] Remarkably, we were able to substantially increase the chemiluminescence quantum yield of Schaap's adamantly‐dioxetanes, in aqueous solution, by simply improving the emissive nature of the excited species formed during the chemiexcitation (Figure 1, species VI* and species III*). Phenoxy‐dioxetane luminophores bearing conjugated electron‐withdrawing substituents at their *ortho* position release a benzoate derivative during their chemiexcitation, resulting in a species that was found to be highly emissive under aqueous conditions (Figure 1B). These phenoxy‐dioxetane luminophores exhibited light emission intensities up to 3000‐fold higher than that obtained by the parent Schaap dioxetane.^[8]^ This discovery has enabled, for the first time, the use of dioxetane chemiluminescent luminophores as single component probes for various applications that require physiological conditions. Numerous groups over the world have already taken advantage of this discovery to prepare various chemiluminescent probes, which are based on these new luminophores.[^9^, ^10^, ^11^, ^12^, ^13^, ^14^, ^15^, ^16^] After more than 30 years of dormancy in this research area, we have succeeded in taking a major step forward, by understanding how to improve and control light emission properties under aqueous conditions. Chemiluminescence applications of dioxetane luminophores is now poised for broader use in a variety of exciting contexts.

**Figure 2:**
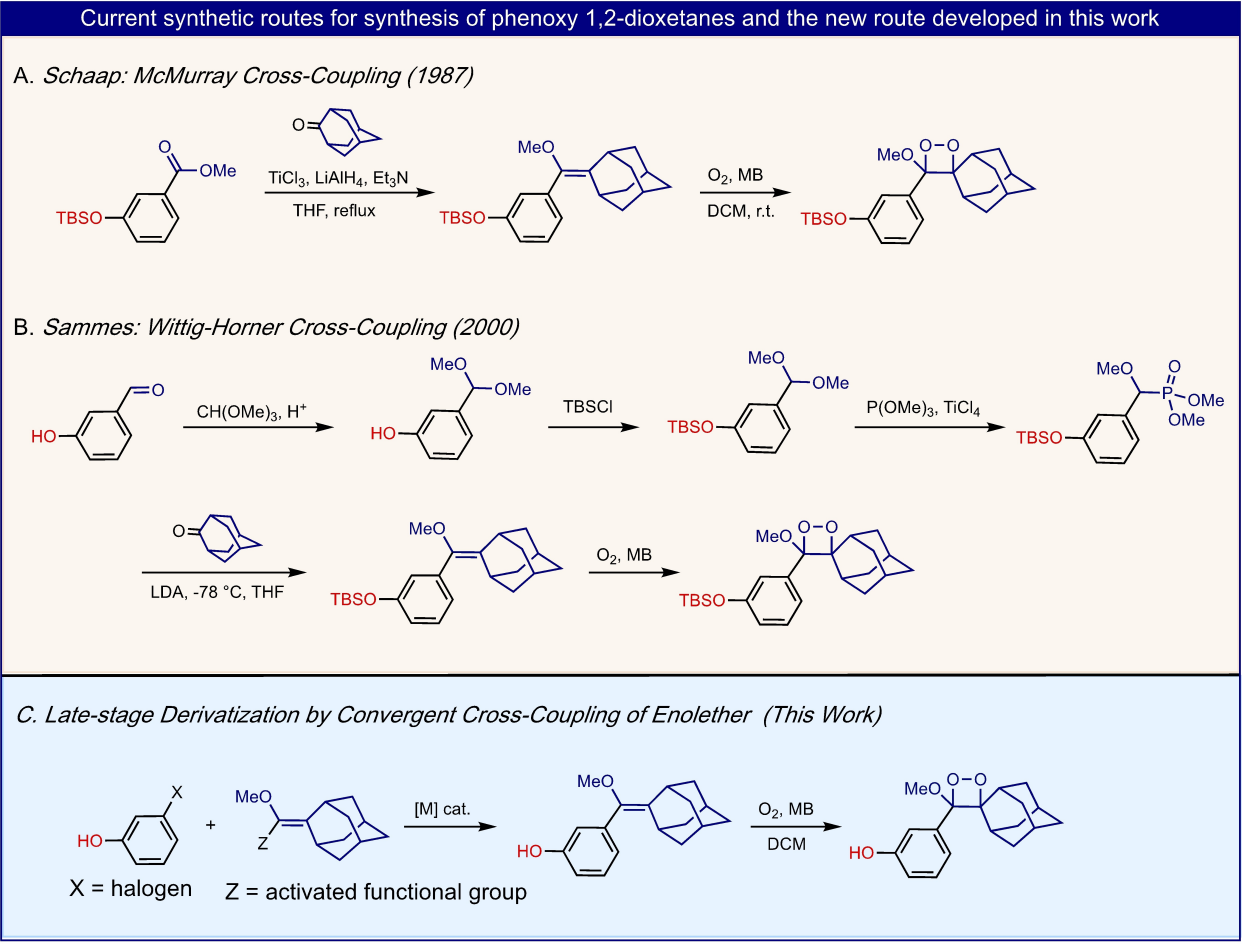
A, B) Current synthetic approaches for the preparation of adamantyl‐phenoxy‐enolethers and their correspondent dioxetanes. C) Late‐stage derivatization by convergent cross‐coupling of halo‐phenol and activated enolether.

The synthetic route developed more than 30 years ago by Schaap and co‐workers, to access this useful motif, relies on a hetero‐McMurray‐type coupling of a ketone with an ester to afford an enolether precursor, which is then oxidized to produce the target molecule: an adamantyl‐phenoxy‐dioxetane (Figure 2A).^[4]^ Generally, this reaction involves the use of a large excess (about 10 eq.) of the coupling reagents. Furthermore, the harsh reaction conditions, resulting of the presence of the pyrophoric titanium trichloride, hamper widespread use in academic or industrial settings. In 2000, the group of Sammes reported a five‐step synthesis that utilized a key Wittig–Horner cross‐coupling reaction to obtain the enolether precursor (Figure 2B).^[17]^ Implementation of this synthetic route by our group and others has enabled the preparation of numerous adamantyl‐phenoxy‐dioxetane derivatives, although this pathway requires multi‐step synthesis, and harsh reaction conditions, such as; the need for a strong base in the Wittig–Horner step.[^9^, ^12^, ^18^, ^19^, ^20^, ^21^, ^22^, ^23^, ^24^] In addition, the scope of available *meta* phenoxy‐aldehydes as starting materials is also limited. Due to the aforementioned limitations, a more robust and efficient synthesis of the enolether intermediate should be developed to enable the preparation of new challenging dioxetane luminophores. With an efficient synthesis in hand, numerous new dioxetane luminophores could be rapidly prepared and screened for their light emission properties. Ideally, such a synthesis would be based on a late‐stage derivatization, alongside with high tolerance towards various functional groups.[^25^, ^26^] This synthetic pathway could possibly be achieved by a metal‐catalyzed cross‐coupling reaction^[27]^ between ubiquitous aryl‐halides and an activated enolether module (Figure 2C).

Herein, we describe a general convergent cross‐coupling approach for the synthesis of 1,2‐adamantylidene enolether precursors of dioxetanes. This late‐stage approach to constructing such systems enables the rapid preparation of a large scope of new 1,2‐dioxetane‐based chemiluminescent luminophores.

## Results and Discussion

**Figure 3:**
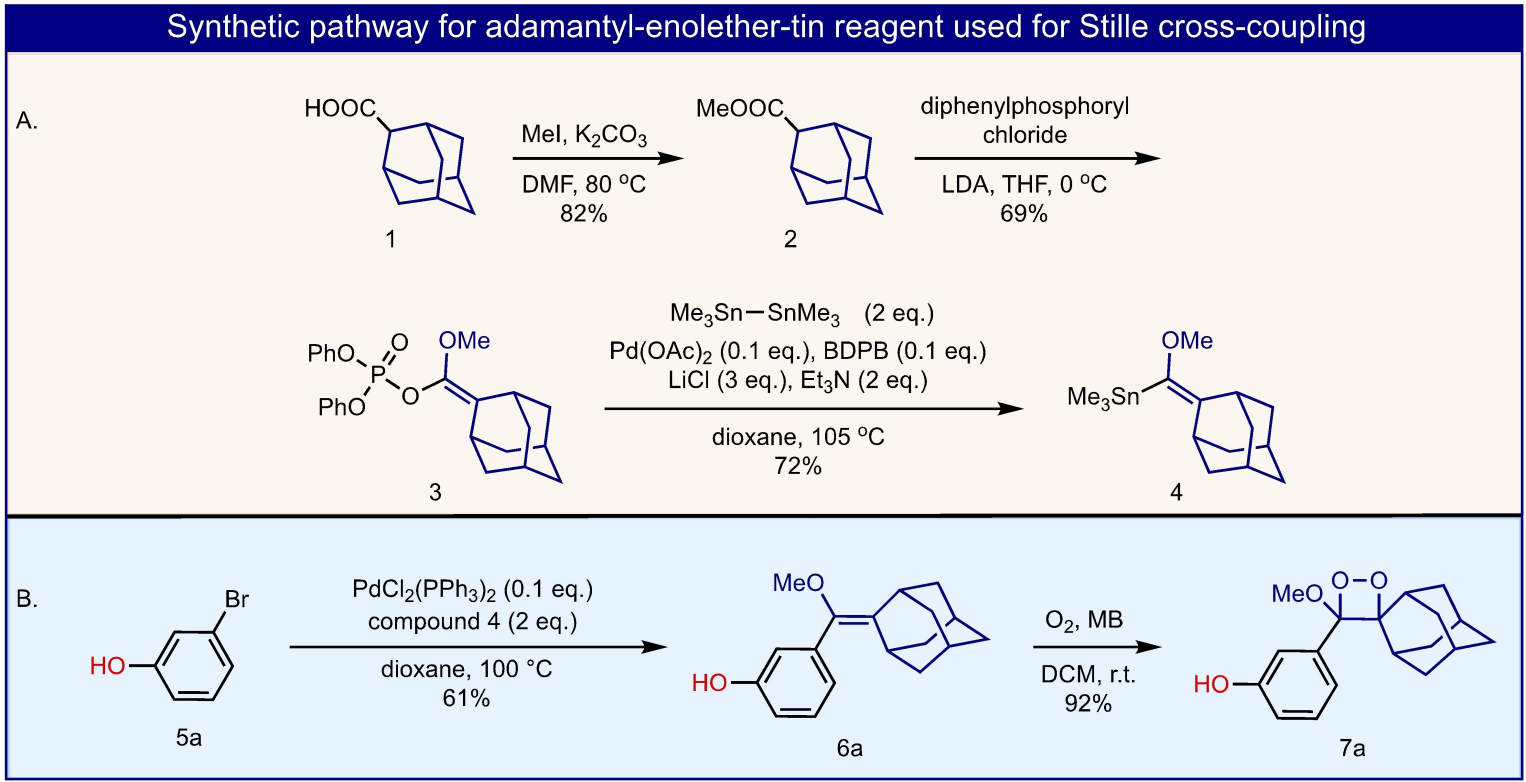
A) Chemical synthetic pathway for preparation of adamantyl‐enolether‐tin **4**. B) Stille cross‐coupling reaction of *meta*‐bromo phenol (**5** **a**) with reagent **4**, followed by oxidation with singlet oxygen to form Schapp's 1,2‐dioxetane.

The Stille cross‐coupling reaction is one of the most efficient transformations to achieve C−C bond formation, with high chemo‐selectivity and tolerance towards various functional groups.^[28]^ Therefore, initially, we sought to develop a simple synthetic procedure for an adamantyl‐enolether‐tin reagent. Such a reagent should be suitable for a general Stille cross‐coupling reaction with diverse aryl‐halides. The synthesis of this reagent (adamantyl‐enolether‐tin **4**), was achieved as presented in Figure 3A. Commercially available 2‐adamantyl‐carboxylic acid **1**, was reacted with iodomethane to provide methyl‐ester **2**. The later was deprotonated by LDA to generate the corresponding enolate, which was subsequently reacted with diphenylphosphoryl chloride to furnish phosphate‐ester **3**. Palladium‐catalyzed reaction of the phosphate‐ester with hexamethydiltin, using 1,2‐bis‐(diphenylphosphino)‐butane (BDPB) ligand, afforded the adamantyl‐enolether‐stannane **4**.

In order to evaluate the feasibility of this Stille reagent in a cross‐coupling reaction for the synthesis of adamantyl‐phenoxy‐enolethers, we used *meta*‐bromo phenol **5** **a** as a model substrate (Figure 3B). The latter was successfully reacted with organostannane **4**, under standard Stille conditions, to directly derivatize its *meta*‐bromo position and to afford enolether **6** **a** in 61 % yield. Subsequently, enolether **6** **a** was successfully oxidized with singlet oxygen to afford the desired Schapp's 1,2‐dioxetane product (**7** **a**). Notably, this transformation was achieved without the need to protect the phenol functional group. With the adamantyl‐enolether‐tin reagent in hand, the synthesis of chemiluminescent luminophore **7** **a** is efficiently achieved in only two simple steps.

**Figure 4:**
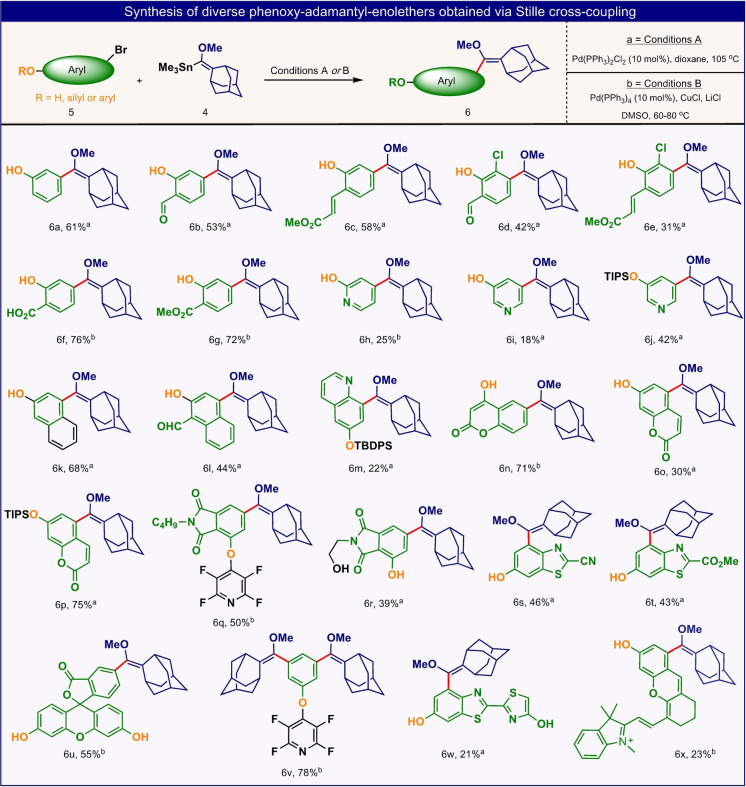
Selected examples of phenoxy adamantyl‐enolethers obtained via the Stille cross‐coupling reaction from their corresponding aryl‐bromides (Reaction conditions A or B are both suitable for non‐polar substrates. For polar substrates conditions B are preferred).

**Figure 5:**
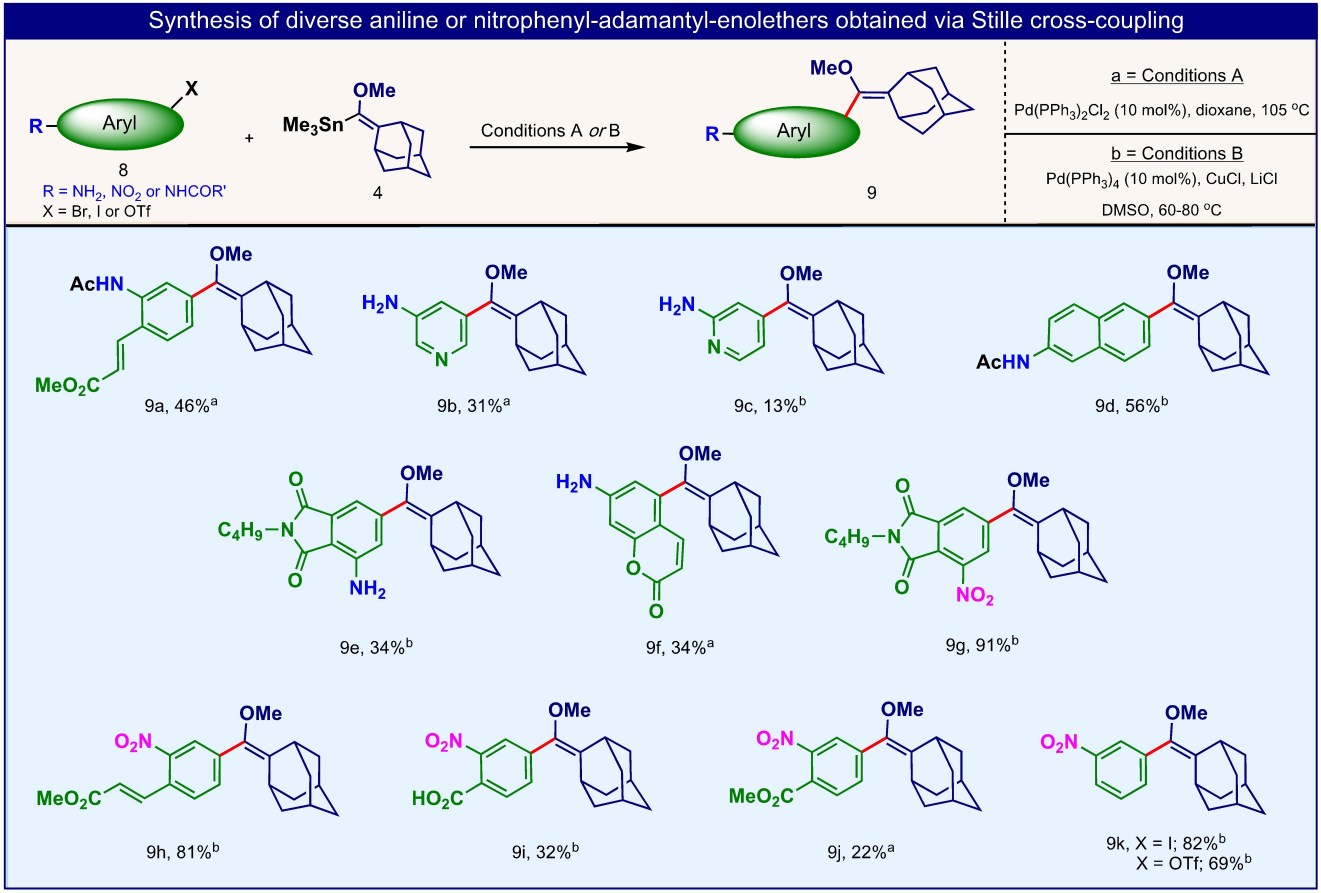
Selected examples of aniline or nitrophenyl adamantyl‐enolethers obtained via the Stille cross‐coupling reaction from their correspondent aryl‐halide/triflate (Reaction conditions A or B are both suitable for non‐polar substrates. For polar substrates conditions B are preferred).

Encouraged by these results, we next applied this synthetic strategy to numerous aryl‐bromides to directly obtain the corresponding adamantyl‐enolethers (Figures 4 and 5). These precursors could subsequently be oxidized by singlet oxygen to form the desired 1,2‐dioxetane chemiluminescent luminophores.

Indeed, various sub‐groups of hydroxy‐aryl‐bromides could be effectively derivatized via the Stille cross‐coupling reaction, with module **4**, to produce their adamantyl‐enolethers counterparts in a single step (Figure 4). The substrate‐scope for this functionalization was extended far beyond typical *meta*‐bromo‐phenols. For example, phenoxy‐enolethers with coumarin, fluorescein or cyanin aryl cores (compounds **6** **o**, **6** **u** and **6** **x**) were directly obtained from their bromo‐phenoxy substrates. Such enolethers are precursors for various dioxetane luminophores that incorporate known fluorogenic dyes in their molecular scaffolds. In addition, aniline‐enolethers (compounds **9** **a**–**9** **f**) could be easily prepared by direct derivatization of *meta*‐bromo‐anilines (Figure 5). Aniline‐dioxetanes can undergo chemiexcitation in analogous manner to that occurs with phenoxy‐dioxetanes. Nitro‐halo/triflate‐aryl compounds, as an aniline precursor, could also efficiently react via Stille cross‐coupling route to afford their corresponding enolethers (Figure 5, compounds **9** **g**–**9** **k**).

Hetero‐aryl‐bromides were used to synthesize hydroxy or amino‐pyridine‐enolethers and other complex enolether precursors of dioxetanes luminophores. In most cases, the aryl‐bromides could be used for the Stille cross‐coupling reaction, where their phenol or aniline functional groups are unprotected. However, in some cases protecting groups were required, such as when the unprotected substrates suffered from low solubility. In a unique example, dibromo protected phenol could be employed, as a model substrate, to simultaneously derivatize two bromo‐aryl sites via double Stille cross‐coupling. This transformation afforded the first phenoxy‐di‐enolether (compound **6** **v**) as a precursor for a dioxetane luminophore that can potentially undergo two consecutive chemiexcitation events, from the same oxy‐phenolate. Two other interesting examples, that are described in detail below, were obtained by preparing the enolether derivatives based on 7‐hydroxy‐coumarin and oxyluciferin dyes (compounds **6** **o** and **6** **w**).

The Stille cross‐coupling reaction of module **4** with substrates other than aryl‐bromides was also evaluated. While a general aryl‐iodide and aryl‐triflate reacted efficiently with module **4** to give their corresponded enol‐ether products, aryl‐chloride could not react at all (see Supporting Information).

**Figure 6:**
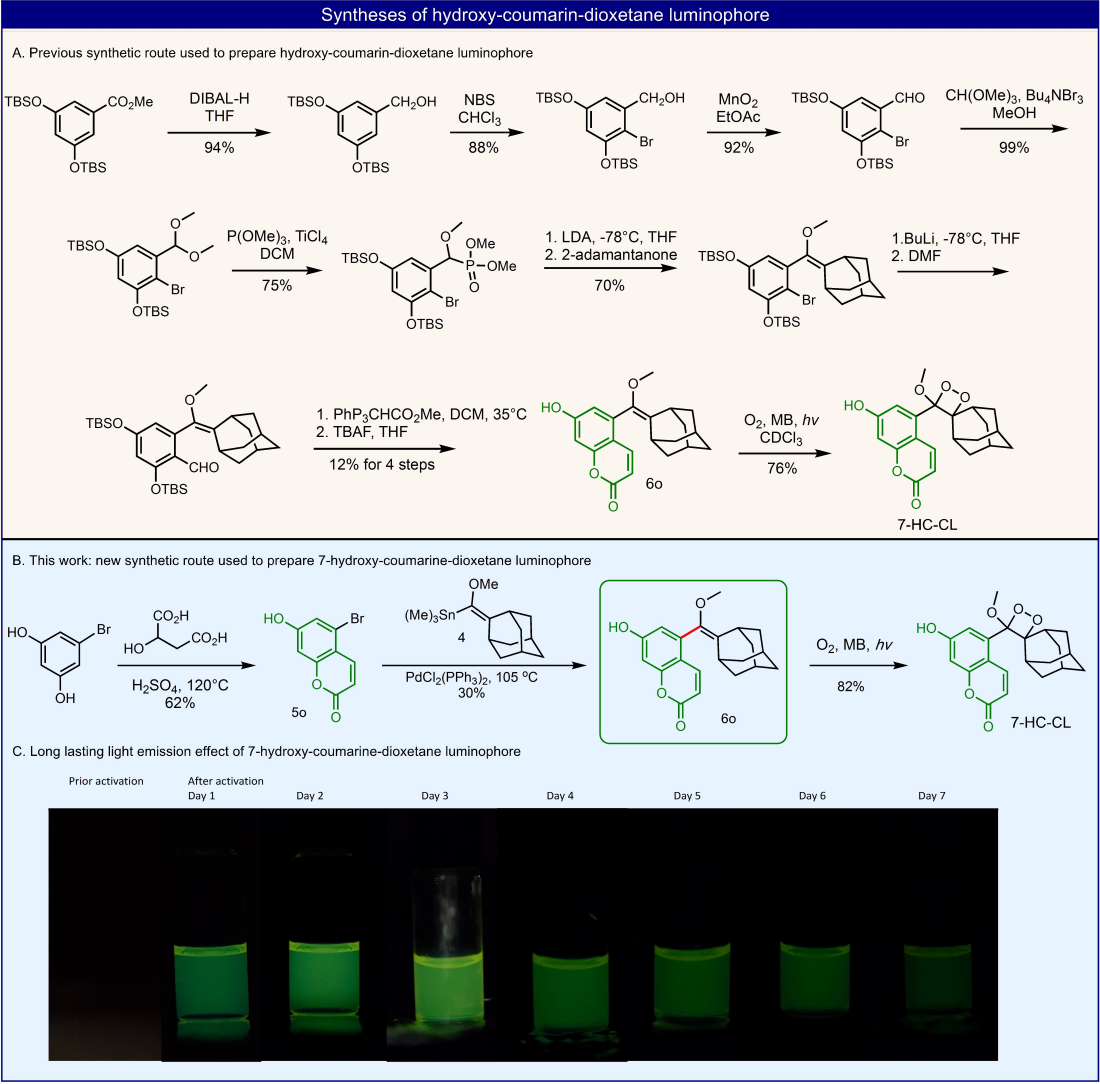
A) Synthesis of 7‐hydroxycoumarin‐dioxetane based on the traditional synthetic method. B) Short efficient synthesis of 7‐hydroxycoumarin‐dioxetane using Stille cross‐coupling reaction. C) Long‐lasting light emission effect observed by 7‐hydroxycoumarin‐dioxetane luminophore (10 mM in DMSO, activation is performed by addition of TBAF).

In order to further demonstrate the advantages of this synthetic route, five important examples that either significantly shorten the synthesis or provide access to highly challenging new luminophores were exemplified. The first of these, concerns with the synthesis of a highly efficient dioxetane luminophore based on a coumarin scaffold. 7‐Hydroxycoumarin‐dioxetane **7**‐**HC**‐**CL**, is a luminophore that exhibits intense glow chemiluminescence; it undergoes very slow chemiexcitation, and it has the highest chemiluminescence quantum yield ever reported under physiological conditions (Φ_CL_=55 %). This dioxetane luminophore was recently prepared by our group, using the traditional Horner–Wittig‐based synthetic route (Figure 6A, 11 step synthesis), with an overall yield of 4 %.^[29]^ In contrast, by applying the new Stille cross‐coupling route, the synthesis of this dioxetane luminophore was achieved by only three synthetic steps with an overall yield of 43 % (Figure 6B). Bromo‐hydroxycoumarin **5** **o** was prepared in one step by reaction of 5‐bromoresorcinol with 2‐hydroxy‐butanedioic acid. Next, bromide **5** **o** was directly reacted with tin reagent **4**, via Stille cross‐coupling reaction, to give the enolether precursor **6** **o**, which was subsequently oxidized to afford the 7‐hydroxycoumarin‐dioxetane **7**‐**HC**‐**CL**. It is worth noting, that performing the Stille coupling on the silyl protect hydroxyl coumarin revealed higher yield (75 %) and could be easily scaled up to gram scale to afford enolether **6** **p** (see Supporting Information).

The long‐lasting light emission effect of **7**‐**HC**‐**CL** luminophore can be clearly visualized over seven days (Figure 6C). Overall, this example effectively demonstrates how the synthesis of such a dioxetane‐luminophore, can be significantly improved by reducing the number of synthetic steps from eleven to only three.

**Figure 7:**
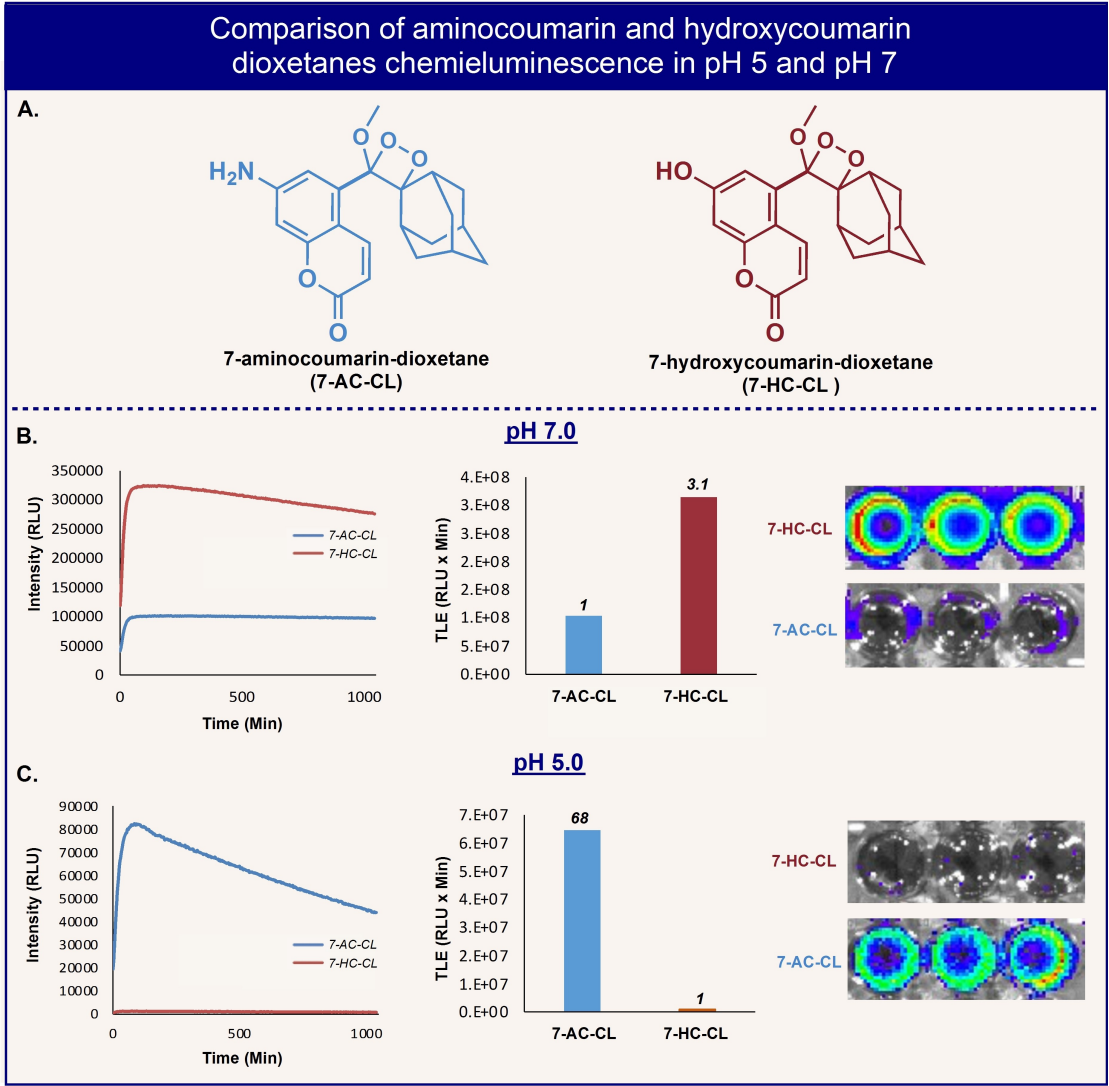
A) Chemical structures of amino‐ and hydroxy‐coumarin dioxetanes. B) Chemiluminescence kinetic profiles and total light emission of 7‐amino‐ and 7‐hydroxy‐coumarin dioxetanes [10 μM] in PBS (pH 7.0) at 37 °C and chemiluminescence images of 7‐amino‐ and 7‐hydroxy‐coumarin dioxetane [50 μM] in buffer solution (pH=7.0), 10 % DMSO at 37 °C. C) Chemiluminescence kinetic profiles and total light emission of 7‐amino‐ and 7‐hydroxy‐coumarin dioxetanes [10 μM] in PBS (pH 5.0) at 37 °C and chemiluminescence images of 7‐amino‐ and 7‐hydroxy‐coumarin dioxetane [50 μM] in buffer solution (pH=5.0), 10 % DMSO at 37 °C. Chemiluminescence images of 7‐amino‐ and 7‐hydroxy‐coumarin dioxetane were taken using IVIS® Lumina, images were acquired directly after the addition of the dioxetanes, with an exposure time of 3 min.

In the second example, we present the light‐emission properties of a new 7‐aminocoumarin‐dioxetane luminophore (**7**‐**AC**‐**CL**), which was prepared by oxidation of enolether precursor **9** **f** (Figure 5), synthesized via the Stille cross‐coupling route from its parent bromo‐arene (see Supporting Information). Such a dioxetane luminophore has never been prepared before. Figure 7 shows the chemiluminescence emission profiles of 7‐aminocoumarin‐dioxetane **7**‐**AC**‐**CL** vs. that of 7‐hydroxycoumarin‐dioxetane **7**‐**HC**‐**CL**. While both luminophores exhibit long‐lasting light‐emission profiles in aqueous pH 7.0 buffer, the total light emission of **7**‐**HC**‐**CL**, was about 3‐fold higher than that of **7**‐**AC**‐**CL** (Figure 7B). However, in aqueous pH 5.0 buffer, **7**‐**HC**‐**CL** exhibited very low light emission signal. Remarkably, **7**‐**AC**‐**CL** generated 68‐fold stronger light emission signal than that produced by **7**‐**HC**‐**CL** at this acidic pH (Figure 7C). This observation can be explained by protonation of the phenolate of **7**‐**HC**‐**CL** (p*K*_a_≈7) in aqueous pH 5.0 buffer, which prevents its chemiexcitation. In contrast, the amino group of aniline **7**‐**AC**‐**CL**, is not protonated at pH 5.0 and therefore, can still initiate chemiexcitation. In order to demonstrate the feasibility of these two dioxetane luminophores to provide chemiluminescence images with different contrast under neutral and acidic pH, solutions of the two luminophores were imaged with an IVIS imaging system. Figures 7B, C clearly show the image contrast obtained for solutions of **7**‐**HC**‐**CL** and **7**‐**AC**‐**CL** in neutral and acidic pHs. The light emission properties of a chemiluminescence luminophore like dioxetane **7**‐**AC**‐**CL**, are particularly advantageous for detection and imaging applications, performed in biological environment with acidic pH, such as the endosomal‐lysosomal system.^[30]^

**Figure 8:**
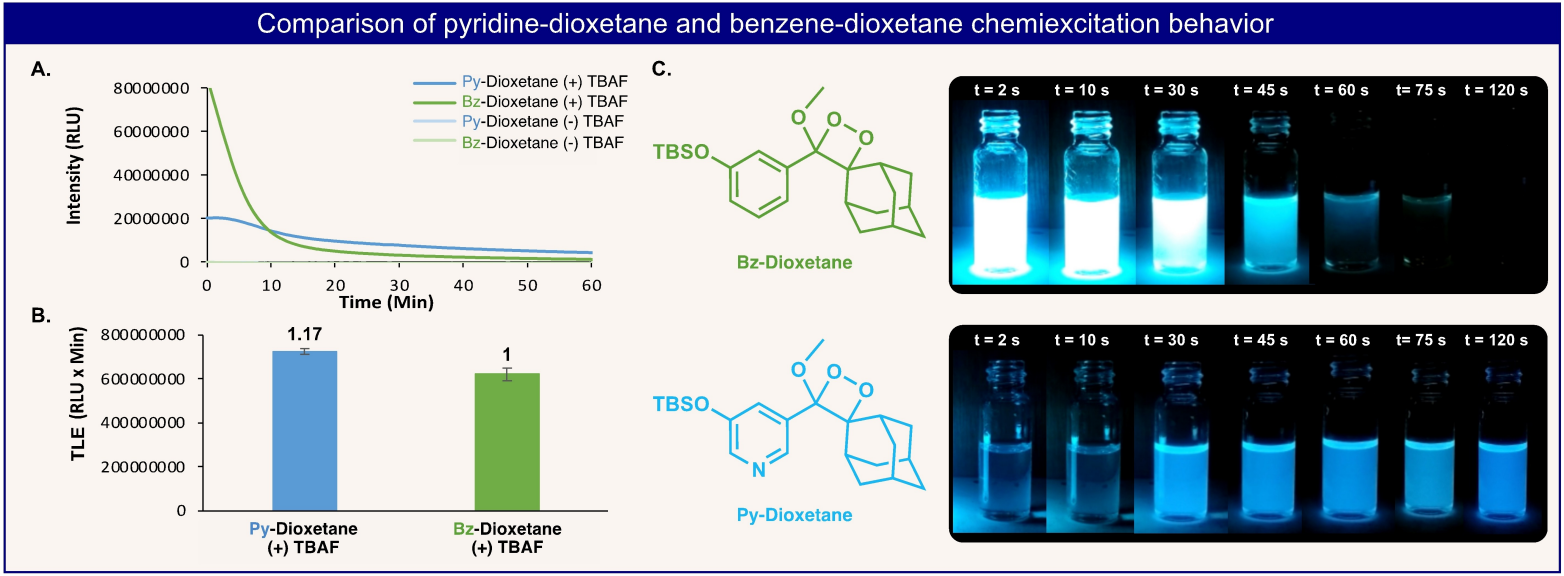
A) Chemiluminescence kinetic profile of **Py**‐**dioxetane** and **Bz**‐**dioxetane** [10 μM] in DMSO in the presence and absence of TBAF [100 μM]. B) Total light emitted from **Py**‐**dioxetane** and **Bz**‐**dioxetane** [10 μM] during 60 minutes in the presence of [100 μM] TBAF in DMSO. C) Visual comparison of the light emitted by **Py**‐**dioxetane** and **Bz**‐**dioxetane** [1 mM] during 120 seconds in the presence of [10 mM] TBAF in DMSO.

In the third example, we sought to determine the light emission profile of the first pyridine analog of the classic benzene‐based Schaap's dioxetane (Figure 8). The enolether precursor of the **Py**‐**dioxetane** was directly synthesized by one step through the Stille cross‐coupling route, starting from commercially available 3‐bromo‐5‐hydroxy pyridine (Figure 4, compound **6** **i**). The hydroxyl functional group was masked with a silyl‐ether (TBS), as a responsive group that can be removed by fluoride. The light emission kinetic profile of the new **Py**‐**dioxetane** was compared with that of the **Bz**‐**dioxetane** of Schapp. The **Bz**‐**dioxetane** exhibited a strong burst of light emission signal, upon addition of TBAF, which lasted for a short time and almost completely turned off after 10 min. In contrast, the intensity of the light emission signal of the **Py**‐**dioxetane** was weaker and continued for more than 60 min (Figure 8A). Interestingly, the total light emission measured for the **Bz**‐**dioxetane** and for the **Py**‐**dioxetane**, was almost identical (Figure 8B). The substantial difference, between the kinetics of the light‐emission profiles, was visually demonstrated in a set of images taken over several time intervals (Figure 8C). The observed kinetic effect can be explained by a relatively slow chemiexcitation process, which occurs for the hydroxy‐pyridine‐dioxetane versus the hydroxy‐benzene‐dioxetane analog. The electron‐withdrawing effect of the pyridine ring decreases its chemiexcitation rate, and thus leads to a long‐lasting light‐emission profile with reduced signal intensity.

**Figure 9:**
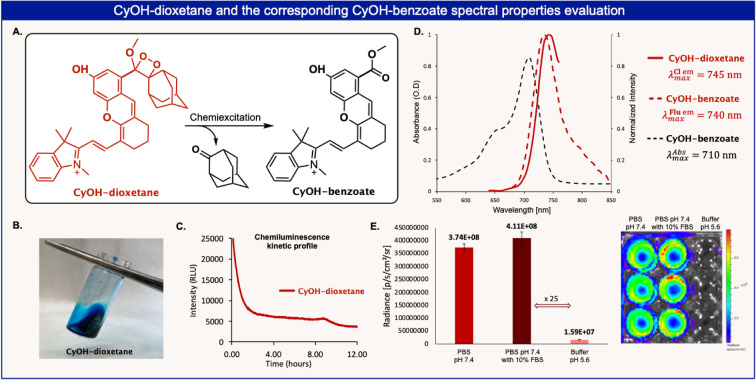
A) Chemiexcitation pathway of **CyOH**‐dioxetane. B) Image of a solution of CyOH‐dioxetane [100 μM] in CHCl_3_. C) Chemiluminescence kinetic profile of **CyOH**‐dioxetane [100 μM] in 1 : 1 mixture of phosphate buffers (pH 7.4) and DMSO at 25 °C. D) Absorbance and fluorescence emission spectra of **CyOH**‐benzoate and chemiluminescent emission spectra of **CyOH**‐dioxetane [100 μM] in 1 : 1 mixture of phosphate buffers (pH 7.4) and DMSO. E) Chemiluminescence images of **CyOH**‐dioxetane solution in PBS pH 7.4, PBS pH 7.4+10 % FBS and buffer pH 5.6. Chemiluminescence images were taken using IVIS® Lumina, images were acquired directly after the addition of **CyOH**‐dioxetane with exposure time of 3 min.

In order to use dioxetane chemiluminescence luminophores for in vivo imaging, it is essential to obtain better emitter species in water and to shift their light emission wavelength toward the near‐infrared (NIR) region. This region is considerably more useful for in vivo imaging, since NIR wavelengths can better penetrate and are less scattered by living tissues. The fourth example of a dioxetane luminophore, which utilized our efficient Stille cross‐coupling synthetic route, deals with the synthesis and light‐emission properties of a new chemiluminescence dioxetane NIR luminophore (Figure 9). The bromo‐arene derivative, of Lin's NIR‐fluorescent cyanin,^[31]^ was simply prepared by two steps as described in the Supporting Information. Stille cross‐coupling reaction of this bromo‐arene with stannane‐enolether **4**, directly afforded the adamantyl‐enolether **6** **x** (see Figure 4 for chemical structure). The latter was oxidized with singlet oxygen to give the chemiluminescence NIR luminophore, **CyOH**‐dioxetane. The chemiexcitation disassembly pathway of **CyOH**‐dioxetane generates the excited‐state form of **CyOH**‐benzoate derivative, which decays to its ground‐state through the emission of an NIR‐photon (Figure 9A). The chemiluminescent kinetic profile of **CyOH**‐dioxetane exhibited long‐lasting light‐emission signal over more than 12 h (Figure 9C). In order to provide a solid support for the NIR‐chemiluminescent‐emission of the **CyOH**‐dioxetane luminophore, we measured the fluorescent‐emission spectrum **CyOH**‐benzoate and compared it to that of the chemiluminescent‐emission spectrum produced by the chemiexcitation of **CyOH**‐dioxetane. As can be seen in Figure 9D, the NIR‐fluorescent emission spectrum of **CyOH**‐benzoate is almost completely overlapping with the NIR‐chemiluminescent‐emission spectrum of **CyOH**‐dioxetane luminophore. To provide a prove of concept for the feasibility the **CyOH**‐dioxetane luminophore to serve as an imaging probe for in vivo use, chemiluminescence images of the luminophore in solution were recorded, using an IVIS imaging system, under physiological conditions (PBS 7.4). Images could also be obtained in the presence of fetal bovine serum (FBS). Control image of **CyOH**‐dioxetane in buffer pH 5.6 showed no light‐emission at all. This example opens a doorway for synthesis of new dioxetane luminophores with various light‐emission wavelengths. Such dioxetane luminophores are particularly important for applications required multiplex chemiluminescent assays.^[32]^

**Figure 10:**
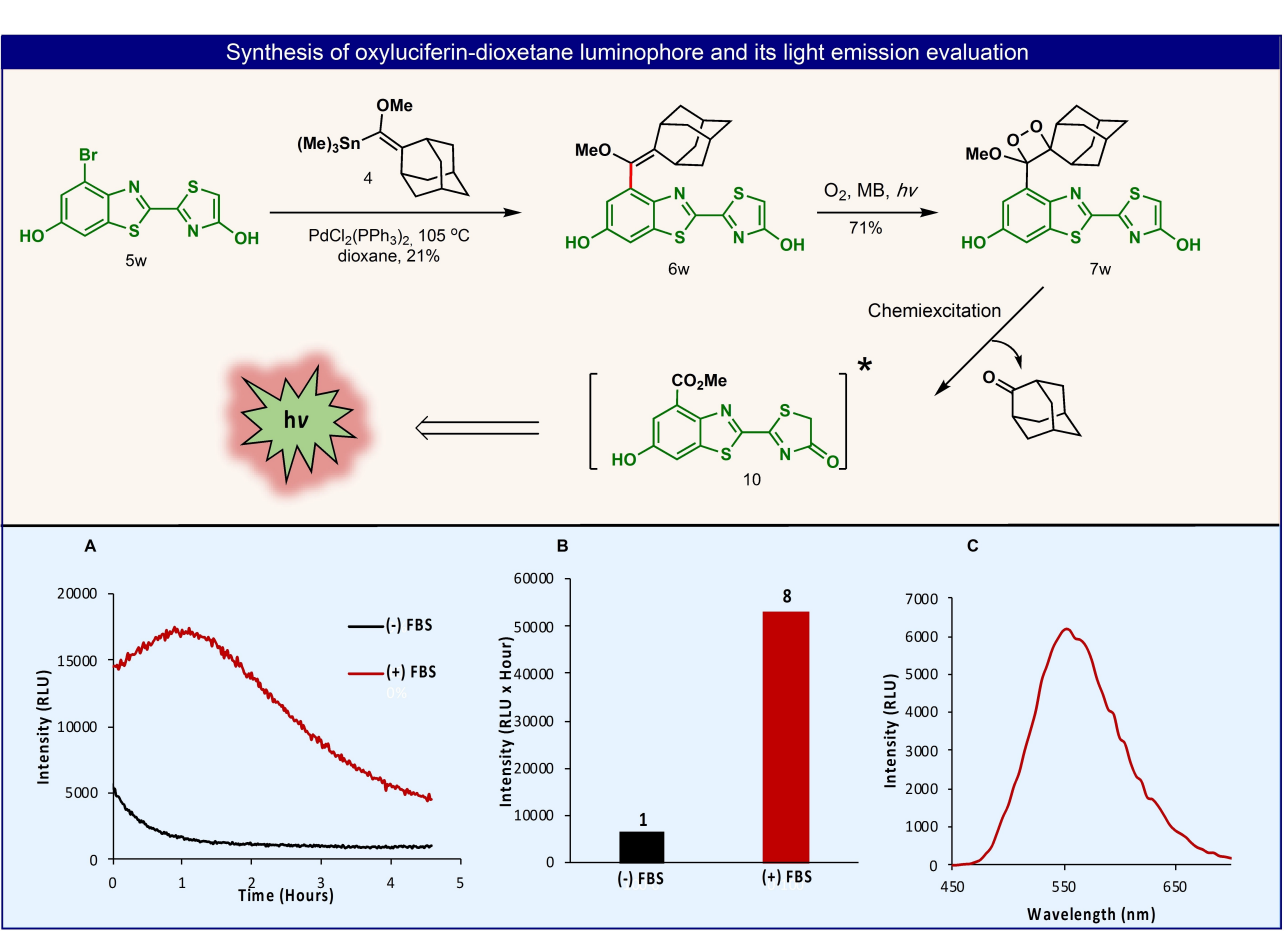
2‐Step synthesis of oxyluciferin‐dioxetane via Stille cross‐coupling and its chemiexcitation pathway. A) Chemiluminescence kinetic profiles of oxyluciferin‐dioxetane [10 μM] in PBS 7.4 and in PBS 7.4+5 % FBS, B) total light emission, C) chemiluminescence emission spectrum of oxyluciferin dioxetane [10 μM] with 5 % of FBS, in PBS, pH 7.4 at room temperature.

The last example demonstrates how the Stille cross‐coupling route enables a concise synthesis of the relatively complex adamantyl‐dioxetane of oxyluciferin. The latter is the active species, responsible for the light emission phenomenon of the firefly bioluminescence.^[33]^ This natural marvel has strongly inspired scientists and has motivated extensive studies due to its widespread applications.^[34]^ Oxyluciferin is generated in its excited‐state after oxidation of D‐luciferin by molecular oxygen, in the presence of magnesium ions and adenosine triphosphate. The luciferase enzyme, that catalyzes this reaction, also provides a specific hydrophobic protein pocket that locks the oxyluciferin molecule in a planar conformation and thereby, enhancing the emissive nature of this excited intermediate.^[35]^ Incorporation of an adamantyl‐dioxetane at a *meta*‐position, relative to the phenol, of the oxyluciferin molecule, would fabricate a luminophore that can undergo direct chemiexcitation, with no involvement of any additive (Figure 10, compound **7** **w**). The synthesis of such molecule can be simply achieved by direct functionalization of the bromo‐oxyluciferin **5** **w** with module **4**, following by oxidation with singlet oxygen. This case‐study inspired us to synthesize and evaluate the light emission properties of such oxyluciferin luminophores in the presence of hydrophobic proteins. Bromo‐oxyluciferin **5** **w** was synthesized as reported before.^[36]^ Stille cross‐coupling reaction of this aryl‐bromide with module **4**, afforded enolether **6** **w** directly with no protecting groups. Oxidation of the latter by singlet oxygen gave the desired adamantyl‐dioxetane of oxyluciferin **7** **w** (Figure 6). The light emission properties of this luminophore were then evaluated in PBS 7.4, in the absence and the presence of Fetal Bovine Serum (FBS). Indeed, upon chemiexcitation of the dioxetane oxyluciferin luminophore, green light emission was observed with a wavelength maximum of 550 nm (Figure 10C). The light emission intensity observed for this oxyluciferin luminophore was relatively weak upon its chemiexcitation in PBS 7.4 solely. However, in presence of FBS, the intensity of the emitted light was enhanced by a factor of 8‐fold (Figure 10A, B). This observation can be explained by the host‐guest interaction of the oxyluciferin **10** excited‐state with Bovine Serum Albumin (BSA), the predominate protein of FBS. BSA is known to encapsulate lyophilic molecules in its hydrophobic pocket. Such an interaction could be used to study and mimic the binding relationships of the natural oxyluciferin excited‐state with its native enzyme, D‐luciferin.^[37]^

**Figure 11:**
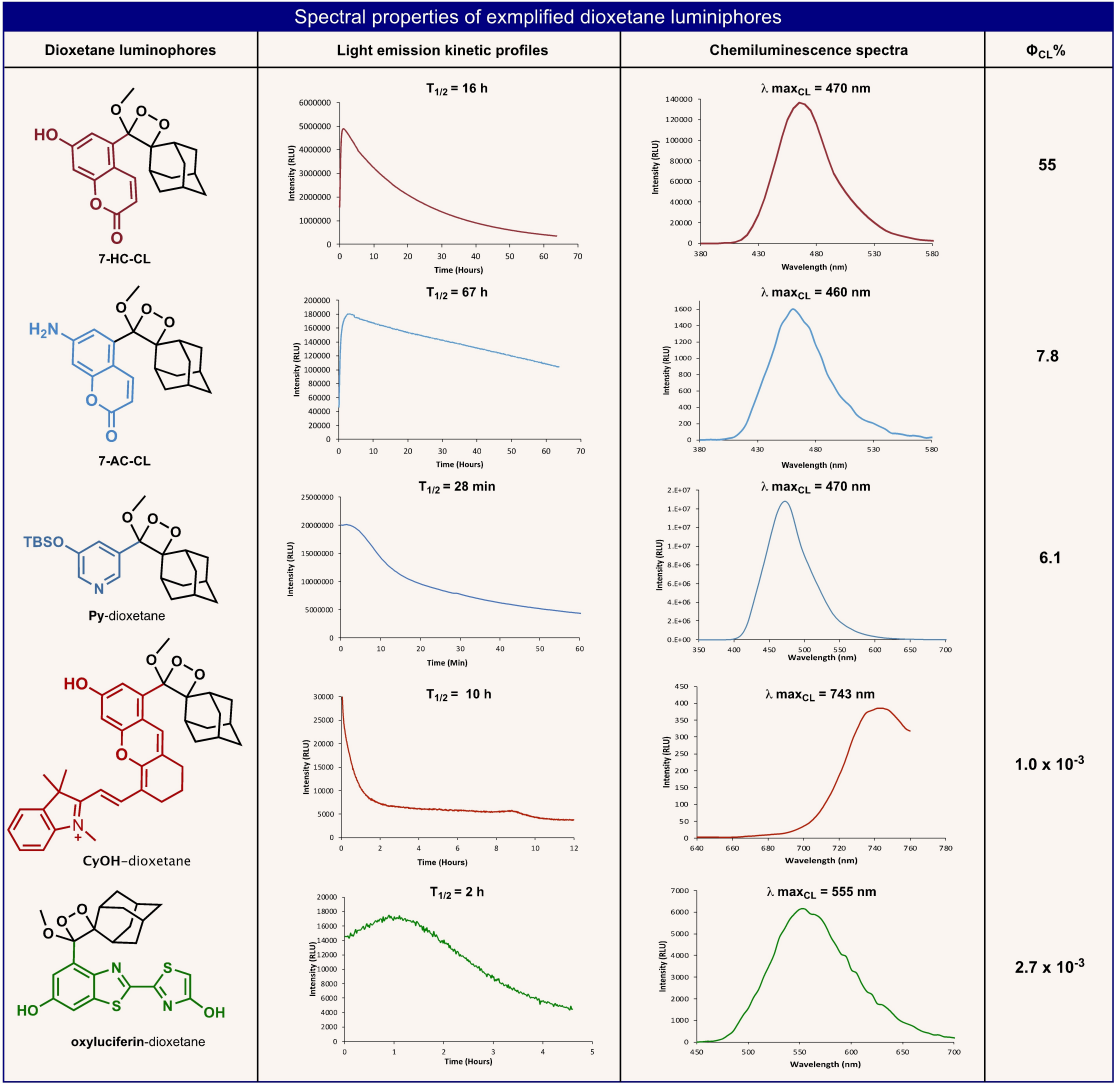
Spectral properties and characterizations of synthesized dioxetane luminophores. Light emission kinetic profiles and chemiluminescence emission spectra of each of the above mentioned dioxetanes was measured in the following conditions: **7**‐**HC**‐**CL** and **7**‐**AC**‐**CL** [1 μM] in PBS (pH 7.0). Py‐dioxetane [10 μM], TBAF [100 μM] in DMSO. CyOH‐dioxetane [100 μM] in 1 : 1 mixture of PBS (pH 7.4) and DMSO. Oxyluciferin‐dioxetane [10 μM] in PBS (pH 7.4)+5 % FBS. Φ_CL_ was calculated in comparison to that of **7**‐**HC**‐**CL**.^[29]^ *T*_1/2_ value was defined as the time taken to emit half of the maximal total light emitted.

Overall, we have showcased five examples of new dioxetane chemiluminescence luminophores, prepared by our late‐stage derivatization strategy, and analyzed their light‐emission profiles. The spectral characterizations of these luminophores are concisely presented in Figure 11. Chemiluminescence luminophores like, 7‐amino‐coumarin dioxetane (**7**‐**AC**‐**CL**), 7‐hydroxy‐coumarin dioxetane (**7**‐**HC**‐**CL**) and **Py**‐dioxetane are very stable and could be stored for long use. However, chemiluminescence luminophores like, **CyOH**‐dioxetane and oxyluciferin dioxetane are highly undatable and must be used immediately after the oxidation of their parent enolethers by singlet oxygen. The instability of some dioxetane compounds with relatively long conjugated π‐electron systems could be explained by a mechanism of light‐induced decomposition. This mechanism involves electron transfer from the LUMO of the excited fluorophore to the antibonding σ* orbital of O−O peroxide bond and thereby, results in bond cleavage and subsequent decomposition of the dioxetane into a benzoate derivative and adamantanone.

Despite its immense potential, chemiluminescence, as a general diagnostic and imaging modality, is still in its infancy and is rarely used outside of test‐tube immunoassays. The new convergent approach disclosed herein enables the development of a myriad of next‐generation chemiluminescence compounds. Such a late‐stage derivatization strategy simplifies the rapid exploration of novel luminogenic molecular structures in a library format. The approach capitalizes on the classic Stille reaction, which demonstrates a high degree of functional group tolerance and that can be performed under mild conditions. Importantly, it enables access to unique constructs that cannot be accessed easily by the current available approaches. The introduction of a new chemical reagent (module **4**) allows one to directly tag the adamantyl‐enolether group toward the final step of the synthesis, thus significantly simplifying the preparation of 1,2‐dioxetane‐based chemiluminescent luminophores. The availability of new dioxetane luminophores with unmatched efficiencies could significantly advance this field and enable the use of chemiluminescence in new applications.

## Conclusion

In summary, we have developed a new efficient synthetic route for preparation of adamantyl‐dioxetane enolether precursors. The synthesis is based on a late‐stage functionalization of aryl‐halides with stannane‐enolether, achieved by the Stille cross‐coupling reaction, to directly afford adamantyl‐enolether. In a following step, the dioxetane is obtained by simple oxidation of the enolether precursor with singlet‐oxygen. The scope of this synthetic route is wide since a large number of haloarenes are either commercially available or easily accessible. Numerous bromoarenes were successfully reacted, by the Stille cross‐coupling reaction, to afford their correspondent enolether derivatives. This new synthetic route has enabled us to significantly shorten the synthesis of known dioxetane luminophores and to provide easy access for the preparation of highly challenging new luminophores. Light‐emission properties of five new interesting dioxetane luminophores, prepared by the synthetic route developed in this work, are comprehensively exemplified. We expect that this new synthetic strategy would be particularly useful in the design and synthesis of yet unexplored dioxetane chemiluminescent luminophores.

## Conflict of interest

The authors declare no conflict of interest.

1

## Supporting information

As a service to our authors and readers, this journal provides supporting information supplied by the authors. Such materials are peer reviewed and may be re‐organized for online delivery, but are not copy‐edited or typeset. Technical support issues arising from supporting information (other than missing files) should be addressed to the authors.

Supporting Information

## Data Availability

The data that support the findings of this study are available in the supplementary material of this article.
