# Supplementary material for: Modular Access to Diverse Chemiluminescent Dioxetane‐Luminophores through Convergent Synthesis
Source: Angew Chem Int Ed Engl. 2022 Mar 29;61(22):e202202187. doi: 10.1002/anie.202202187 (PMC9311660; doi:10.1002/anie.202202187)
Supplement: Supplementary file 1 — Supporting Information [file ANIE-61-0-s001.pdf]

Angewandte Chemie
Eine Zeitschrift der Gesellschaft Deutscher Chemiker

Supporting Information

Modular Access to Diverse Chemiluminescent Dioxetane-Luminophores through Convergent Synthesis

S. Gnaim, S. P. Gholap, L. Ge, S. Das, S. Gutkin, O. Green, O. Shelef, N. Hananya, P. S. Baran, D. Shabat**

Supporting Information

Sr. No.	Topic	Page no.
1	General information	02
2	Synthetic schemes and experimental procedures	03
3	NMR and MS Spectra	31
4	References	120

1. General Information

All reactions requiring anhydrous conditions were performed under an Argon atmosphere. All reactions were carried out at room temperature unless stated otherwise. Chemicals and solvents were either A.R. grade or purified by standard techniques. Thin-layer chromatography (TLC): silica gel plates Merck 60 F254: compounds were visualized by irradiation with UV light. Column chromatography (FC): silica gel Merck 60 (particle size 0.040-0.063 mm), eluent given in parentheses. Reverse-phase high-pressure liquid chromatography (RP-HPLC): C18 5u, 250x4.6mm, eluent given in parentheses. Preparative RP-HPLC: C18 5u, 250x21mm, eluent given in parentheses. ^{1}H-NMR spectra were measured using Bruker Avance operated at 400 MHz. ^{13}C-NMR spectra were measured using Bruker Avance operated at 101 MHz. Chemical shifts were reported in ppm on the δ scale relative to a residual solvent ($CDCl_3$: δ = 7.26 for ^{1}H-NMR and 77.16 for ^{13}C-NMR, DMSO-d_6: δ = 2.50 for ^{1}H-NMR and 39.52 for ^{13}C-NMR). Mass spectra were measured on Waters Xevo TQD. Chemiluminescence was recorded on Molecular Devices Spectramax i3x and SpectraMax M5 plate reader. Fluorescence was recorded on Tecan infinite 200 Pro. All general reagents, including salts and solvents, were purchased from Sigma-Aldrich. Light irradiation for photochemical reactions: LED PAR38 lamp (19W, 3000K). 1-Adamantanecarboxylic acid and hexamethylditin were obtained from Biosynth-Carbosynth.

Abbreviations

ACN - Acetonitrile, **CDI** - 1,1'-Carbonyldiimidazole, **DCC** - *N*, *N*'-Dicyclohexylcarbodiimide, **DCM** - dichloromethane, **DIPEA** - *N*,*N*-Diisopropylethylamine, **DMF** - *N*,*N*'-Dimethylformamide, **DMBA** - Dimethylbarbituric acid, **DMAP** - 4-(Dimethylamino)-pyridine, **DMSO** - Dimethyl sulfoxide, **EDC** - 1-Ethyl-3-(3-dimethylaminopropyl)-carbodiimide, **EEDQ** - N-Ethoxycarbonyl-2-ethoxy-1,2-dihydroquinoline, **EtOAc** - Ethylacetate, **HBTU** - 2-(1H-Benzotriazol-1-yl)-1,1,3,3-tetramethyluronium hexafluorophosphate, **Hex**- Hexane, **TFA** - Trifluoroacetic acid, **TEA** - Triethylamine, **THF** - Tetrahydrofuran, **TMSCl** - Trimethylsilyl chloride, **PBS** - Phosphate-buffered saline.

2. Synthetic Schemes and Experimental Procedures

Scheme 1. Synthesis of Tin-adamantyl reagent 4.

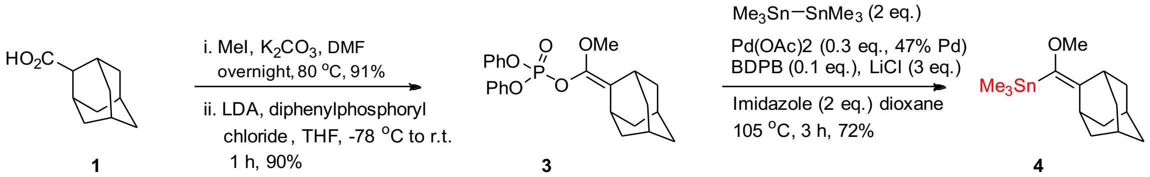

Compound 3:

To a solution of compound **1** (10 g, 55.5 mmol, 1 eq.) in DMF (50 ml), K_2CO_3 (15.36 g, 111.1 mmol, 2 eq.) and iodomethane (8.64 ml, 138.8 mmol, 2.5 eq.) were added. The reaction mixture was heated to 80 °C for overnight. After completion, the solution was diluted in EtOAc (1 L) and washed with brine (2 × 200 mL) and water (2 × 200 mL). The organic layer was dried over Na_2SO_4 and evaporated under reduced pressure. The crude residue was purified by column chromatography on silica gel (Hex:EtOAc 90:10) to afford methyl adamantane-1-carboxylate **2** as a pale yellow oil (9.823 g, 91% yield). ^{1}H NMR (400 MHz, $CDCl_3$) δ 3.69 (s, 3H), 2.61 (s, 1H), 2.33 (s, 2H), 1.93 – 1.56 (m, 12H). ^{13}C NMR (101 MHz, $CDCl_3$) δ 175.26, 51.44, 49.73, 38.28, 37.55, 33.72, 29.71, 27.63, 27.58.

Methyl adamantane-1-carboxylate **2** (5 g, 25.77 mmol, 1 eq.) was dissolved in anhydrous THF (50 mL) and cooled to -78 °C under argon. Lithium diisopropylamide (2 M in THF, 15.463 mL, 30.93 mmol, 1.2 eq.) was added dropwise (around 10 min) and the dark brown solution was allowed to warm to room temperature and stirred for 30 minutes. The reaction mixture was cooled again to -78 °C and diphenylphosphoryl chloride (6.944 mL, 33.51 mmol, 1.3 eq.) was added. The reaction was allowed to warm to room temperature and stirred for 60 minutes. After completion, the solution was diluted with EtOAc (500 mL) and washed with NH_4Cl, brine, and water. The organic layer was dried over Na_2SO_4 and evaporated under reduced pressure. The residue was purified by column chromatography on silica gel (Hex:EtOAc 75:25) to afford compound **3** as a yellow oil (9.882 g, 90% yield). ^{1}H NMR (400 MHz, $CDCl_3$) δ 7.41 – 7.11 (m, 10H), 3.62 (s, 3 H), 2.78 (s, 1H), 2.64 (s, 1H), 1.91 – 1.68 (m, 12H). ^{13}C NMR (101 MHz, $CDCl_3$) δ 150.89, 142.16, 129.84, 125.47, 120.33, 120.28, 115.67,

115.59, 60.09, 38.76, 38.22, 37.07, 30.24, 30.08, 28.11. MS (ESI+) m/z calculated for $C_{24}H_{27}O_5P$: 426.2; found 449.4 $[M + Na]^+$.

Compound 4:

A dry flask was charged under argon with compound **3** (400 mg, 0.938 mmol, 1 eq.), $Pd(OAc)_2$ (63 mg, 0.281 mmol, 0.3 eq., 47% Pd), 1,4-Bis(diphenylphosphino)butane (42 mg, 0.093 mmol, 0.1 eq.), hexamethylditin (615 mg, 1.877 mmol, 2 eq.), dry LiCl (119 mg, 2.81 mmol, 3 eq.) and imidazole (128 mg, 1.877 mmol, 2 eq.) dissolved in Dioxane (8 mL). The reaction mixture was heated to 105 °C for 3 hours. After completion (the reaction monitored by ^{1}H-NMR), the solution was diluted with EtOAc (50 mL) and washed with water (5 mL). The organic layer was dried over Na_2SO_4 and evaporated under reduced pressure. The residue was extracted by hexane (5 mL × 3) using 10 min sonication each time; afford compound **4** as a colorless gel (230 mg, 72%). ^{1}H NMR (400 MHz, $CDCl_3$) δ 3.34 (s, 3H), 3.23 (s, 1H), 2.21 (s, 1H), 1.99 – 1.67 (m, 12H), 0.20 (s, 9H). ^{13}C NMR (101 MHz, $CDCl_3$) δ 152.21, 148.26, 59.57, 39.96, 39.26, 37.36, 36.16, 30.09, 28.83, -7.92. MS (ESI+) m/z calculated for $C_{15}H_{26}OSn$: 342.1; found 365.3 $[M + Na]^+$.

Synthesis of the Aryl-bromide compounds:

All commercially available aryl-bromides were purchased from commercial sources. Aryl-bromides **5d**,[1] **5j**,[2] **5l**,[3] **5u**,[4] **8a**,[5] **8d**[6] and **8h**[7] were prepared as previously reported. Aryl-bromides **5c** and **5e** were prepared from their respective aldehyde by Wittig reaction with Methyl (triphenylphosphoranylidene)acetate. Bromo-compound **5m** were prepared from TBDPS-protection of 8-bromoquinolin-6-ol. Bromo-coumarin **5o** was synthesized by a similar synthetic procedure reported in the literature.[8] Bromo-coumarin **5p** were prepared by phenolic TIPS-protection of bromo-coumarin **5o**. Aryl-bromide **5t** were synthesized from compound **5s** by a similar synthetic protocol reported in the literature.[9] Bromo-amino coumarin **8f** were prepared from bromo-hydroxy coumarin **5o** by Smiles rearrangement.[10]

Compound **5r**:

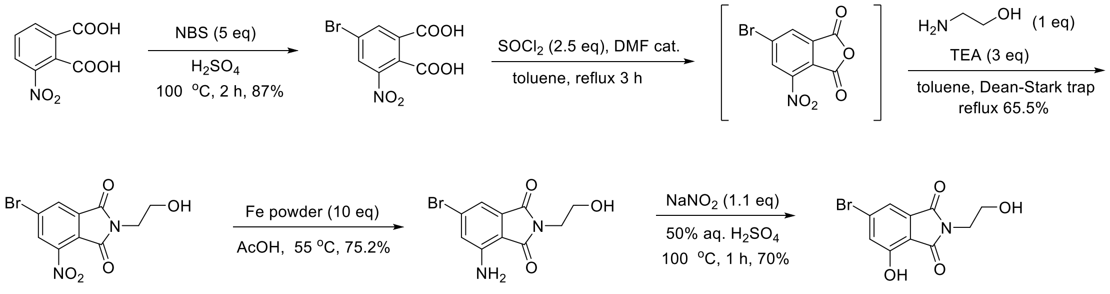

5r

To a 100 °C hot solution of 3-nitrophthalic acid (1.0 g, 4.73 mmol, 1 eq.) in conc. H_2SO_4 (50 ml), was added NBS (8.4 g, 23.7 mmol, 5 eq.) slowly over 20 mins. The reaction mixture was stirred at 100 °C for 2 hours. After HPLC monitoring showed that most of the starting material was consumed, the solution was cooled to room temperature and then pour into water（1 L）and extracted with EtOAc (5 × 100 mL). The EA layers were combined and washed with brine (100 mL) and was dried over Na_2SO_4 and evaporated under reduced pressure. The crude residue **5-bromo-3-nitrophthalic acid** as white solid was directly used for the next step without purification (1.2 g, 87% yield).

The crude **5-bromo-3-nitrophthalic acid** (500 mg, 1.72 mmol, 1 eq.) was added into anhydrous toluene (10 mL), followed by $SOCl_2$ (520 mg, 4.3 mmol, 2.5 eq.) and DMF (1 drop). The reaction mixture was heated to reflux and stirred under relux for 3 hours. Then the reaction mixture was cooled down to room temperature and was evaporated under reduced pressure and the residue compound was directly used for the next step without purification.

To the residue above was added 2-aminoethan-1-ol (105 mg, 1.72 mmol, 1 eq.), TEA (526 mg, 5.2 mmol, 3.0 eq.), and toluene (20 mL). The reaction mixture was heated to reflux and stirred under reflux for 16 hours with separating the water by Dean-Stark Trap. TLC (EA:Hex 1:1) monitoring showed the reaction was finished, then the reaction mixture was cooled down to room temperature and was diluted with 50 mL water, then extracted with ethyl acetate (50 mL × 3). The organic layer was combined, washed with brine, and dried over anhydrous Na_2SO_4. After removal of the solvent, the solid was purified by silica gel flash chromatography using ethyl acetate /n-Hexane (1 : 1) as eluent to afford compound **6-bromo-2-(2-hydroxyethyl)-4-nitroisoindoline-1,3-dione** (355 mg, 1.12 mmol, 65.5% yield).

To a solution of **6-bromo-2-(2-hydroxyethyl)-4-nitroisoindoline-1,3-dione** (315 mg, 1 mmol) in AcOH (2 mL) at 55 °C was added the Fe powder (0.56 g, 10 mmol). After stirring for 30 min at 55 °C, the mixture was cooled down to room temperature and poured into sat. $NaHCO_3$ (30 mL). The reaction mixture was filtered through celite, and the filter cake was washed with ethyl acetate (50 mL × 3). The organic layer was separated from the filtrate and was washed with brine and dried over anhydrous Na_2SO_4. After removal of the solvent, the solid was purified by silica gel flash chromatography using ethyl acetate /n-Hexane (1 : 1) as eluent to afford compound **4-amino-6-bromo-2-(2-hydroxyethyl)isoindoline-1,3-dione** (214 mg, 0.752 mmol, 75.2% yield).

To a solution of compound **4-amino-6-bromo-2-(2-hydroxyethyl)isoindoline-1,3-dione** (0.14 g, 0.5 mmol) in 50% sulfuric acid (5 mL) at 0 °C was added the solution of $NaNO_2$ (0.038 g, 1.1 mmol) in 1 mL water dropwise. After stirring for 30 min at 0 °C, the mixture was heated to 90 °C and stirred for 1 h. The reaction mixture was diluted with 15 mL water, then extracted with ethyl acetate (20 mL × 3). The organic layer was combined, washed with brine, and dried over anhydrous Na_2SO_4. After removal of the solvent, the solid was purified by silica gel flash chromatography using ethyl acetate /n-Hexane (1 : 1) as eluent to afford compound **5r** (100 mg, 0.35 mmol, 70% yield). ^{1}H NMR (400 MHz, CD_3OD) δ 7.40 (d, J = 1.3 Hz, 1H), 7.30 (d, J = 1.4 Hz, 1H), 3.73 (s, 4H). ^{13}C NMR (101 MHz, CD_3OD) δ 167.41, 155.74, 135.17, 129.03, 125.29, 117.43, 114.47, 58.82, 39.97.

Compound **8g**:

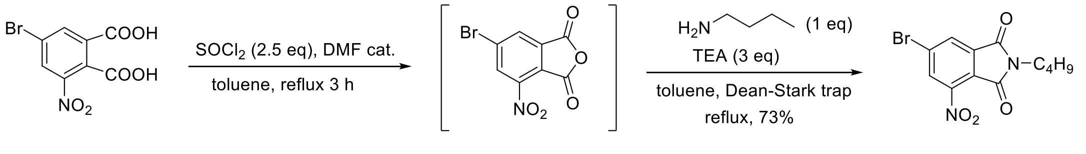

5-Bromo-3-nitrophthalic acid (500 mg, 1.72 mmol, 1 eq.) was added into anhydrous Toluene (10 mL), followed by $SOCl_2$ (520 mg, 4.3 mmol, 2.5 eq.) and DMF (1 drop). The reaction mixture was heated to reflux and stirred under reflux for 3 hours. Then the reaction mixture was cooled down to room temperature and was evaporated under reduced pressure and the residue compound was directly used for the next step without purification.

To the residue above was added butan-1-amine (126 mg, 1.72 mmol, 1 eq.), TEA (526 mg, 5.2 mmol, 3.0 eq.), and toluene (20 mL). The reaction mixture was heated to reflux and stirred under reflux for 16 hours with separating the water by Dean-Stark Trap. TLC (EA:Hex 1:1) monitoring showed the reaction was finished, then the reaction mixture was cooled down to room temperature and was diluted with 50 mL water, then extracted with ethyl acetate (50 mL × 3). The organic layer was combined, washed with brine, and dried over anhydrous Na_2SO_4. After removal of the solvent, the solid was purified by silica gel flash chromatography using ethyl acetate /n-Hexane (1 : 1) as eluent to afford compound **8g** (412 mg, 1.26 mmol, 73% yield). ^1H NMR (400 MHz, $CDCl_3$) δ 8.20 (d, J = 5.7 Hz, 2H), 3.68 (t, J = 7.3 Hz, 2H), 1.68 – 1.55 (m, 2H), 1.34 (dd, J = 15.0, 7.5 Hz, 2H), 0.92 (t, J = 7.3 Hz, 3H). ^{13}C NMR (101 MHz, $CDCl_3$) δ 164.81, 162.36, 145.19, 135.52, 131.37, 130.29, 129.36, 122.45, 77.48, 77.16, 76.84, 38.83, 30.34, 20.10, 13.63. MS (ESI+) m/z calculated for $C_{12}H_{11}O_4N_2Br$: 326.0; found 327.1 $[M + H]^+$.

Compound **8e**:

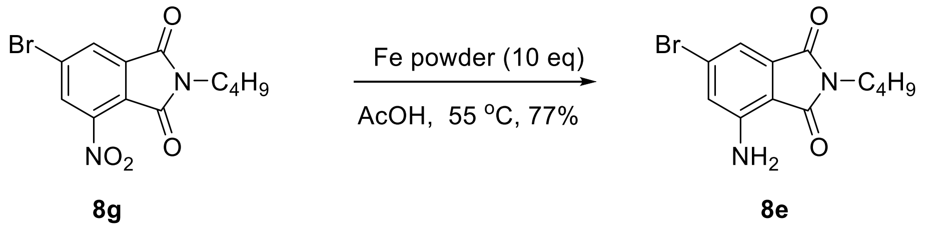

To a solution of **8g** (328 mg, 1 mmol) in AcOH (2 mL) at 55 °C was added Fe powder (0.56 g, 10 mmol). After stirring for 30 min at 55 °C, the mixture was cooled down to room temperature and poured into sat. $NaHCO_3$ (30 mL). The reaction mixture was filtered through celite, and the filter cake was washed with ethyl acetate (50 mL × 3). The organic layer was separated from the filtrate and was washed with brine and dried over anhydrous Na_2SO_4. After removal of the solvent, the solid was purified by silica gel flash chromatography using ethyl acetate /n-Hexane (1 : 2) as eluent to afford compound **8e** (229 mg, 0.77 mmol, 77% yield). ^1H NMR (400 MHz, $CDCl_3$) δ 7.23 (s, 1H), 7.01 (s, 1H), 3.60 (t, J = 7.2 Hz, 2H), 1.68 – 1.52 (m, 2H), 1.34 (dd, J = 15.0, 7.4 Hz, 2H), 0.93 (t, J = 7.3 Hz, 3H). ^{13}C NMR (101 MHz, $CDCl_3$) δ 169.86, 167.66,

145.83, 134.37, 129.53, 123.08, 116.09, 110.56, 37.70, 30.78, 20.19, 13.77. MS (ESI+) m/z calculated for $C_{12}H_{13}O_2N_2Br$: 296.0; found 295.0 $[M - H]^-$.

Compound **5q**:

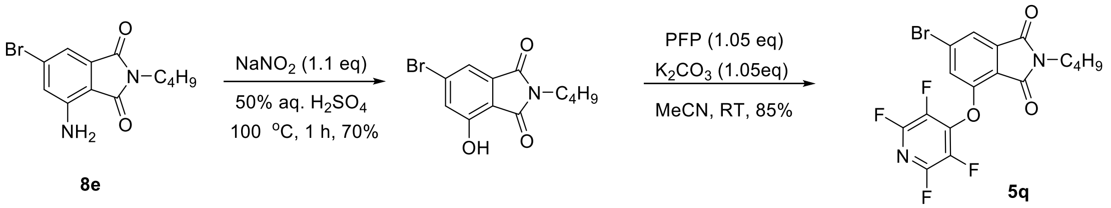

To a solution of compound **8e** (0.15 g, 0.5 mmol) in 50% sulfuric acid (5 mL) at 0 °C was added the solution of $NaNO_2$ (0.038 g, 1.1 mmol) in 1 mL water dropwise. After stirring for 30 min at 0 °C, the mixture was heated to 90 °C and stirred for 1 h. The reaction mixture was diluted with 15 mL water, then extracted with ethyl acetate (20 mL × 3). The organic layer was combined, washed with brine, and dried over anhydrous Na_2SO_4. After removal of the solvent, the solid was purified by silica gel flash chromatography using ethyl acetate /n-Hexane (1 : 2) as eluent to afford compound **6-bromo-2-butyl-4-hydroxyisoindoline-1,3-dione** (104 mg, 0.35 mmol, 70% yield).

To a stirred solution of **6-bromo-2-butyl-4-hydroxyisoindoline-1,3-dione** (104 mg, 0.35 mmol, 1 equiv.) in acetonitrile (2 mL) was added pentafluoropyridine (62 mg, 0.37 mmol, 1.05 equiv.) and potassium carbonate (52 mg, 0.37 mmol, 1.05 eq.). The reaction mixture was stirred at room temperature for 16 h. After this time the reaction mixture was concentrated under reduced pressure and the resulting residue was purified by flash column chromatography using ethyl acetate /n-Hexane (1 : 1) as eluent to afford compound **5q** (190 mg, 0.425 mmol, 85% yield). ^1H NMR (400 MHz, $CDCl_3$) δ 7.87 (s, 1H), 7.52 (s, 1H), 3.61 (t, J = 7.3 Hz, 2H), 1.66 – 1.55 (m, 2H), 1.30 (dt, J = 7.1, 6.5 Hz, 2H), 0.92 (t, J = 7.3 Hz, 3H). ^{13}C NMR (101 MHz, $CDCl_3$) δ 166.01, 164.83, 151.06, 135.64, 130.08, 127.07, 124.24, 118.84, 38.43, 30.48, 20.13, 13.67. ^{19}F NMR (376 MHz, $CDCl_3$) δ -87.78, -154.79.

Compound **5s**:

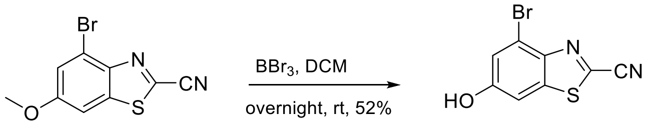

11 5s

Compound **11** was synthesized in a similar way to the synthesis of its isopropyl-analogues reported in the literature.[11] Compound **11** (100 mg, 0.374 mmol,) was dissolved in dry DCM (5 mL). To this solution, BBr_3 (0.053 mL, 561 mmol) was added dropwise at 0 °C. The reaction mixture was allowed to warm to room temperature and stirred overnight. After completion, the solution was diluted with DCM (100 mL) and washed water. The organic layer was dried over Na_2SO_4 and evaporated under reduced pressure. The residue was purified by flash column chromatography to afford compound **5s** (49 mg, 52% yield). ^{1}H NMR (400 MHz, CD_3OD) δ 7.35 (s, 2H). ^{13}C NMR (101 MHz, CD_3OD) δ 159.17, 144.33, 138.14, 133.16, 121.51, 118.17, 112.62, 105.38. MS (ESI+) m/z calculated for $C_8H_3OSN_2Br$: 253.9; found 255.0 $[M + H]^+$.

Compound **5v**:

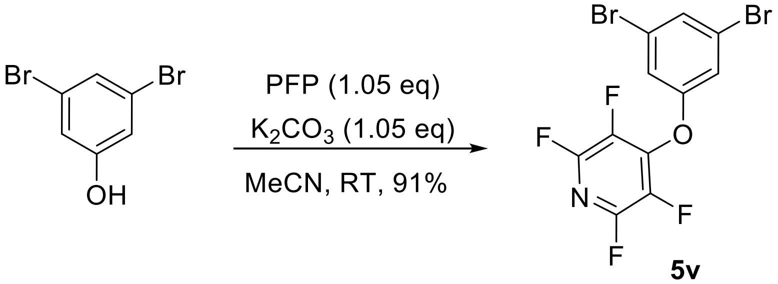

5v

To a stirred solution of **3,5-dibromophenol** (252 mg, 1 mmol, 1 eq.) in acetonitrile (20 mL) was added pentafluoropyridine (178 mg, 1.05 mmol, 1.05 equiv.) and potassium carbonate (146 mg, 0.37 mmol, 1.05 equiv.). The reaction mixture was stirred at room temperature for 16 h. After this time the reaction mixture was concentrated under reduced pressure and the resulting residue was purified by flash column chromatography using ethyl acetate /n-Hexane (1 : 2) as eluent to afford compound **5v** (365 mg, 0.91 mmol, 91% yield). ^{1}H NMR (400 MHz, $CDCl_3$) δ 7.53 (s, 1H), 7.16 (s, 2H). ^{13}C NMR (101 MHz, $CDCl_3$) δ 156.32, 131.28, 123.81, 119.05, 77.48, 77.16, 76.84. ^{19}F NMR (376 MHz, $CDCl_3$) δ -87.12, -153.50.

Compound **5w**:

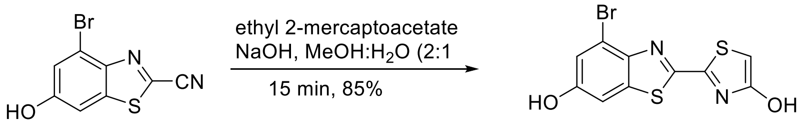

5s **5w**

Compound **5s** (100 mg, 0.393 mmol)was dissolved in methanol (2 mL) and ethyl 2-mercaptoacetate (0.048 mL, 0.433 mmol) and NaOH (17 mg, 0.433 mmol) in 1 mL water were added at 0 °C. The reaction was allowed to warm to room temperature and stirred for 15 minutes. After completion, the solution was diluted with EtOAc (100 mL) and washed with NH_4Cl, brine, and water. The organic layer was dried over Na_2SO_4 and evaporated under reduced pressure. The residue was purified by column chromatography on silica gel (Hex:EtOAc 25:75) to afford compound **5w** as a white solid (79 mg, 85% yield). ^{1}H NMR (400 MHz, DMSO-D$_6$) δ 11.05 (s, 1H), 10.39 (s, 1H), 7.45 (s, 1H), 7.25 (s, 1H), 6.55 (s, 1H). ^{13}C NMR (101 MHz, DMSO-D$_6$) δ 163.72, 158.63, 157.33, 156.17, 137.66, 120.23, 116.53, 107.43, 95.01. MS (ESI-) m/z calculated for $C_{10}H_5O_2N_2S_2Br$: 327.9; found 326.9 [M - H]$^-$.

Compound **5x**:

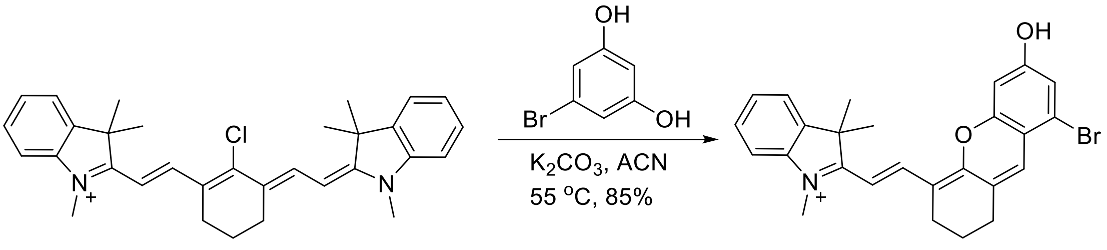

IR-775 Cl **5x**

K_2CO_3 (530 mg, 5.0 mmol) was added to a solution of 3-bromo resorcinol (550 mg, 5.0 mmol) in ACN (50 mL) at room temperature and stirred for 20 min under N_2 atmosphere. Then a solution of **IR-775 Cl** (1.295 g, 2.5 mmol) in ACN (20 mL) was added to the above solution and stirred for 12 h at 55 °C. After reaction, the solvent was evaporated under reduced pressure and the crude product was purified by silica gel column chromatography (DCM/MeOH = 25:1), obtaining product **5x** (1.052 g, 85%) as a blue-green solid. ^{1}H NMR (400 MHz, $CDCl_3$) δ: 8.56 (d, J = 14.6 Hz, 1H), 7.54 – 7.48 (m, 1H), 7.43 (dd, J = 12.9, 7.5 Hz, 1H), 7.35 (d, J = 6.5 Hz, 1H), 7.26 (dd, J =

4.3, 3.5 Hz, 1H), 7.06 – 6.96 (m, 1H), 6.91 (d, J = 2.0 Hz, 1H), 6.40 – 6.29 (m, 1H), 6.19 (d, J = 14.6 Hz, 1H), 3.72 (s, 3H), 2.69 (t, 2H), 2.58 (t, J = 5.7 Hz, 2H), 1.87 (m, 2H), 1.71 (s, 6H). ^{13}C NMR (100 MHz, $CDCl_3$) δ 177.78, 162.62, 161.90, 154.99, 146.04, 142.05, 141.61, 133.77, 129.20, 127.43, 126.82, 123.03, 122.67, 119.43, 114.53, 112.00, 110.40, 102.87, 102.41, 101.80, 50.69, 32.27, 29.13, 27.94, 22.49, 20.36. MS (ESI+) m/z calculated for $C_{26}H_{25}O_2NBr^+$: 462.1; found 462.4 $[M]^+$.

Scheme 2. General procedure for Stille coupling.

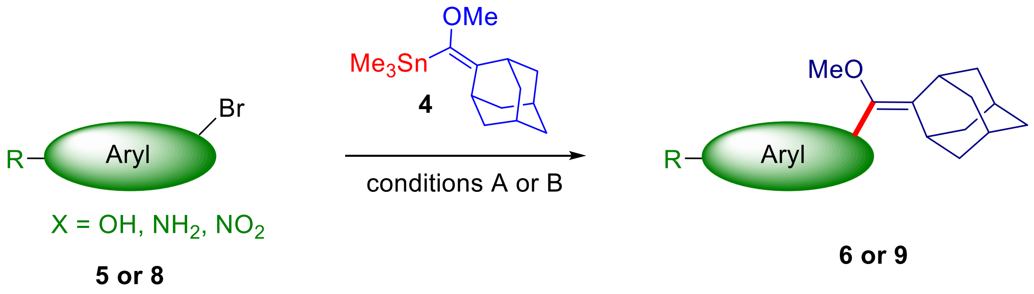

Conditions A: Compound **5** or **8** (1 mmol, 1 eq.), compound **4** (2 mmol, 2 eq.), and $Pd(PPh_3)_2Cl_2$ (0.1 mmol) were dissolved in dry Dioxane (1 mL) and the reaction mixture was stirred at 105 °C. After consumption of compound **5** or **8** (as indicated by TLC or RP-HPLC), the solvent was evaporated under reduced pressure and the crude residue was purified using silica column chromatography or RP-HPLC.

Conditions B: Compound **5** or **8** (1 mmol, 1 eq.), compound **4** (2 mmol, 2 eq.), CuCl (5 mmol, 5 eq), LiCl (5 mmol, 5 eq), and $Pd(PPh_3)_4$ (0.1 mmol) were dissolved in dry DMSO (1 mL) and the reaction mixture was stirred at 60-80 °C. After consumption of compound **5** or **8** (as indicated by TLC or RP-HPLC), the reaction was diluted with EtOAc (20 mL), washed with H_2O (2 × 10 mL), dried over Na_2SO_4, and concentrated under reduced pressure. The crude residue was purified using silica column chromatography or RP-HPLC.

Compound **6a**:

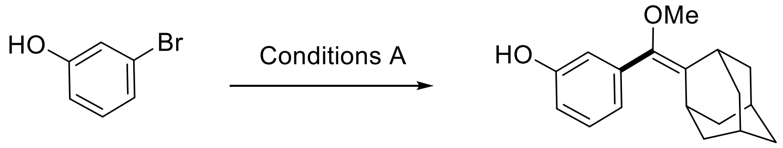

Conditions A were used for the synthesis of compound **6a**. Yield = 61%.

Compound **6b**:

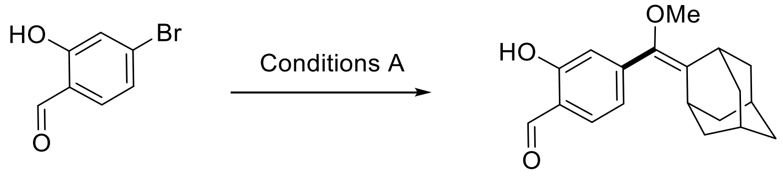

Conditions A were used for the synthesis of compound **6b**. Yield = 53%. ^{1}H NMR (400 MHz, $CDCl_3$) δ 11.05 (s, 1H), 9.86 (s, 1H), 7.52 (d, *J* = 8.0 Hz, 1H), 7.00 (dd, *J* = 8.0, 1.1 Hz, 1H), 6.94 (s, 1H), 3.32 (s, 3H), 3.25 (s, 1H), 2.72 (s, 1H), 2.00 – 1.75 (m, 12H). ^{13}C NMR (101 MHz, $CDCl_3$) δ 195.96, 161.56, 145.03, 142.54, 135.79, 133.45, 121.05, 119.81, 118.11, 39.33, 39.19, 37.14, 32.52, 30.64, 28.28. MS (ESI+) m/z calculated for $C_{19}H_{22}O_3$: 298.2; found 297.4 $[M - H]^-$.

Compound **6c**:

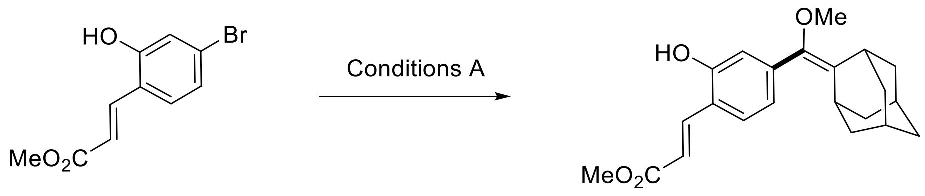

Conditions A were used for the synthesis of compound **6c.** Yield = 58%. ^{1}H NMR (400 MHz, $CDCl_3$) δ 8.01 (d, *J* = 16.1 Hz, 1H), 7.42 (d, *J* = 7.9 Hz, 1H), 6.93 (s, 1H), 6.86 (d, *J* = 7.8 Hz, 1H), 6.64 (d, *J* = 16.1 Hz, 1H), 3.82 (s, 3H), 3.34 (s, 3H), 3.23 (s, 1H), 2.70 (s, 1H), 2.00 – 1.68 (m, 12H). ^{13}C NMR (101 MHz, $CDCl_3$) δ 168.86, 155.95, 142.51, 140.69, 138.85, 134.19, 128.96, 122.06, 121.09, 117.95, 116.75, 58.16, 51.90, 39.30, 39.17, 37.18, 32.50, 30.59, 28.33. MS (ESI+) m/z calculated for $C_{22}H_{26}O_4$: 354.2; found 353.4 $[M - H]^-$.

Compound **6d**:

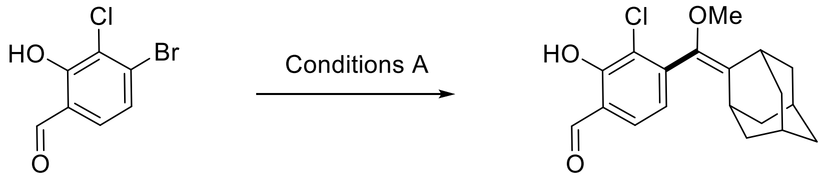

Conditions A were used for the synthesis of compound **6d.** Yield = 42%. ^{1}H NMR (400 MHz, $CDCl_3$) δ 11.62 (s, 1H), 9.90 (s, 1H), 7.49 (d, *J* = 7.9 Hz, 1H), 6.98 (d, *J* = 7.9 Hz, 1H), 3.34 (s, 3H), 3.28 (s, 1H), 2.07 (s, 1H), 2.00 – 1.65 (m, 12H). ^{13}C NMR (101 MHz, $CDCl_3$) δ 195.83, 157.72, 143.68, 139.05, 133.53, 131.03, 123.29, 123.03, 120.55, 57.66, 39.37, 39.18, 38.72, 37.14, 33.12, 29.90, 28.45, 28.29. MS (ESI+) m/z calculated for $C_{19}H_{21}O_3Cl$: 332.1; found 331.2 $[M - H]^-$.

Compound **6e**:

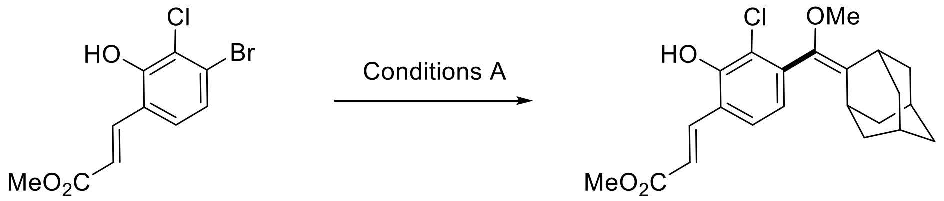

Conditions A were used for the synthesis of compound **6e**. Yield = 31%. ^{1}H NMR (400 MHz, $CDCl_3$) δ 7.94 (d, *J* = 16.2 Hz, 1H), 7.38 (d, *J* = 7.9 Hz, 1H), 6.86 (d, *J* = 7.8 Hz, 1H), 6.61 (d, *J* = 16.2 Hz, 1H), 6.24 (s, 1H), 3.82 (s, 3H), 3.31 (s, 3H), 3.27 (s, 1H), 2.12 (s, 1H), 2.01 – 1.66 (m, 12H). This compound was previously reported.[12]

Compound **6f**:

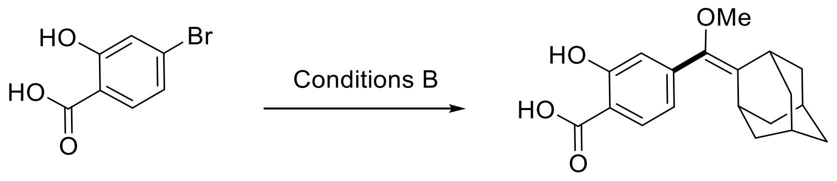

Conditions B were used for the synthesis of compound **6f.** Yield = 76%. ^{1}H NMR (400 MHz, $CDCl_3$) δ 10.47 (s, 1H), 7.89 (d, *J* = 8.2 Hz, 1H), 6.97 (d, *J* = 1.3 Hz, 1H), 6.92 (dd, *J* = 8.2, 1.4 Hz, 1H), 3.34 (s, 3H), 3.26 (s, 1H), 2.72 (s, 1H), 2.01 – 1.73 (m, 12H).

^{13}C NMR (101 MHz, $CDCl_3$) δ 174.00, 162.07, 144.71, 142.51, 135.16, 130.66, 120.81, 118.29, 110.36, 58.32, 39.35, 39.22, 37.20, 32.51, 30.61, 28.34. MS (ESI+) m/z calculated for $C_{19}H_{22}O_4$: 314.2; found 313.4 $[M - H]^-$.

Compound **6g**:

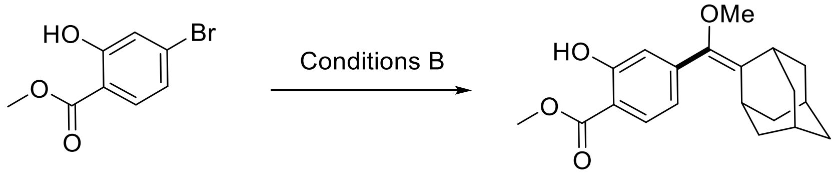

Conditions B were used for the synthesis of compound **6g.** Yield = 72%. ^1H NMR (400 MHz, $CDCl_3$) δ 10.75 (s, 1H), 7.80 (d, *J* = 8.2 Hz, 1H), 6.93 (s, 1H), 6.87 (d, *J* = 8.2 Hz, 1H), 3.95 (s, 3H), 3.31 (s, 3H), 3.24 (s, 1H), 2.69 (s, 1H), 2.00 – 1.76 (m, 15H). ^{13}C NMR (101 MHz, $CDCl_3$) δ 170.53, 161.42, 143.55, 142.65, 134.46, 129.66, 120.45, 118.18, 111.36, 58.22, 52.40, 39.33, 39.20, 37.22, 32.45, 30.52, 28.35. MS (ESI+) m/z calculated for $C_{20}H_{24}O_4$: 328.2; found 329.4 $[M + H]^+$.

Compound **6h**:

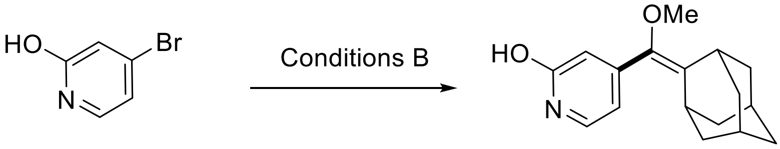

Conditions B were used for the synthesis of compound **6h.** Yield = 25%. ^1H NMR (400 MHz, $CDCl_3$) δ 7.45 (d, *J* = 6.7 Hz, 1H), 6.59 (s, 1H), 6.48 (d, *J* = 6.6 Hz, 1H), 3.35 (s, 3H), 3.23 (s, 1H), 2.79 (s, 1H), 2.00 – 1.69 (m, 12H). ^{13}C NMR (101 MHz, $CDCl_3$) δ 164.97, 150.63, 140.94, 138.54, 134.29, 118.62, 110.06, 58.61, 39.33, 39.16, 37.00, 32.55, 30.75, 28.15. MS (ESI+) m/z calculated for $C_{17}H_{21}O_2N$: 271.2; found 272.3 $[M + H]^+$.

Compound **6i**:

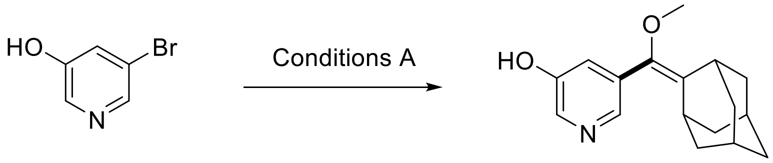

Conditions A were used for the synthesis of compound **6i.** Yield = 18%. ^{1}H NMR (400 MHz, $CDCl_3$ + CD_3OD) δ 7.90 (d, J = 2.7 Hz, 1H), 7.84 (d, J = 1.6 Hz, 1H), 7.02 (dd, J = 2.6, 1.8 Hz, 1H), 3.19 (s, 3H), 3.10 (s, 1H), 2.45 (s, 1H), 1.88 – 1.58 (m, 12H). ^{13}C NMR (101 MHz, $CDCl_3$ + CD_3OD) δ 153.74, 140.59, 139.96, 136.15, 134.75, 132.36, 123.58, 57.89, 39.04, 38.85, 36.87, 32.25, 30.21, 28.06. MS (ESI+) m/z calculated for $C_{17}H_{21}O_2N$: 271.2; found 272.2 $[M + H]^+$.

Compound **6j**:

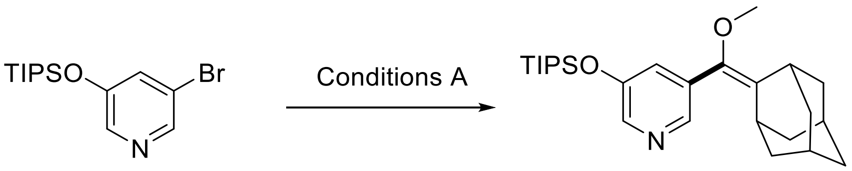

Conditions A were used for the synthesis of compound **6j.** Yield = 42%. ^{1}H NMR (400 MHz, $CDCl_3$) δ 8.17 (d, J = 2.6 Hz, 1H), 8.14 (d, J = 1.5 Hz, 1H), 7.14 (dd, J = 2.7, 1.8 Hz, 1H), 3.30 (s, 3H), 3.25 (s, 1H), 2.57 (s, 1H), 2.01 – 1.73 (m, 12H), 1.29 – 1.23 (m, 3H), 1.10 (d, J = 7.2 Hz, 18H). ^{13}C NMR (101 MHz, $CDCl_3$) δ 152.54, 143.14, 141.38, 140.40, 134.32, 132.08, 127.23, 58.08, 39.32, 39.14, 37.21, 32.50, 30.39, 28.35, 17.95, 12.75. MS (ESI+) m/z calculated for $C_{26}H_{41}O_2NSi$: 427.3; found 428.4 $[M + H]^+$.

Compound **6k**:

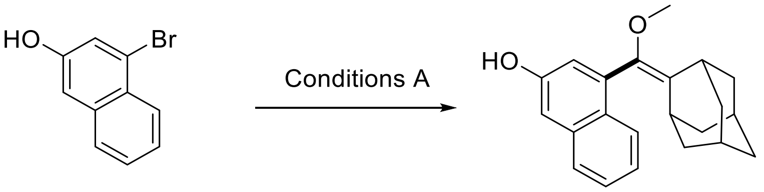

Conditions A were used for the synthesis of compound **6k.** Yield = 68%. ^{1}H NMR (400 MHz, $CDCl_3$) δ 8.04 (d, J = 8.3 Hz, 1H), 7.68 (d, J = 8.2 Hz, 1H), 7.43 (t, J = 7.3 Hz,

1H), 7.33 (t, J = 7.3 Hz, 1H), 7.15 (d, J = 2.2 Hz, 1H), 7.05 (d, J = 2.4 Hz, 1H), 3.40 (s, 1H), 3.26 (s, 3H), 2.14 (s, 1H), 2.01 – 1.56 (m, 12H). ^{13}C NMR (101 MHz, $CDCl_3$) δ 152.87, 140.71, 135.20, 135.04, 132.15, 128.35, 126.75, 126.68, 126.22, 123.89, 120.05, 109.94, 57.15, 39.37, 39.24, 39.08, 37.24, 32.84, 29.97, 28.59, 28.43. MS (ESI+) m/z calculated for $C_{22}H_{24}O_2$: 320.2; found 319.2 $[M - H]^-$.

Compound **6l**:

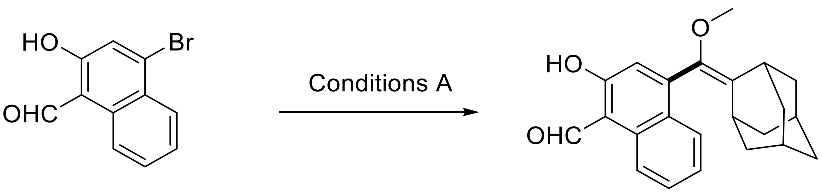

Conditions A were used for the synthesis of compound **6l**. Yield = 44%. ^{1}H NMR (400 MHz, $CDCl_3$) δ 13.18 (s, 1H), 10.91 – 10.77 (m, 1H), 8.37 (d, J = 8.5 Hz, 1H), 8.13 (d, J = 8.4 Hz, 1H), 7.68 – 7.54 (m, 1H), 7.44 (dd, J = 11.2, 4.0 Hz, 1H), 7.06 (s, 1H), 3.40 (s, 1H), 3.28 (s, 3H), 2.19 (s, 1H), 2.04 – 1.55 (m, 12H). ^{13}C NMR (101 MHz, $CDCl_3$) δ 193.30, 164.08, 144.94, 140.21, 134.11, 133.34, 129.23, 127.89, 127.43, 124.76, 121.18, 118.89, 111.46, 57.65, 39.35, 39.29, 39.03, 37.12, 32.95, 29.98, 28.50, 28.35. MS (ESI+) m/z calculated for $C_{23}H_{24}O_3$: 348.2; found 347.3 $[M - H]^-$.

Compound **6m**:

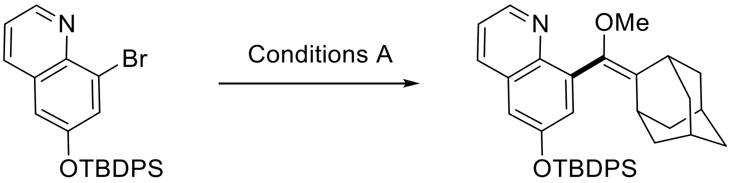

Conditions A were used for the synthesis of compound **6m**. Yield = 22%. ^{1}H NMR (400 MHz, $CDCl_3$) δ 8.80 (dd, J = 4.2, 1.7 Hz, 1H), 7.83 (dd, J = 8.3, 1.7 Hz, 1H), 7.75 (d, J = 6.9 Hz, 4H), 7.40 (dd, J = 18.4, 6.5 Hz, 6H), 7.25 (dd, J = 8.3, 4.2 Hz, 1H), 7.13 (d, J = 2.8 Hz, 1H), 7.07 (d, J = 2.8 Hz, 1H), 3.34 (s, 1H), 3.10 (s, 3H), 2.05 (s, 1H), 1.97 – 1.54 (m, 12H), 1.16 (s, 9H). ^{13}C NMR (101 MHz, $CDCl_3$) δ 152.86, 148.46, 143.57, 140.88, 136.11, 135.66, 134.94, 132.77, 131.10, 130.20, 129.54, 128.00,

127.20, 121.21, 114.75, 57.14, 39.34, 37.43, 32.97, 29.83, 29.77, 28.64, 26.69, 19.67. MS (ESI+) m/z calculated for $C_{37}H_{41}O_2NSi$: 559.3; found 560.4 $[M + H]^+$.

Compound **6n**:

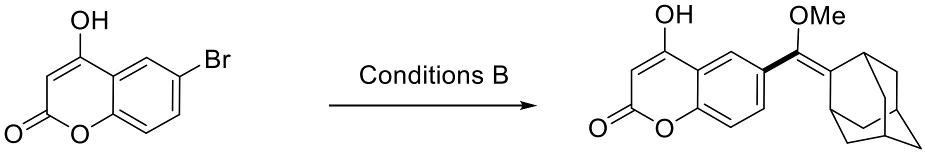

Conditions B were used for the synthesis of compound **6n**. Yield = 71%. ^{1}H NMR (400 MHz, $CDCl_3$) δ 7.89 (d, J = 8.5 Hz, 1H), 7.30 (m, 2H), 5.92 (s, 1H), 3.33 (s, 3H), 3.27 (s, 1H), 2.71 (s, 1H), 1.99 – 1.72 (m, 12H). ^{13}C NMR (101 MHz, $CDCl_3$) δ 167.27, 165.84, 153.84, 142.26, 140.92, 135.34, 125.43, 123.48, 117.29, 115.08, 91.84, 58.41, 39.34, 39.22, 38.25, 37.18, 33.73, 32.53, 30.68, 29.70, 28.32. MS (ESI+) m/z calculated for $C_{21}H_{22}O_4$: 338.2; found 339.2 $[M + H]^+$.

Compound **6o**:

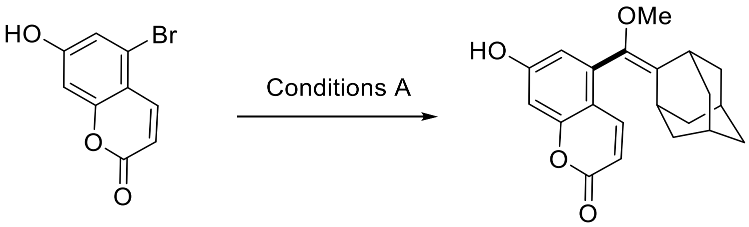

Conditions A were used for the synthesis of compound **6o**. Yield = 30%. ^{1}H NMR (400 MHz, $CD_3OD + CDCl_3$) δ 7.84 (d, J = 9.6 Hz, 1H), 6.68 (d, J = 1.7 Hz, 1H), 6.62 (d, J = 2.4 Hz, 1H), 6.12 (d, J = 9.6 Hz, 1H), 3.22 (s, 1H), 3.19 (s, 3H), 2.15 (s, 1H), 1.92 – 1.59 (m, 12H). ^{13}C NMR (101 MHz, $CD_3OD + CDCl_3$) δ 162.41, 160.43, 156.28, 142.92, 139.02, 135.71, 134.23, 115.65, 111.59, 111.29, 102.56, 57.27, 39.13, 39.02, 36.93, 32.64, 29.89, 28.20, 28.16, 17.61. MS (ESI+) m/z calculated for $C_{21}H_{22}O_4$: 338.2; found 339.2 $[M + H]^+$.

Compound **6p**:

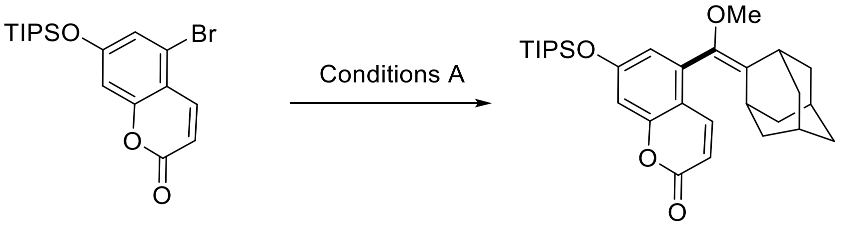

Conditions A were used for the synthesis of compound **6p**. Yield = 75%. ^{1}H NMR (400 MHz, $CDCl_3$) δ 7.87 (d, J = 9.5 Hz, 1H), 6.79 (d, J = 1.9 Hz, 1H), 6.69 (d, J = 2.4 Hz, 1H), 6.23 (d, J = 9.7 Hz, 1H), 3.31 (s, 1H), 3.25 (s, 3H), 2.19 (s, 1H), 2.02 – 1.71 (m, 12H), 1.32 – 1.24 (m, 3H), 1.12 (s, 6H), 1.10 (s, 6H), 1.05 (s, 6H). ^{13}C NMR (101 MHz, $CDCl_3$) δ 161.46, 158.88, 156.14, 142.14, 139.15, 135.42, 134.12, 119.54, 113.15, 112.61, 107.50, 39.20, 39.08, 36.99, 32.75, 29.90, 28.22, 17.77, 12.38. MS (ESI+) m/z calculated for $C_{30}H_{42}O_4Si$: 494.3; found 495.4 $[M + H]^+$.

Compound **6q**:

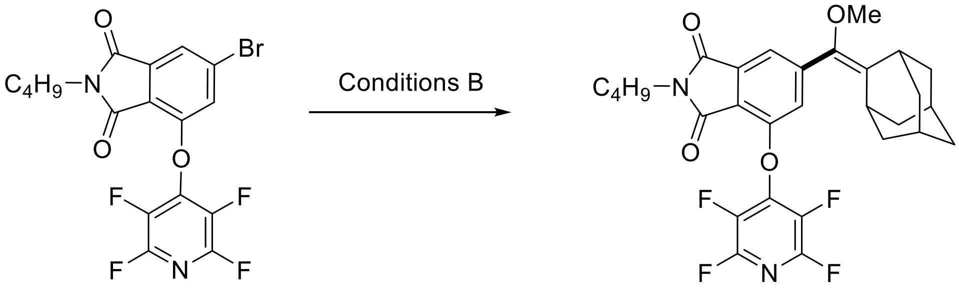

Conditions B were used for the synthesis of compound **6q**. Yield = 50%. ^{1}H NMR (400 MHz, $CDCl_3$) δ 7.66 (s, 1H), 7.28 (s, 1H), 3.63 (t, J = 7.3 Hz, 2H), 3.33 (s, 3H), 3.26 (s, 1H), 2.65 (s, 1H), 2.04 – 1.56 (m, 14H), 1.39 – 1.28 (m, 2H), 0.93 (t, J = 7.3 Hz, 3H). ^{13}C NMR (101 MHz, $CDCl_3$) δ 167.39, 165.36, 150.87, 145.16, 141.16, 137.95, 134.52, 123.69, 121.26, 118.35, 58.67, 39.28, 39.13, 38.21, 36.96, 32.68, 30.82, 30.63, 28.12, 20.18, 13.73. ^{19}F NMR (376 MHz, $CDCl_3$) δ -88.32, -154.82.

Compound **6r**:

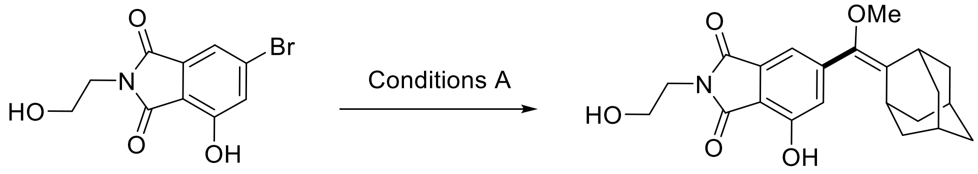

Conditions A were used for the synthesis of compound **6r**. Yield = 39%. ^{1}H NMR (400 MHz, CD_3OD) δ 7.23 (d, J = 1.1 Hz, 1H), 7.09 (d, J = 1.1 Hz, 1H), 3.75 (t, J = 2.8 Hz, 4H), 3.32 (s, 3H), 3.25 (s, 1H), 2.65 (s, 1H), 2.02 – 1.79 (m, 12H). ^{13}C NMR (101 MHz, CD_3OD) δ 168.62, 167.88, 155.00, 143.86, 142.29, 134.93, 133.82, 122.93, 115.17, 114.07, 58.90, 57.28, 39.83, 38.88, 38.76, 36.77, 32.64, 30.53, 28.35. MS (ESI+) m/z calculated for $C_{22}H_{25}O_5N$: 383.2; found 382.4 $[M - H]^-$.

Compound **6s**:

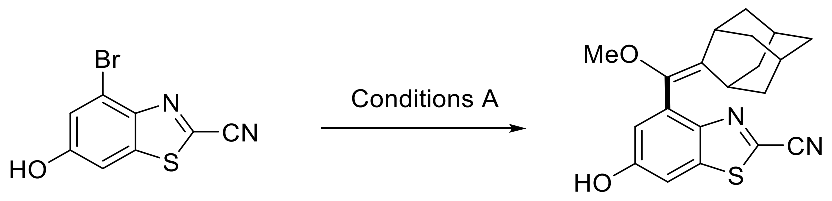

Conditions A were used for the synthesis of compound **6s**. Yield = 46%. ^{1}H NMR (400 MHz, $CDCl_3$) δ 7.34 (d, J = 2.4 Hz, 1H), 7.16 (d, J = 2.4 Hz, 1H), 3.40 (s, 3H), 3.34 (s, 1H), 2.11 (s, 1H), 2.01 – 1.65 (m, 12H). ^{13}C NMR (101 MHz, $CDCl_3$) δ 157.02, 146.29, 138.60, 137.93, 135.94, 134.04, 132.86, 119.31, 113.42, 106.22, 58.42, 39.06, 38.96, 37.16, 33.19, 30.46, 28.32. MS (ESI+) m/z calculated for $C_{20}H_{20}O_2N_2S$: 352.1; found 353.1 $[M + H]^+$.

Compound **6t**:

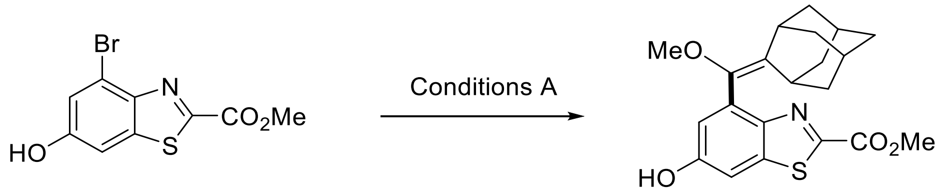

Conditions A were used for the synthesis of compound **6t**. Yield = 43%. ^{1}H NMR (400 MHz, $CDCl_3$) δ 7.34 (s, 1H), 7.09 (s, 1H), 3.99 (s, 3H), 3.36 (s, 3H), 3.33 (s, 1H), 2.20 (s, 1H), 1.99 – 1.63 (m, 12H). ^{13}C NMR (101 MHz, Acetone-D_6) δ 160.79, 156.74, 152.96, 145.99, 139.41, 138.53, 133.21, 132.64, 118.40, 105.59, 57.06, 52.53, 38.43, 38.35, 36.74, 32.48, 29.64, 27.91. MS (ESI+) m/z calculated for $C_{21}H_{23}O_4NS$: 385.1; found 386.3 $[M + H]^+$.

Compound **6u**:

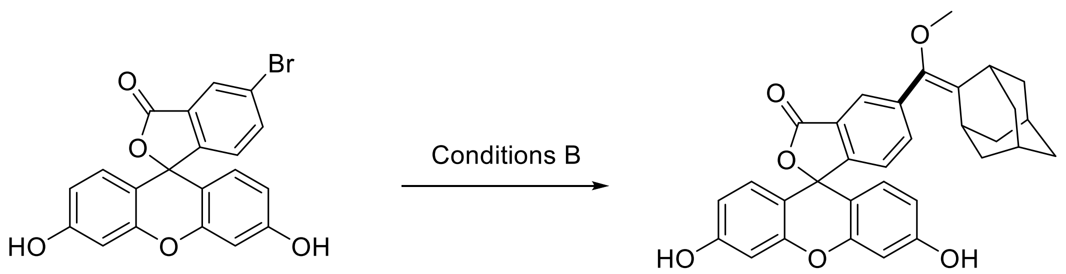

Conditions B were used for the synthesis of compound **6u**. Yield = 55%. ^{1}H NMR (400 MHz, Acetone-D_6 + $CDCl_3$) δ 7.81 (s, 1H), 7.52 (dd, *J* = 7.9, 1.4 Hz, 1H), 7.05 (d, *J* = 7.9 Hz, 1H), 6.69 (d, *J* = 2.3 Hz, 2H), 6.60 (d, *J* = 8.7 Hz, 2H), 6.52 (dd, *J* = 8.7, 2.3 Hz, 2H), 3.24 (s, 3H), 3.19 (s, 1H), 2.57 (s, 1H), 1.91 – 1.68 (m, 12H). ^{13}C NMR (101 MHz, Acetone-D_6 + $CDCl_3$) δ 169.30, 159.70, 152.92, 150.85, 142.03, 137.50, 135.66, 134.27, 129.23, 127.08, 125.56, 124.04, 112.81, 110.88, 102.89, 58.15, 40.57, 39.16, 38.97, 36.95, 32.25, 28.12. MS (ESI+) m/z calculated for $C_{32}H_{28}O_6$: 508.2; found 509.4 $[M + H]^+$.

Compound **6v**:

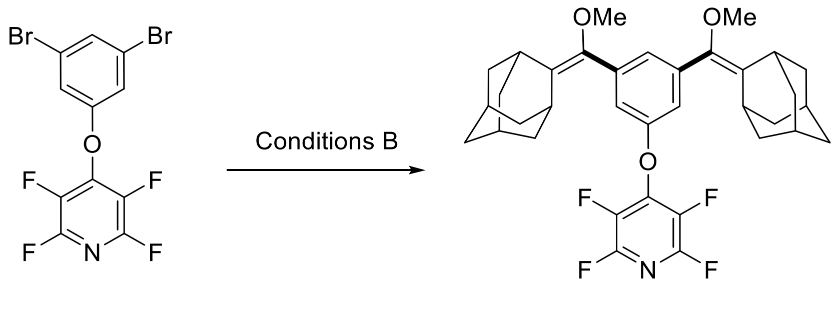

Conditions B were used for the synthesis of compound **6v** and in addition 4 eq of compound **4** were added. Yield = 78%. ^{1}H NMR (400 MHz, $CDCl_3$) δ 7.08 (s, 1H), 6.95 (s, 2H), 3.31 (s, 6H), 3.24 (s, 2H), 2.63 (s, 2H), 2.00 – 1.71 (m, 24H). ^{13}C NMR (101 MHz, $CDCl_3$) δ 155.91, 142.41, 137.39, 133.57, 127.43, 116.03, 58.07, 39.37, 39.14, 37.21, 32.55, 30.50, 28.35. ^{19}F NMR (376 MHz, $CDCl_3$) δ -88.70, -154.16. MS (ESI+) m/z calculated for $C_{35}H_{37}O_3NF_4$: 595.3; found 596.4 $[M + H]^+$.

Compound **6w**:

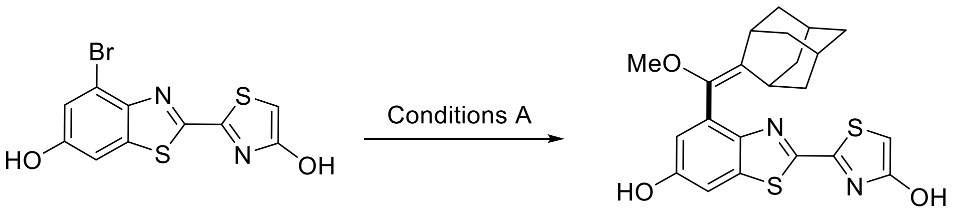

Conditions A were used for the synthesis of compound **6w** and reaction was heated to 140 °C for overnight. Yield = 21%. This enol ether was not very stable, so used for next step without collecting 13C-NMR data. ^{1}H NMR (400 MHz, DMSO-D_6) δ 11.06 (s, 1H), 10.32 (s, 1H), 7.63 (d, *J* = 2.2 Hz, 1H), 7.25 (d, *J* = 2.2 Hz, 1H), 6.53 (s, 1H), 3.25 (s, 3H), 3.23 (s, 1H), 2.19 (s, 1H), 1.94 – 1.60 (m, 12H). MS (ESI+) m/z calculated for $C_{22}H_{22}O_3N_2S_2$: 426.1; found 449.2 $[M + H]^+$.

Compound **6x**:

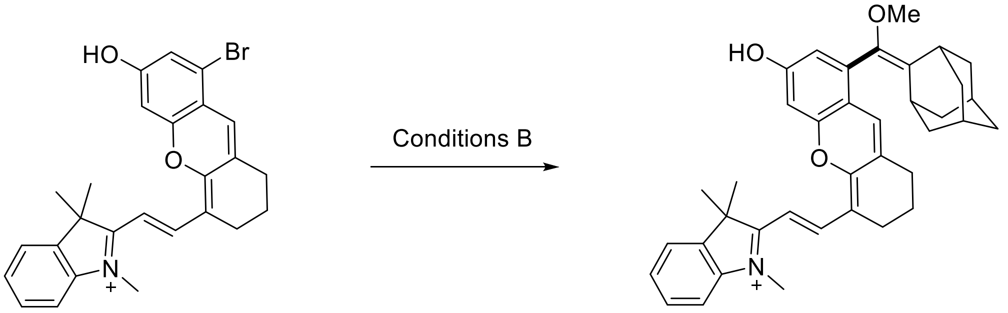

Conditions B were used for the synthesis of compound **6x**. Yield = 23%. ^{1}H NMR (400 MHz, $CDCl_3$) δ: 8.69 (d, *J* = 14.5 Hz, 1H), 7.65 (s, 1H), 7.47 (d, *J* = 7.2 Hz, 1H), 7.45 – 7.39 (m, 1H), 7.35 – 7.28 (m, 2H), 7.17 (d, *J* = 7.8 Hz, 1H), 6.88 (d, *J* = 2.2 Hz, 1H), 6.01 (d, *J* = 14.4 Hz, 1H), 3.67 (s, 3H), 3.30 (s, 1H), 3.27 (s, 3H), 2.77 (t, *J* = 5.5 Hz,

2H), 2.63 (t, J = 6.1 Hz, 2H), 2.29 (s, 1H), 2.04 – 1.79 (m, 16H), 1.76 (s, 6H). ^{13}C NMR (100 MHz, CDCl3) δ: 175.90, 164.17, 164.12, 161.29, 160.92, 156.13, 144.60, 142.38, 141.27, 139.06, 137.36, 135.44, 132.22, 128.93, 128.72, 126.40, 124.83, 122.87, 119.64, 114.58, 114.32, 110.79, 102.74, 99.90, 57.77, 50.15, 39.32, 37.07, 32.73, 31.79, 30.15, 29.83, 29.07, 28.32, 24.19, 20.67. MS (ESI+) m/z calculated for $C_{38}H_{42}O_3N$+: 560.3; found 560.6 $[M]^+$.

Compound **9a**:

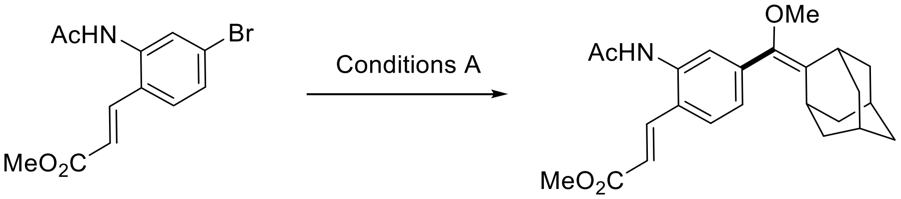

Conditions A were used for the synthesis of compound **9a**. Yield = 46%. ^{1}H NMR (400 MHz, $CDCl_3$) δ 7.80 (d, J = 15.7 Hz, 1H), 7.72 (s, 1H), 7.55 (d, J = 7.8 Hz, 1H), 7.26 – 7.16 (m, 2H), 6.42 (d, J = 15.8 Hz, 1H), 3.81 (s, 3H), 3.32 (s, 3H), 3.24 (s, 1H), 2.74 (s, 1H), 2.25 (s, 3H), 1.84 (m, 12H). ^{13}C NMR (101 MHz, $CDCl_3$) δ 168.82, 167.44, 142.72, 139.29, 138.60, 135.68, 134.21, 126.99, 126.77, 126.50, 126.05, 119.94, 58.28, 52.02, 39.33, 39.22, 37.27, 32.48, 30.60, 28.37, 24.43. MS (ESI+) m/z calculated for $C_{24}H_{29}O_4N$: 395.2; found 396.4 $[M + H]^+$.

Compound **9b**:

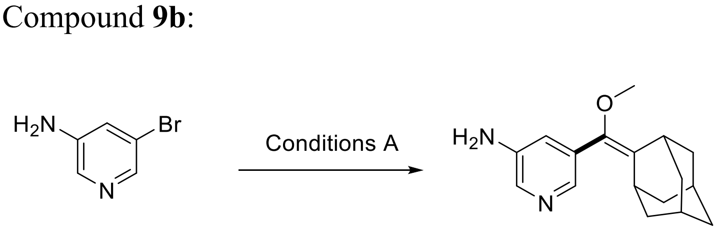

Conditions A were used for the synthesis of compound **9b**. Yield = 31%. ^{1}H NMR (400 MHz, CD_3OD) δ 7.91 (dd, J = 2.6, 0.5 Hz, 1H), 7.83 (s, 1H), 7.58 (dd, J = 2.5, 1.5 Hz, 1H), 3.38 (s, 3H), 3.26 (s, 1H), 2.60 (s, 1H), 1.75 – 2.10 (m, 12H). ^{13}C NMR (101 MHz, CD_3OD) δ 148.29, 138.25, 137.89, 135.71, 128.02, 127.94, 124.18, 57.56, 38.68, 38.50, 36.45, 32.44, 30.48, 28.07. MS (ESI+) m/z calculated for $C_{17}H_{22}ON_2$: 270.2; found 271.2 $[M + H]^+$.

Compound **9c**:

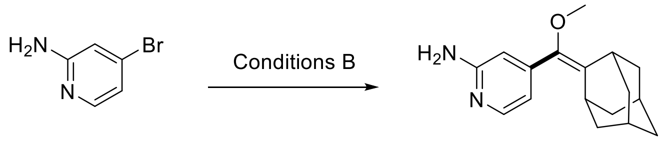

Conditions B were used for the synthesis of compound **9c**. Yield = 13%. ^{1}H NMR (400 MHz, CD_3OD) δ 7.80 (d, *J* = 6.7 Hz, 1H), 6.91 (s, 1H), 6.83 (d, *J* = 6.7 Hz, 1H), 3.39 (s, 3H), 3.27 (s, 1H), 2.81 (s, 1H), 2.08 – 1.77 (m, 12H). ^{13}C NMR (101 MHz, CD_3OD) δ 154.43, 151.51, 140.79, 140.33, 134.82, 112.85, 112.15, 57.82, 48.22, 48.01, 47.80, 47.58, 47.37, 47.16, 46.94, 38.79, 38.63, 36.40, 32.61, 30.87, 28.02. MS (ESI+) m/z calculated for $C_{17}H_{22}ON_2$: 270.2; found 271.3 $[M + H]^+$.

Compound **9d**:

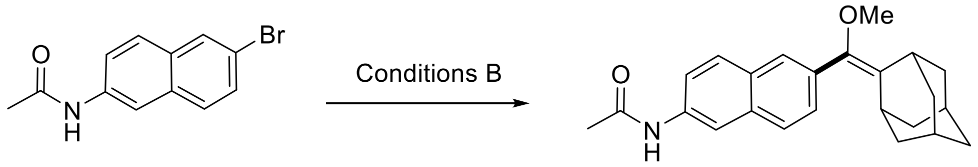

Conditions B were used for the synthesis of compound **9d**. Yield = 56%. ^{1}H NMR (400 MHz, C_6D_6) δ 8.23 (d, *J* = 6.5 Hz, 1H), 7.81 (s, 1H), 7.62 – 7.46 (m, 3H), 7.36 – 7.27 (m, 1H), 3.58 (s, 1H), 3.23 (s, 3H), 2.86 (s, 1H), 2.02 – 1.49 (m, 15H). ^{13}C NMR (101 MHz, C_6D_6) δ 167.45, 144.33, 136.41, 133.74, 132.23, 131.37, 130.49, 128.76, 120.06, 116.07, 57.33, 39.37, 39.23, 37.31, 32.60, 30.50, 28.63, 23.97. MS (ESI+) m/z calculated for $C_{24}H_{27}O_2N$: 361.2; found 362.4 $[M + H]^+$.

Compound **9e**:

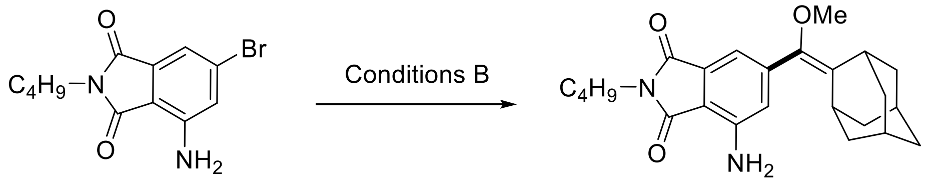

Conditions B were used for the synthesis of compound **9e**. Yield = 34%. ^{1}H NMR (400 MHz, $CDCl_3$) δ 7.09 (s, 1H), 6.80 (s, 1H), 5.18 (s, 2H), 3.62 (t, *J* = 7.2 Hz, 2H), 3.31 (s, 3H), 3.23 (s, 1H), 2.62 (s, 1H), 1.98 – 1.71 (m, 12H), 1.69 – 1.60 (m, 2H), 1.36 (m, 2H), 0.94 (t, *J* = 7.3 Hz, 3H). ^{13}C NMR (101 MHz, $CDCl_3$) δ 170.21, 168.91, 145.03, 143.39, 142.41, 134.81, 132.96, 121.26, 114.36, 110.71, 77.48, 77.16, 76.84, 58.26, 39.35, 39.17, 37.54, 37.14, 32.56, 30.91, 30.52, 29.83, 28.30, 20.23, 13.81. MS (ESI+) m/z calculated for $C_{24}H_{30}O_3N_2$: 394.2; found 417.4 $[M + Na]^+$.

Compound **9f**:

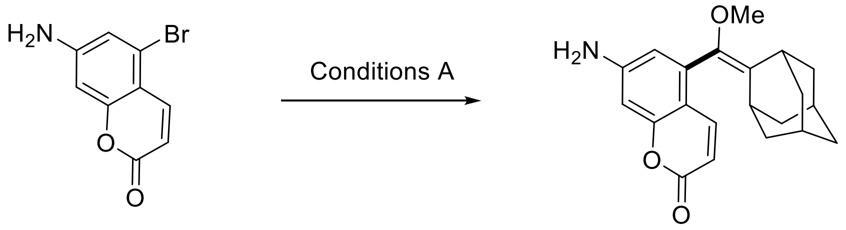

Conditions A were used for the synthesis of compound **9f**. Yield = 46%. ^{1}H NMR (400 MHz, $CDCl_3$) δ 7.81 (d, *J* = 9.6 Hz, 1H), 6.55 (d, *J* = 2.0 Hz, 1H), 6.45 (d, *J* = 2.1 Hz, 1H), 6.11 (d, *J* = 9.6 Hz, 1H), 4.22 (s, 2H), 3.29 (s, 1H), 3.26 (s, 3H), 2.21 (s, 1H), 2.00 – 1.73 (m, 12H). ^{13}C NMR (101 MHz, $CDCl_3$) δ 161.94, 156.80, 149.73, 142.50, 139.30, 135.76, 133.73, 114.02, 110.99, 110.31, 100.85, 57.36, 39.26, 37.09, 32.72, 29.94, 29.83, 28.39. MS (ESI+) m/z calculated for $C_{21}H_{23}O_3N$: 337.7; found 360.6 $[M - H]^+$.

Compound **9g**:

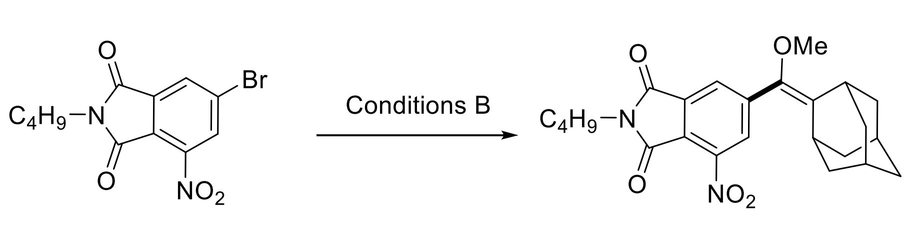

Conditions B were used for the synthesis of compound **9g**. Yield = 91%. ^{1}H NMR (400 MHz, $CDCl_3$) δ 8.05 – 7.94 (m, 2H), 3.72 (t, *J* = 7.3 Hz, 2H), 3.36 (s, 3H), 3.29 (s, 1H), 2.67 (s, 1H), 2.08 – 1.78 (m, 12H), 1.72 – 1.62 (m, 2H), 1.40 – 1.32 (m, 2H), 0.95 (t, *J* = 7.4 Hz, 3H). ^{13}C NMR (101 MHz, $CDCl_3$) δ 166.27, 163.05, 145.23, 144.49, 140.60, 139.67, 134.39, 128.48, 127.09, 122.05, 59.04, 39.33, 39.16, 38.66, 36.90, 32.77, 30.98,

30.55, 29.84, 28.06, 20.20, 13.73. MS (ESI+) m/z calculated for $C_{24}H_{28}O_5N_2$: 424.2; found 447.3 $[M + Na]^+$.

Compound **9h**:

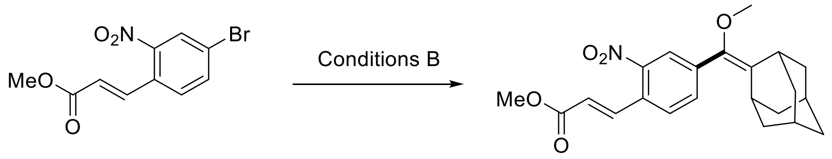

Conditions B were used for the synthesis of compound **9h**. Yield = 81%. ^1H NMR (400 MHz, $CDCl_3$) δ 8.08 (d, *J* = 15.8 Hz, 1H), 7.95 (d, *J* = 1.6 Hz, 1H), 7.63 – 7.52 (m, 2H), 6.39 (d, *J* = 15.8 Hz, 1H), 3.82 (s, 3H), 3.33 (s, 3H), 3.26 (s, 1H), 2.65 (s, 1H), 2.02 – 1.76 (m, 12H). ^{13}C NMR (101 MHz, $CDCl_3$) δ 166.41, 148.55, 141.21, 139.81, 138.73, 136.45, 133.90, 128.98, 128.78, 125.31, 122.66, 58.56, 52.12, 39.26, 39.12, 37.04, 32.53, 30.67, 28.18. MS (ESI+) m/z calculated for $C_{22}H_{25}O_5N$: 383.2; found 384.2 $[M + H]^+$.

Compound **9i**:

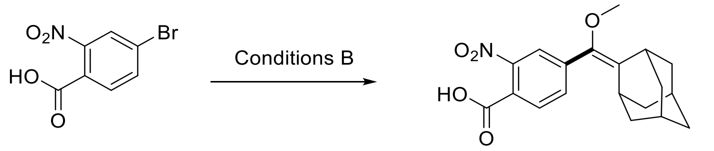

Conditions B were used for the synthesis of compound **9i**. Yield = 32%. ^1H NMR (400 MHz, $CDCl_3$) δ 7.89 (d, *J* = 7.9 Hz, 1H), 7.76 (d, *J* = 1.1 Hz, 1H), 7.61 (dd, *J* = 7.9, 1.3 Hz, 1H), 3.35 (s, 3H), 3.27 (s, 1H), 2.66 (s, 1H), 2.00 – 1.78 (m, 12H). ^{13}C NMR (101 MHz, $CDCl_3$) δ 168.92, 149.58, 141.49, 140.94, 137.62, 132.73, 130.63, 124.26, 123.81, 58.73, 39.30, 39.16, 37.02, 32.57, 30.78, 28.17. MS (ESI+) m/z calculated for $C_{19}H_{21}O_5N$: 343.1; found 344.4 $[M + H]^+$.

Compound **9j**:

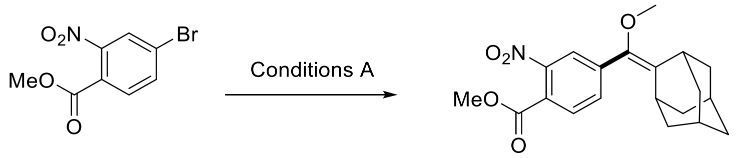

Conditions A were used for the synthesis of compound **9j**. Yield = 22%. ^{1}H NMR (400 MHz, $CDCl_3$) δ 7.81 (d, J = 1.1 Hz, 1H), 7.73 (d, J = 7.9 Hz, 1H), 7.59 (dd, J = 7.9, 1.2 Hz, 1H), 3.92 (s, 3H), 3.32 (s, 3H), 3.26 (s, 1H), 2.63 (s, 1H), 1.99 – 1.58 (m, 12H). ^{13}C NMR (101 MHz, $CDCl_3$) δ 165.81, 148.69, 140.94, 140.37, 136.99, 133.17, 129.91, 125.73, 124.32, 58.60, 53.36, 39.24, 39.11, 37.00, 32.50, 30.67, 28.14. MS (ESI+) m/z calculated for $C_{20}H_{23}O_5N$: 357.2; found 358.4 $[M + H]^+$.

Compound **9k**:

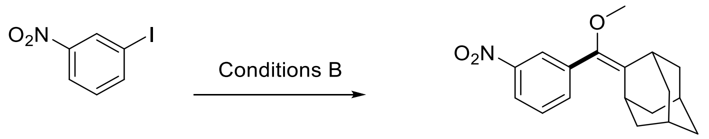

Conditions B were used for the synthesis of compound **9k**. Yield = 82%. ^{1}H NMR (400 MHz, $CDCl_3$) δ 8.17 (d, J = 1.7 Hz, 1H), 8.16 – 8.11 (m, 1H), 7.64 (d, J = 7.6 Hz, 1H), 7.52 (t, J = 7.9 Hz, 1H), 3.32 (s, 3H), 3.28 (s, 1H), 2.59 (s, 1H), 1.97 (dd, J = 15.0, 2.2 Hz, 5H), 1.89 – 1.78 (m, 9H). ^{13}C NMR (101 MHz, $CDCl_3$) δ 148.40, 141.67, 137.57, 135.36, 134.96, 129.15, 124.20, 122.43, 58.37, 39.28, 39.14, 37.14, 32.49, 30.54, 28.27. MS (ESI+) m/z calculated for $C_{18}H_{21}O_3N$: 299.2; found 300.2 $[M + H]^+$.

Compound **9k**:

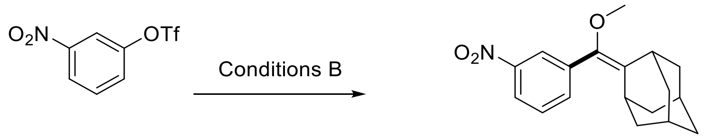

Conditions B were used for the synthesis of compound **9k**. Yield = 69%.

Synthesis of 7-amino coumarin dioxetane:

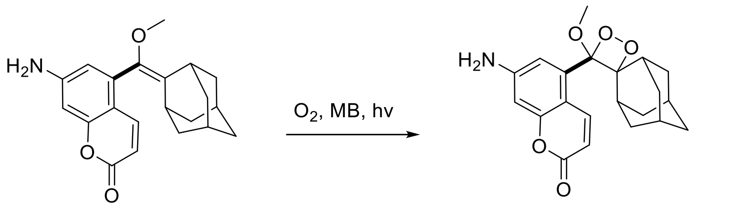

9f **7-amino coumarin dioxetane**

Enol-ether **9f** (20 mg, 0.059 mmol, 1 eq) and catalytic amount (~2 mg) of methylene blue were dissolved in 10 mL of DCM. Oxygen was bubbled through the solution while irradiating yellow light for 3 min. The reaction progress was monitored by RP-HPLC. Upon completion, the solvent was removed and the crude mixture was then purified by preparative RP-HPLC [50-100% ACN in water (0.1 % TFA), 20 min] to afford **7-amino coumarin dioxetane** as a yellow solid (16.5 mg, 76%). ^{1}H NMR (400 MHz, DMSO) δ 8.21 (d, *J* = 9.9 Hz, 1H), 7.16 (d, *J* = 2.1 Hz, 1H), 6.48 (d, *J* = 1.9 Hz, 1H), 6.10 (d, *J* = 9.9 Hz, 1H), 3.11 (s, 3H), 2.93 (s, 1H), 2.05 (s, 1H), 1.80 – 1.37 (m, 12H). ^{13}C NMR (101 MHz, DMSO) δ 160.28, 157.68, 152.69, 141.88, 132.32, 115.73, 112.17, 109.46, 105.47, 100.16, 95.25, 49.83, 36.05, 34.55, 33.13, 32.80, 31.88, 31.54, 31.34, 25.70, 25.50. MS (ESI+) m/z calculated for $C_{21}H_{23}O_5N$: 369.2; found 370.4 [M - H]$^{+}$.

Synthesis of Py-dioxetane:

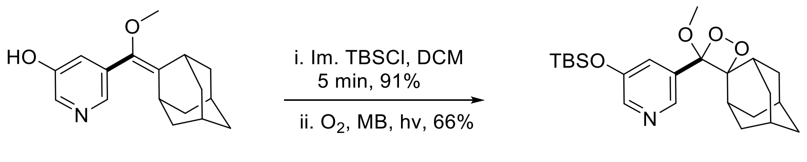

6i **Py-dioxetane**

Mixture of compound **6i** (100 mg, 0.37 mmol, 1 equiv.) and imidazole (38 mg, 0.55 mmol, 1.5 equiv.) was dissolved in DCM (4 mL) and then tert-butyl dimethyl silyl chloride (83 mg, 0.55 mmol, 1.5 equiv.) was added and the reaction mixture was stirred at room temperature for 5 min. Upon completion, pure water was added to the stirring mixture. The mixture was extracted with DCM (3 x 10 mL). The organic layer was dried over anhydrous sodium sulfate, solvent was evaporated and the residue was purified by silica gel column chromatography, to obtain **TBSO-pyridine enol-ether** (140 mg, 91%). ^{1}H NMR (400 MHz, $CDCl_3$) δ 8.12 (s, 1H), 8.08 (d, *J* = 2.3 Hz, 1H), 7.10 (d, *J* = 1.6 Hz, 1H), 3.28 (s, 3H), 3.23 (s, 1H), 2.56 (s, 1H), 1.96 – 1.76 (m, 12H),

0.97 (s, 9H), 0.20 (s, 6H). ^{13}C NMR (101 MHz, $CDCl_3$) δ 152.15, 143.13, 141.20, 140.20, 134.62, 132.22, 127.61, 58.10, 39.28, 39.08, 37.13, 32.44, 30.38, 28.27, 25.67, -4.39. MS (ESI+) m/z calculated for $C_{23}H_{35}O_2NSi$: 385.2; found 386.5 $[M + H]^+$.

TBSO-pyridine enol-ether (20 mg, 0.051 mmol, 1 eq) and catalytic amount (~2 mg) of methylene blue were dissolved in 5 mL of DCM. Oxygen was bubbled through the solution while irradiating yellow light for 3 min. Upon completion, solvent was evaporated and the residue was purified by silica gel column chromatography, to obtain **Py-dioxetane** (14 mg, 66%).^{1}H NMR (400 MHz, $CDCl_3$) δ 8.45 (s, 1H), 8.26 (s, 1H), 7.42 (s, 1H), 3.25 (s, 3H), 3.02 (s, 1H), 2.14 (s, 1H), 1.94 – 1.48 (m, 12H), 0.99 (s, 9H), 0.23 (s, 6H). ^{13}C NMR (101 MHz, $CDCl_3$) δ 152.16, 143.36, 142.65, 131.56, 127.37, 110.71, 95.50, 50.19, 36.36, 34.85, 33.25, 33.01, 32.34, 31.72, 31.54, 29.76, 25.66, -4.34. . MS (ESI+) m/z calculated for $C_{23}H_{35}O_4NSi$: 417.2; found 418.2 $[M + H]^+$.

Synthesis of CyOH dioxetane:

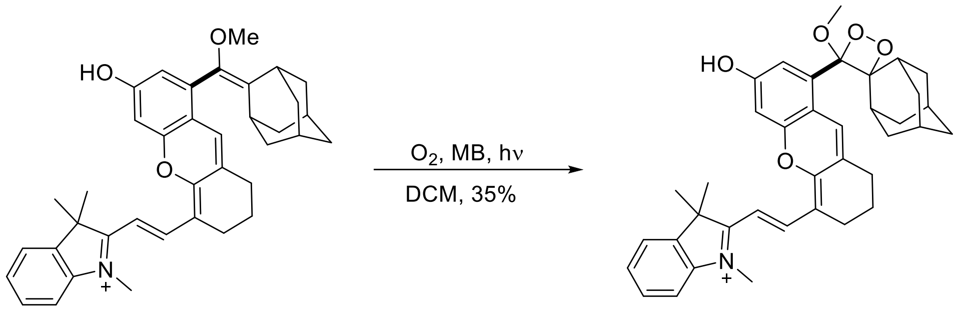

6x **CyOH-dioxetane**

Enol ether (5 mg) was dissolved in DCM (10 mL) and a catalytic amount of methylene blue was added to the mixture (~1 mg). Then, oxygen was bubbled through the solution while irradiating with yellow light. The reaction was monitored by RP-HPLC [70-100% ACN in water (0.1 % TFA), 20 min]. Upon completion, 3 min, the solvent was concentrated under reduced pressure and the product was purified by preparative RP-HPLC [70-100% ACN in water (0.1 % TFA), 20 min]. MS (ESI+) m/z calculated for $C_{38}H_{42}O_5N$: 592.3; found 592.5 $[M]^+$.

RP-HPLC chromatograms of CyOH-dioxetane:

HPLC elution gradient ACN and water with 0.1 TFA (70-100%).

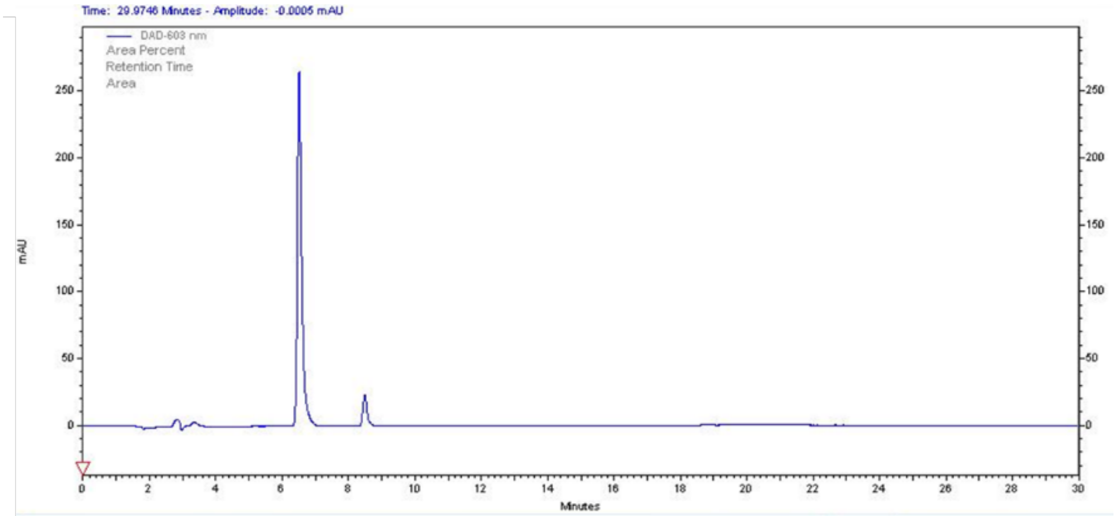

Synthesis of compound 7w:

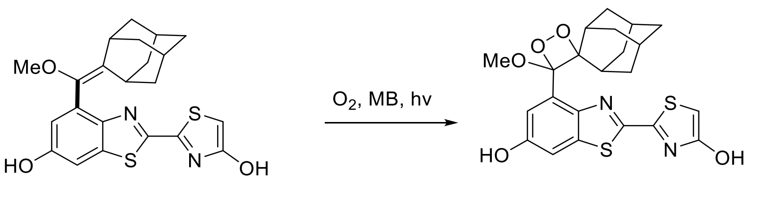

Enol-ether **6w** (20 mg, 0.047 mmol, 1 eq) and catalytic amount (~2 mg) of methylene blue were dissolved in 10 mL of DCM. Oxygen was bubbled through the solution while irradiating with red light for 1.5 h. The reaction progress was monitored by RP-HPLC. Upon completion, the solvent was removed and the crude mixture was then purified by preparative RP-HPLC [50-100% ACN in water (0.1 % TFA), 20 min] to afford **7w** as a white solid (15 mg, 71%). MS (ESI-) m/z calculated for $C_{22}H_{22}O_5N_2S_2$: 458.1; found 457.4 $[M - H]^-$.

RP-HPLC chromatograms of compound 7w:

HPLC elution gradient ACN and water with 0.1 TFA (30-100%).

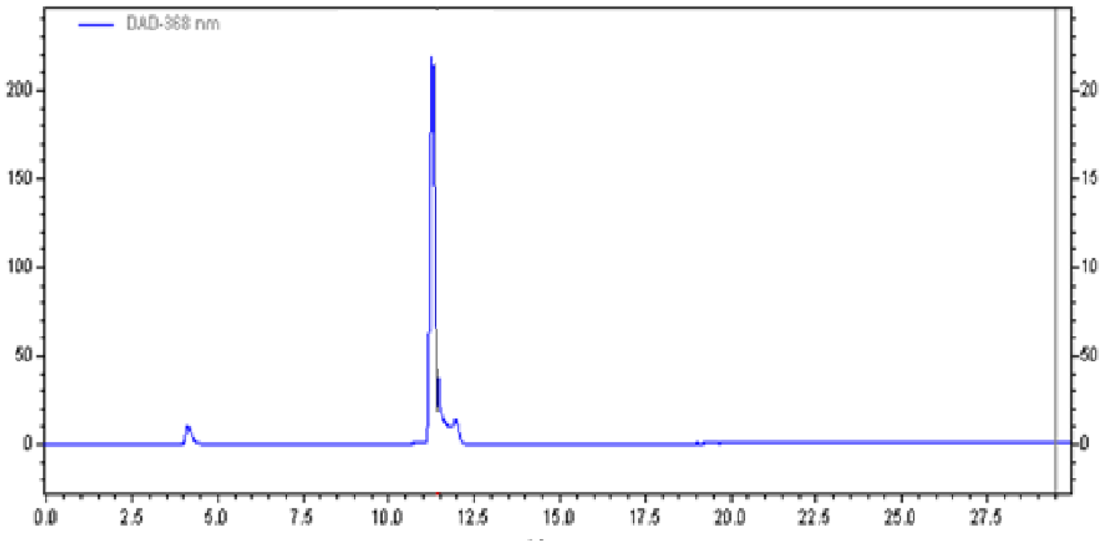
DAD-368 nm
200
150
100
50
0
20
15
10
50
0
0.0
2.5
5.0
7.5
10.0
12.5
15.0
17.5
20.0
22.5
25.0
27.5

3. NMR and MS Spectra:

^{1}H-NMR and ^{13}C-NMR spectra of compound **2:**

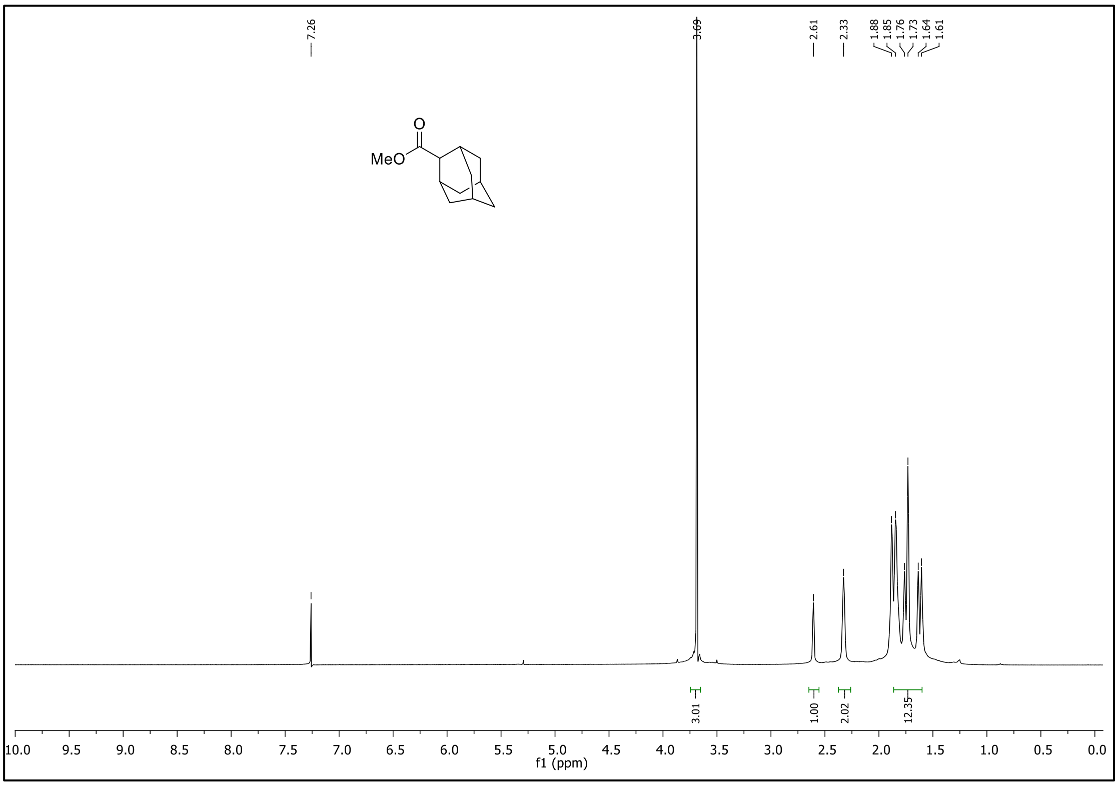

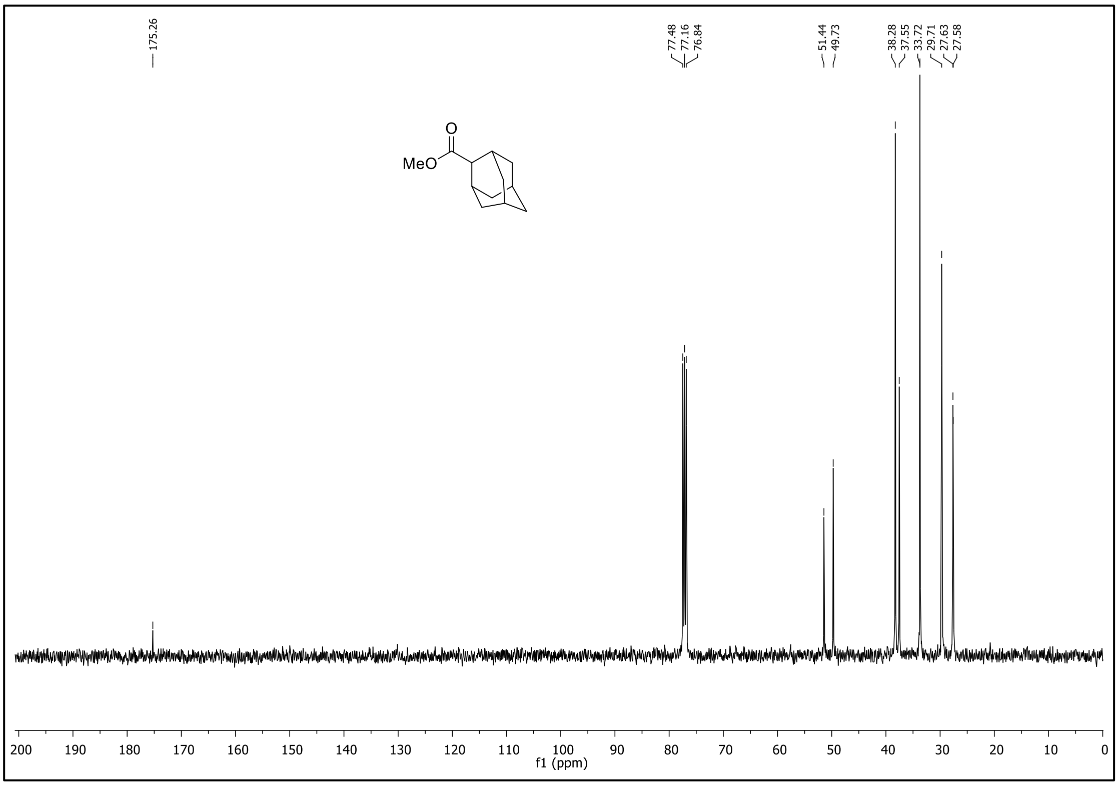

^{1}H-NMR and ^{13}C-NMR spectra of compound **3:**

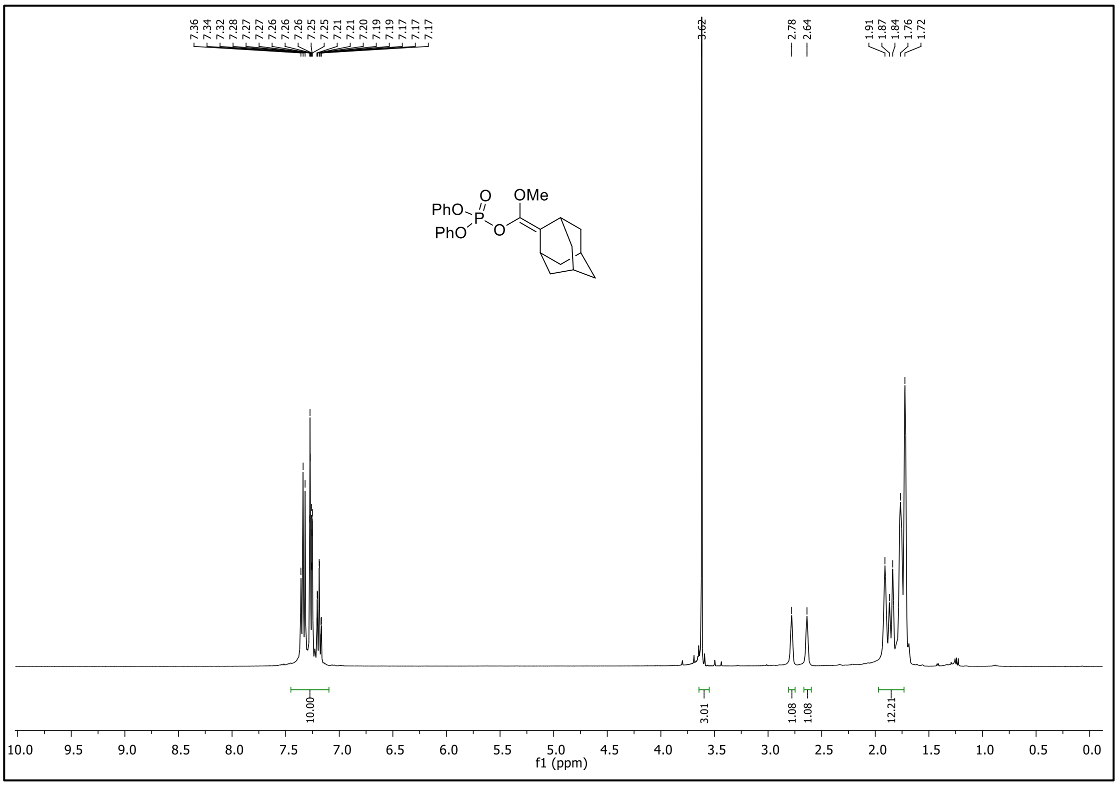

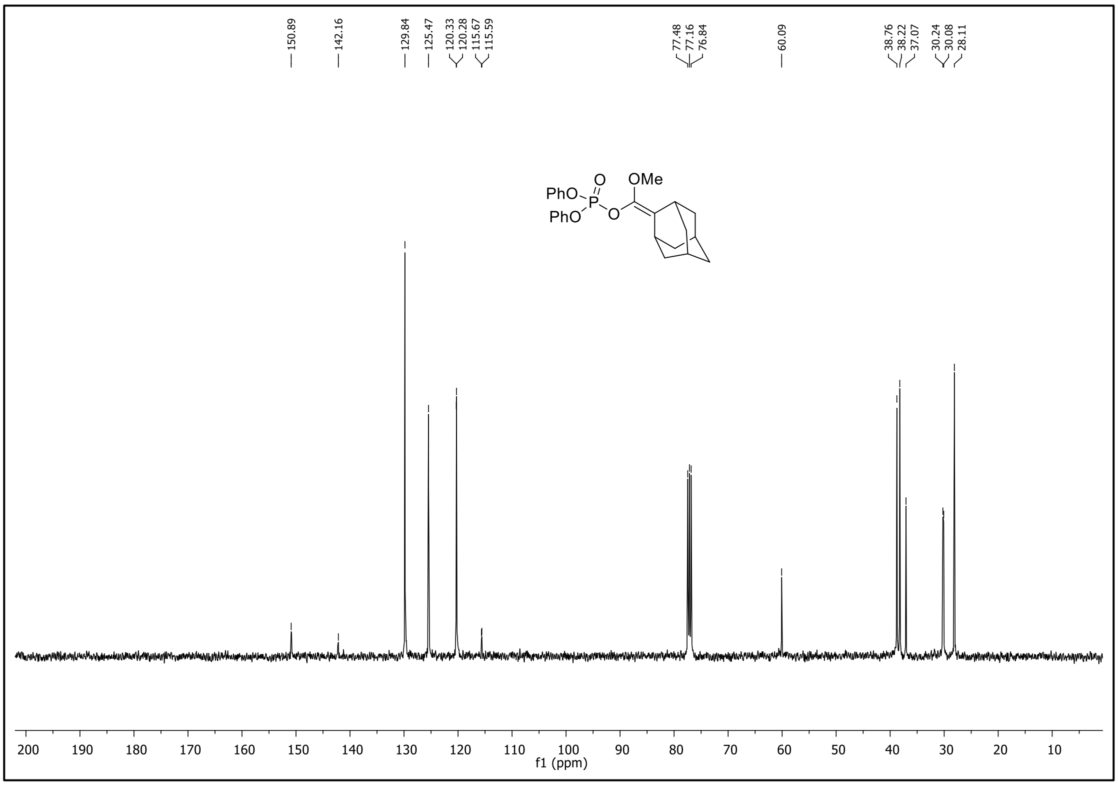

MS of compound **3:**

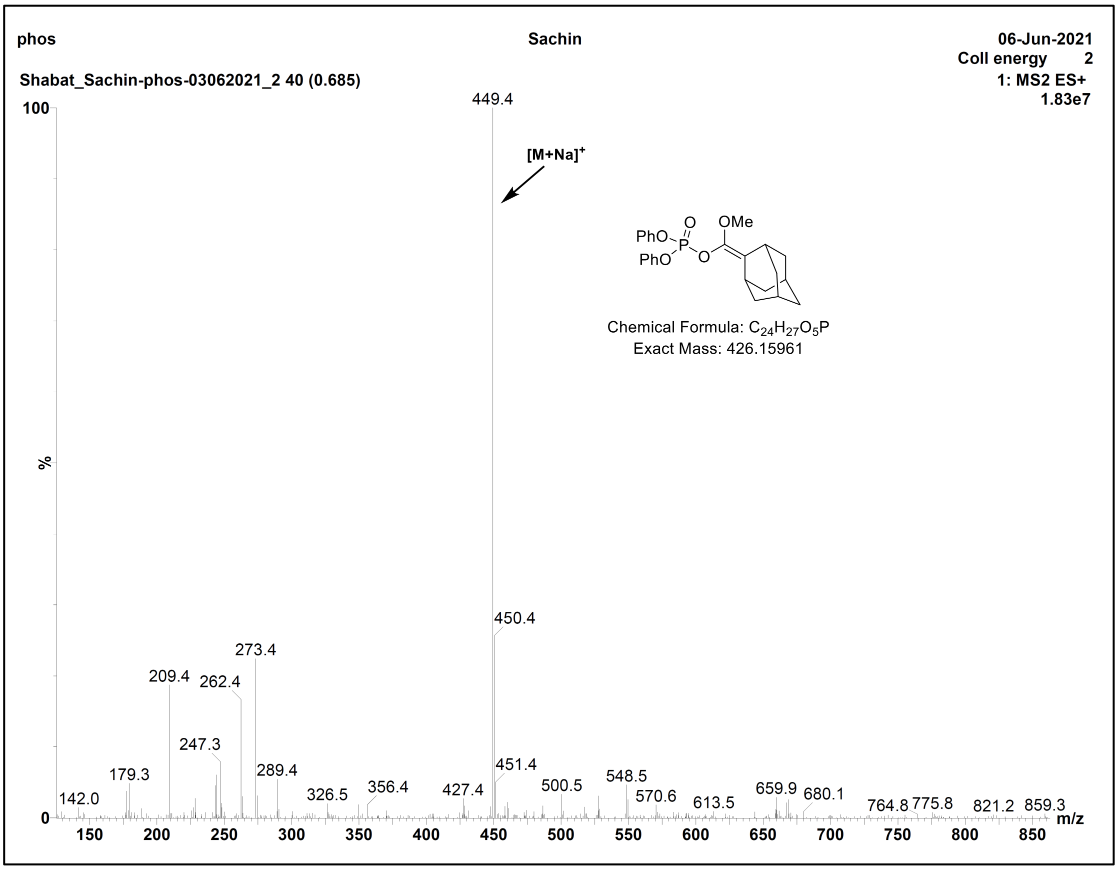

^{1}H-NMR and ^{13}C-NMR spectra of compound **4:**

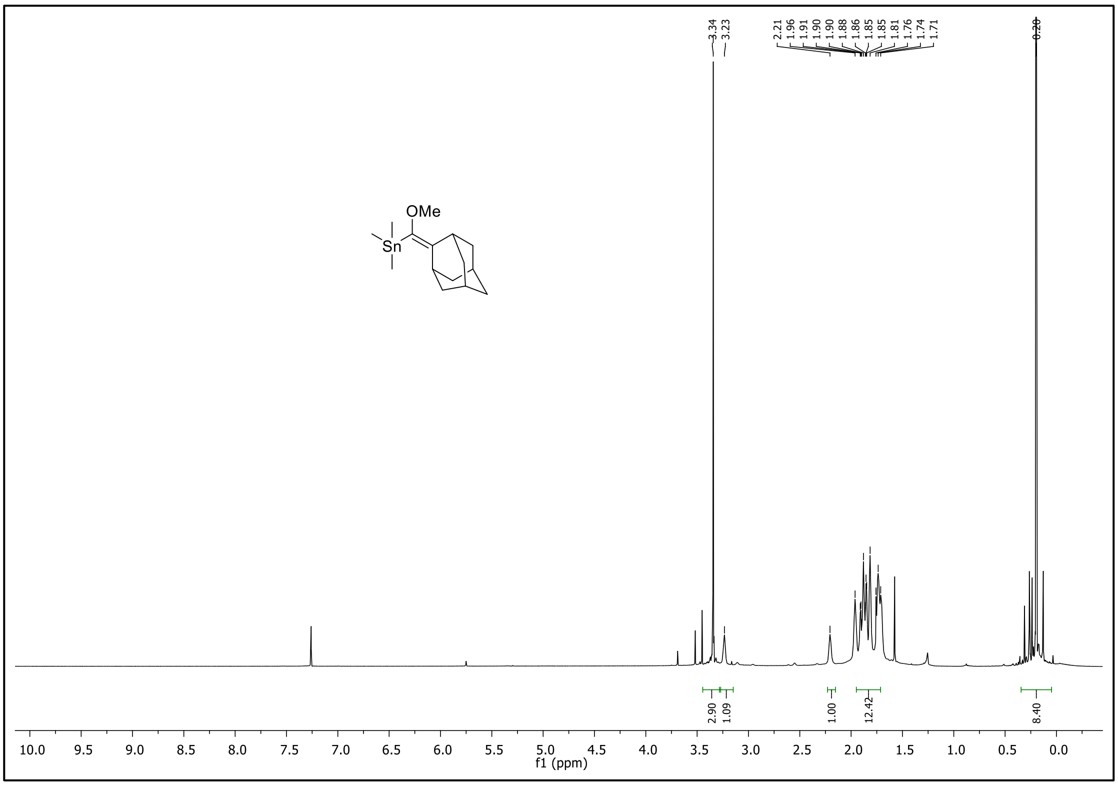

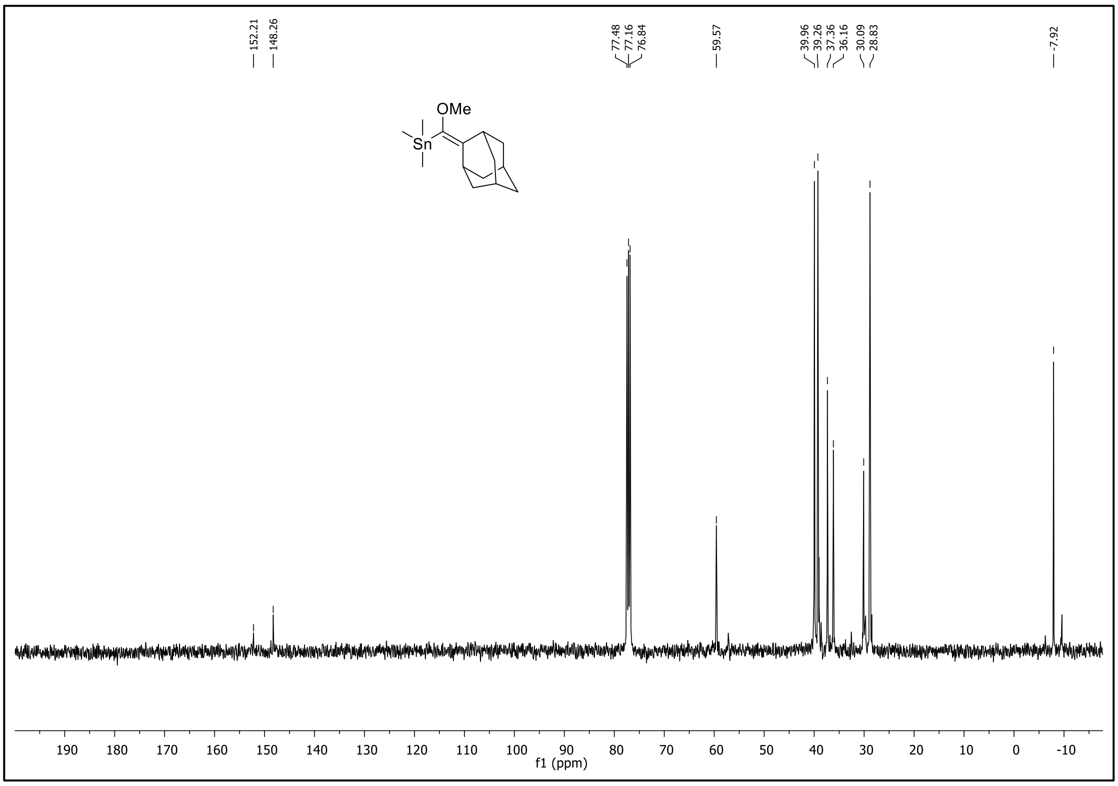

MS of compound **4:**

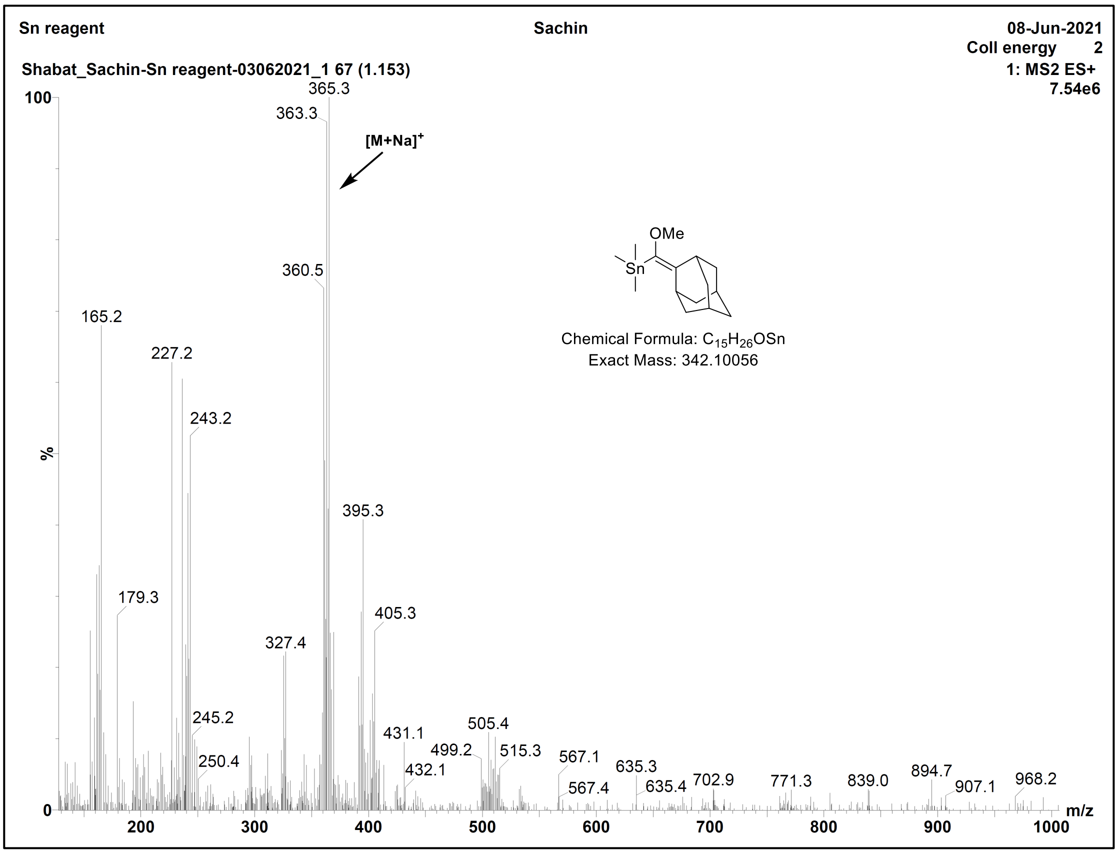

^{1}H-NMR and ^{13}C-NMR spectra of compound **5r:**

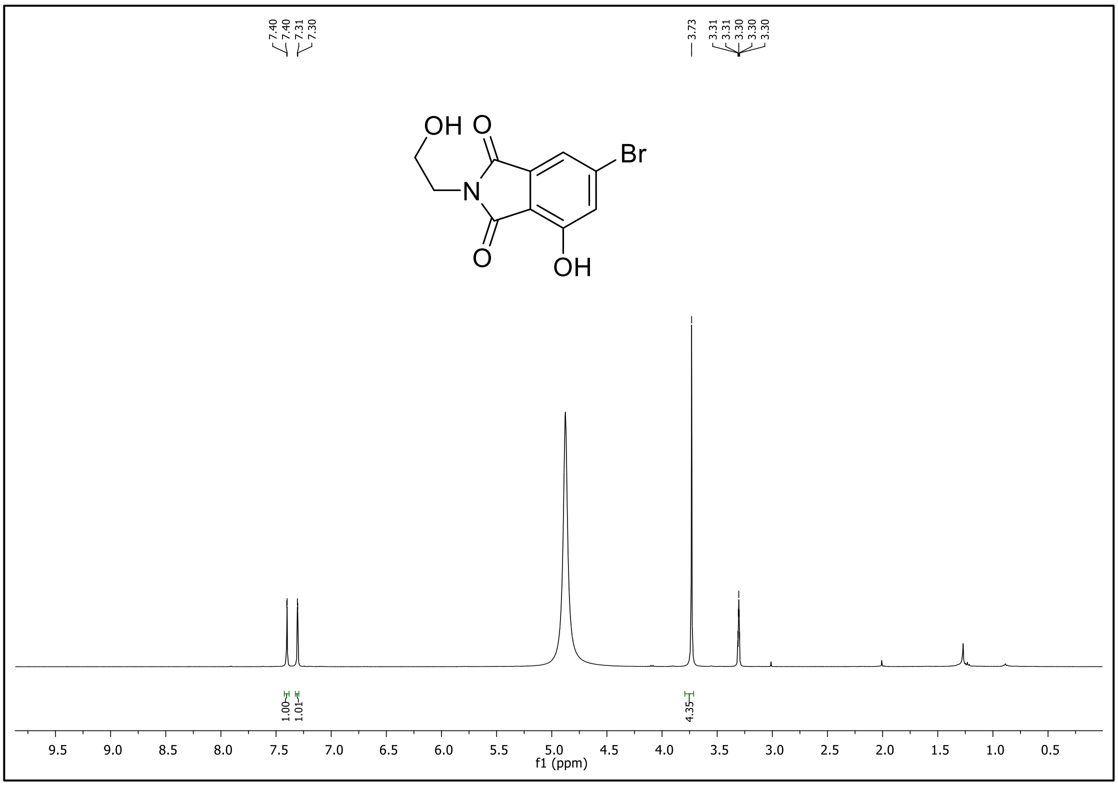

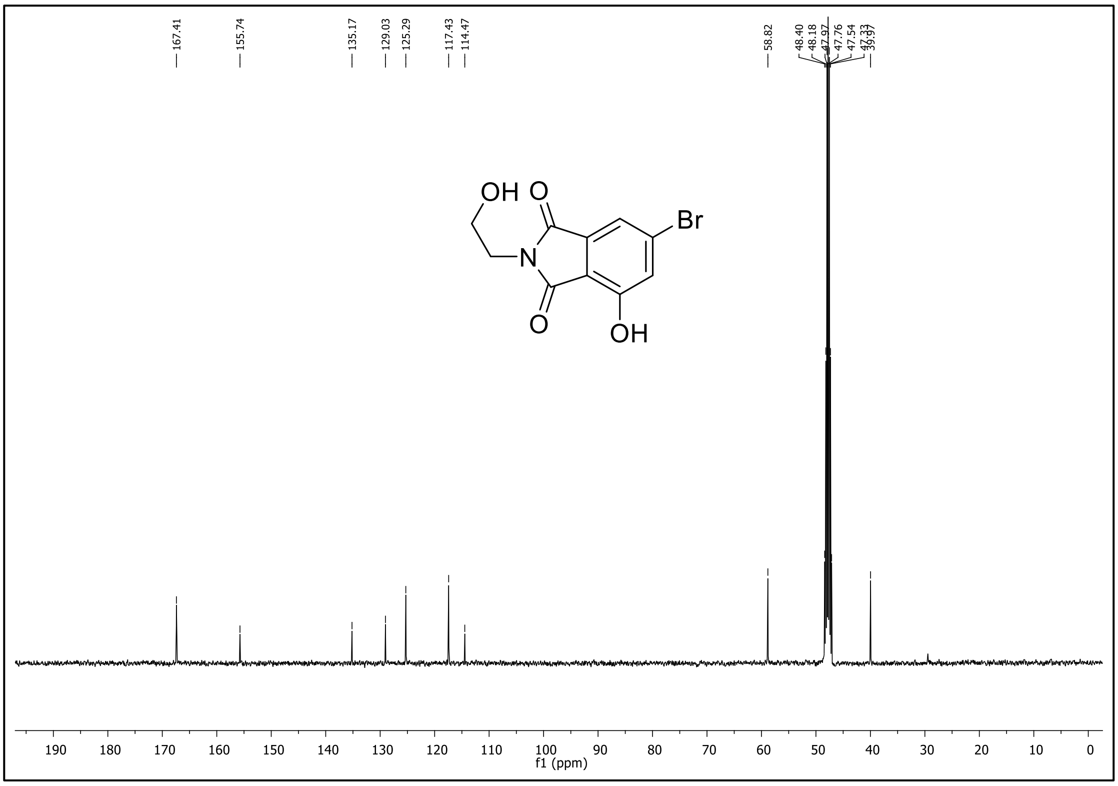

^{1}H-NMR and ^{13}C-NMR spectra of compound **8g:**

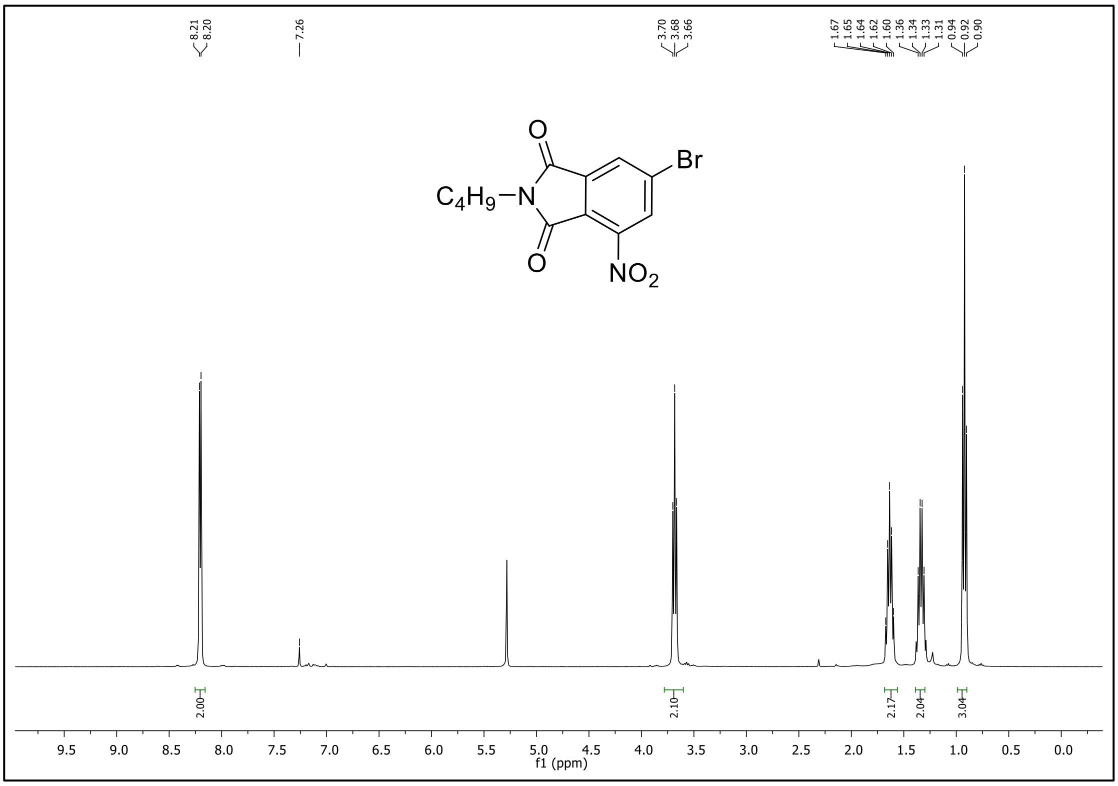

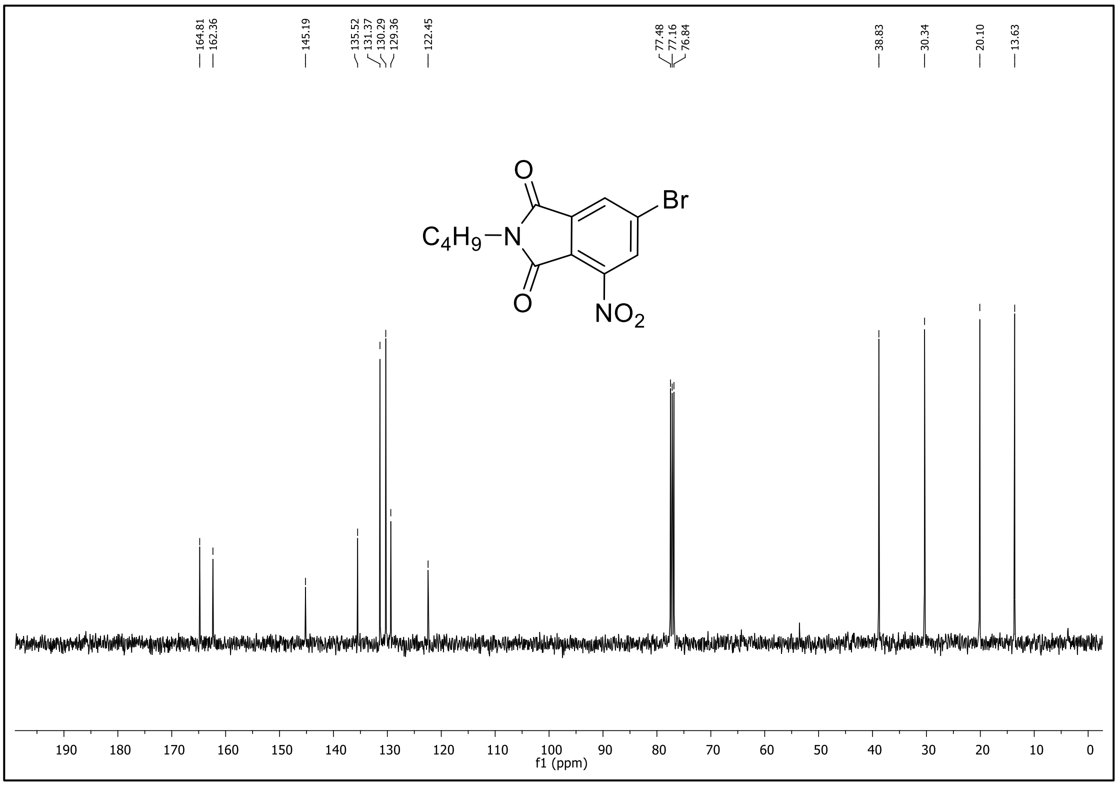

^{1}H-NMR and ^{13}C-NMR spectra of compound **8e:**

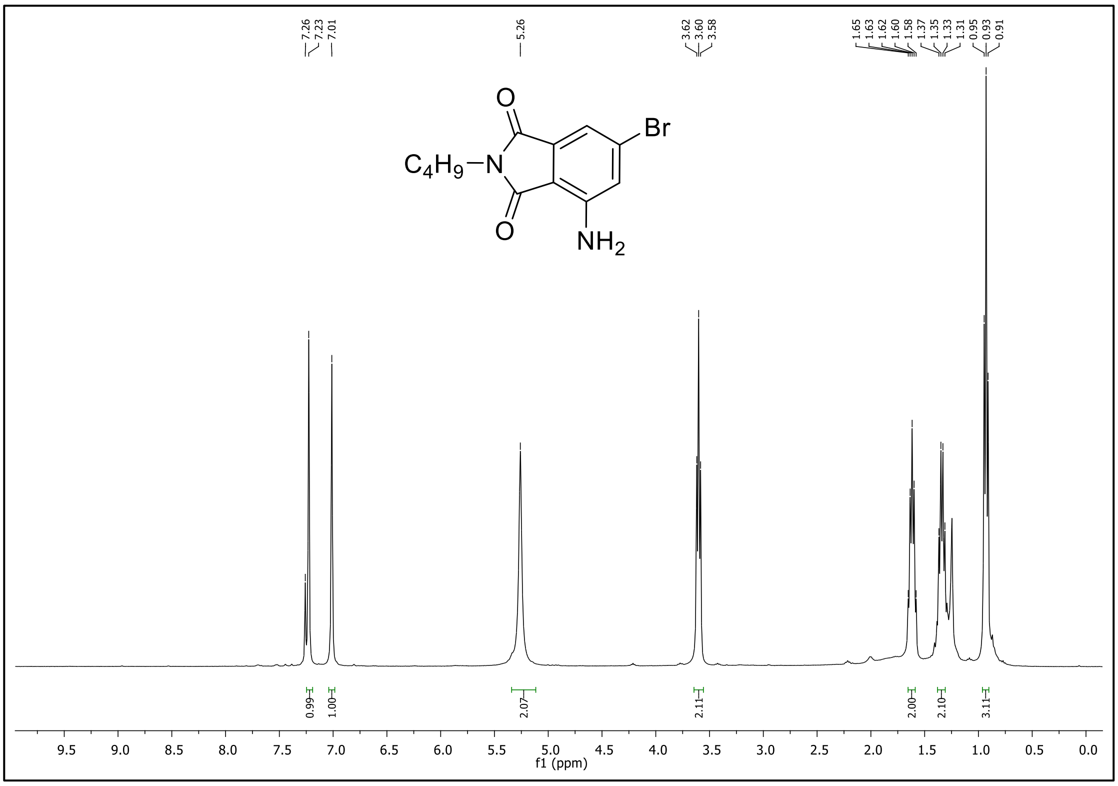

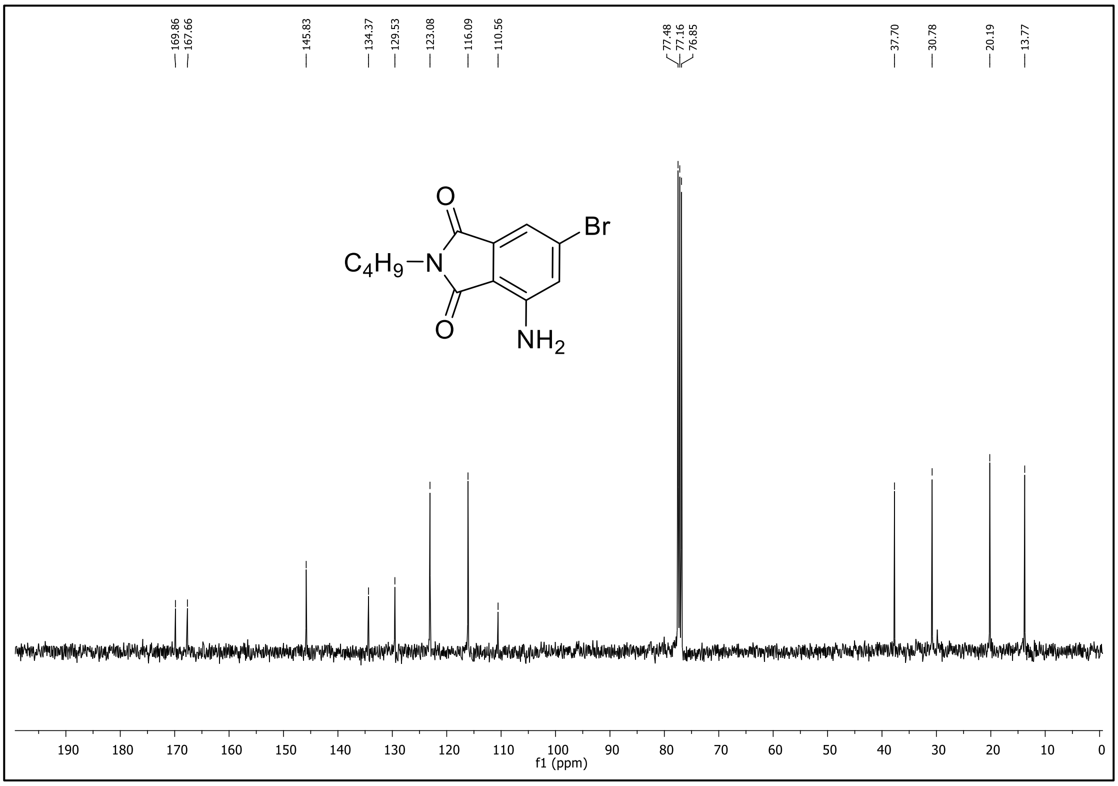

^{1}H-NMR and ^{13}C-NMR spectra of compound **5q:**

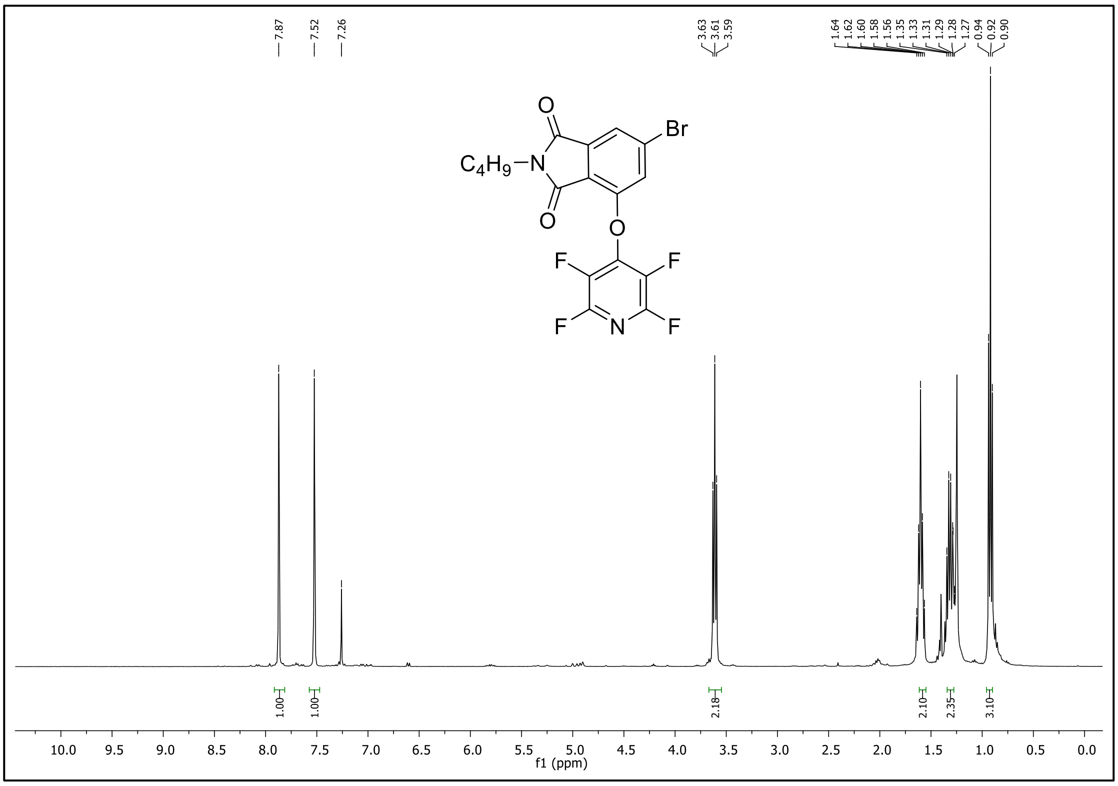

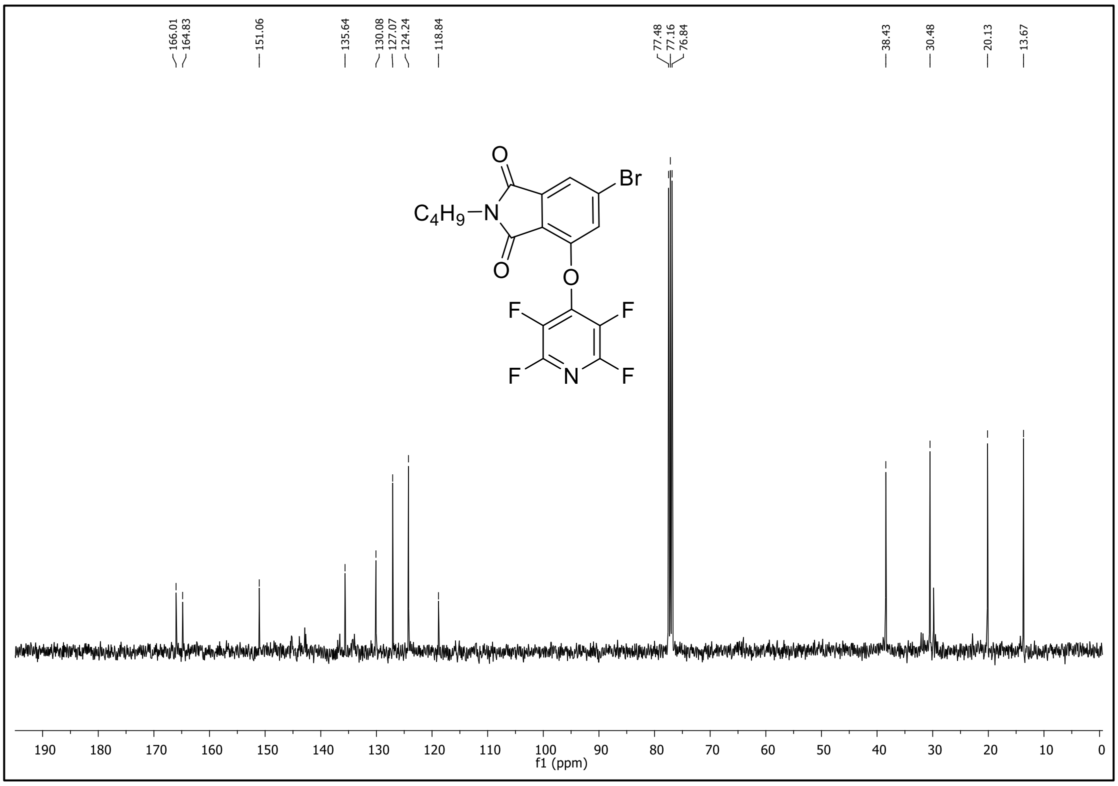

^{19}F-NMR spectra of compound **5q:**

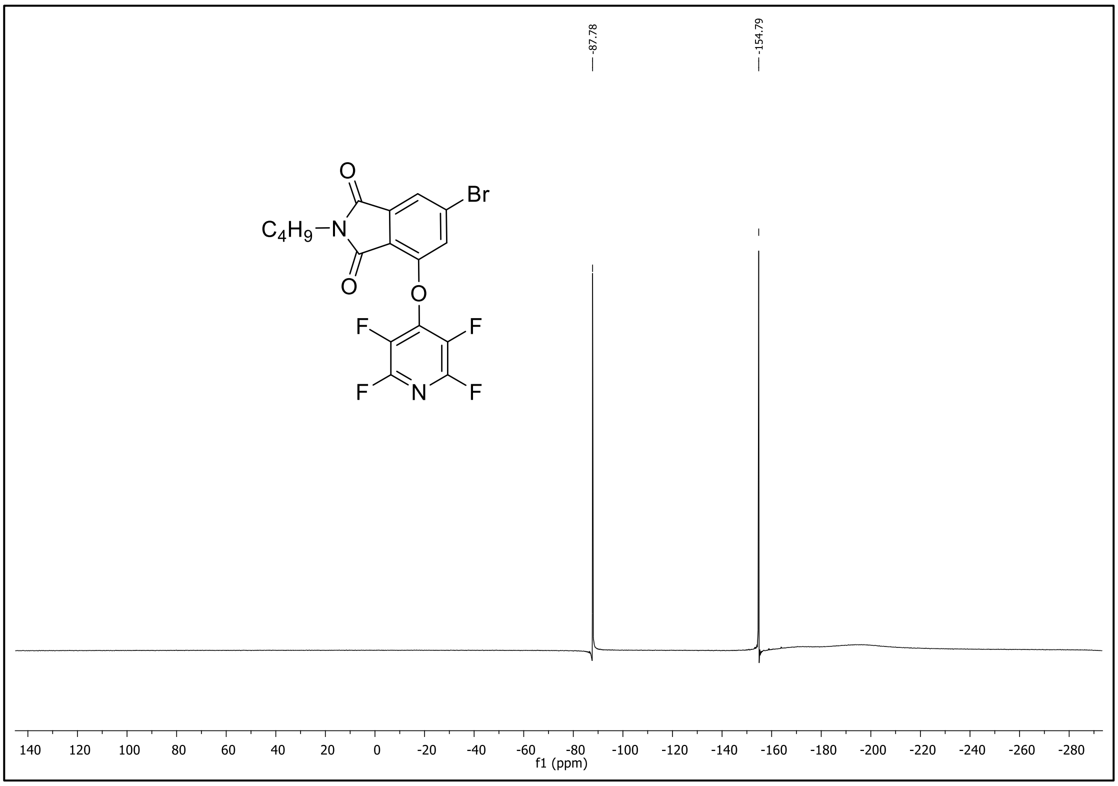

^{1}H-NMR and ^{13}C-NMR spectra of compound **5s:**

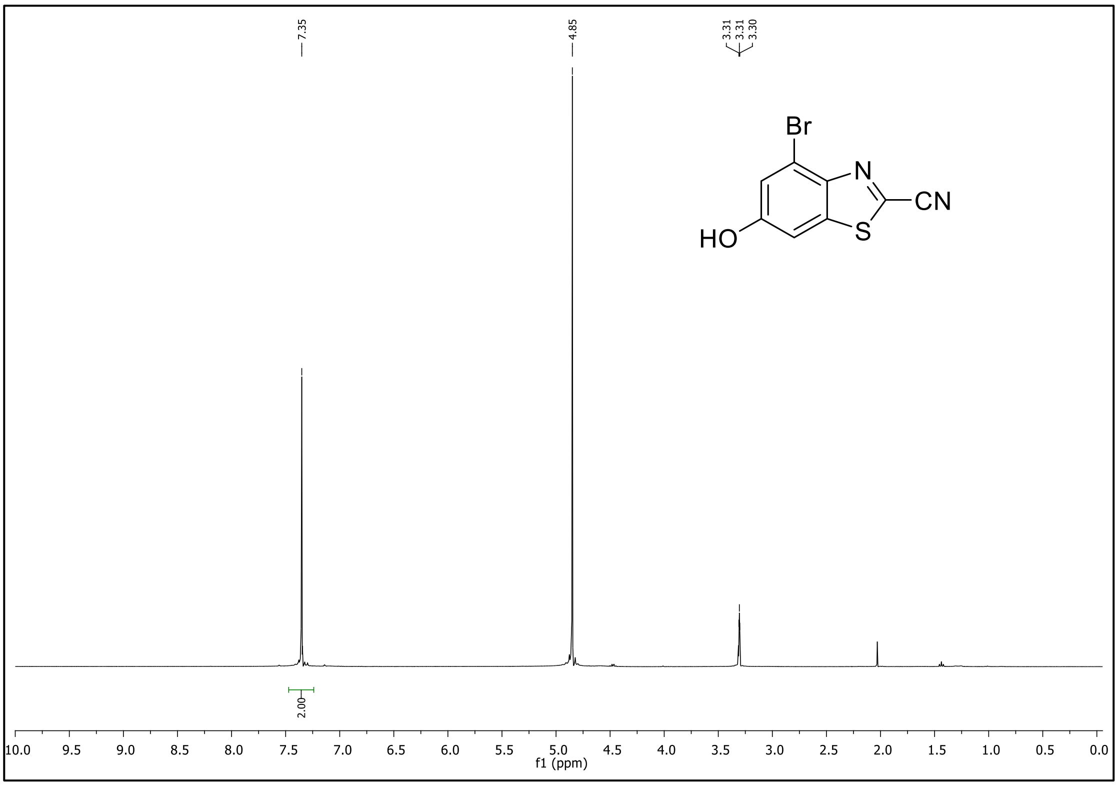

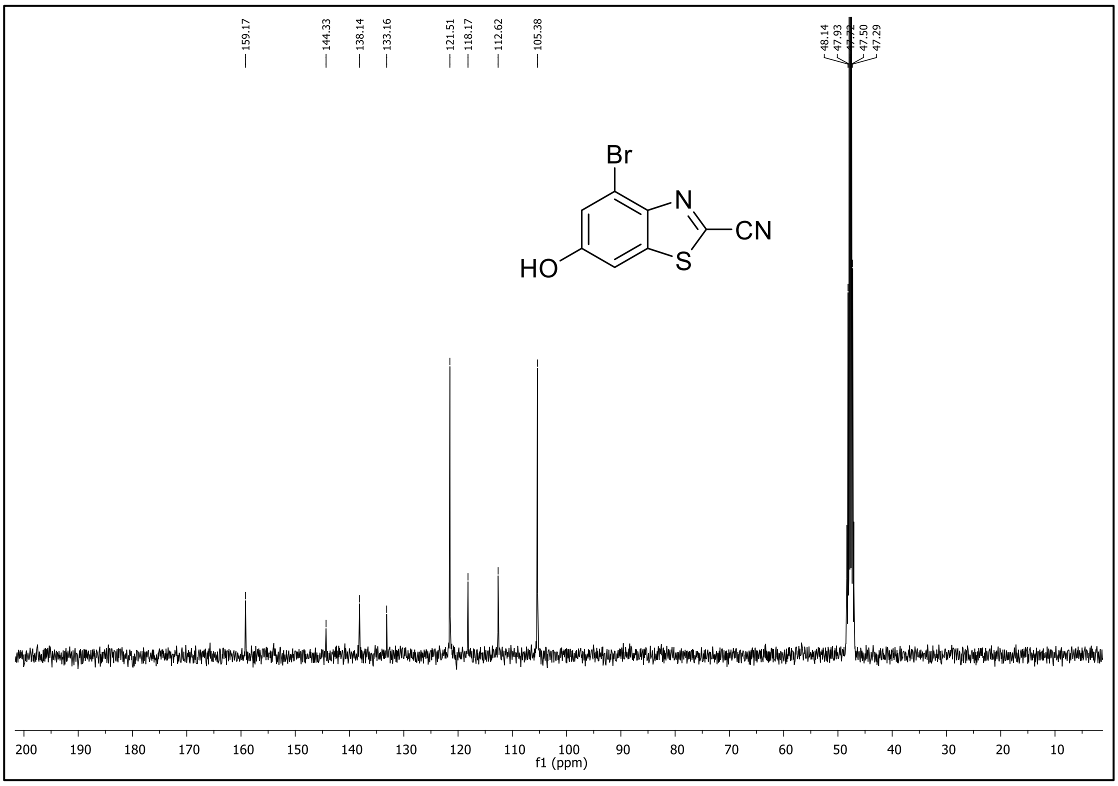

^{1}H-NMR and ^{13}C-NMR spectra of compound **5v:**

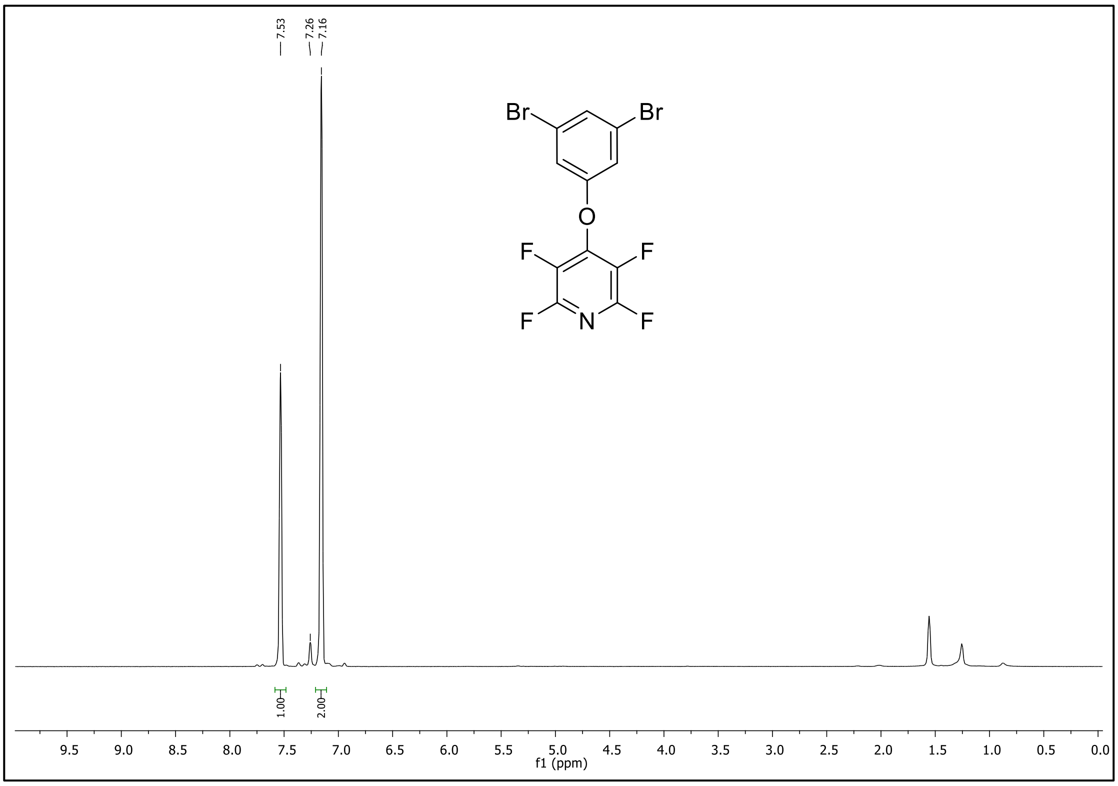

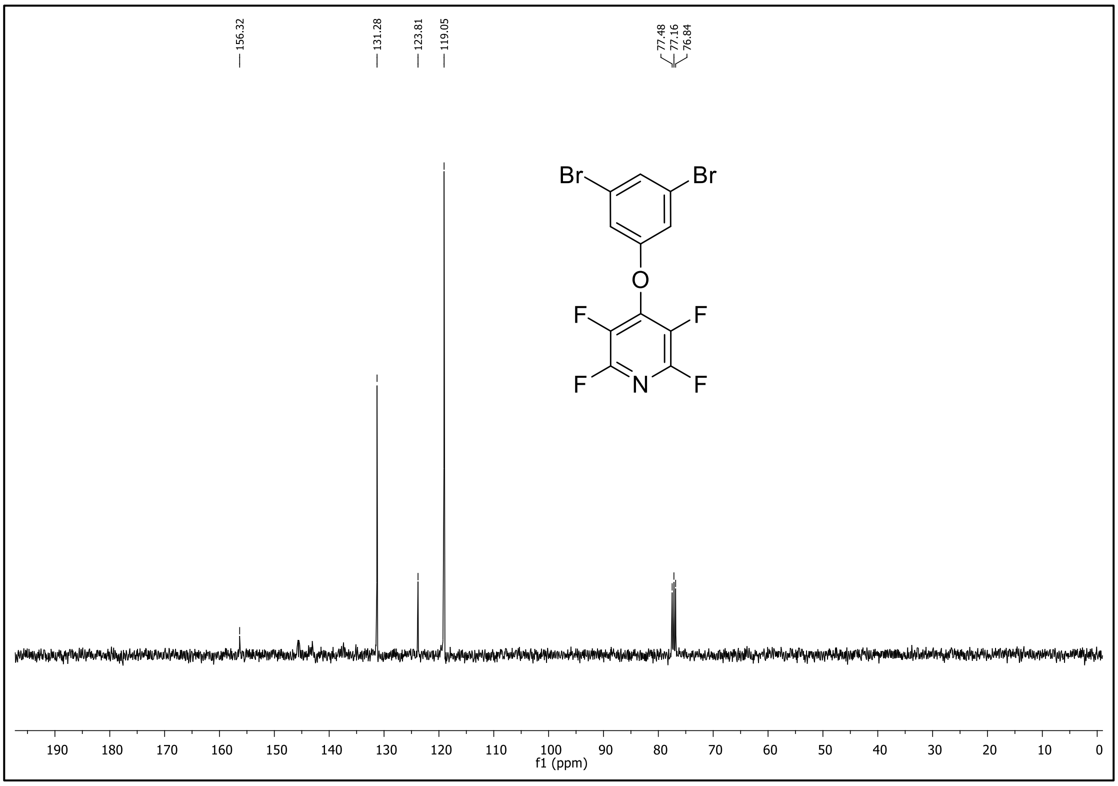

MS of compound **5v:**

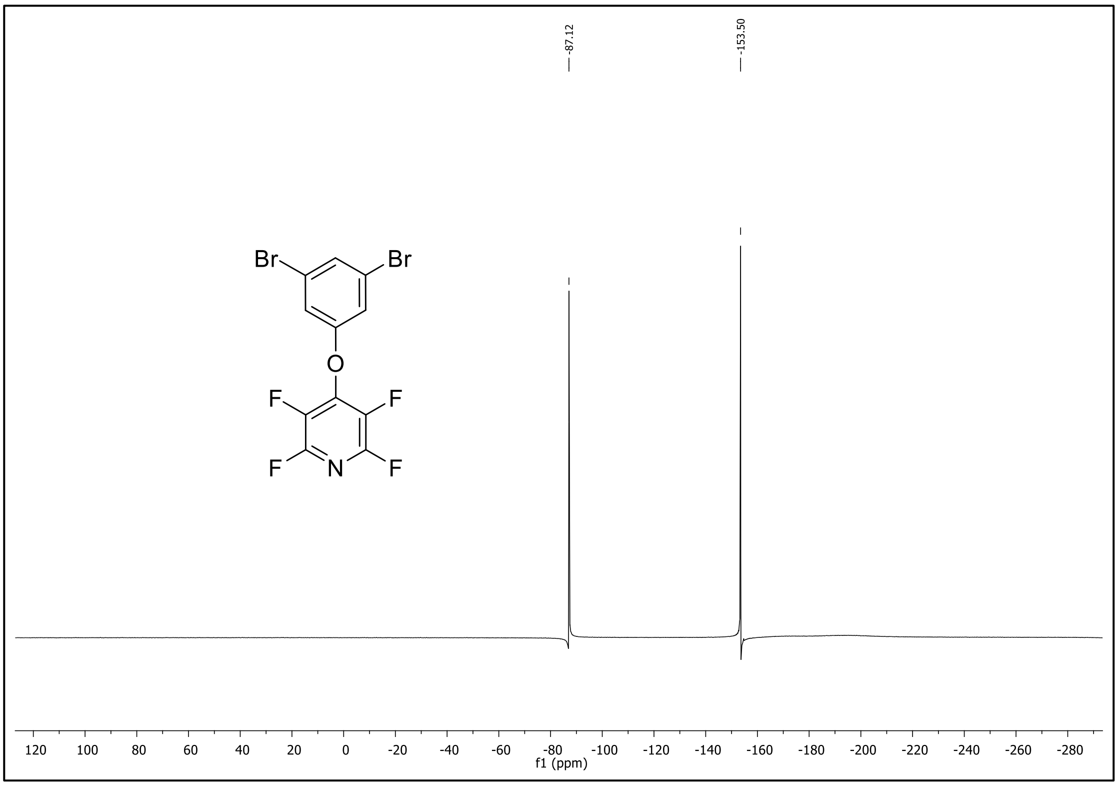

^{1}H-NMR and ^{13}C-NMR spectra of compound **5w:**

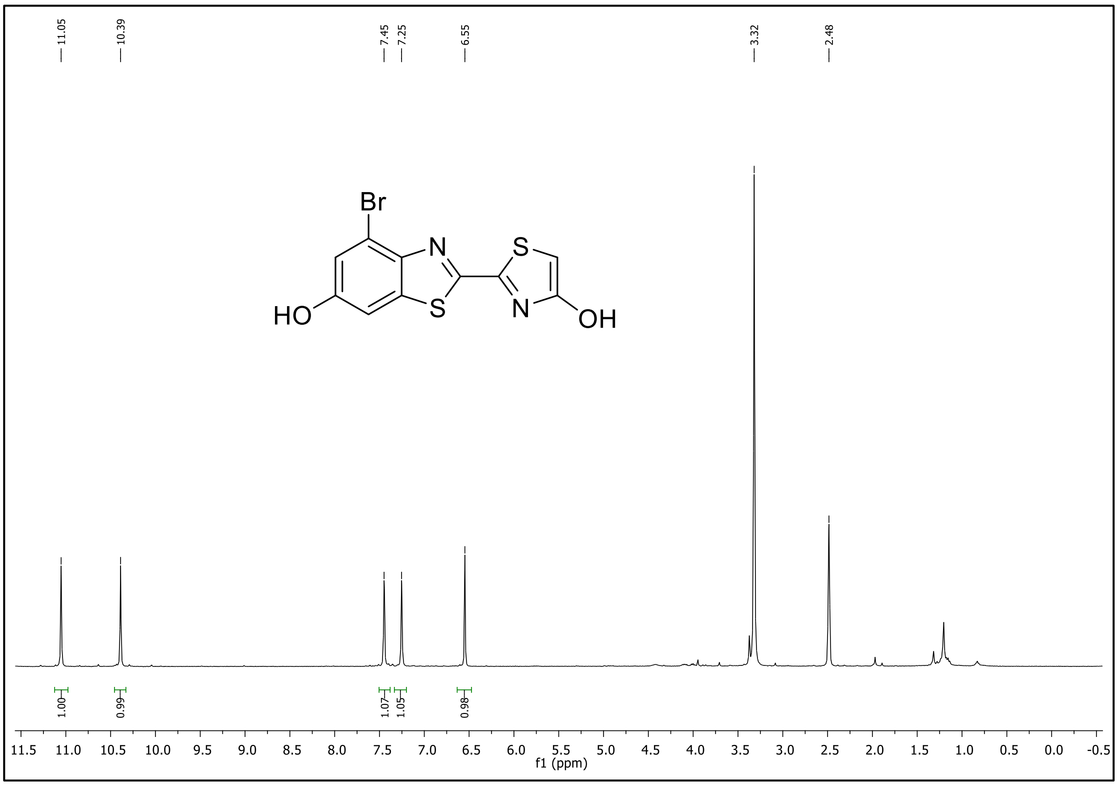

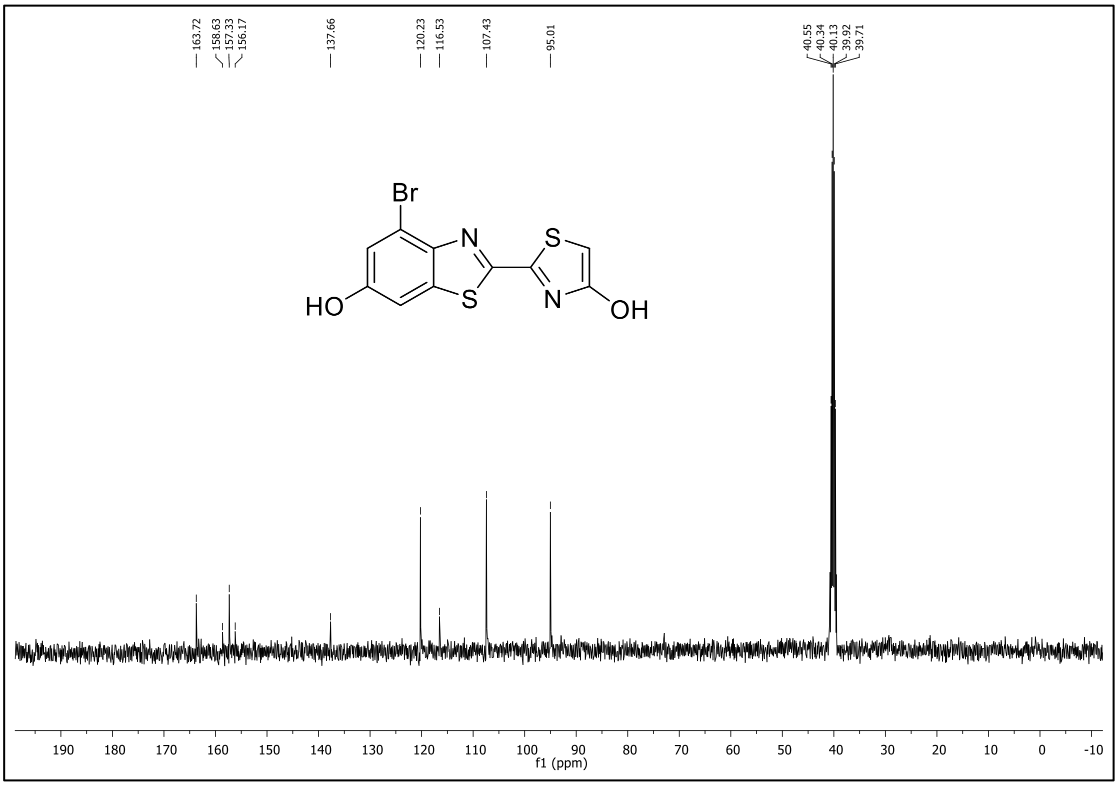

MS of compound **5w:**

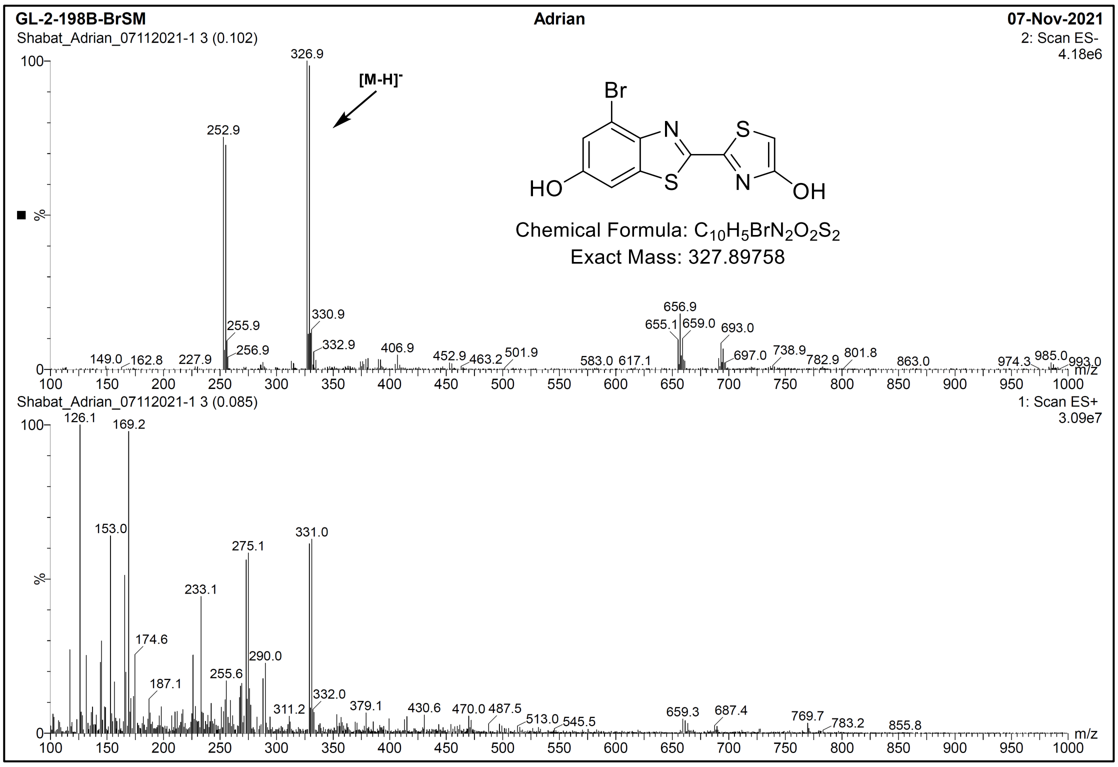

^{1}H-NMR and ^{13}C-NMR spectra of compound **6b:**

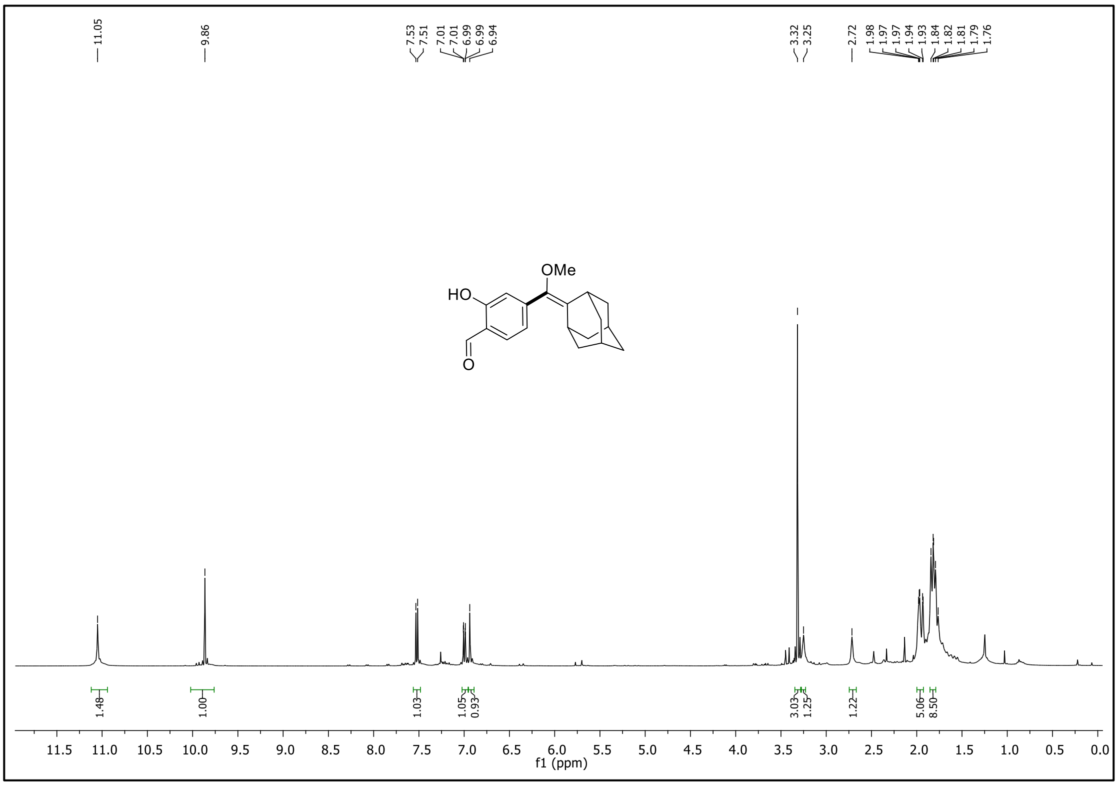

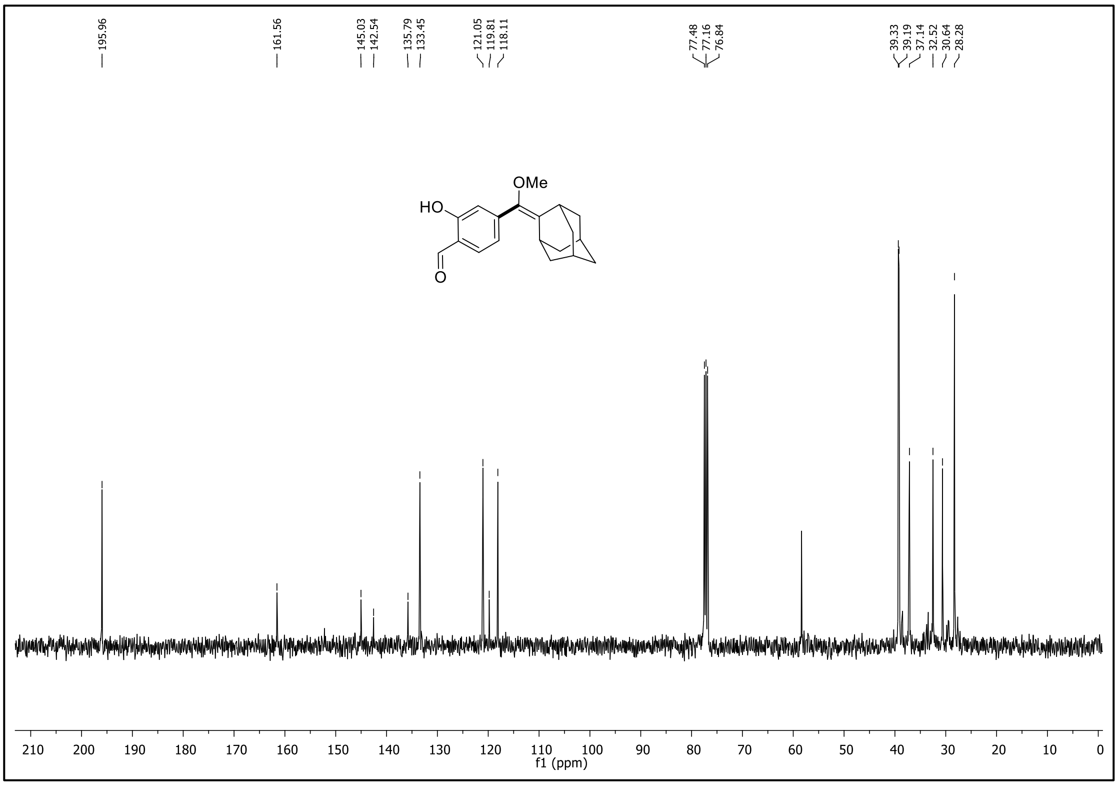

MS of compound **6b:**

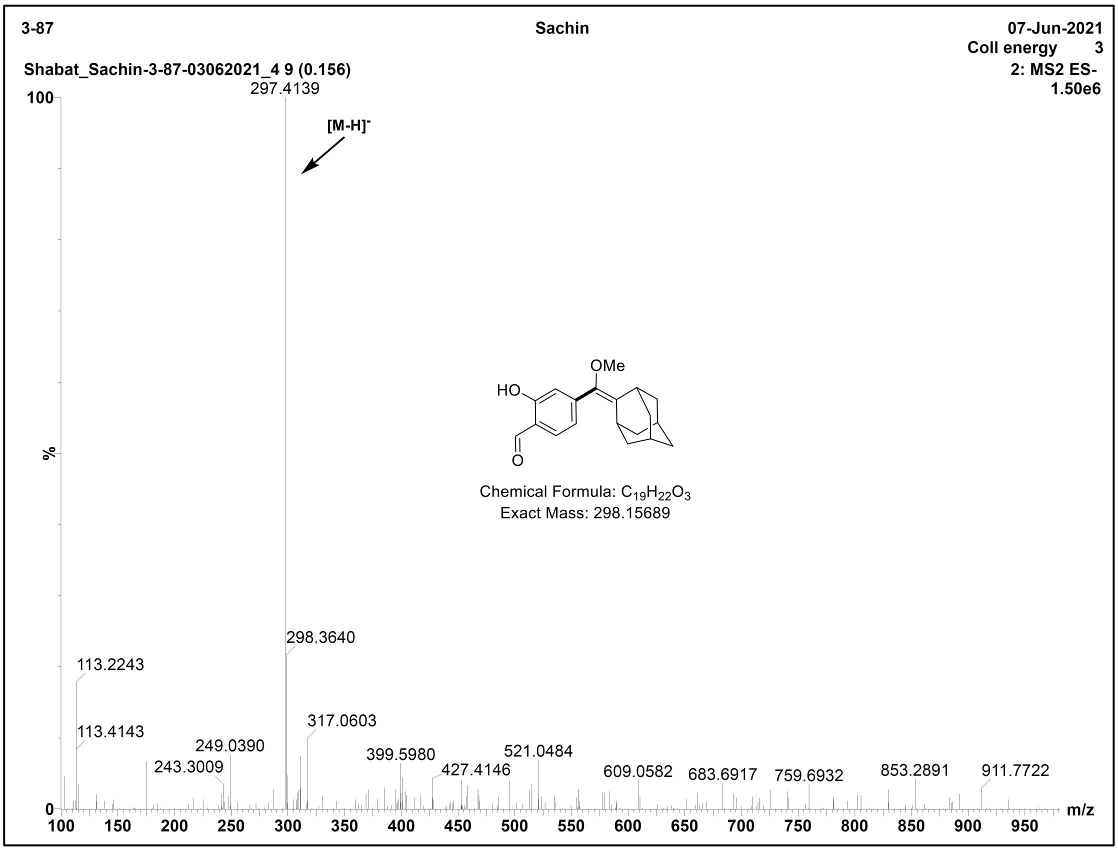

^{1}H-NMR and ^{13}C-NMR spectra of compound **6c:**

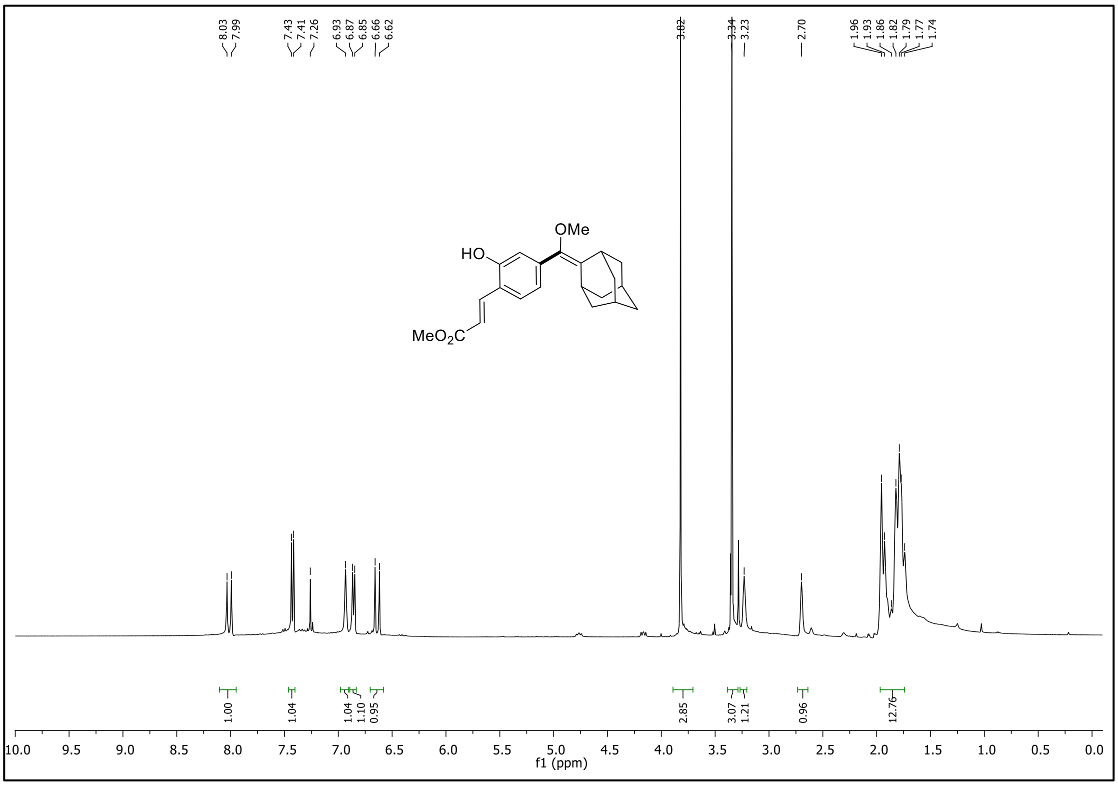

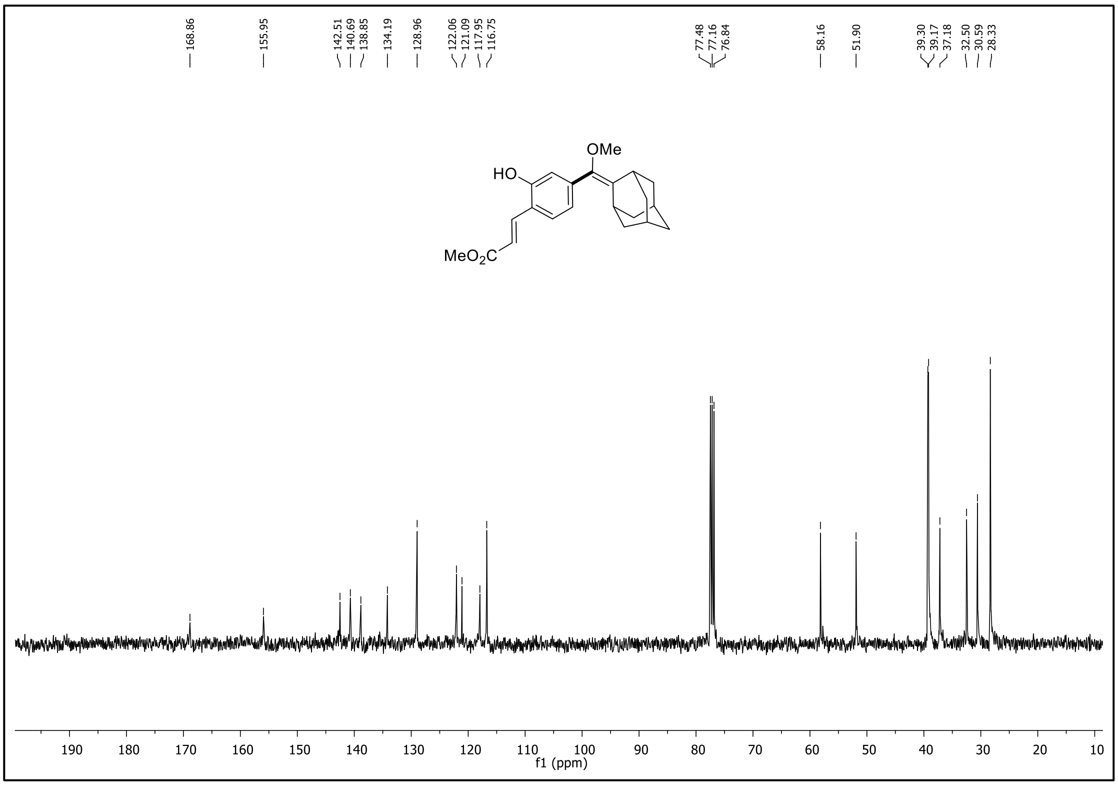

MS of compound **6c:**

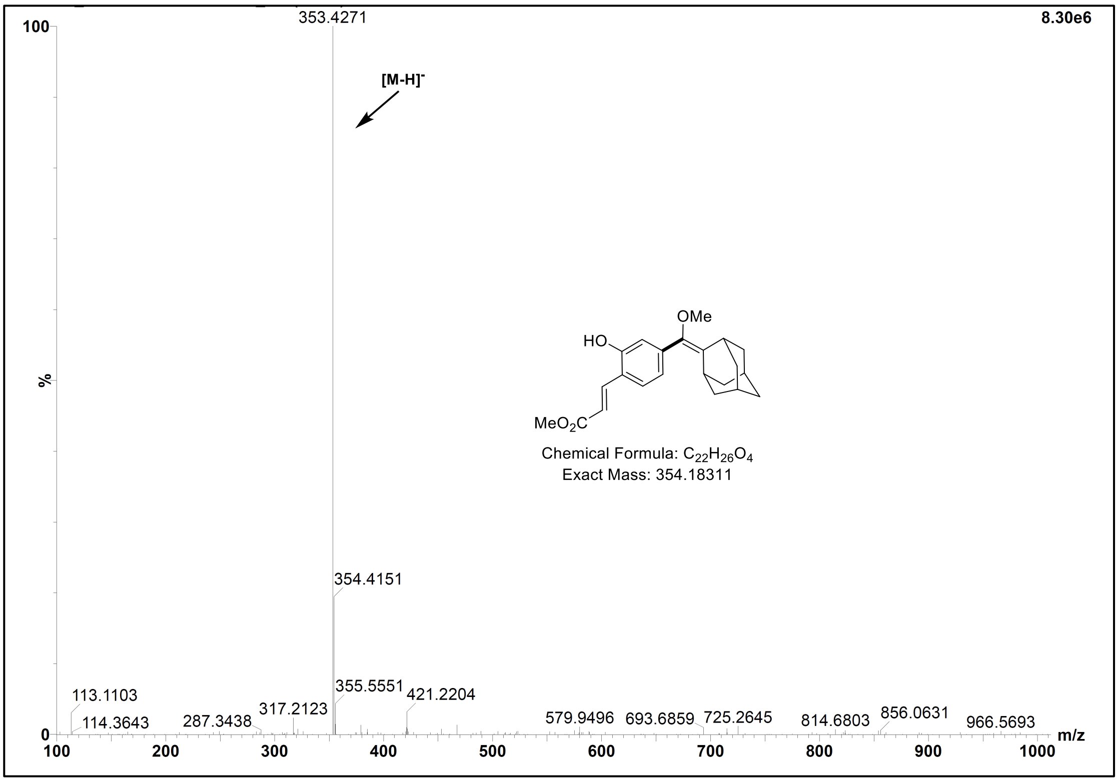

^{1}H-NMR and ^{13}C-NMR spectra of compound **6d:**

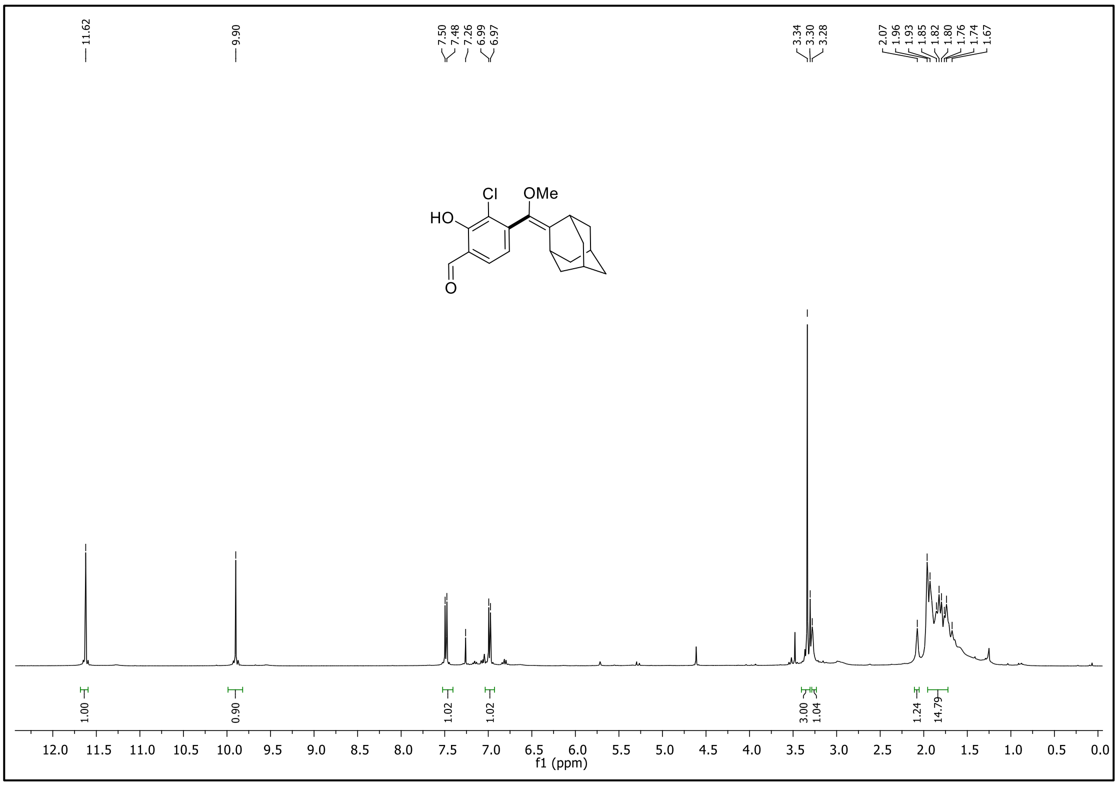

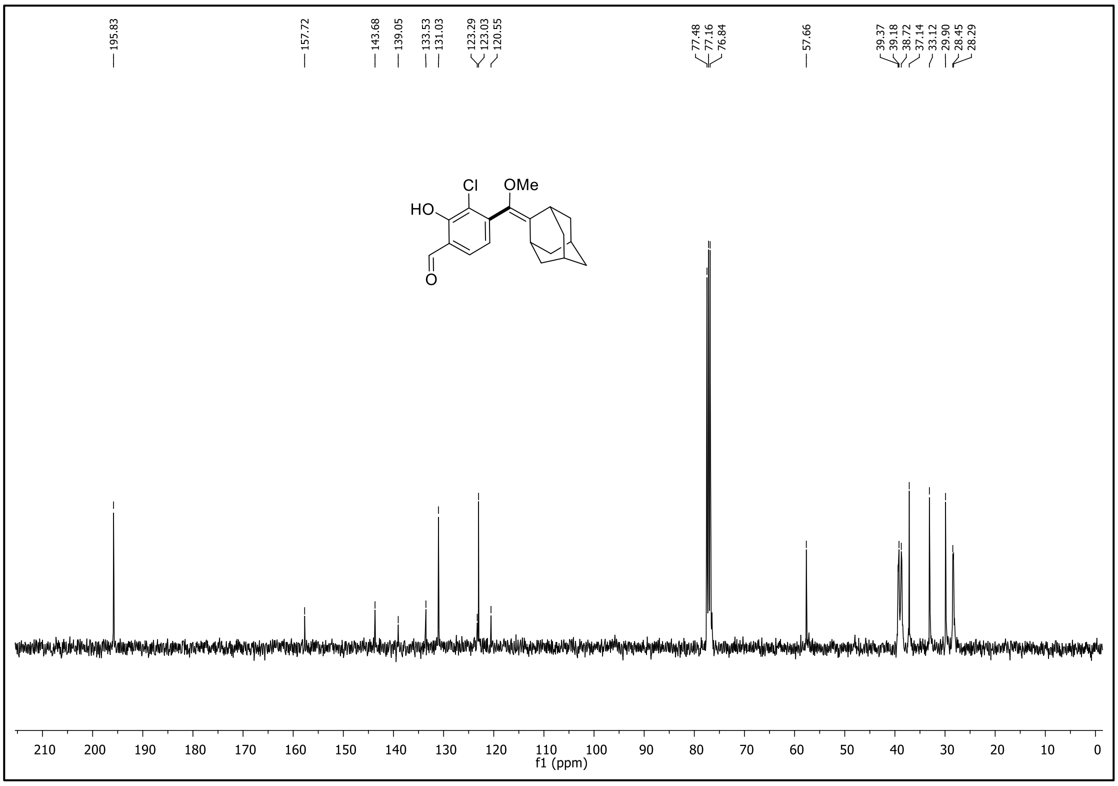

MS of compound **6d:**

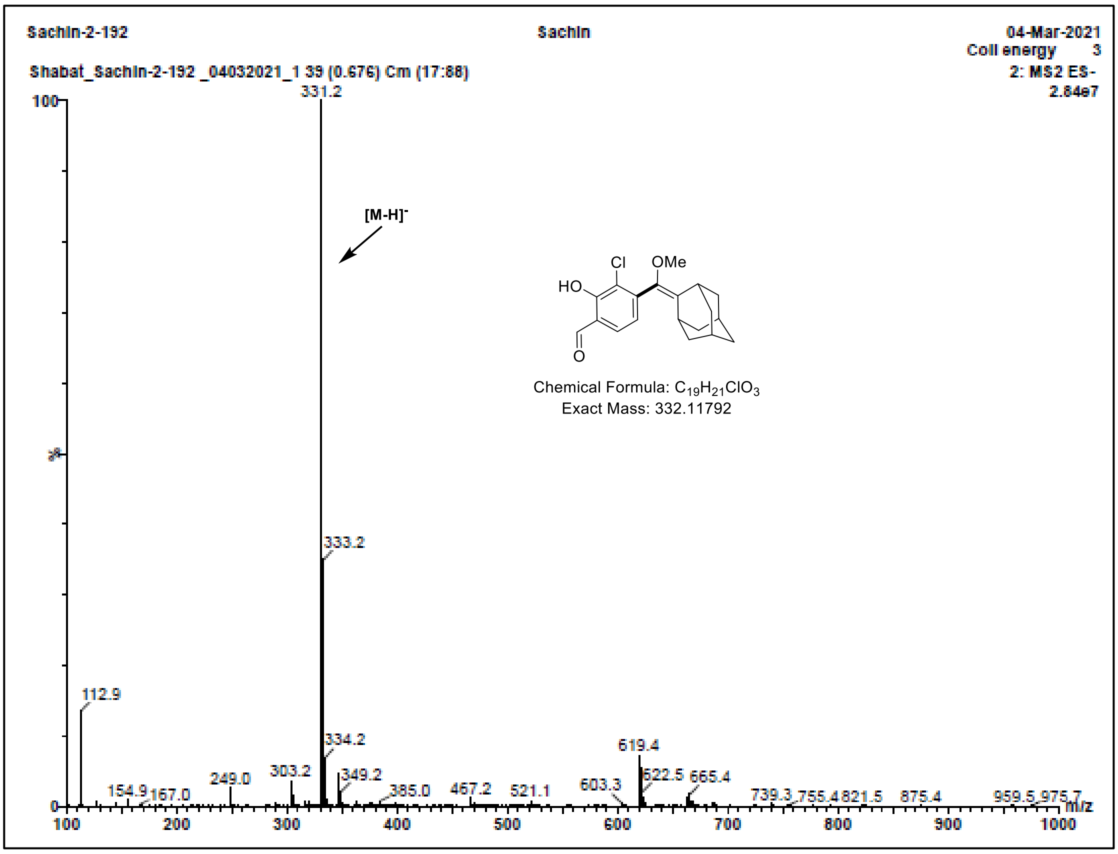

^{1}H-NMR spectra of compound **6e:**

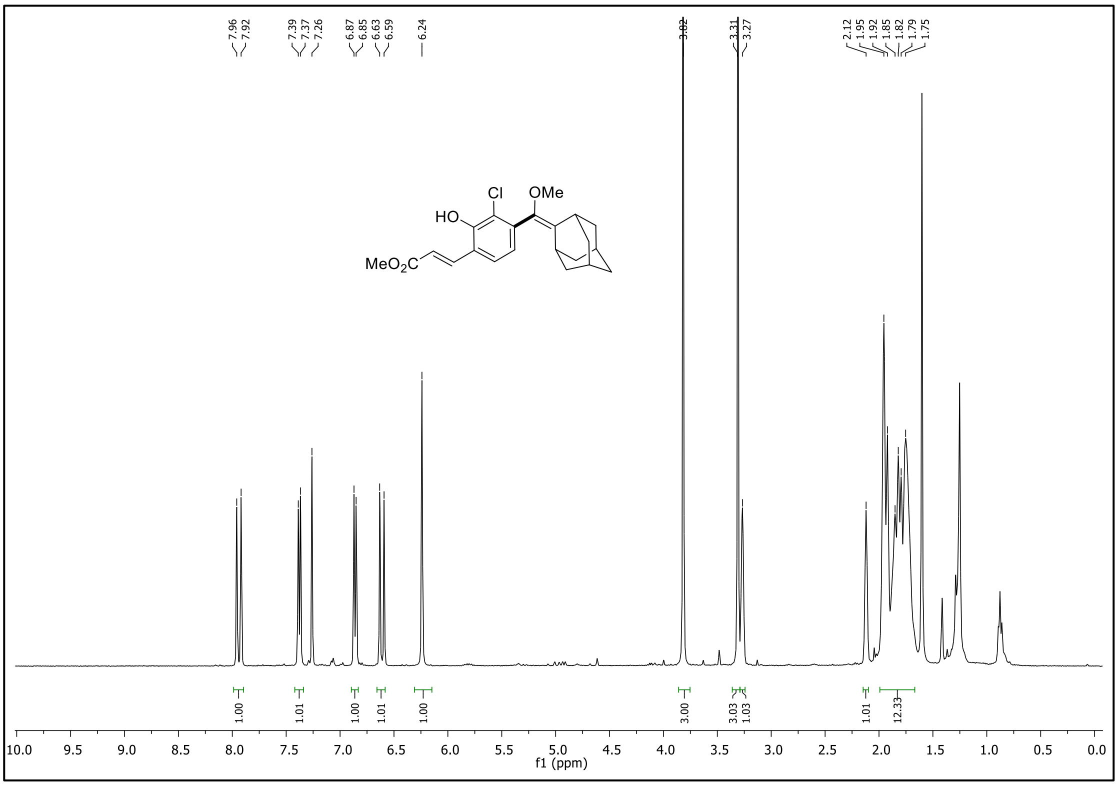

^{1}H-NMR and ^{13}C-NMR spectra of compound **6f:**

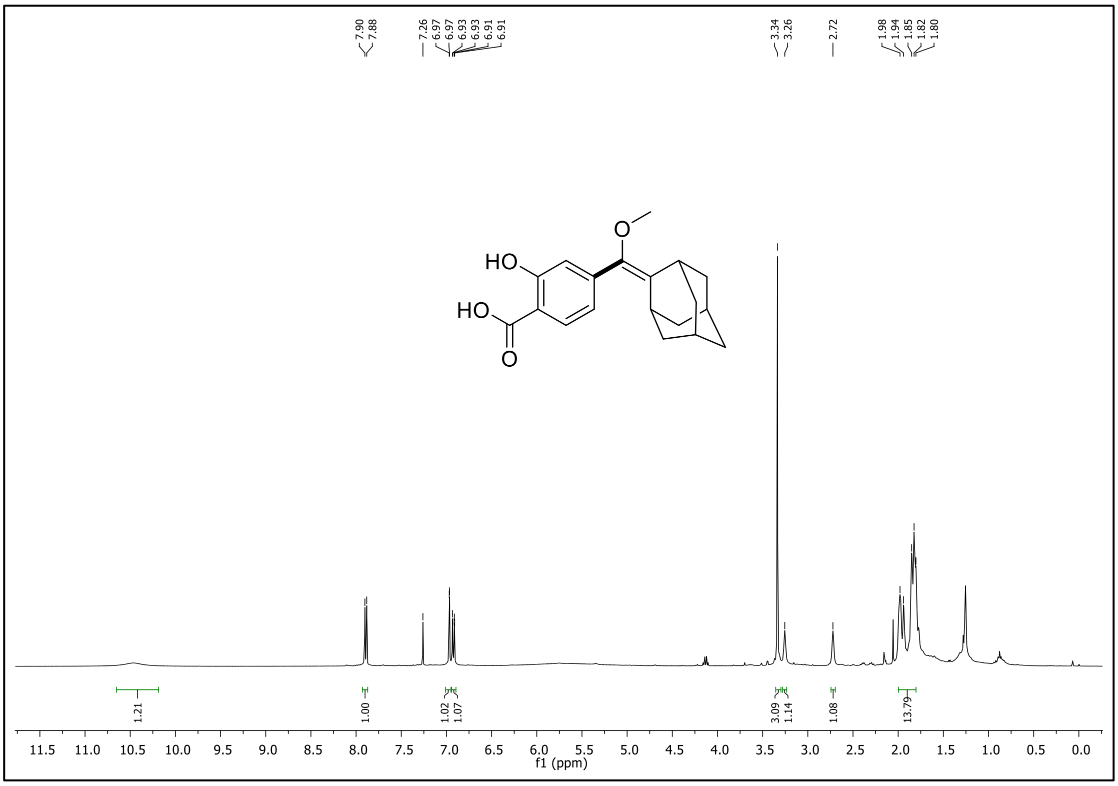

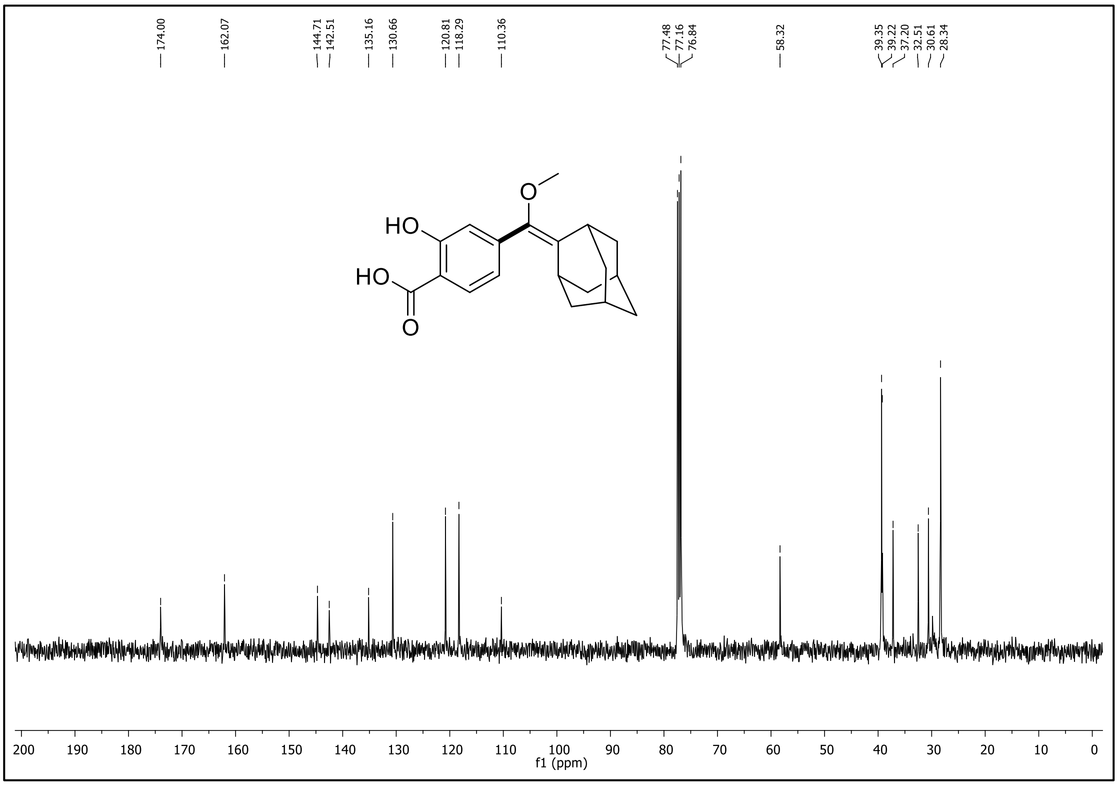

MS of compound **6f:**

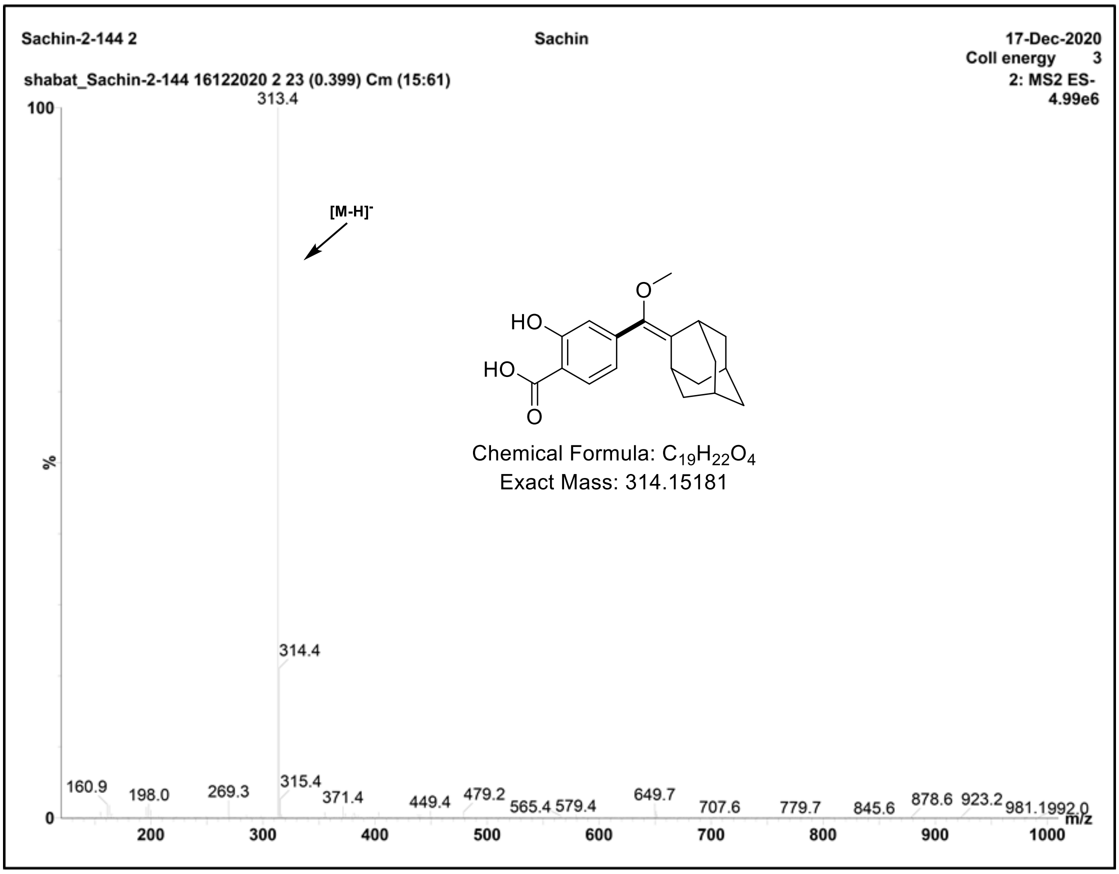

^{1}H-NMR and ^{13}C-NMR spectra of compound **6g:**

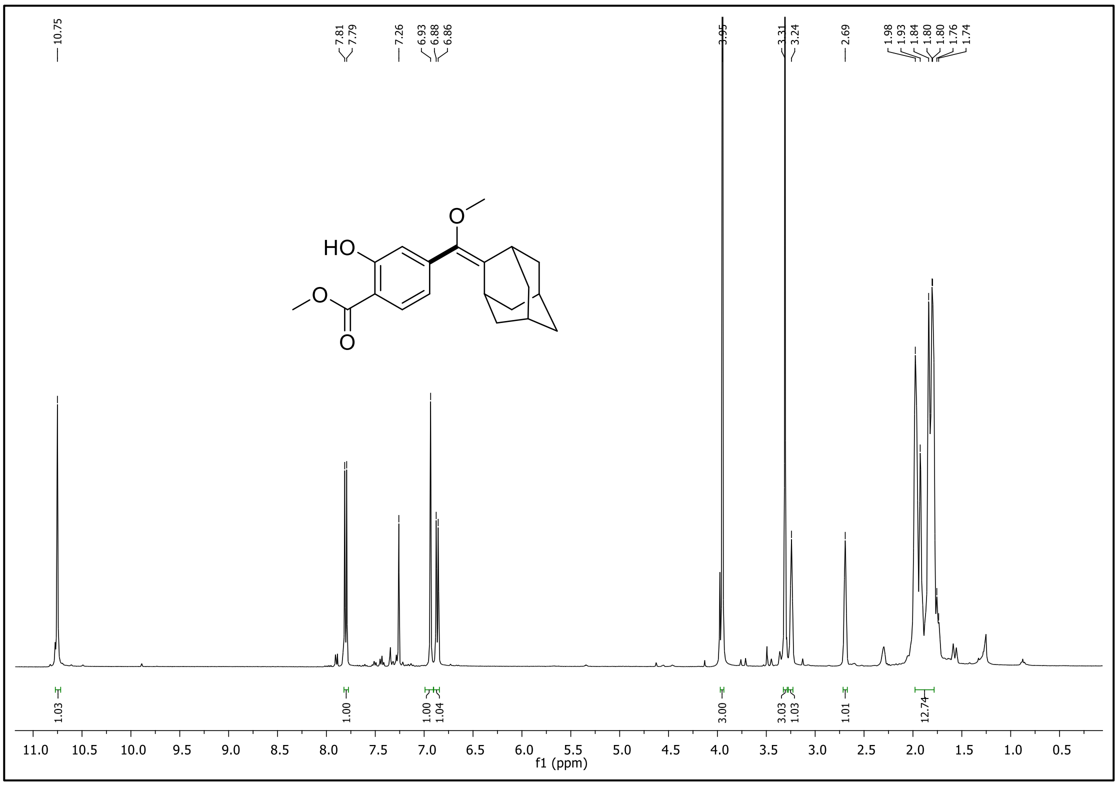

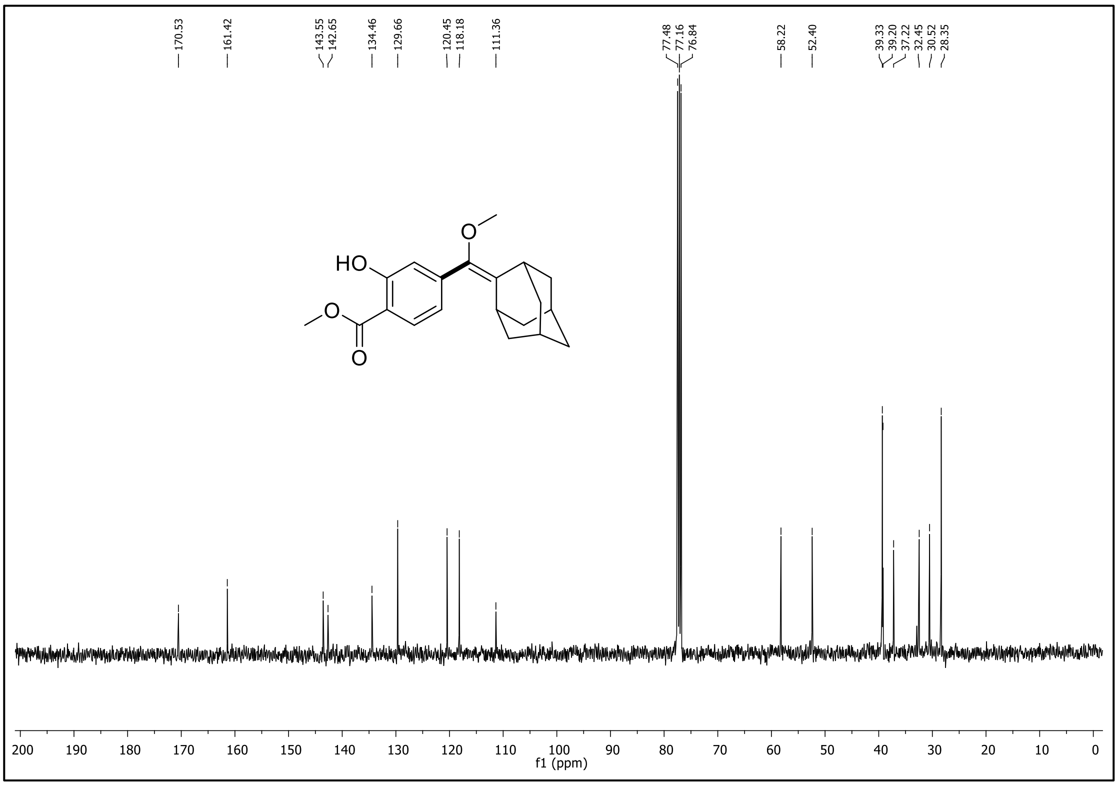

MS of compound **6g:**

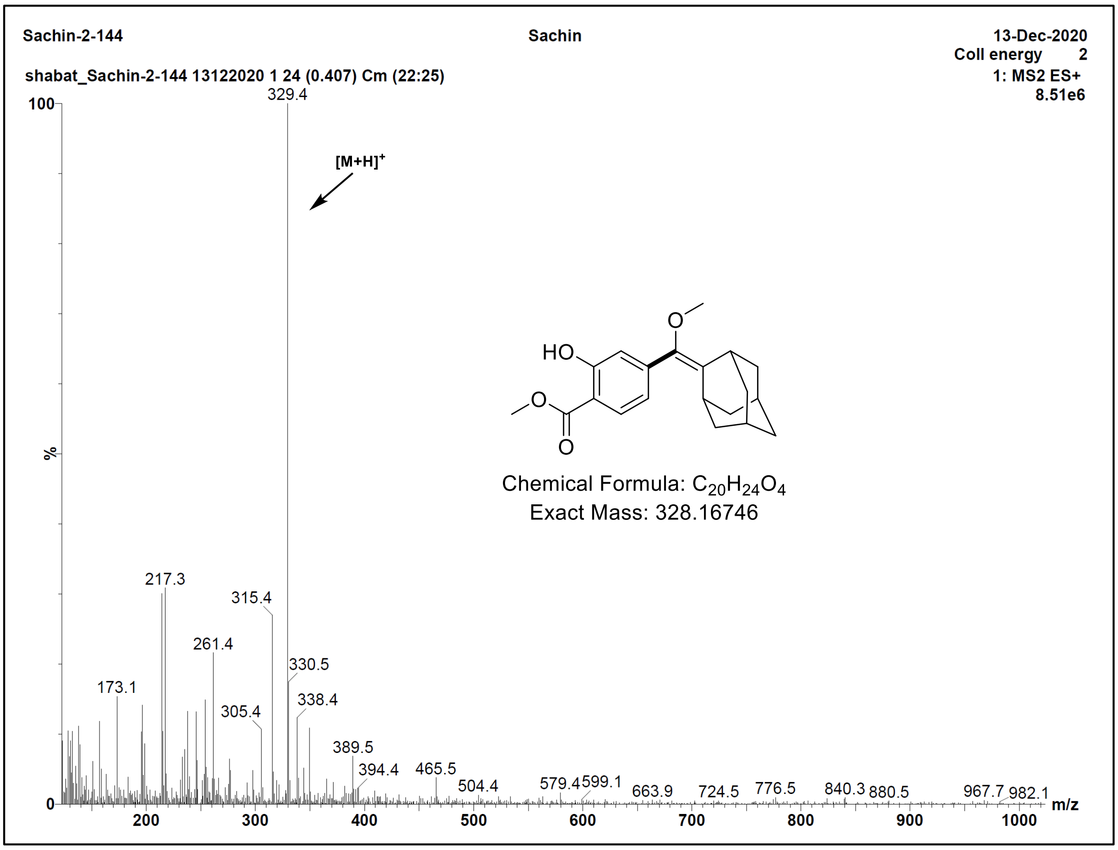

^{1}H-NMR and ^{13}C-NMR spectra of compound **6h:**

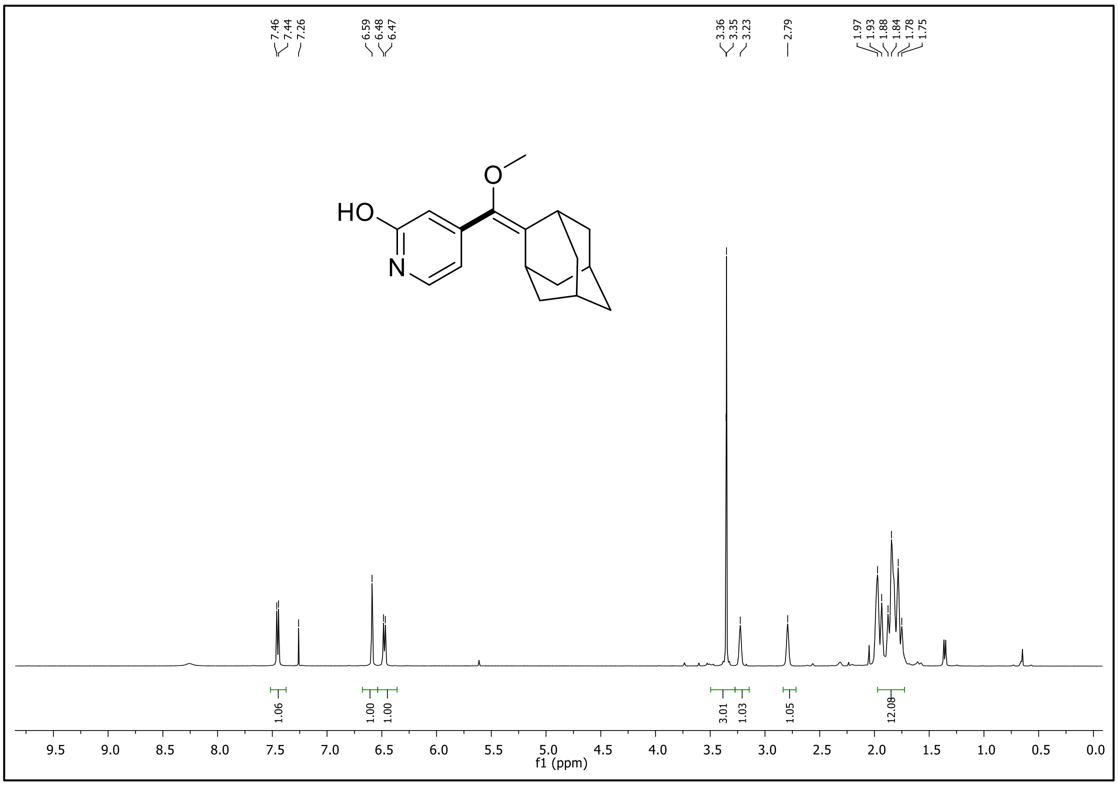

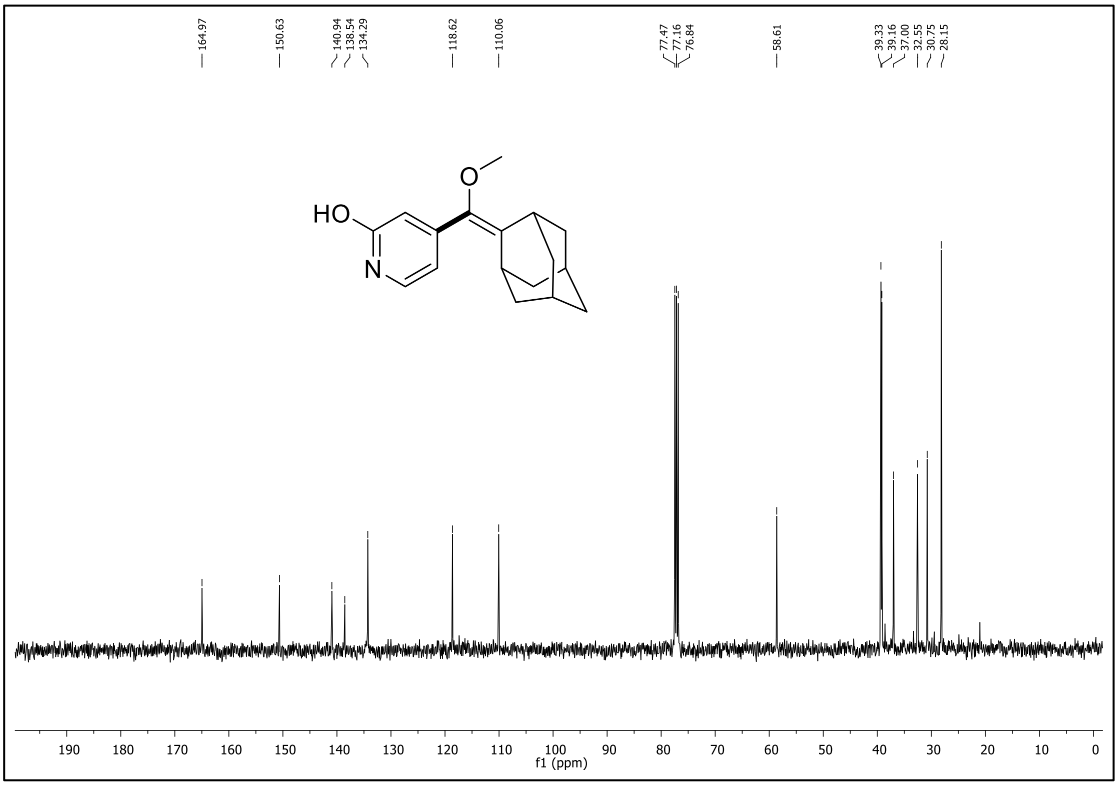

MS of compound **6h:**

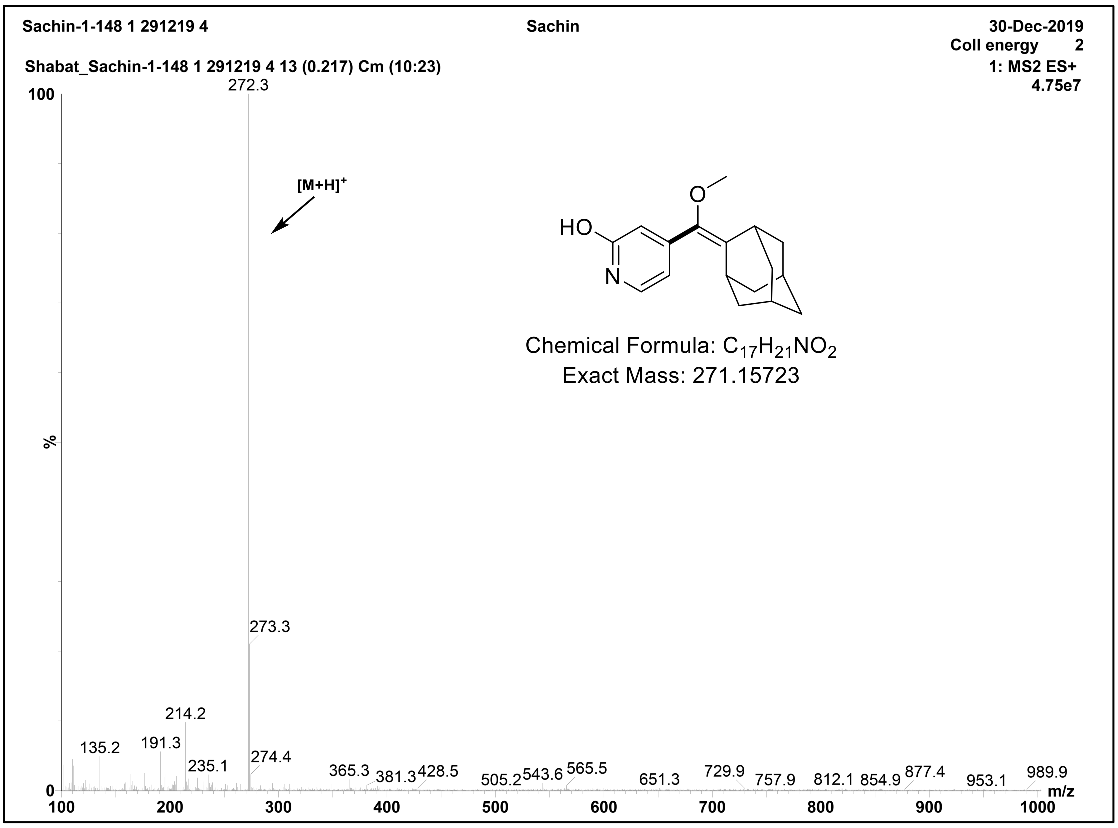

^{1}H-NMR and ^{13}C-NMR spectra of compound **6i:**

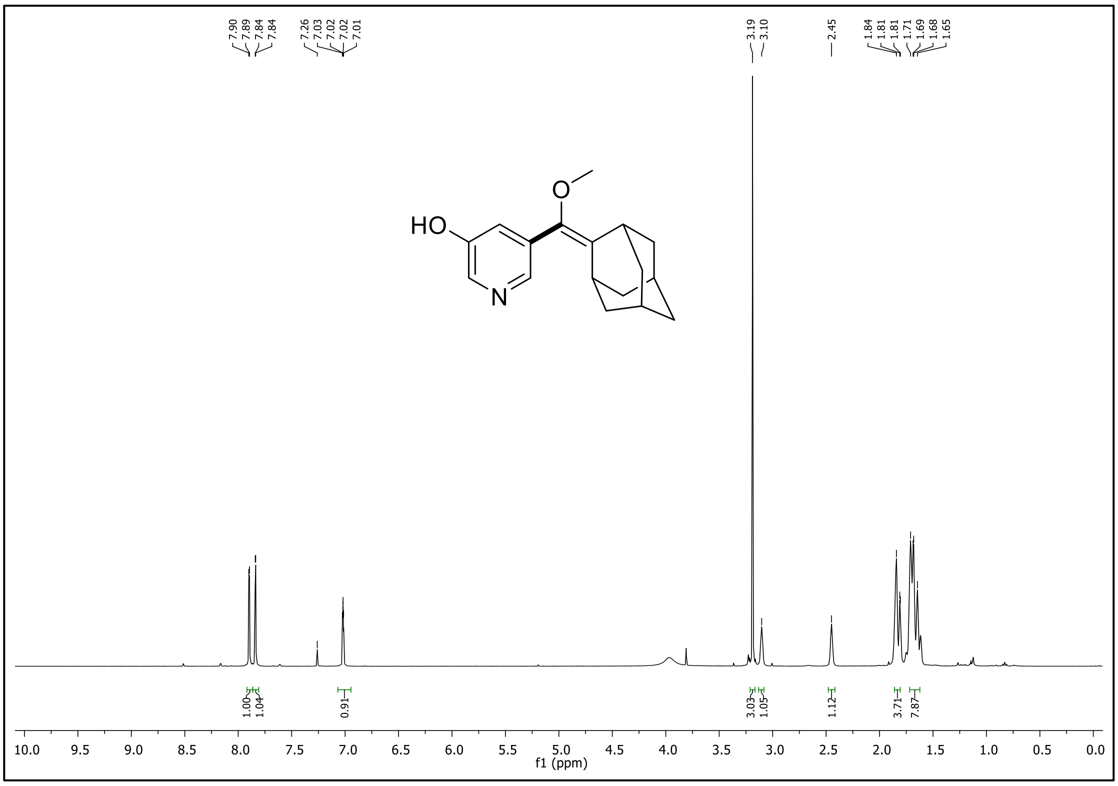

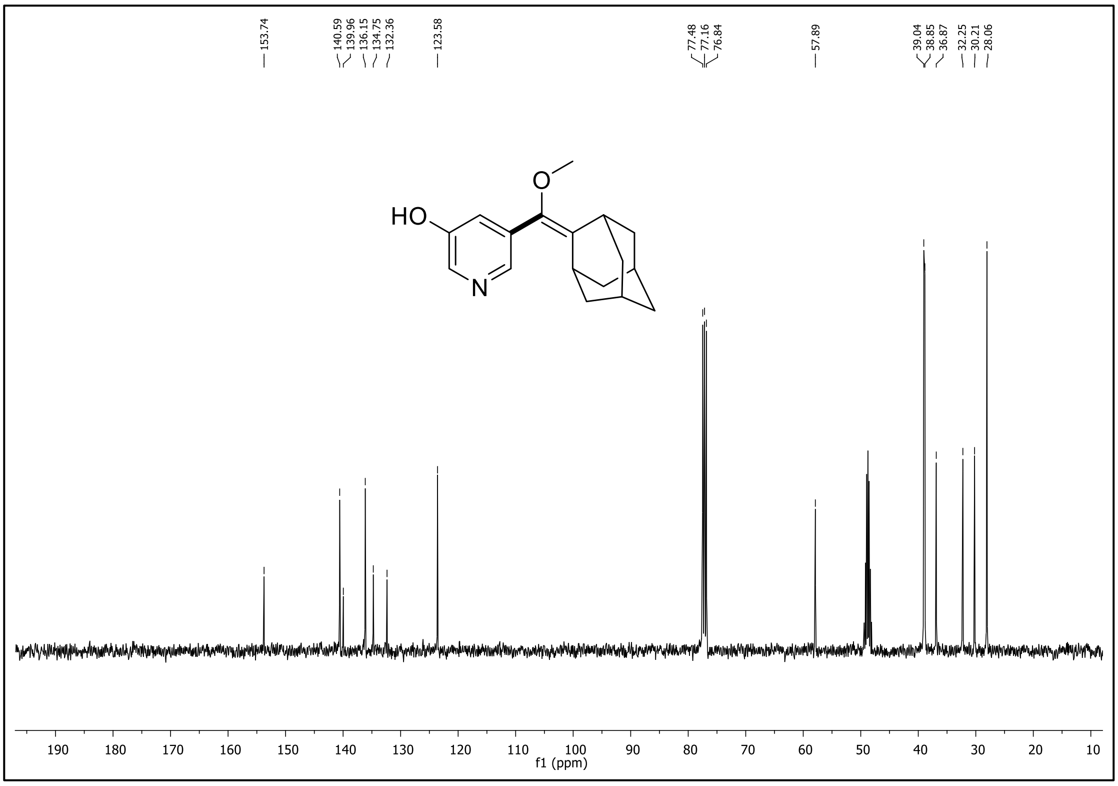

MS of compound **6i:**

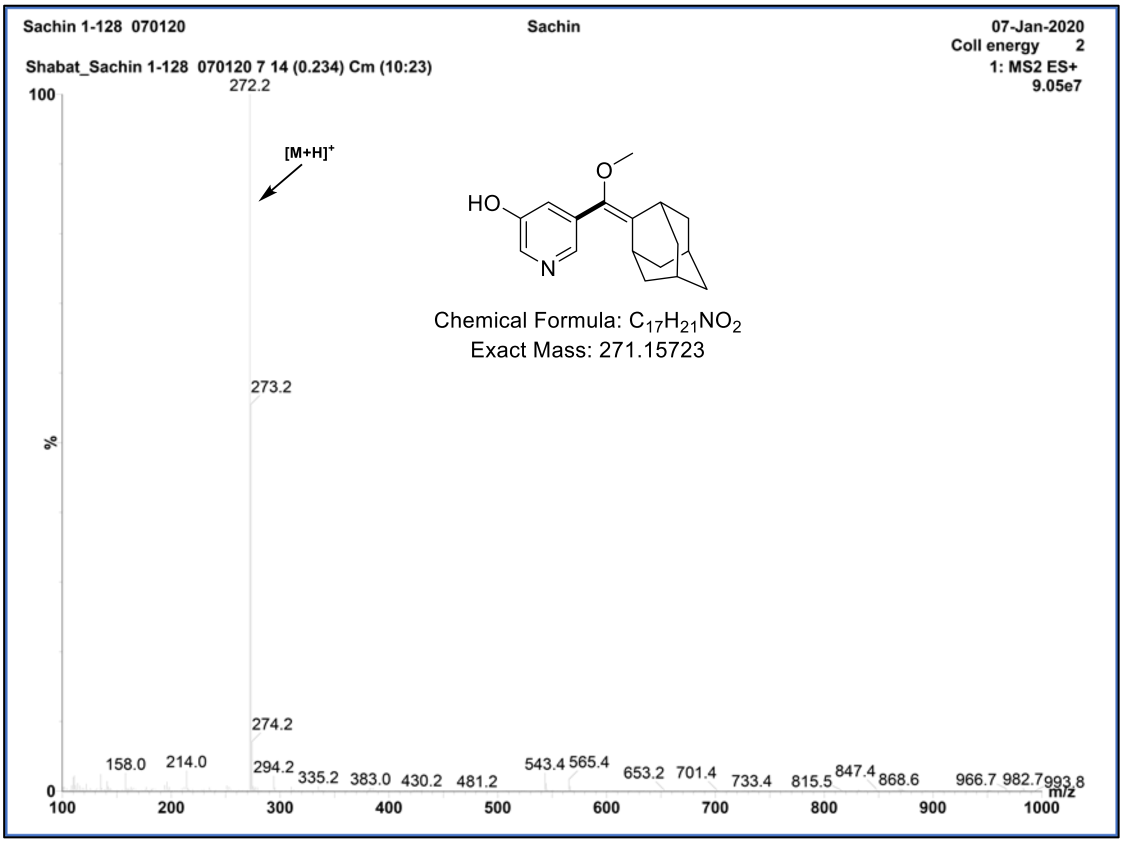

^{1}H-NMR and ^{13}C-NMR spectra of compound **6j:**

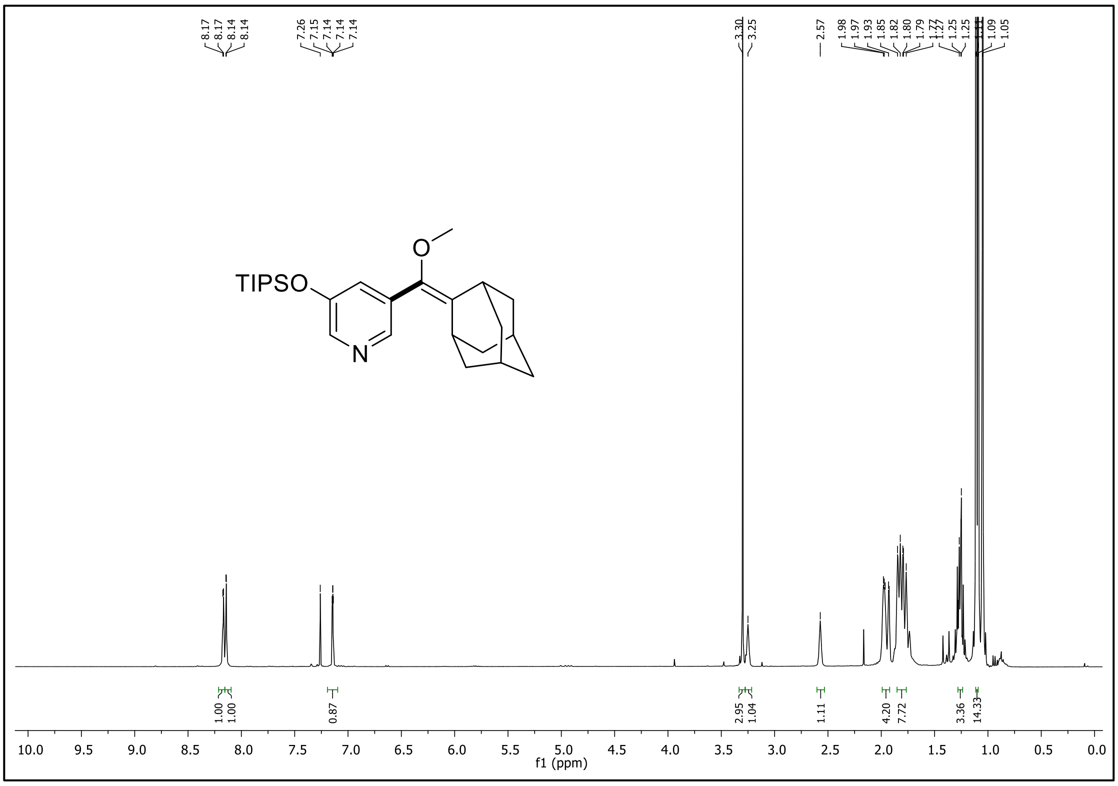

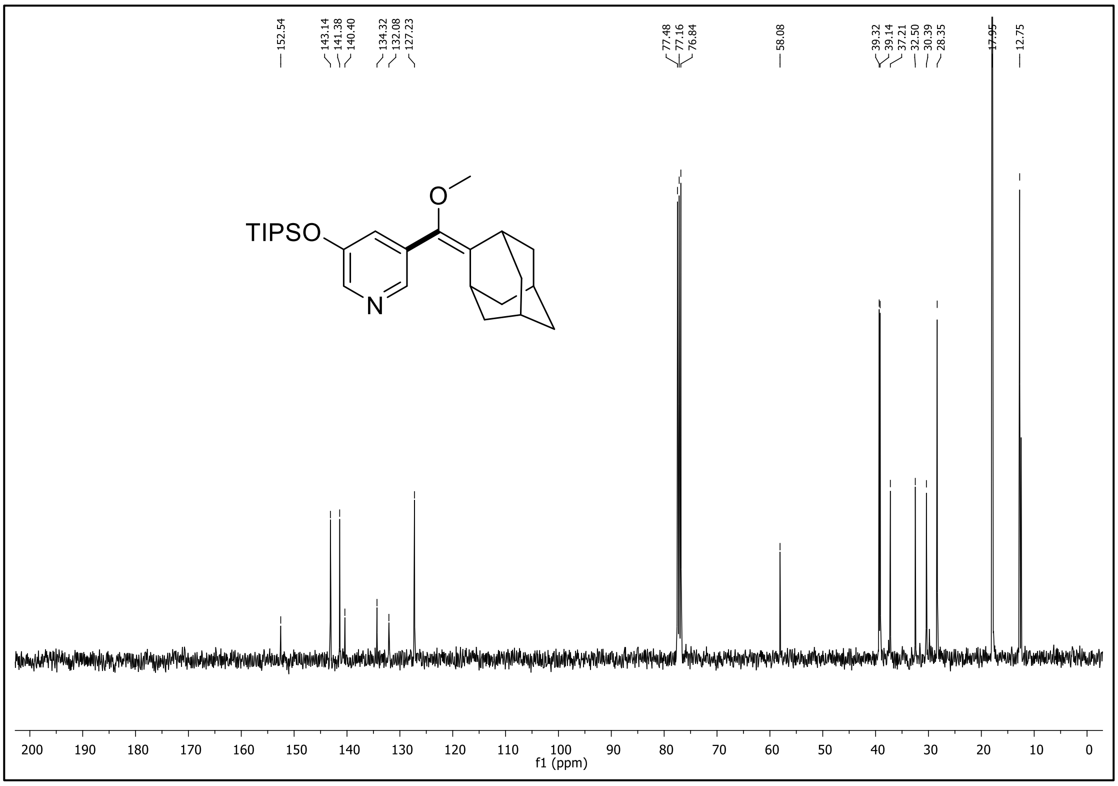

MS of compound **6j:**

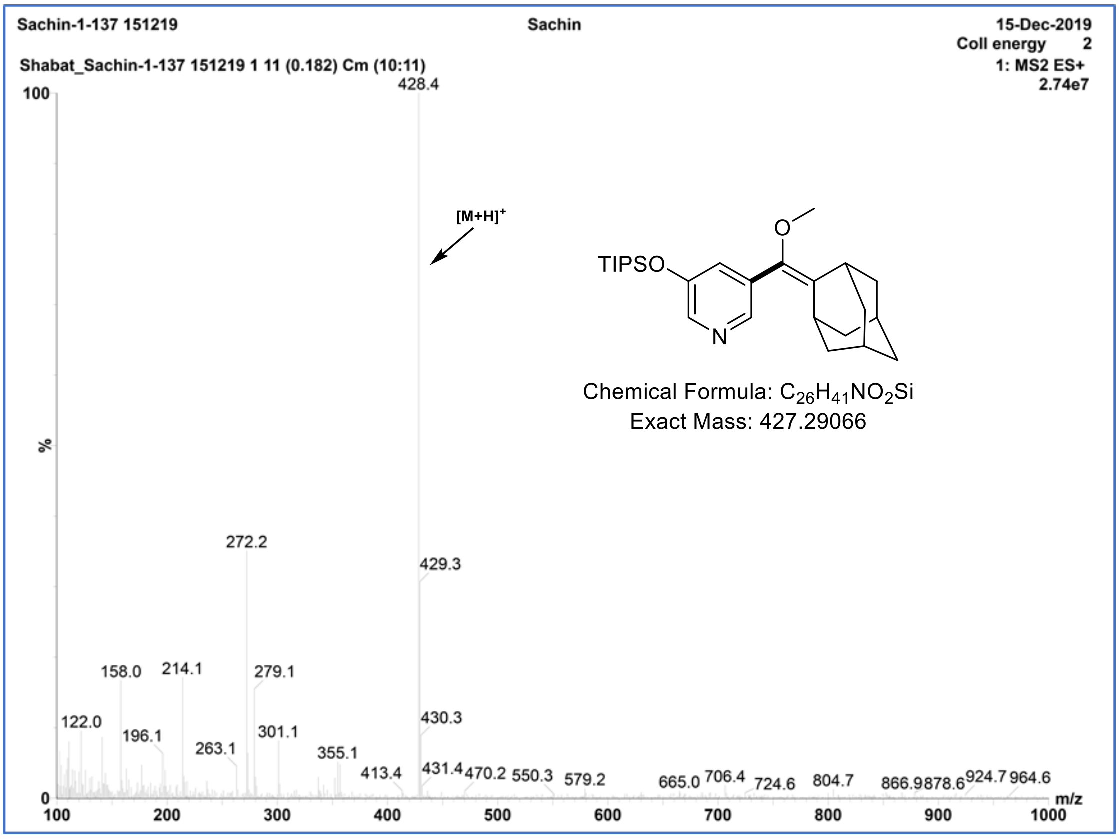

^{1}H-NMR and ^{13}C-NMR spectra of compound **6k:**

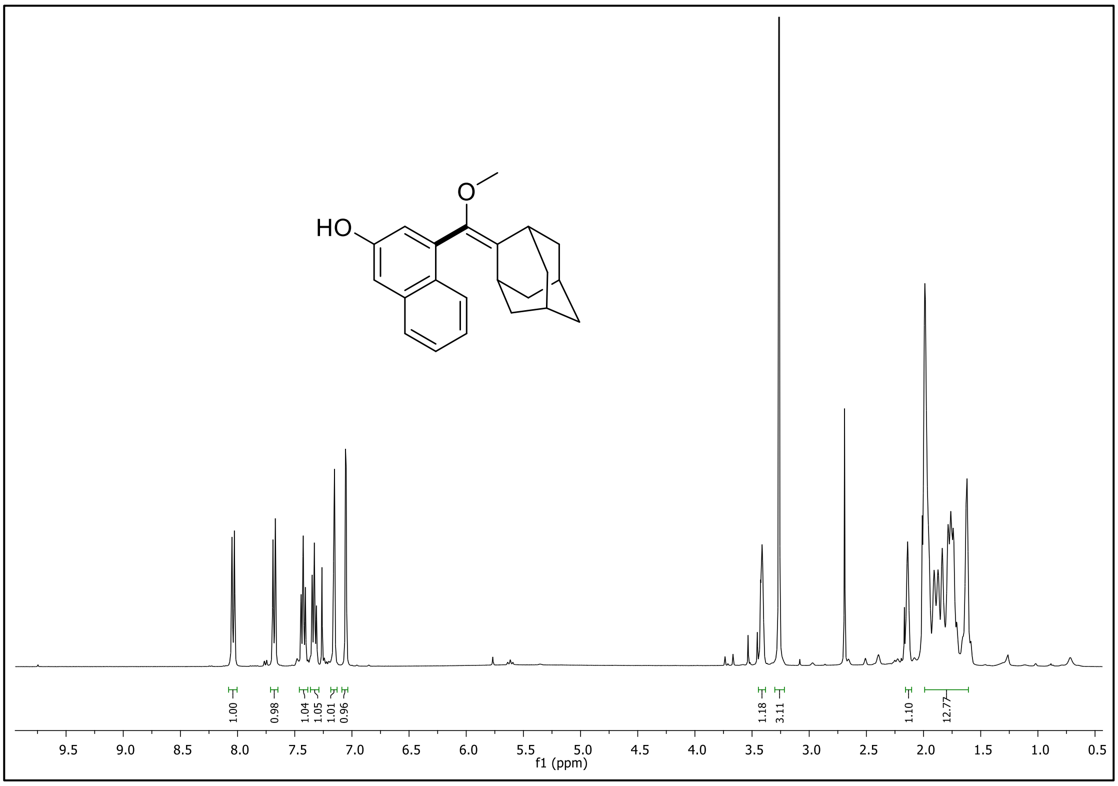

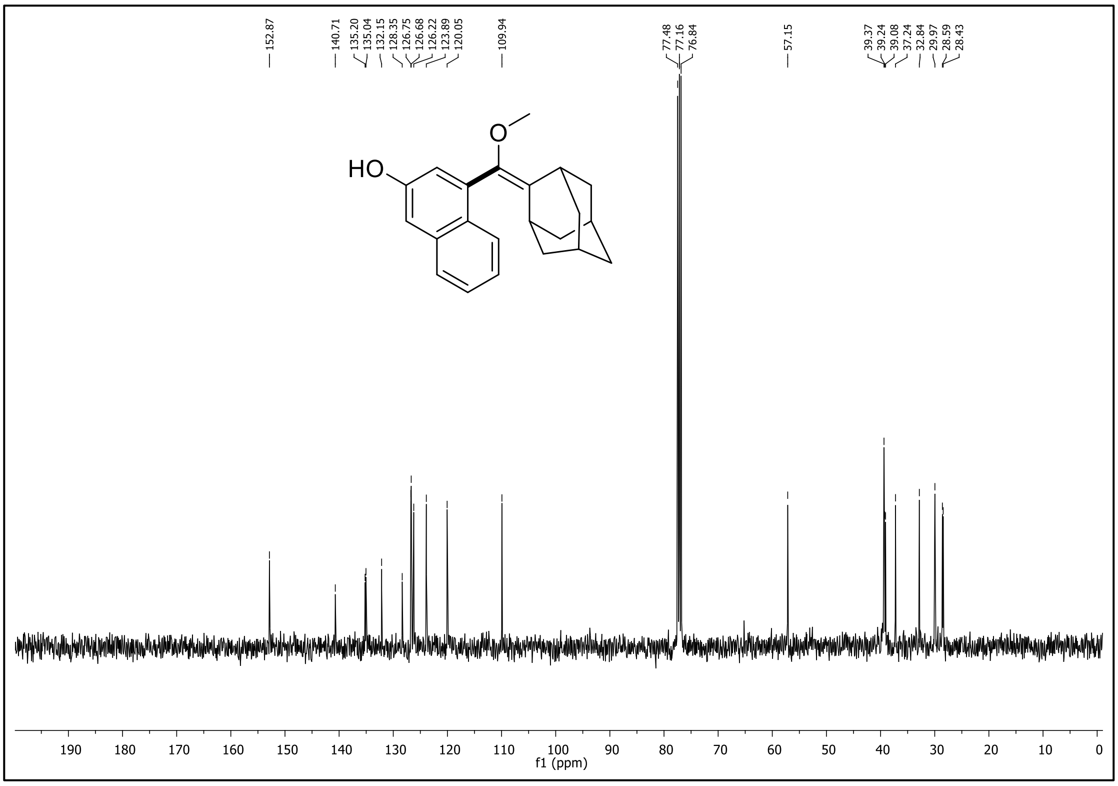

MS of compound **6k:**

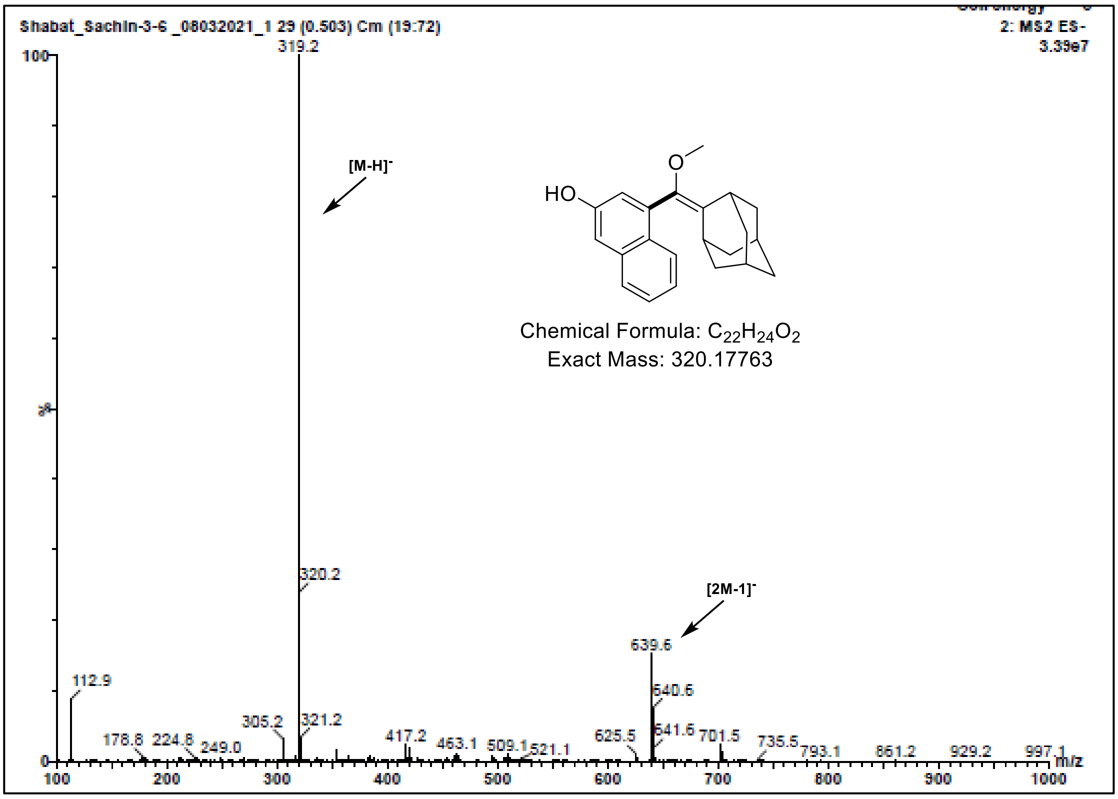

^{1}H-NMR and ^{13}C-NMR spectra of compound **6l:**

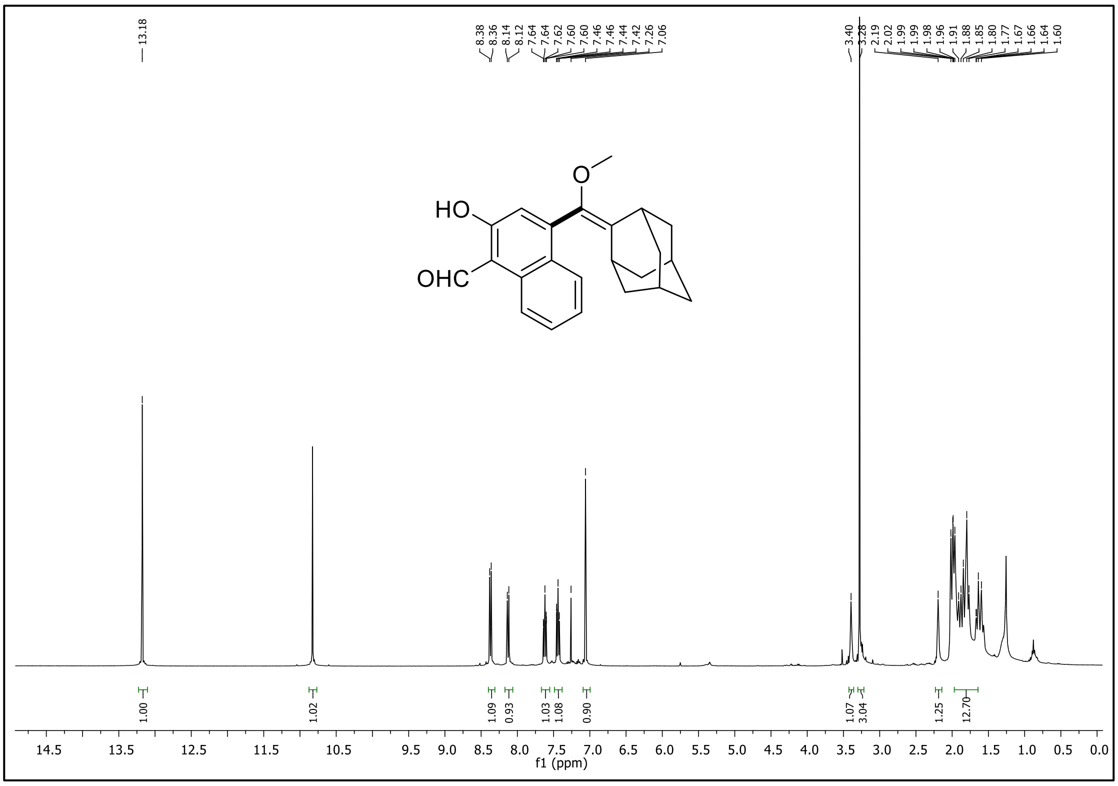

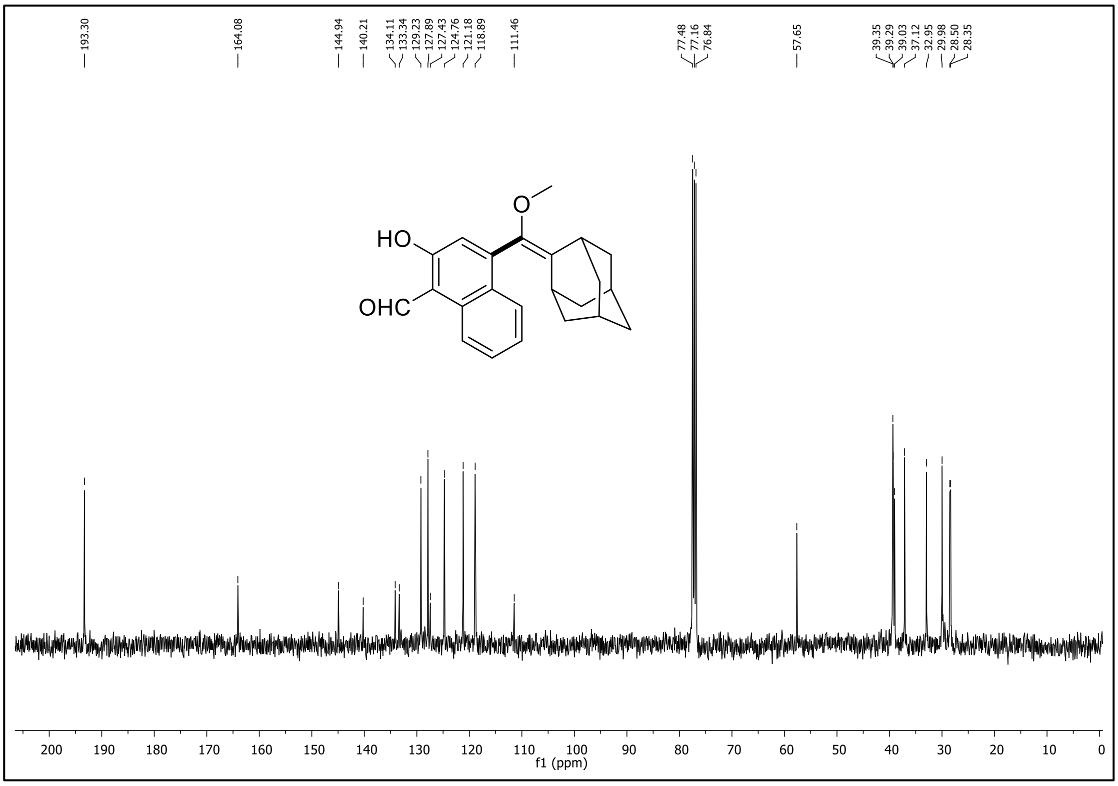

MS of compound **6l:**

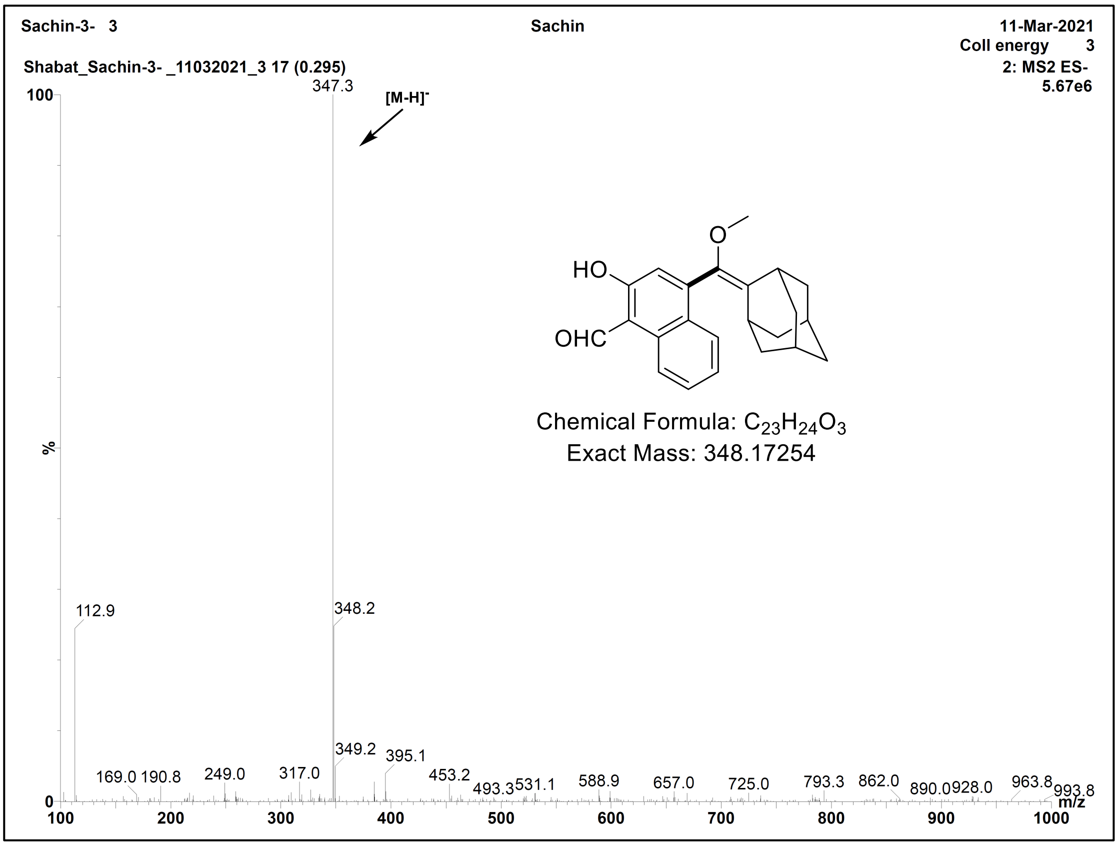

^{1}H-NMR and ^{13}C-NMR spectra of compound **6m:**

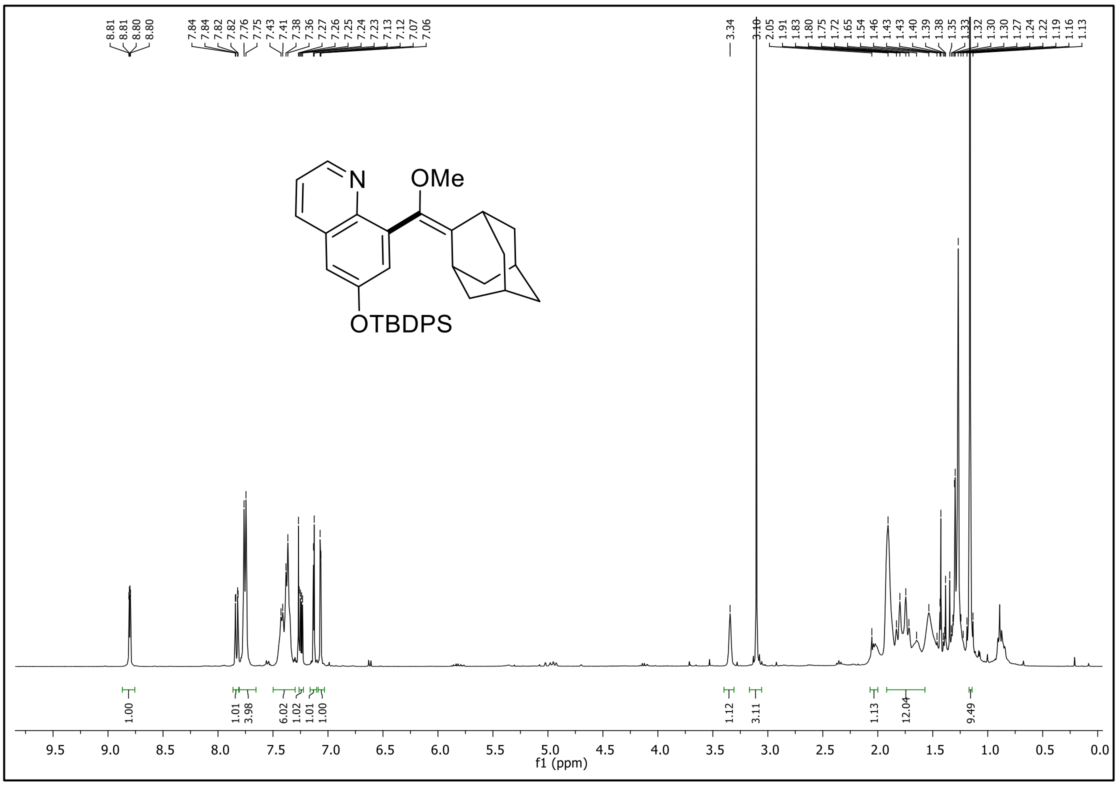

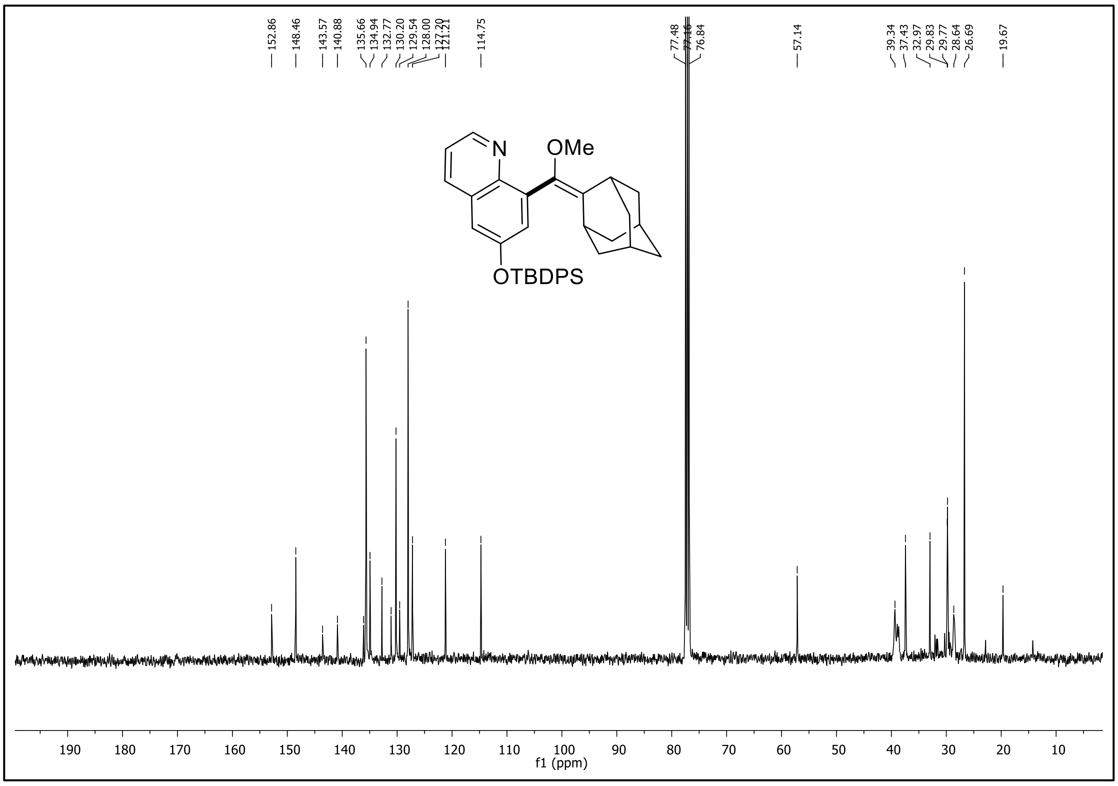

MS of compound **6m:**

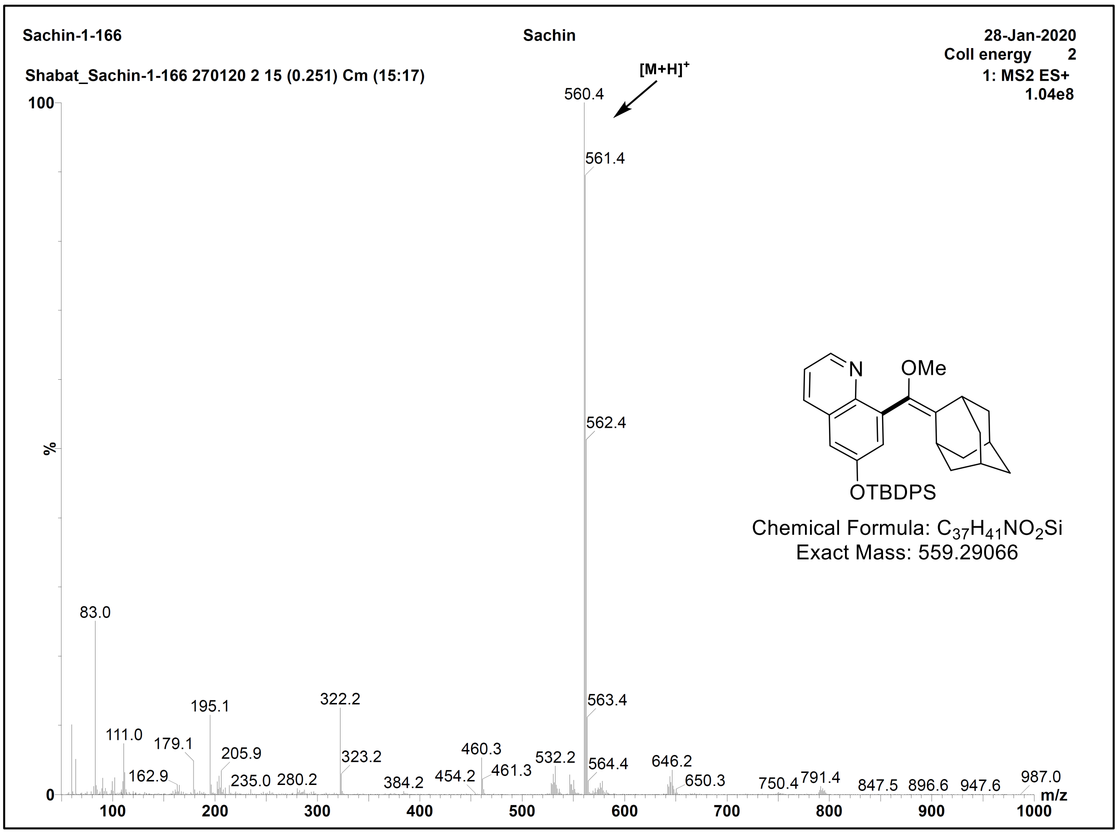

^{1}H-NMR and ^{13}C-NMR spectra of compound **6n:**

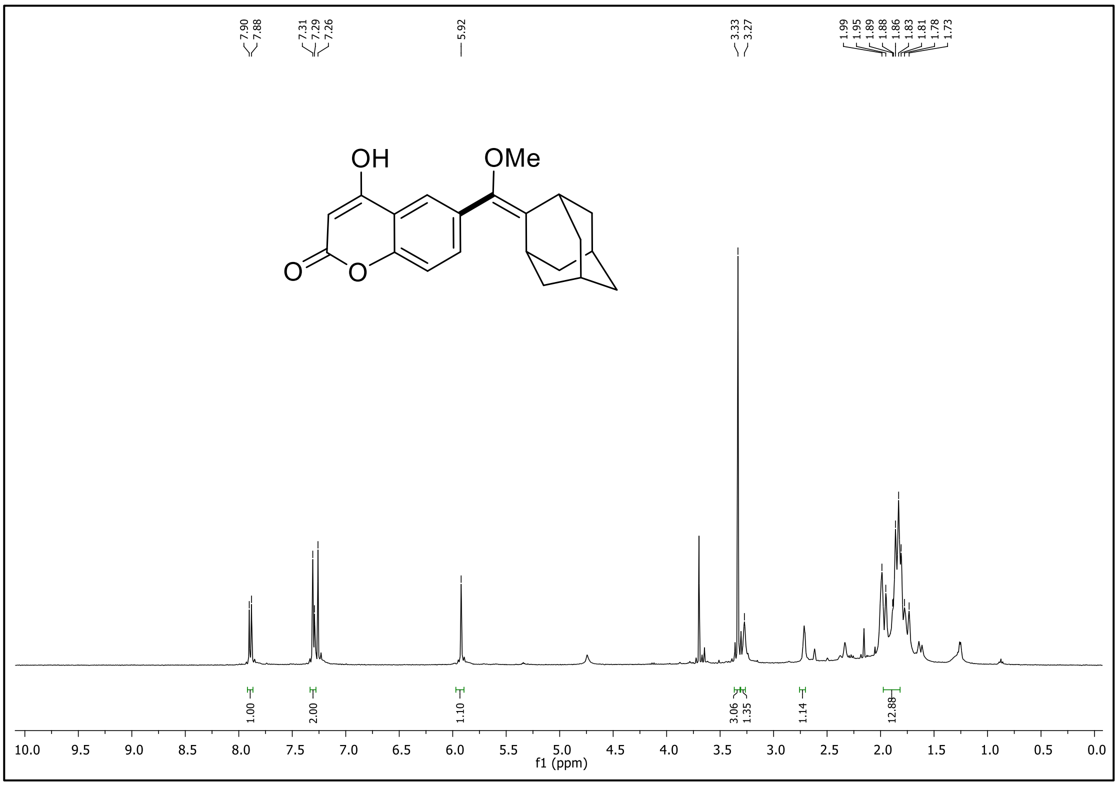

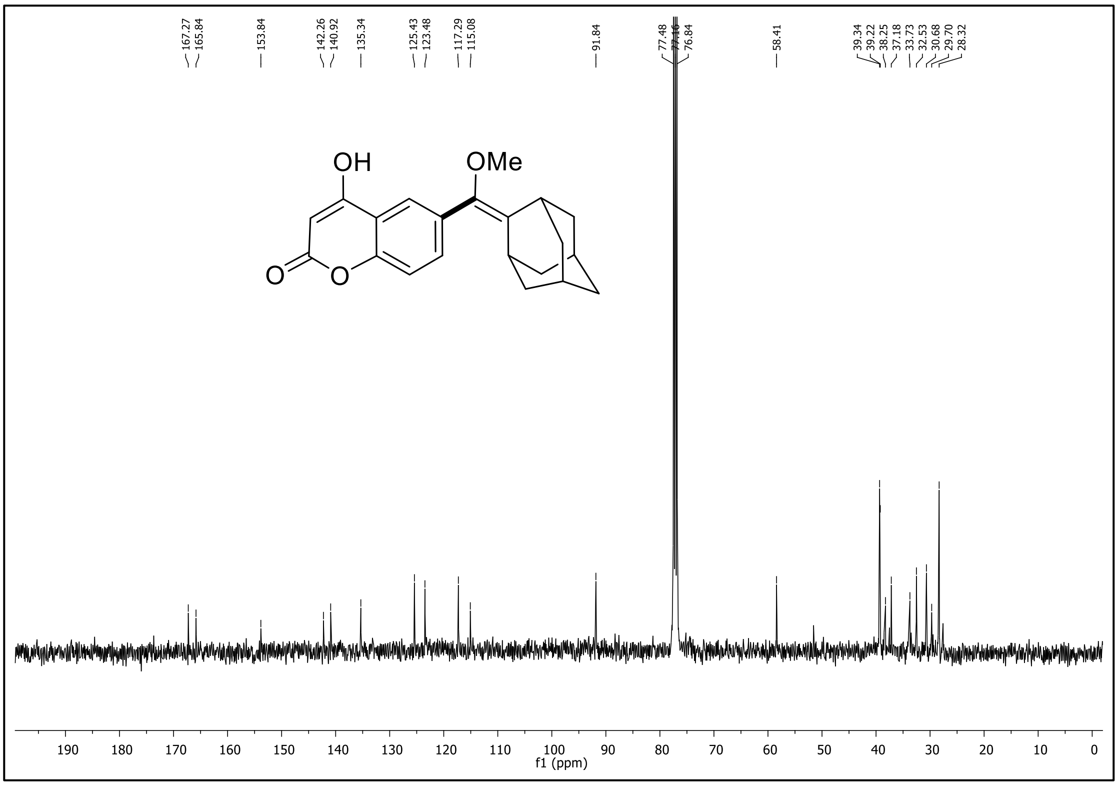

MS of compound **6n:**

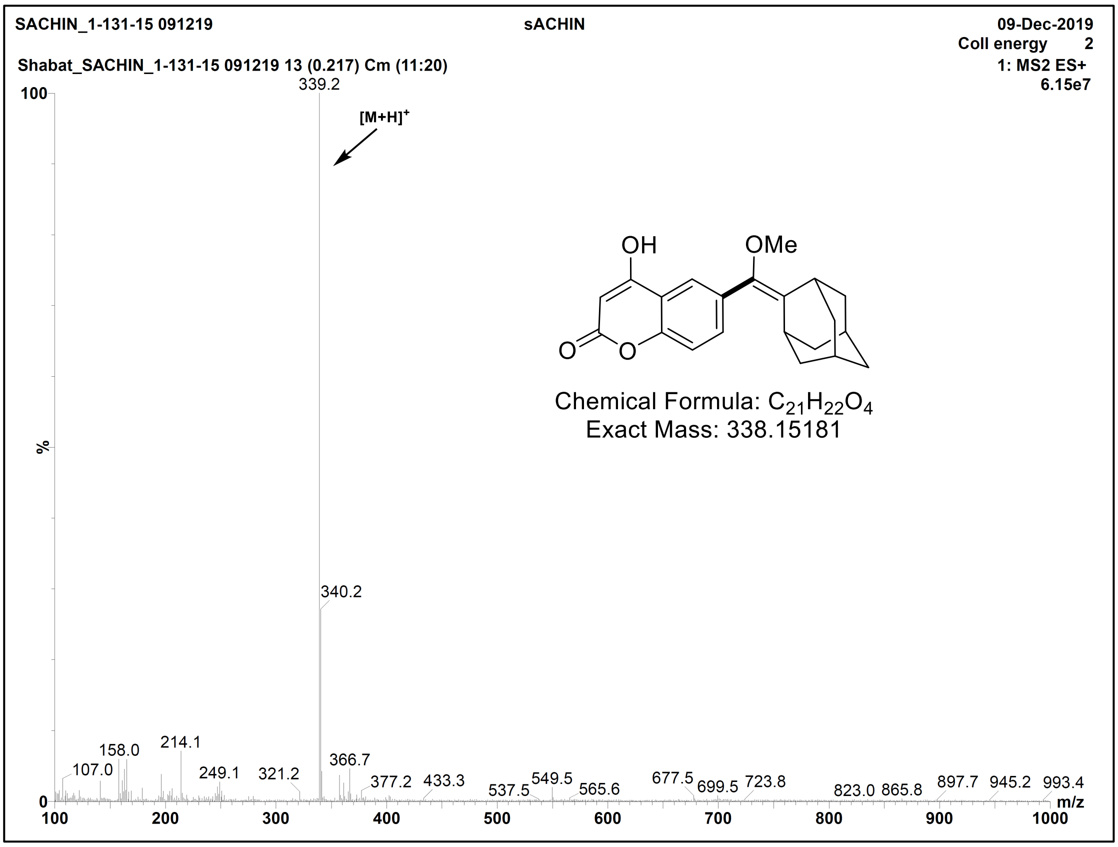

^{1}H-NMR and ^{13}C-NMR spectra of compound **6o:**

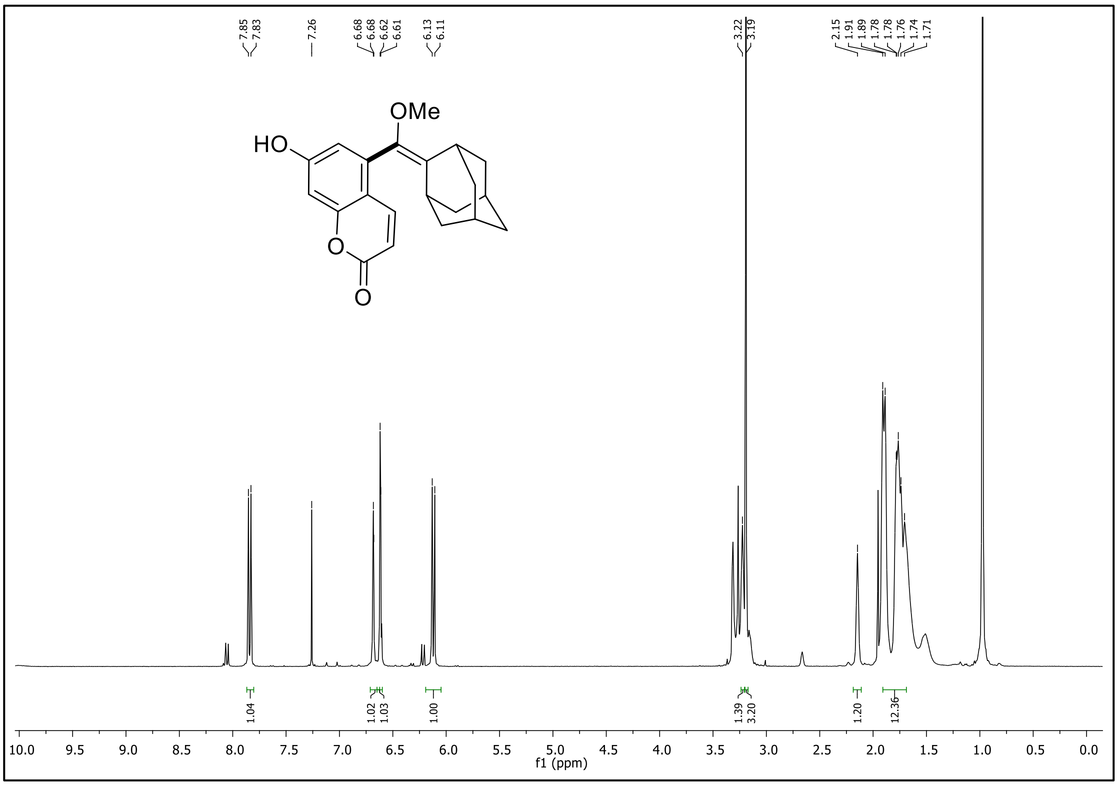

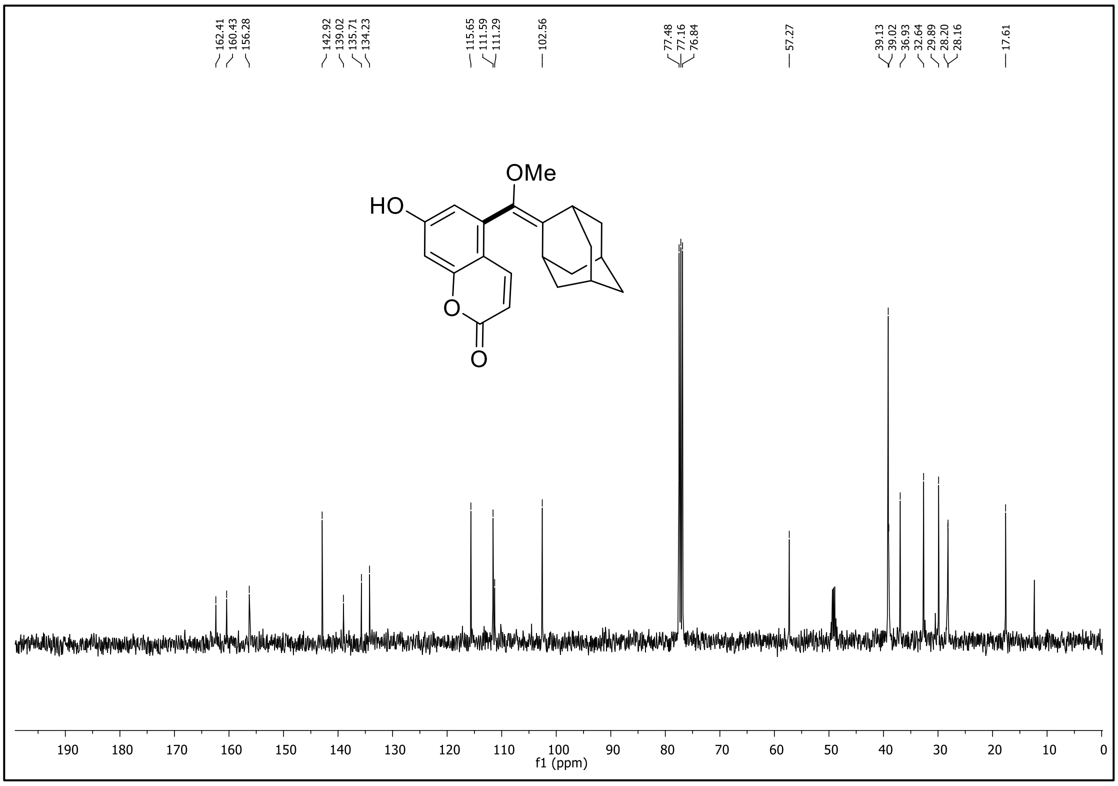

MS of compound **6o:**

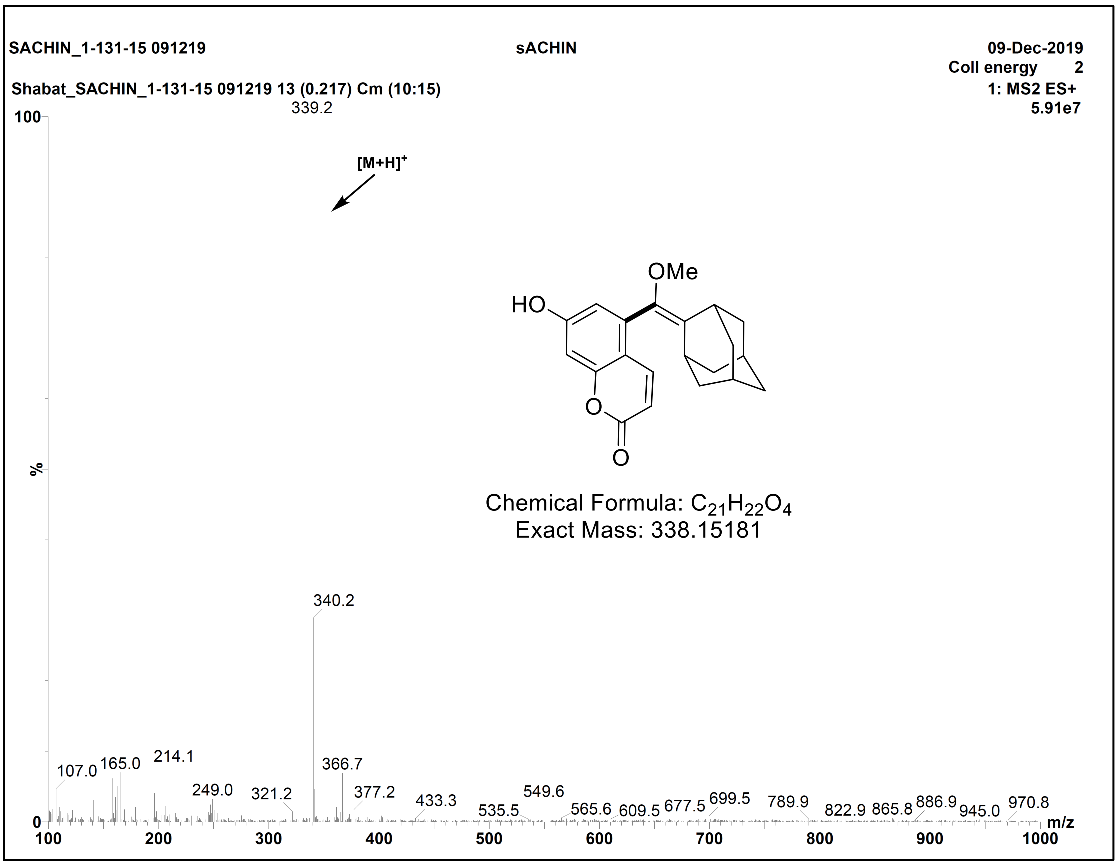

^{1}H-NMR and ^{13}C-NMR spectra of compound **6p:**

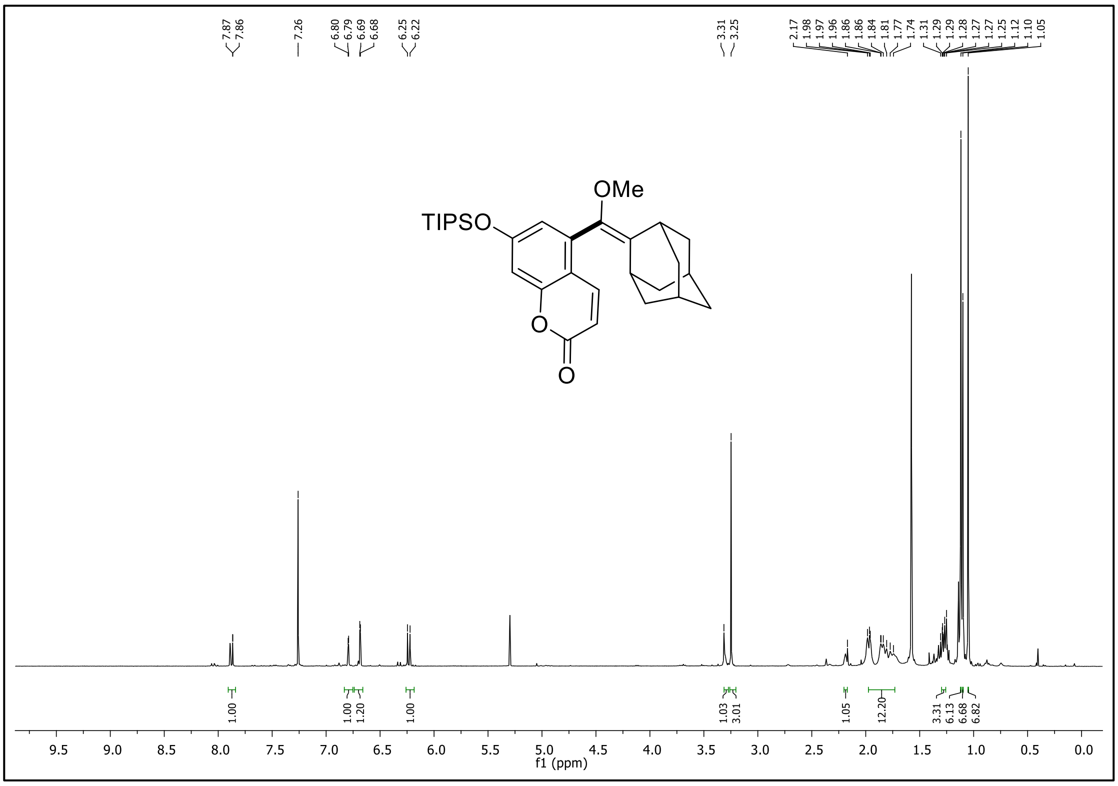

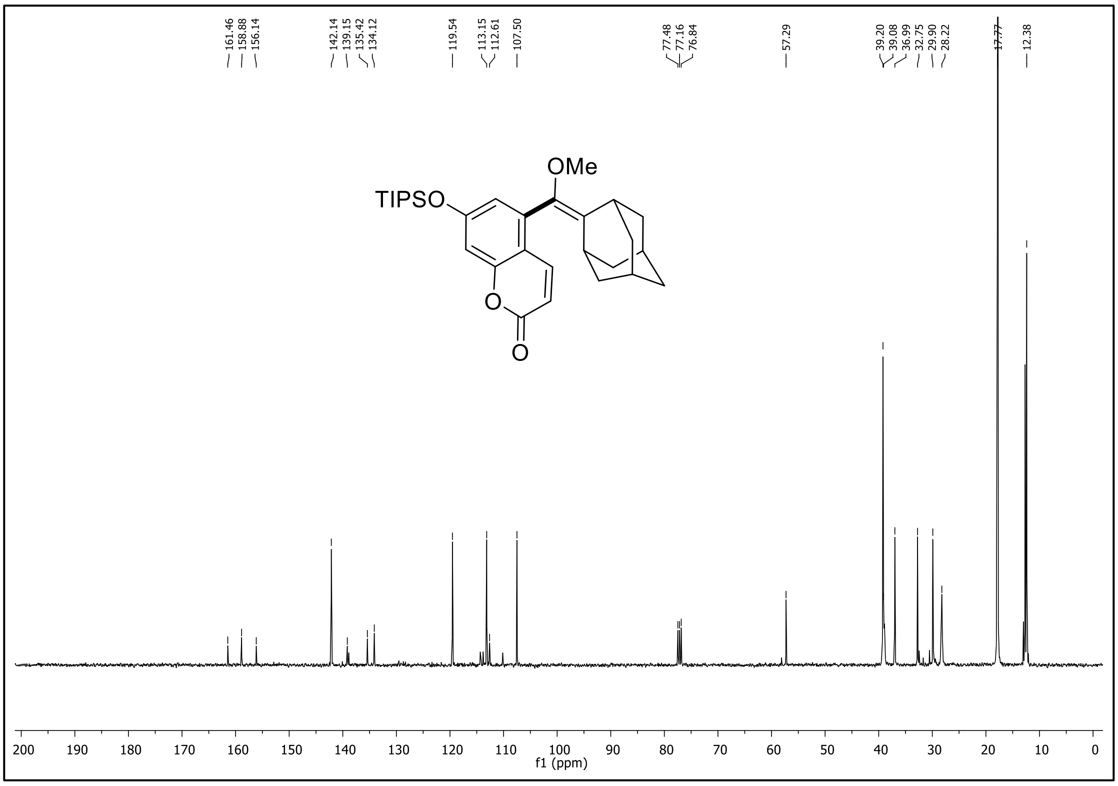

MS of compound **6p:**

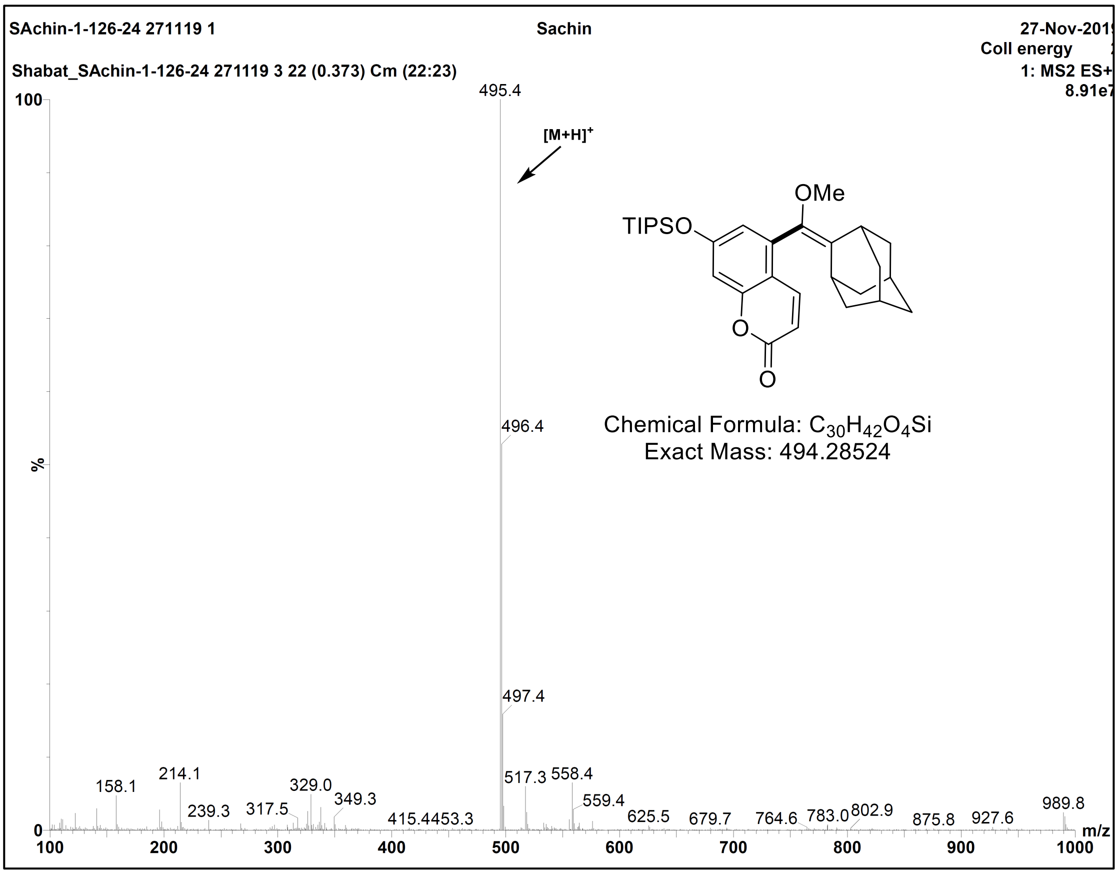

^{1}H-NMR and ^{13}C-NMR spectra of compound **6q:**

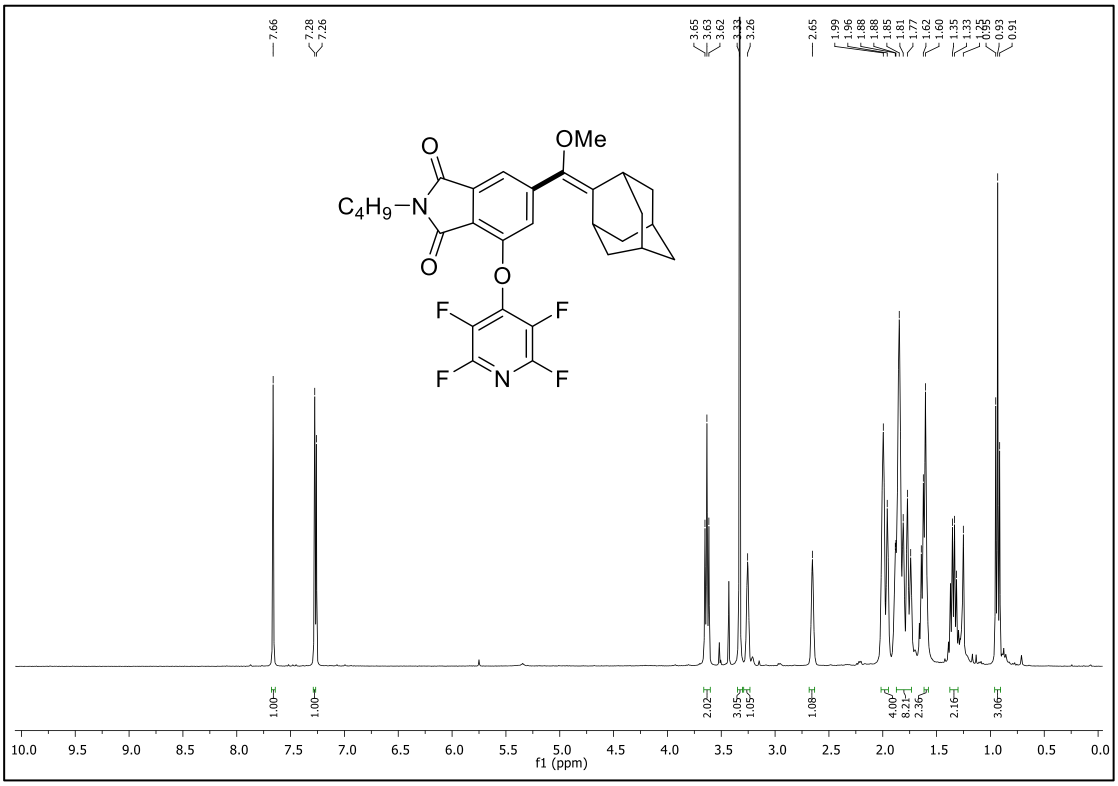

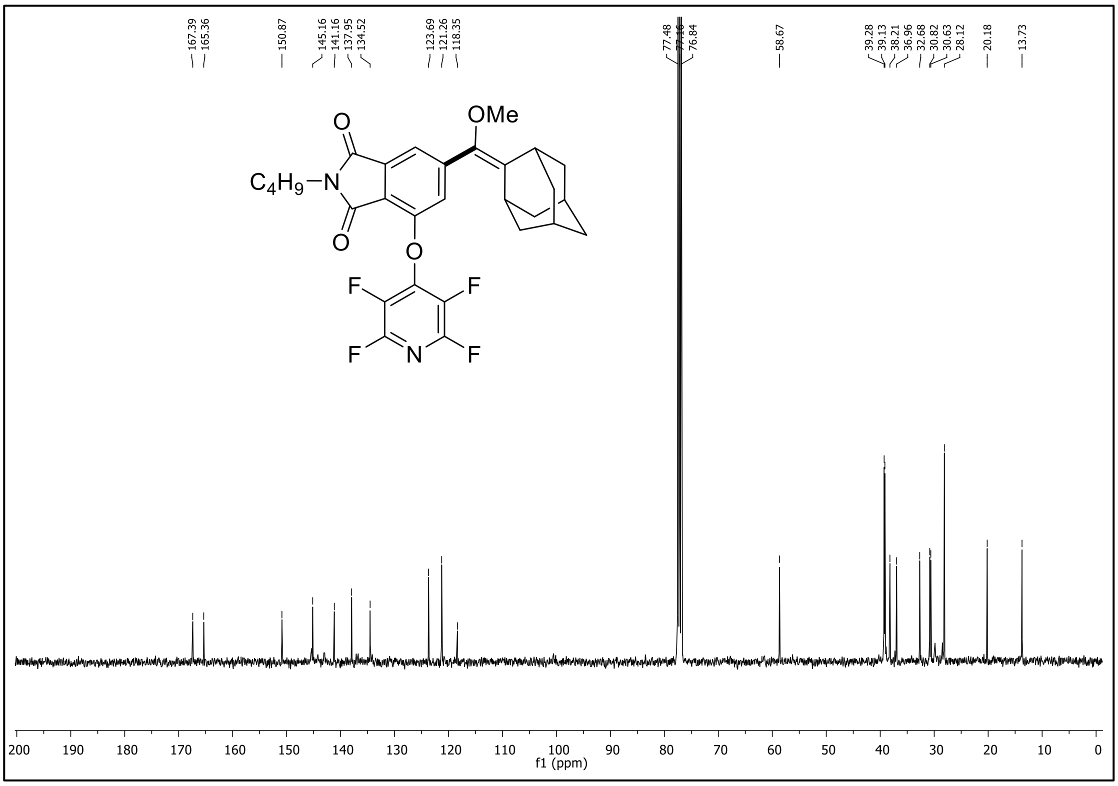

^{19}F-NMR spectra of compound **6q:**

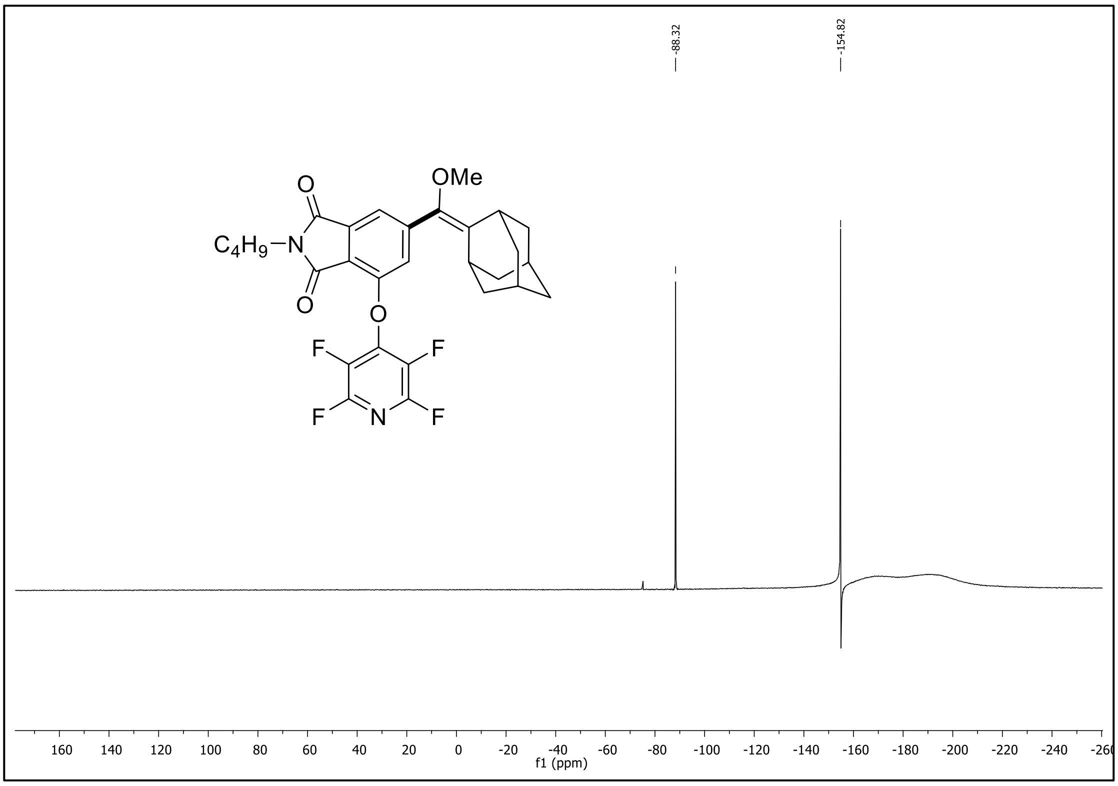

^{1}H-NMR and ^{13}C-NMR spectra of compound **6r:**

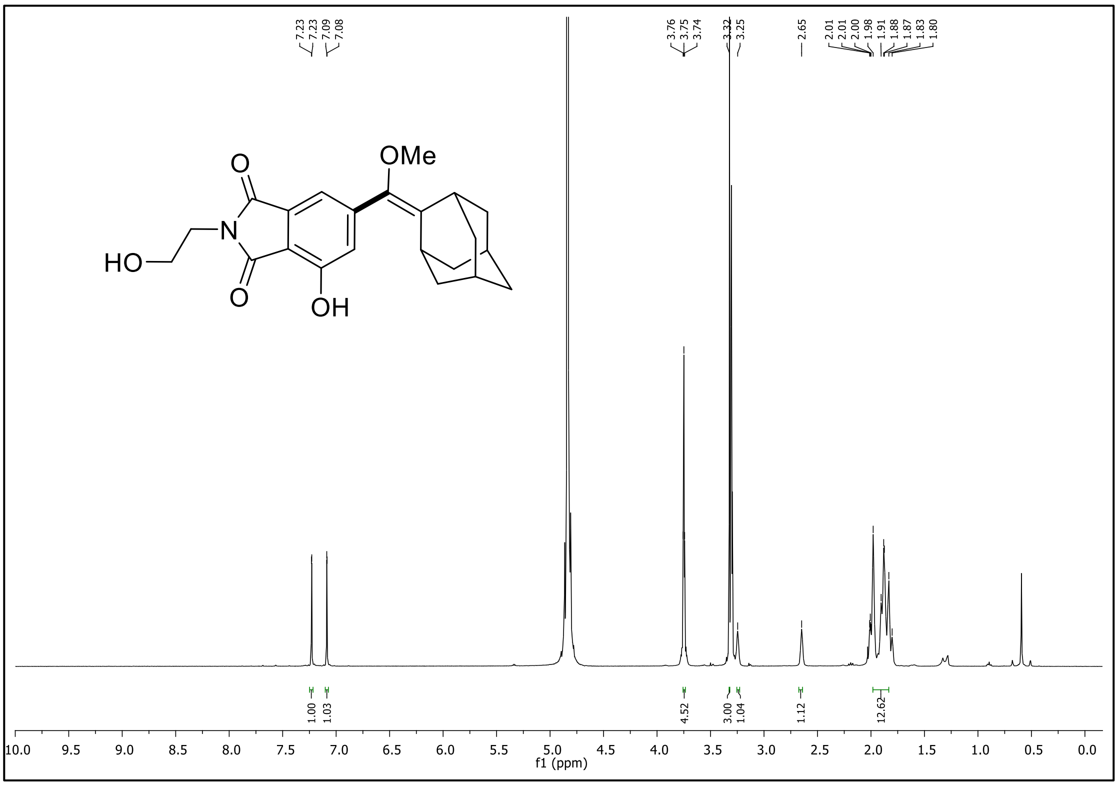

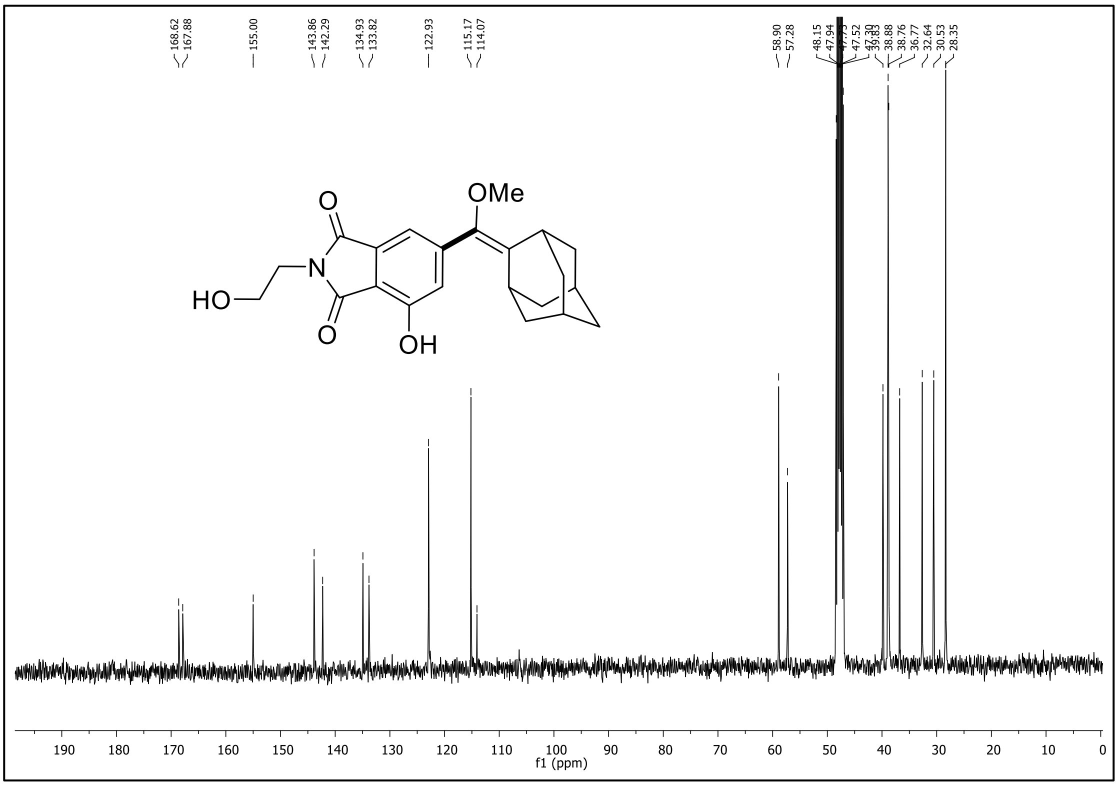

MS of compound **6r:**

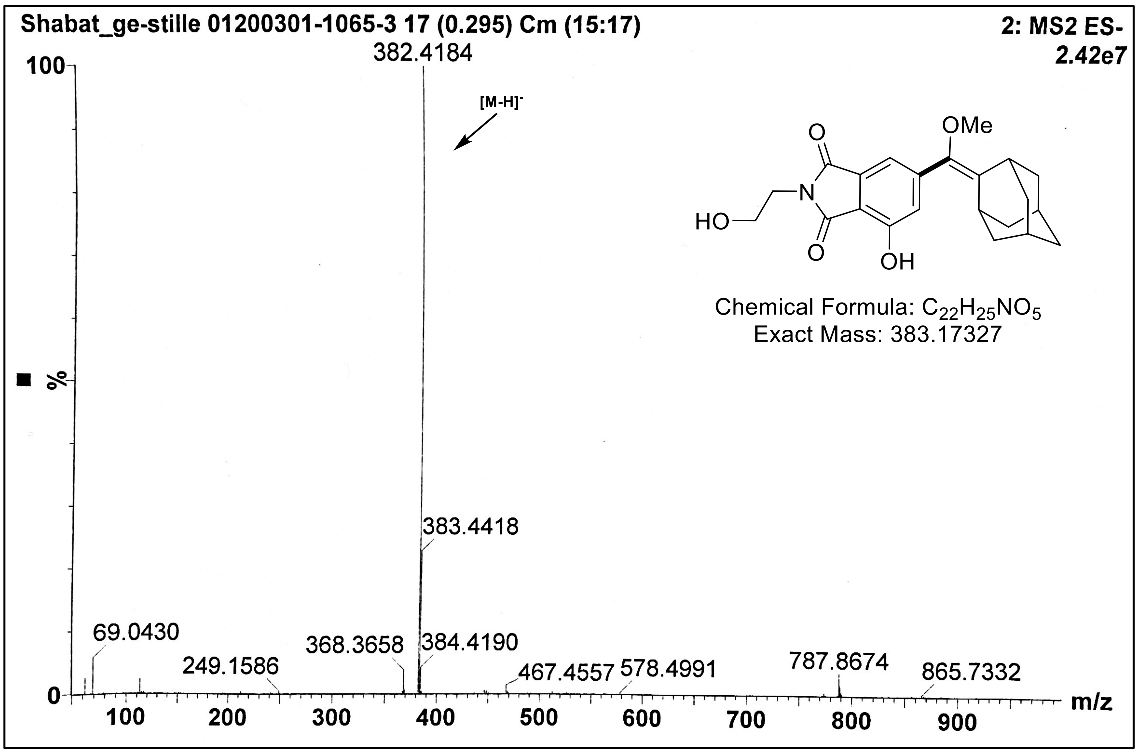

^{1}H-NMR and ^{13}C-NMR spectra of compound **6s:**

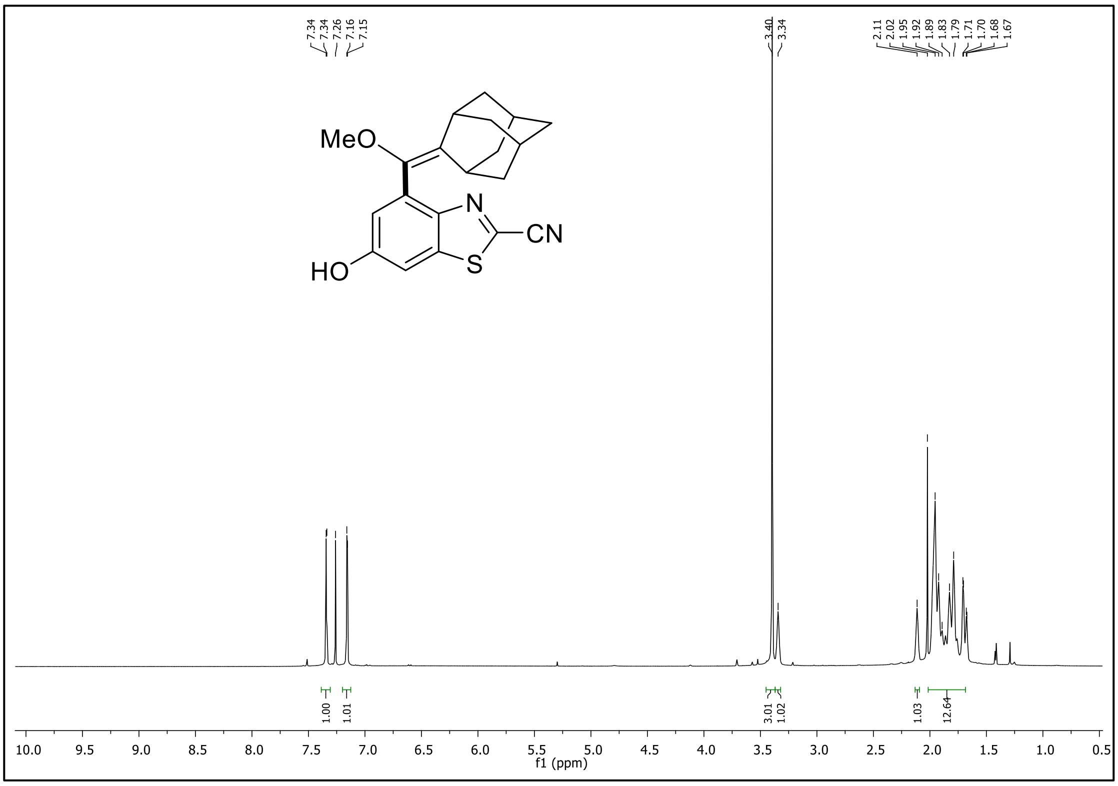

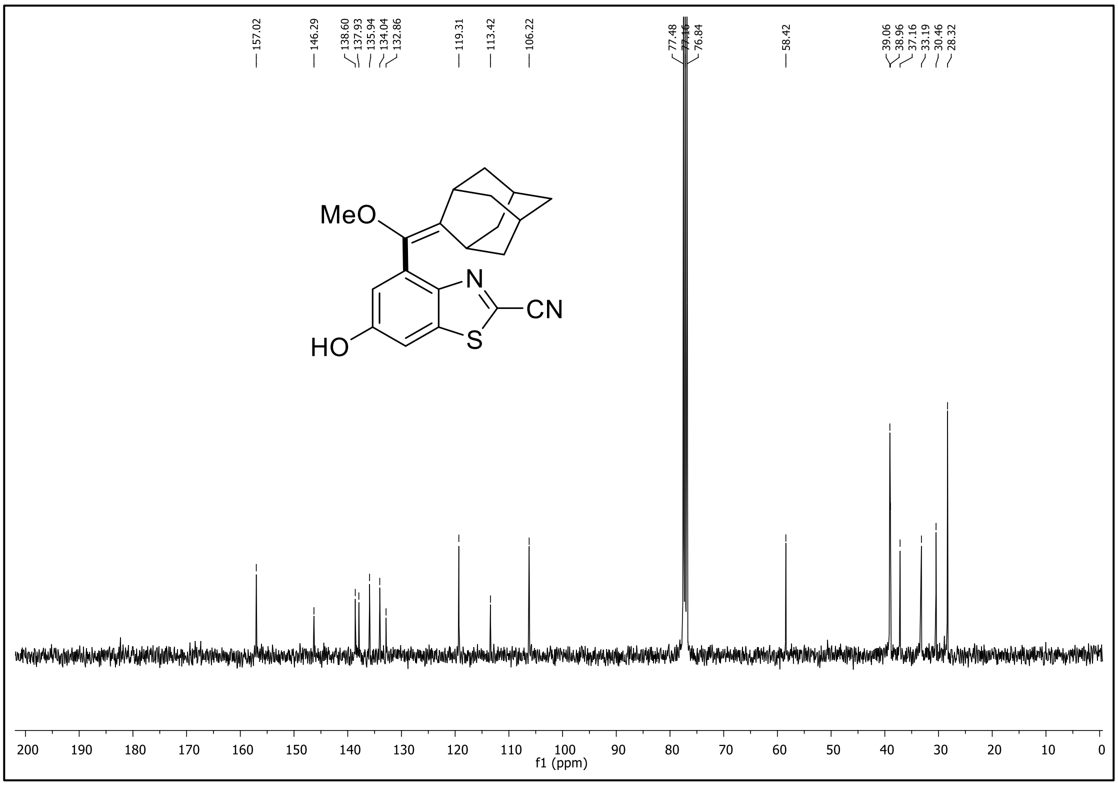

MS of compound **6s:**

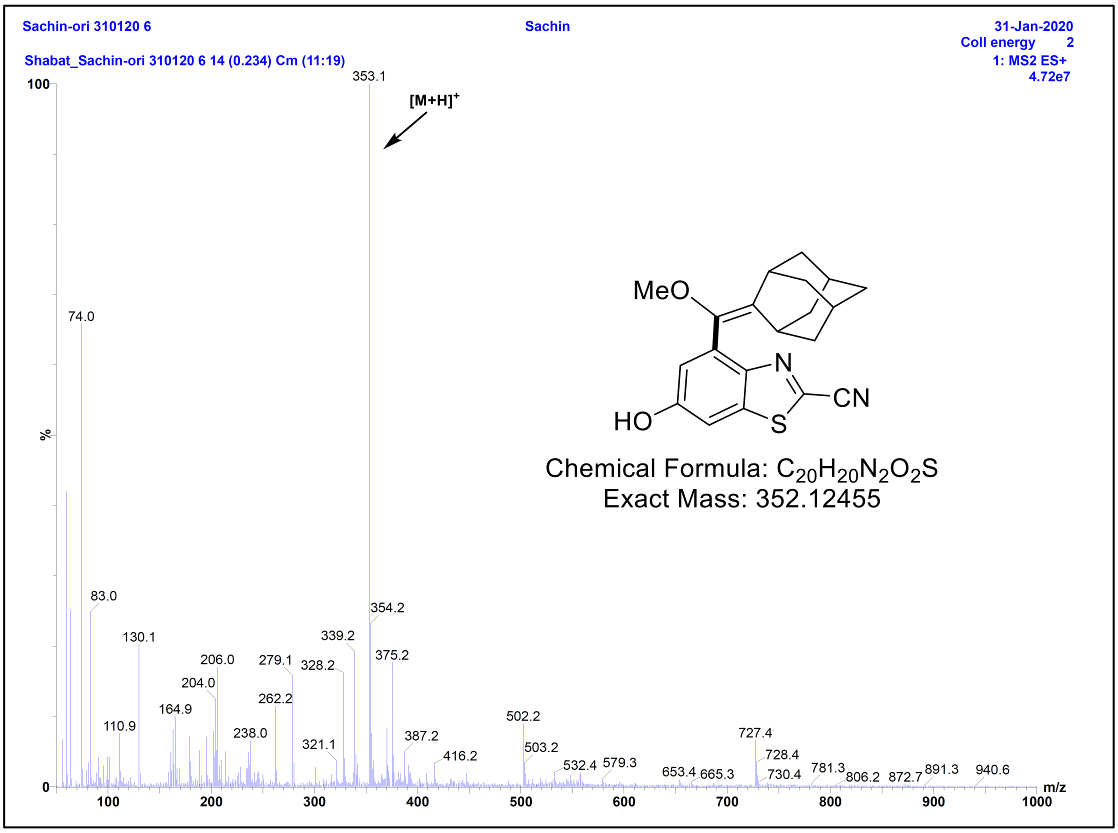

^{1}H-NMR and ^{13}C-NMR spectra of compound **6t:**

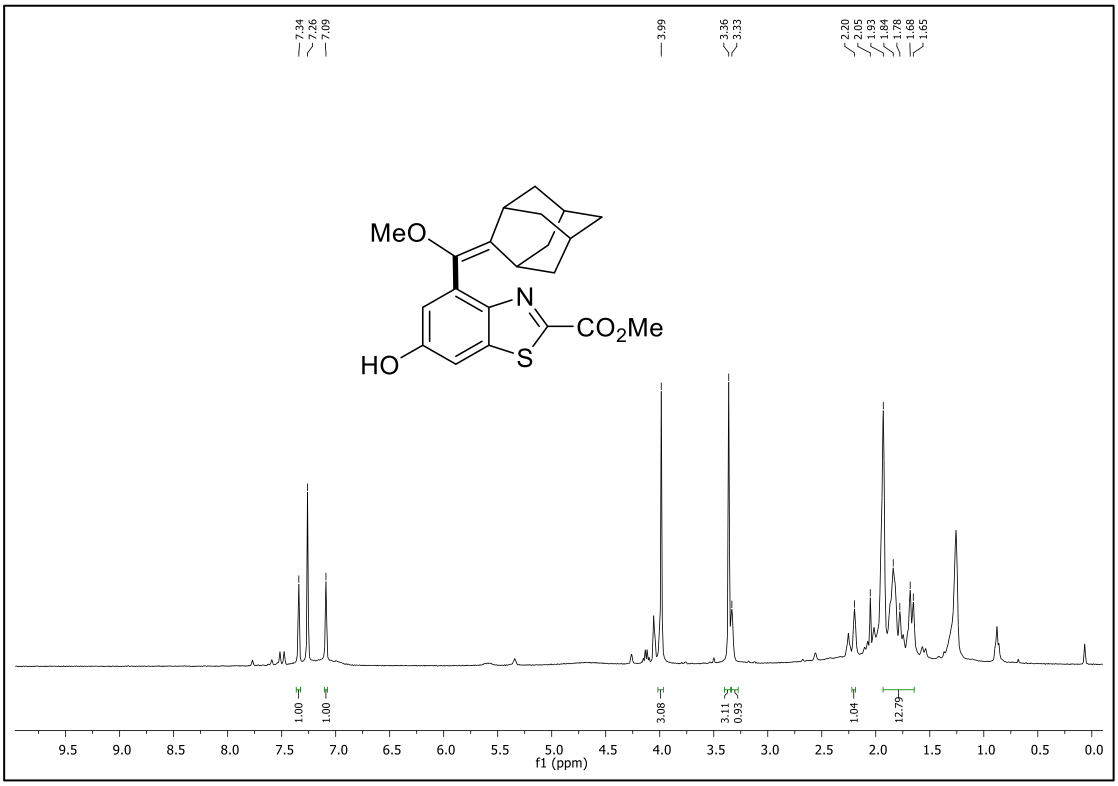

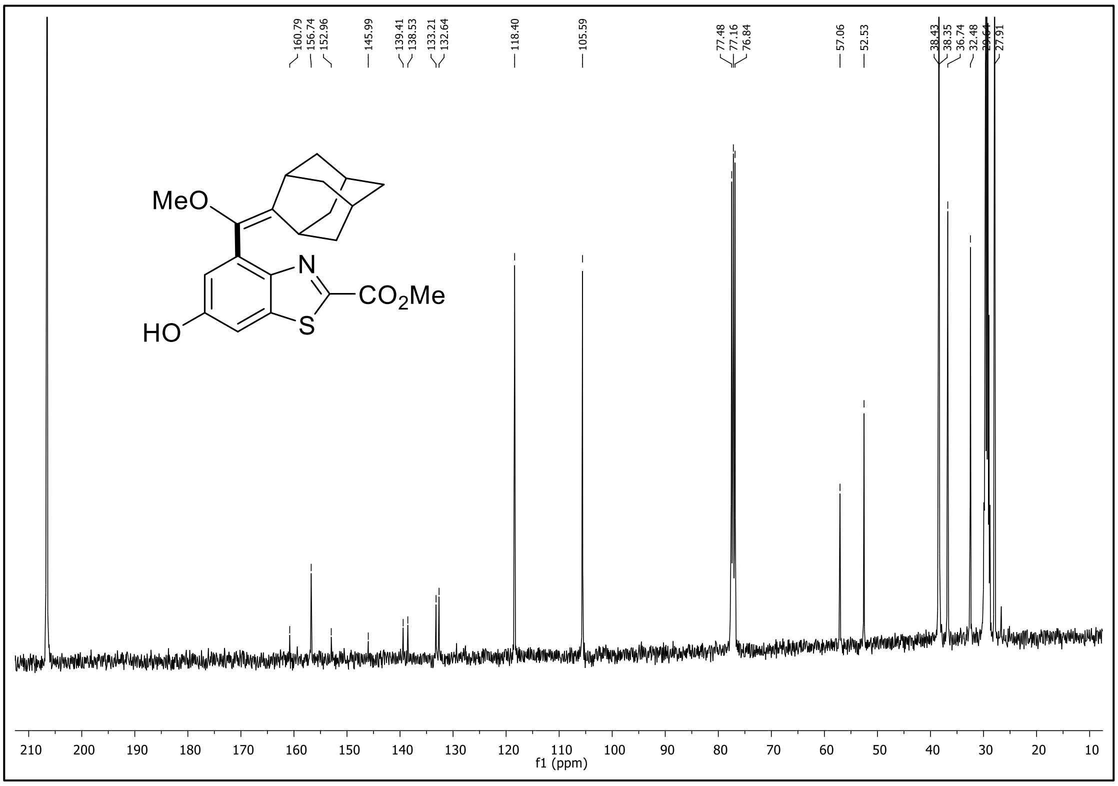

MS of compound **6t:**

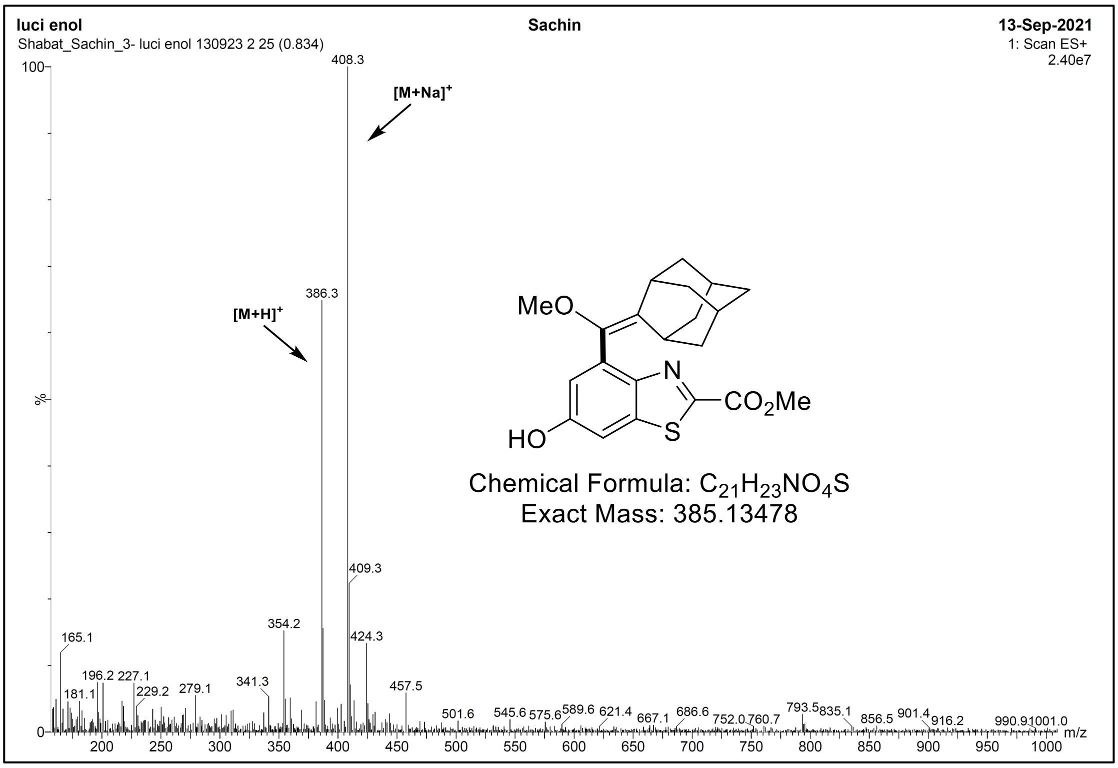

^{1}H-NMR and ^{13}C-NMR spectra of compound **6u:**

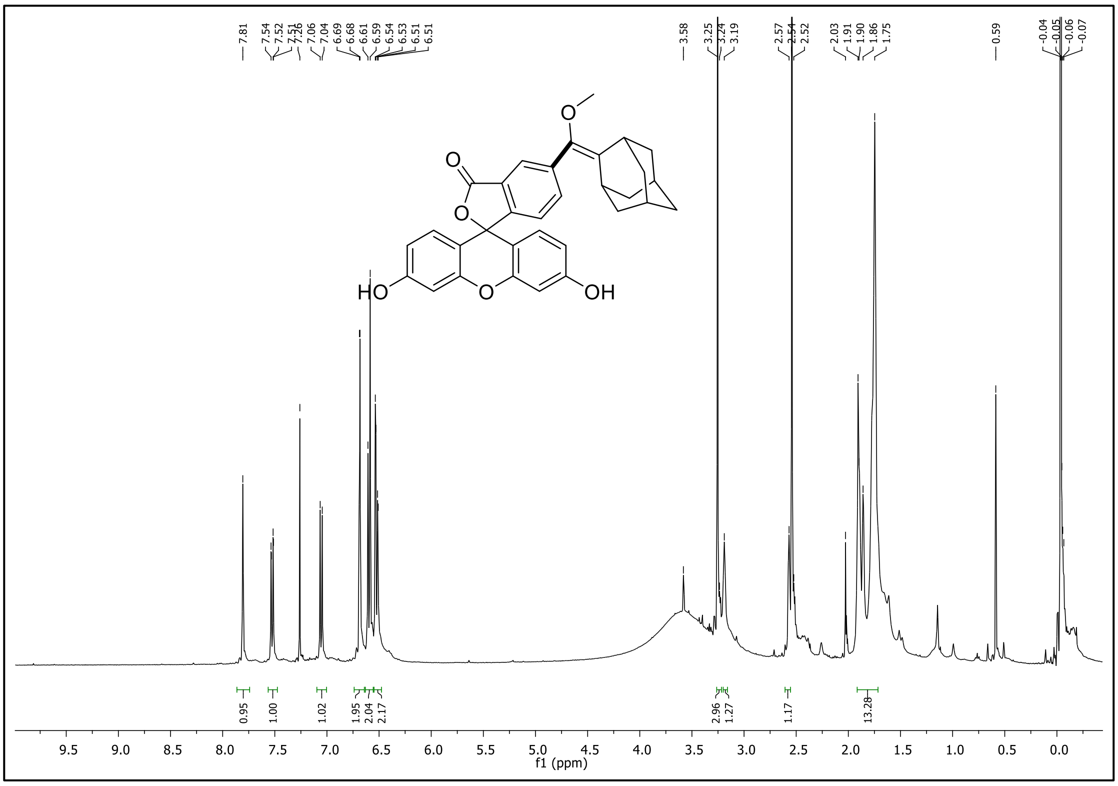

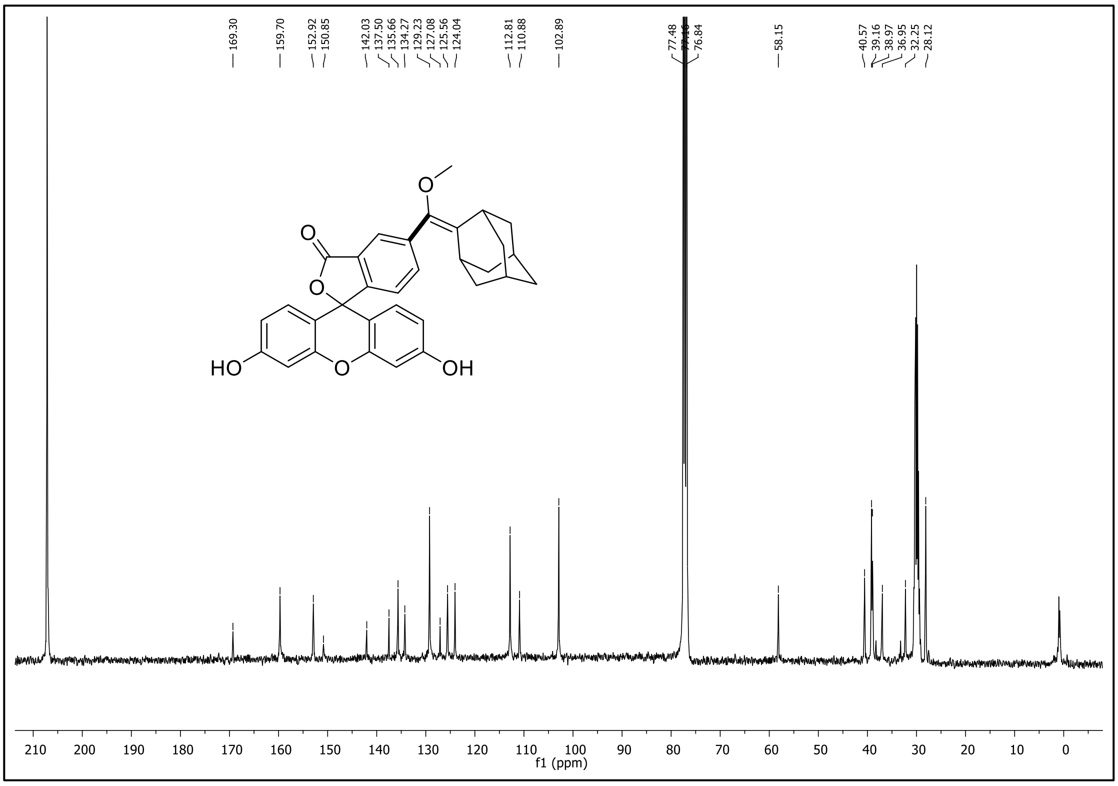

MS of compound **6u:**

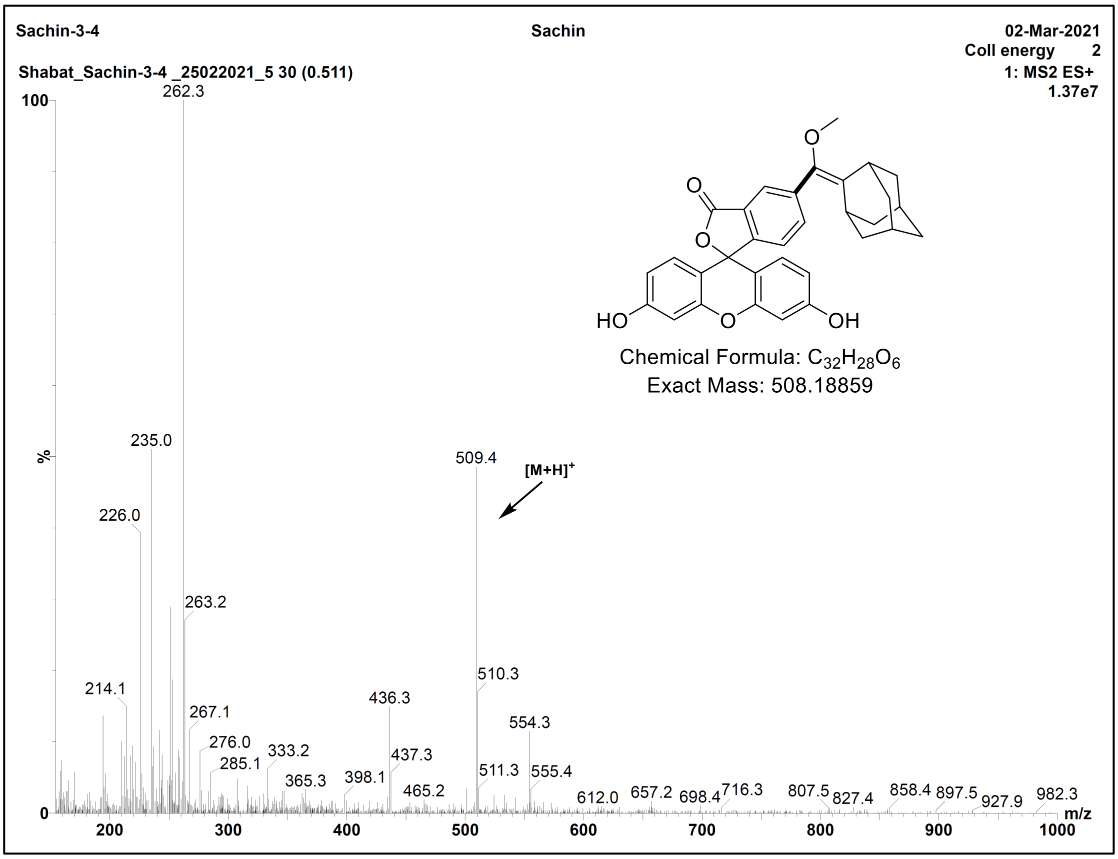

^{1}H-NMR and ^{13}C-NMR spectra of compound **6v:**

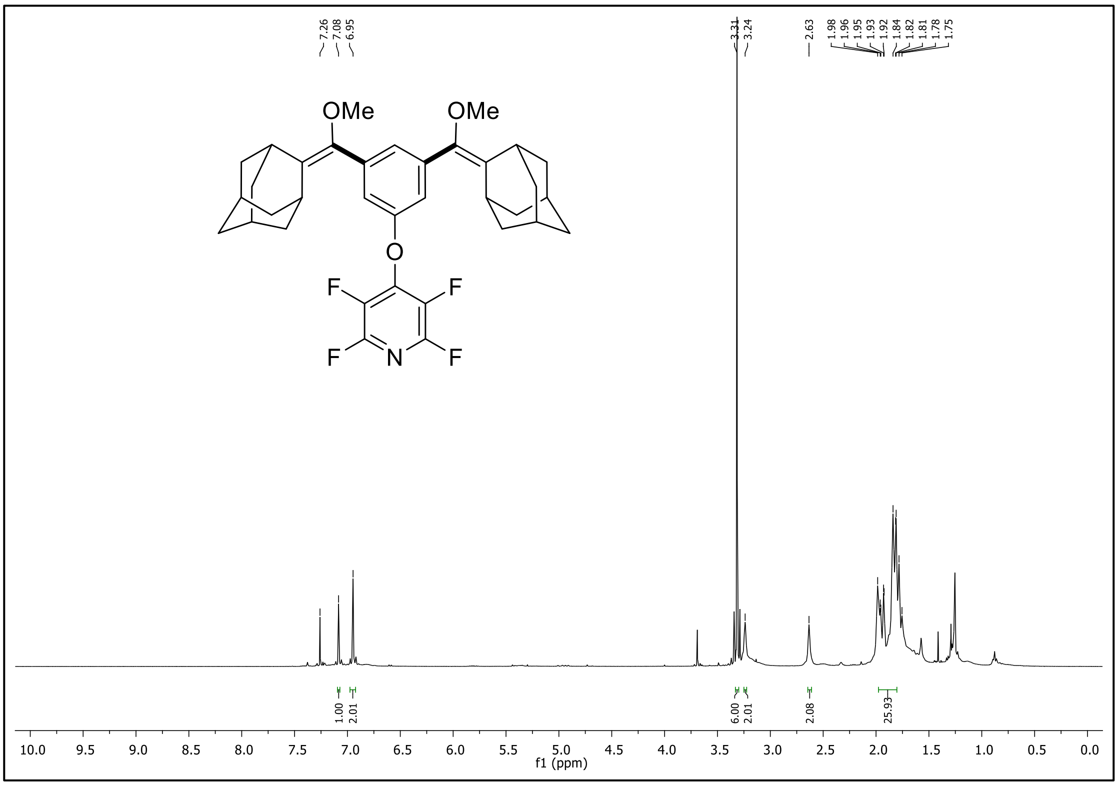

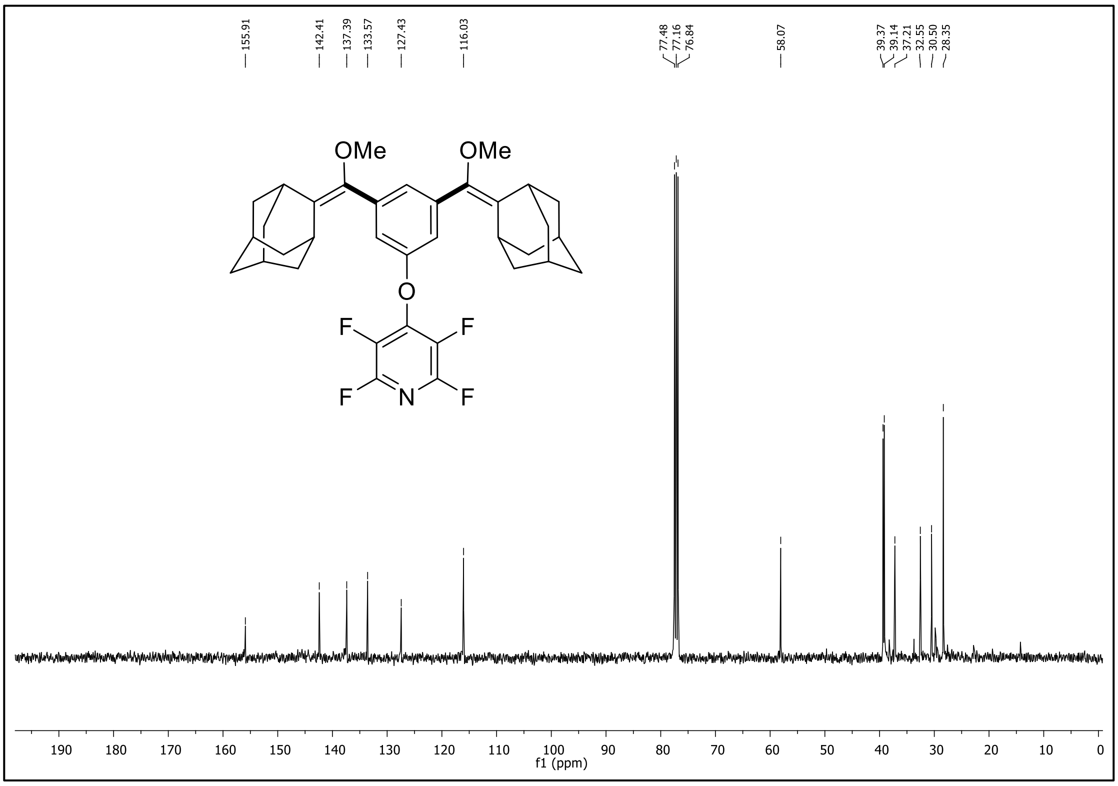

^{19}F-NMR spectra of compound **6v:**

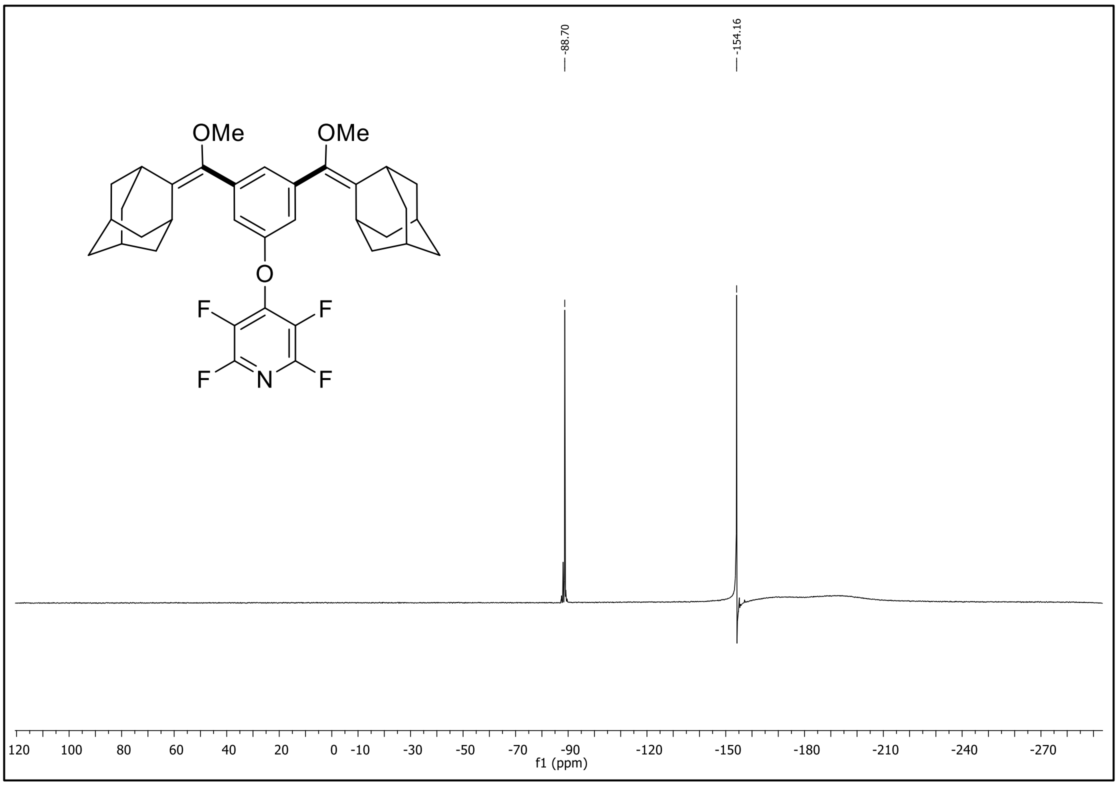

MS of compound **6v:**

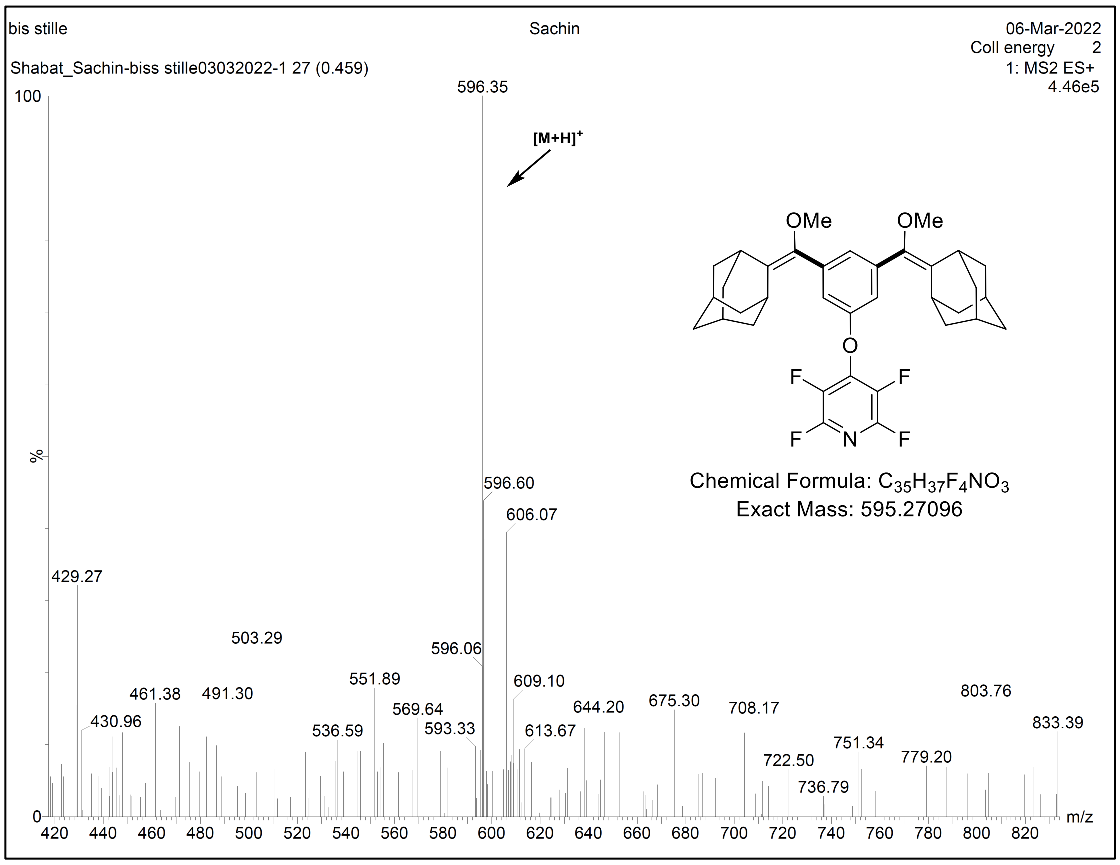

^{1}H-NMR spectra of compound **6w: (This compond was obtained in the form of two atropisomers.)**

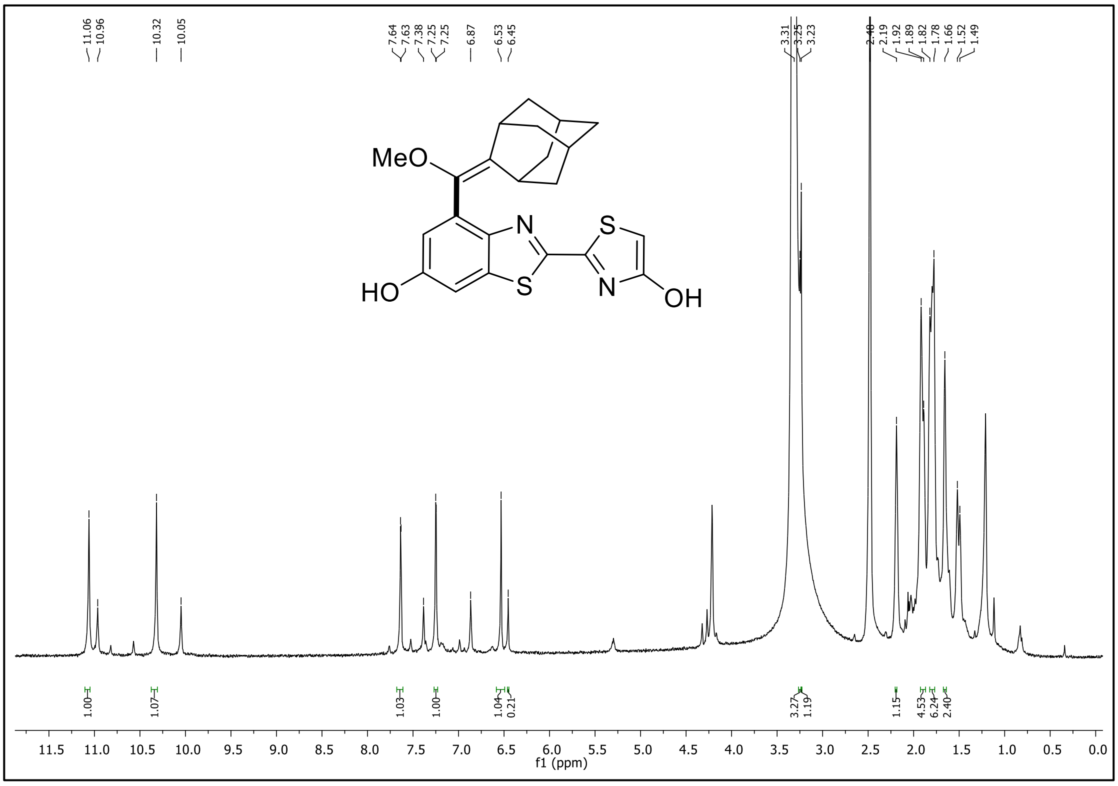

MS of compound **6w:**

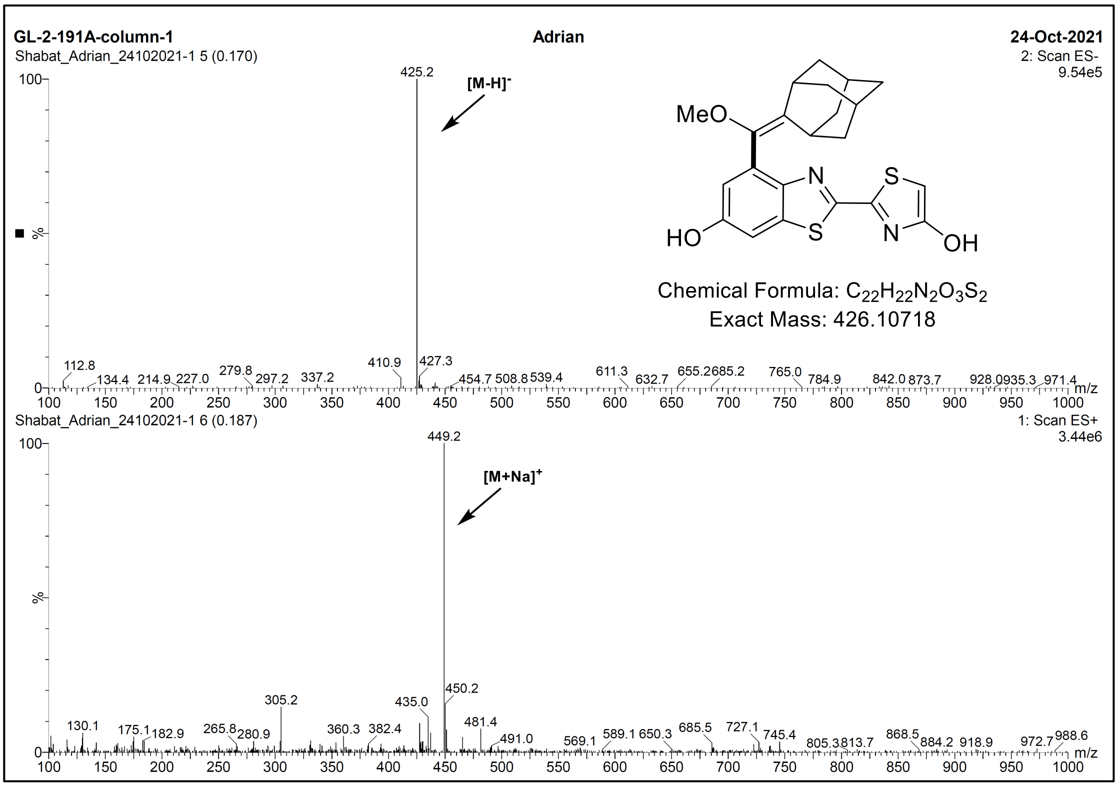

^{1}H-NMR and ^{13}C-NMR spectra of compound **6x:**

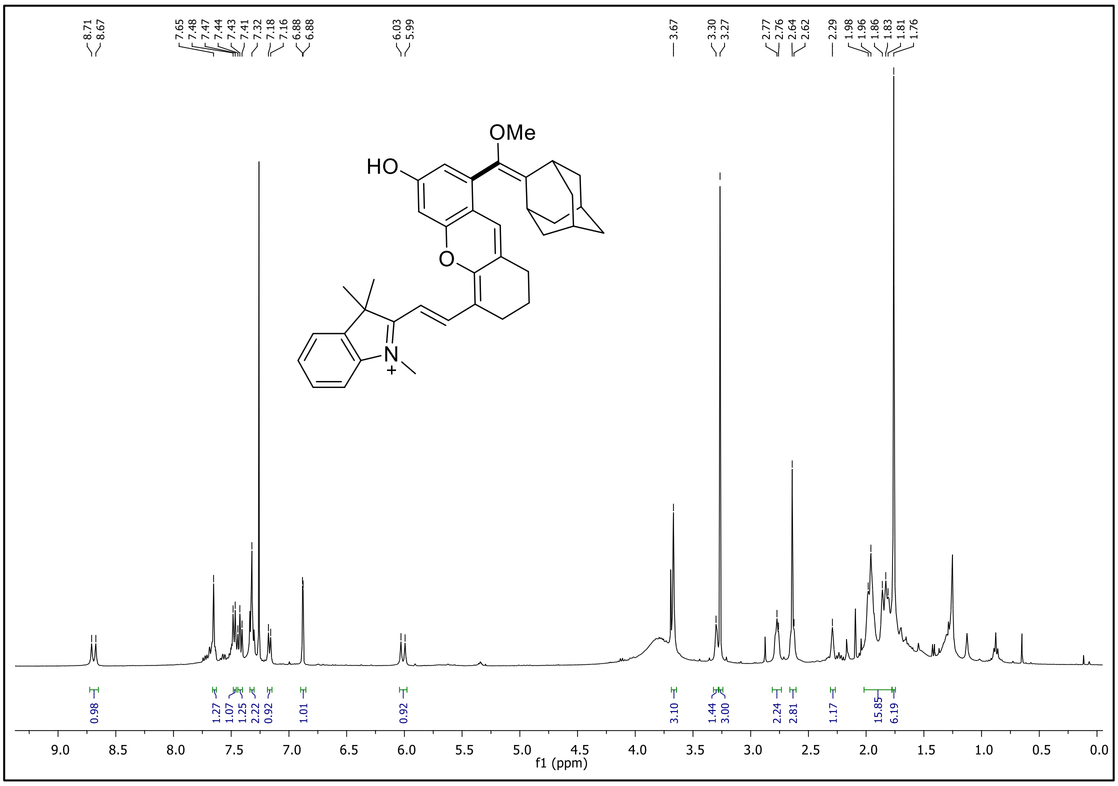

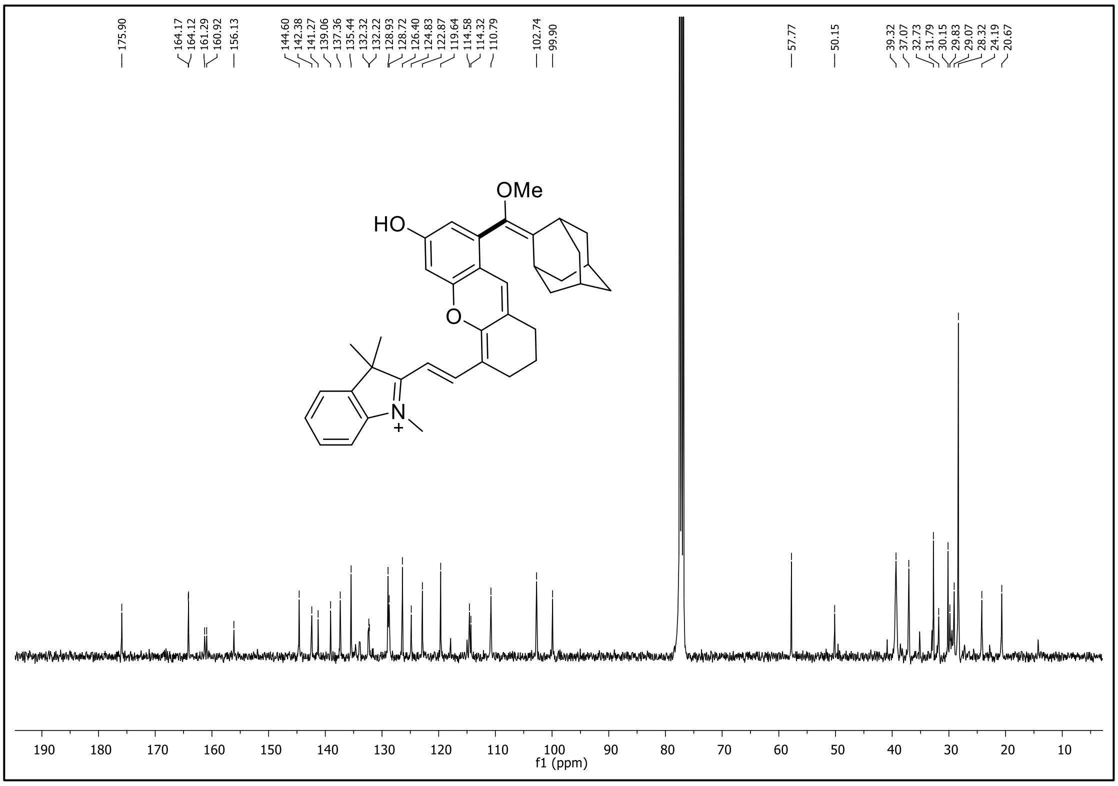

MS of compound **6x:**

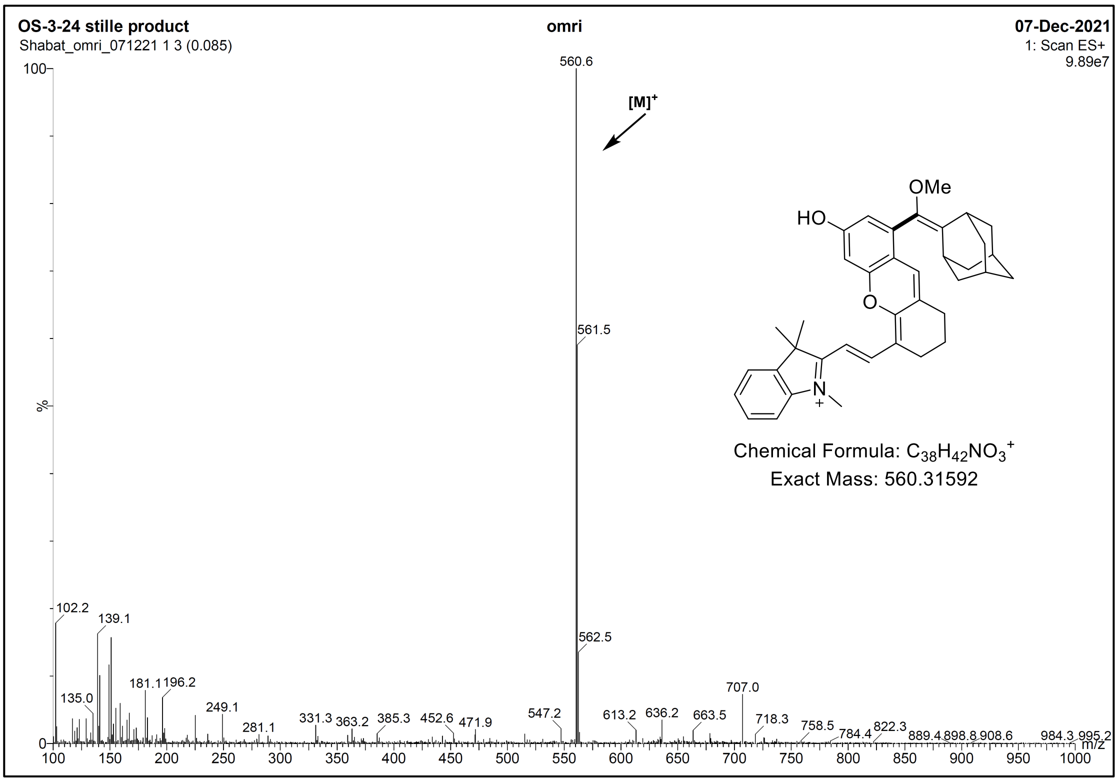

^{1}H-NMR and ^{13}C-NMR spectra of compound **9a:**

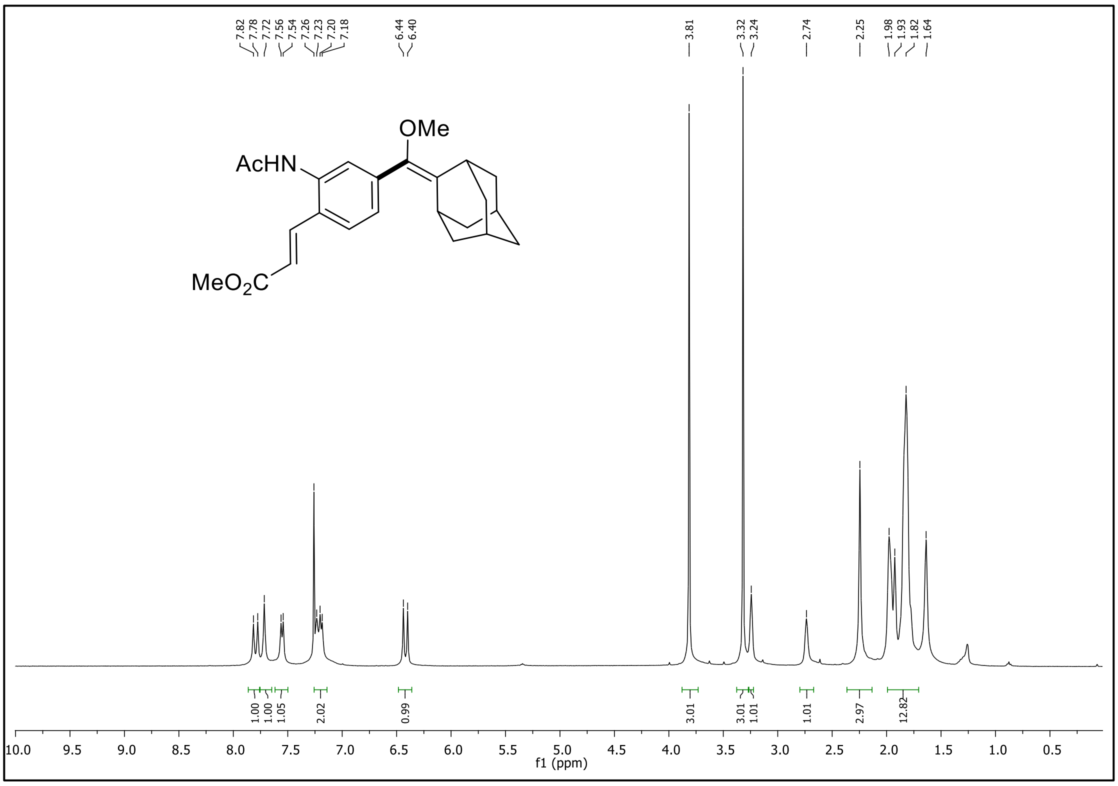

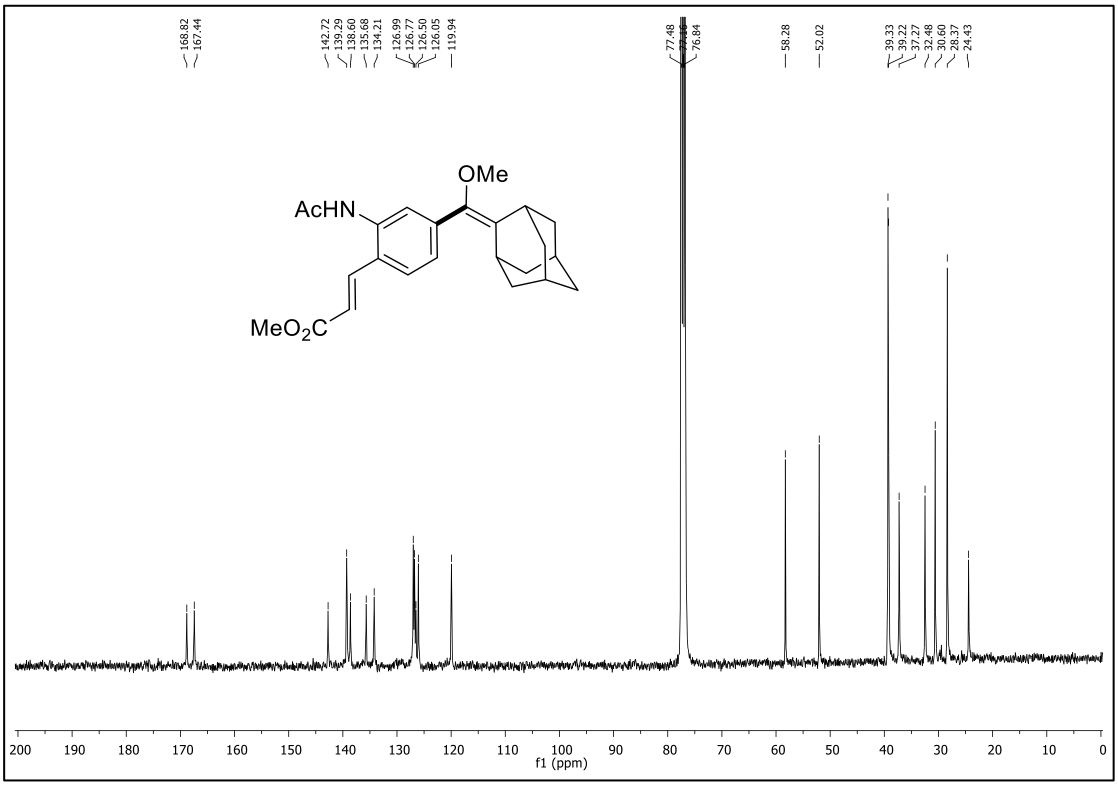

MS of compound **9a:**

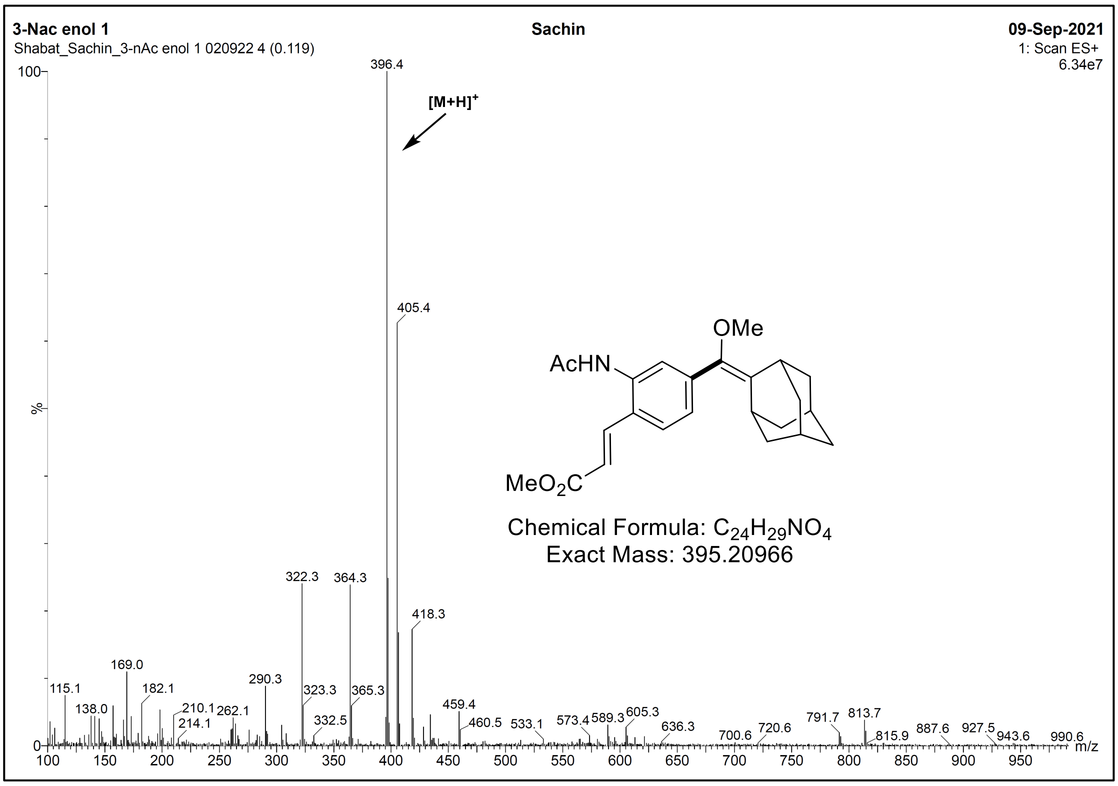

^{1}H-NMR and ^{13}C-NMR spectra of compound **9b:**

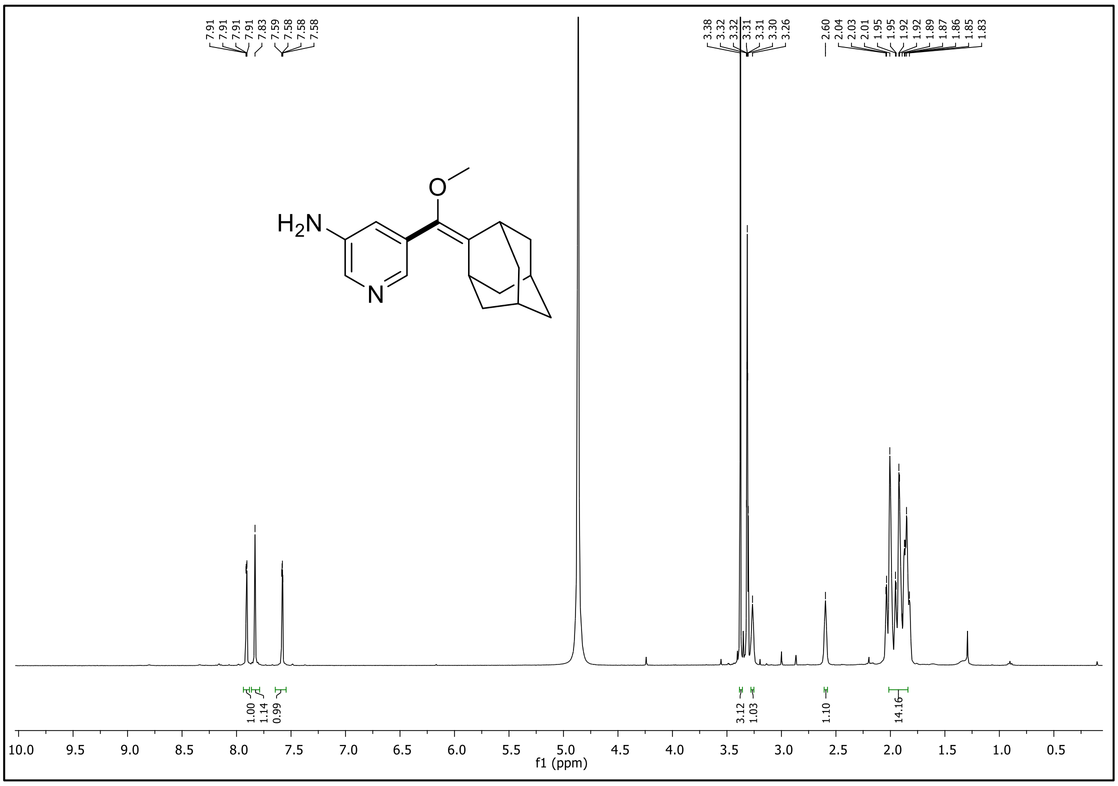

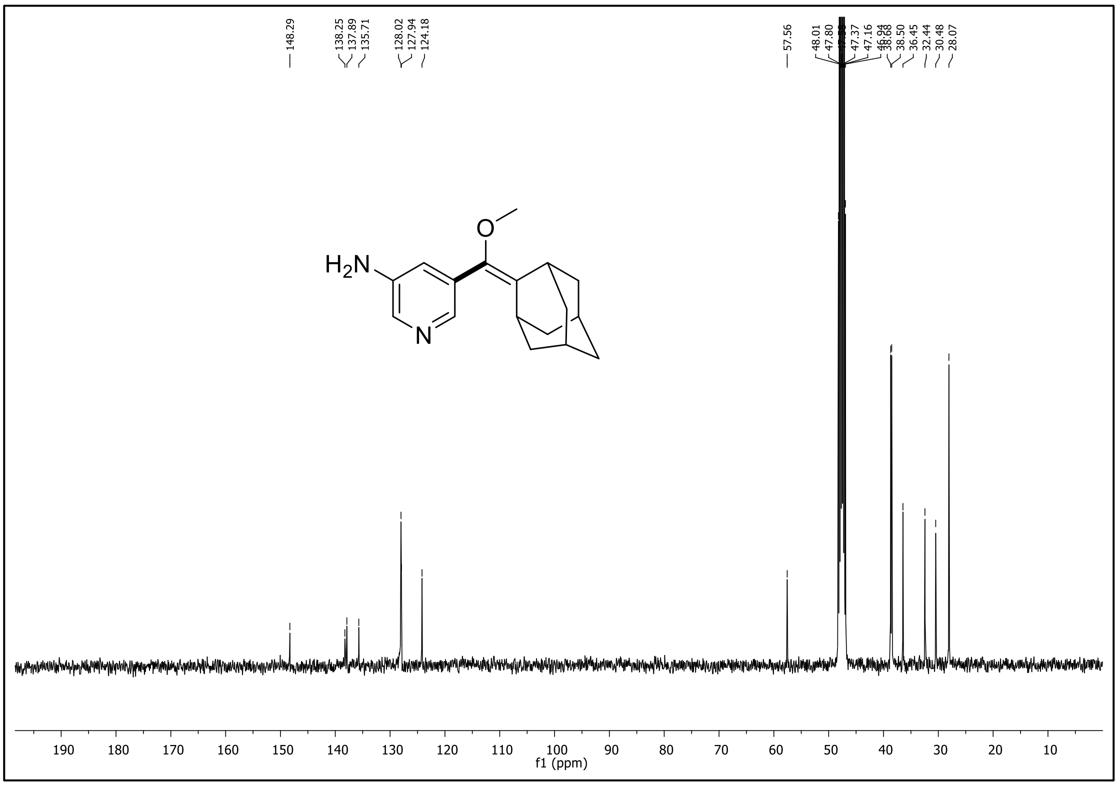

MS of compound **9b:**

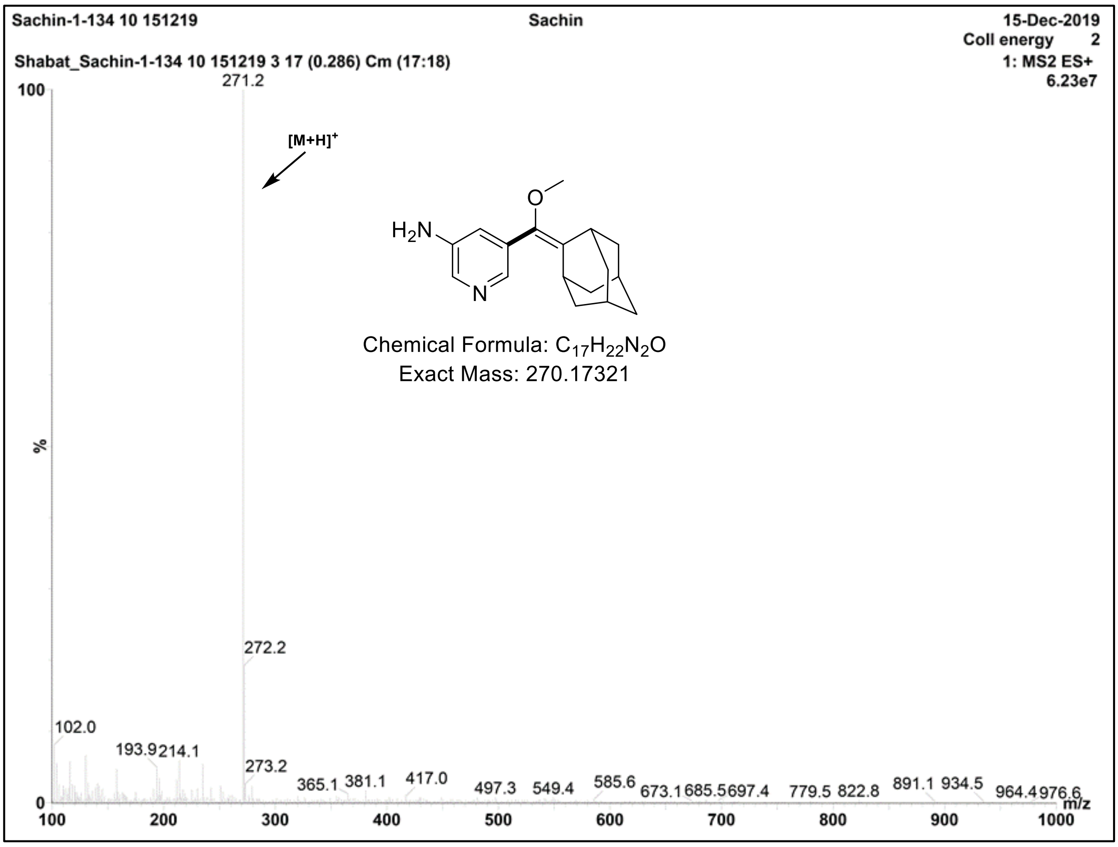

^{1}H-NMR and ^{13}C-NMR spectra of compound **9c:**

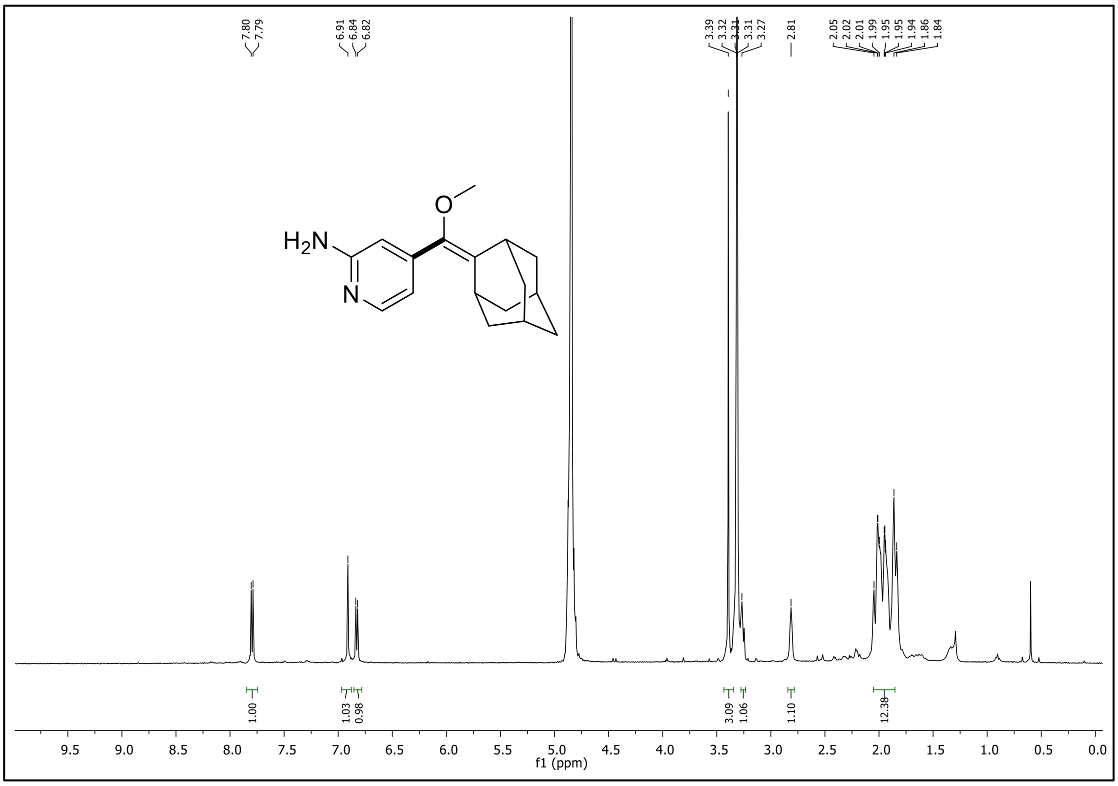

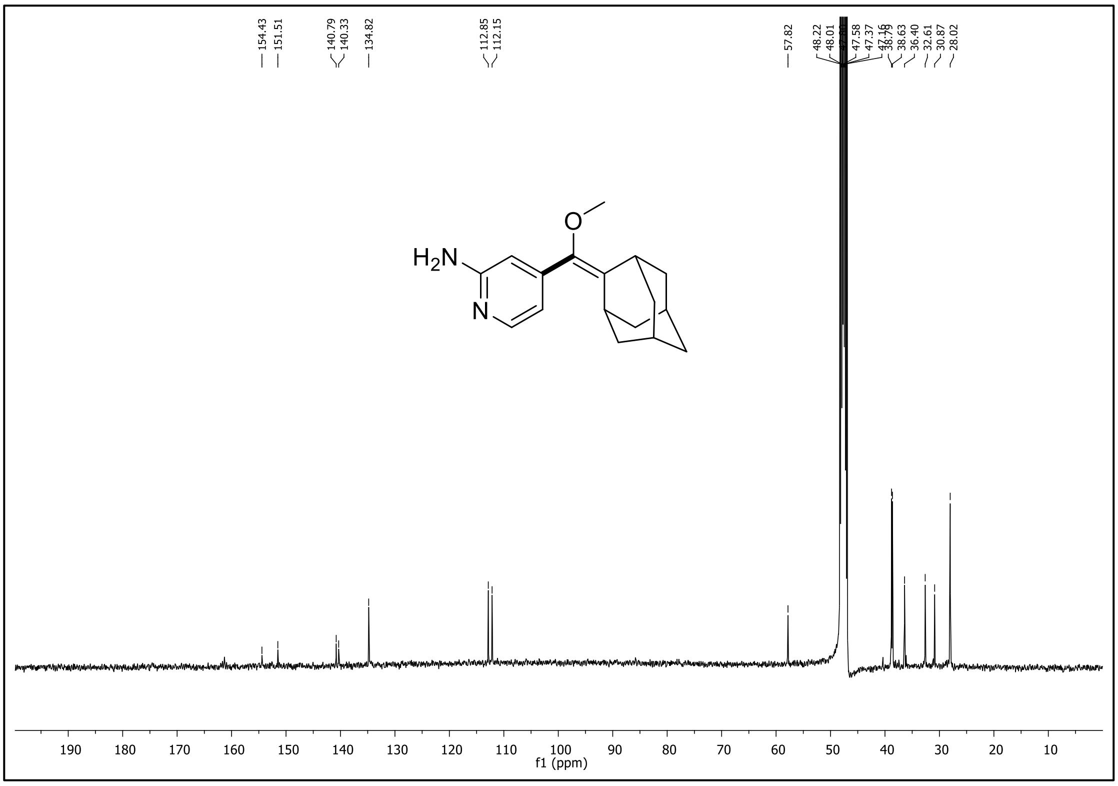

MS of compound **9c:**

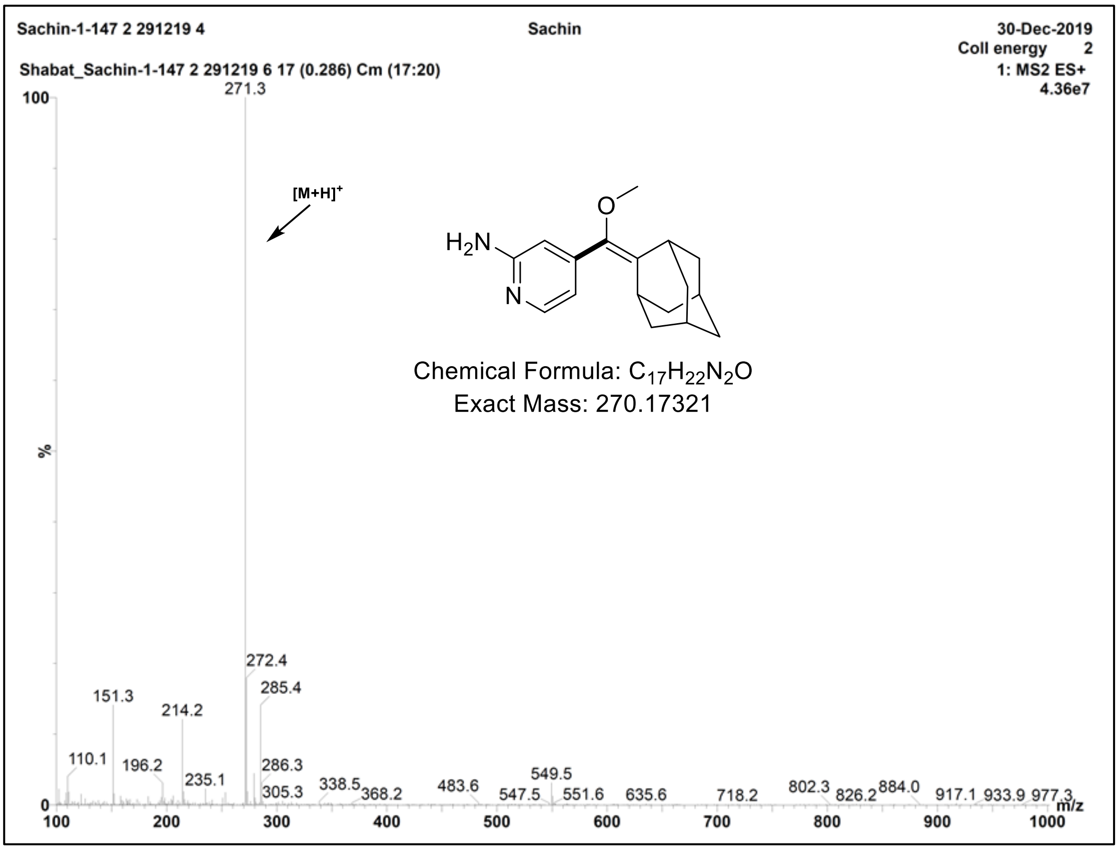

^{1}H-NMR and ^{13}C-NMR spectra of compound **9d:**

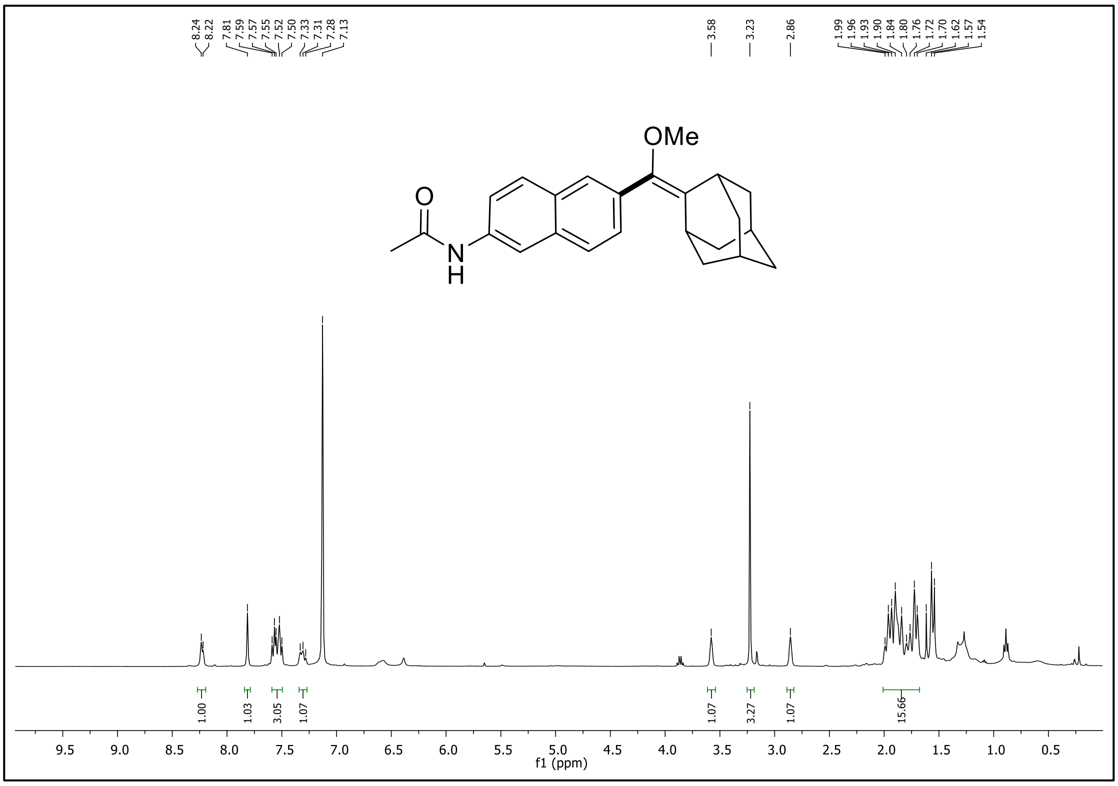

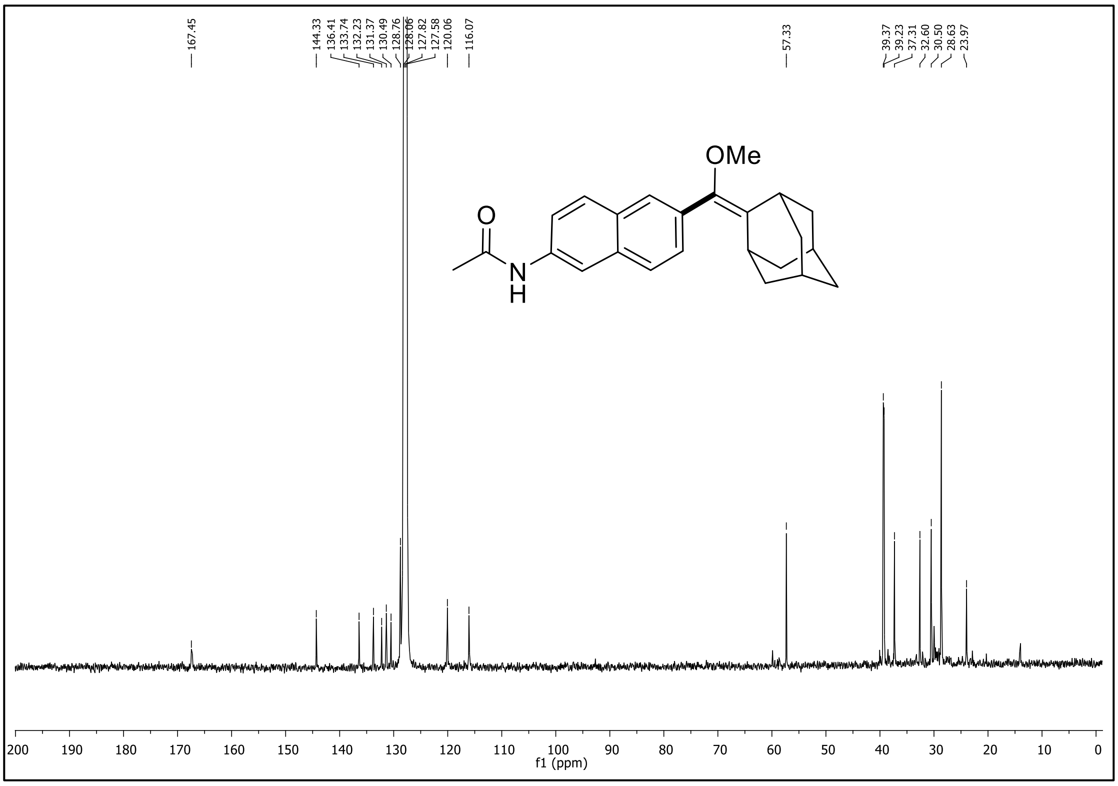

MS of compound **9d:**

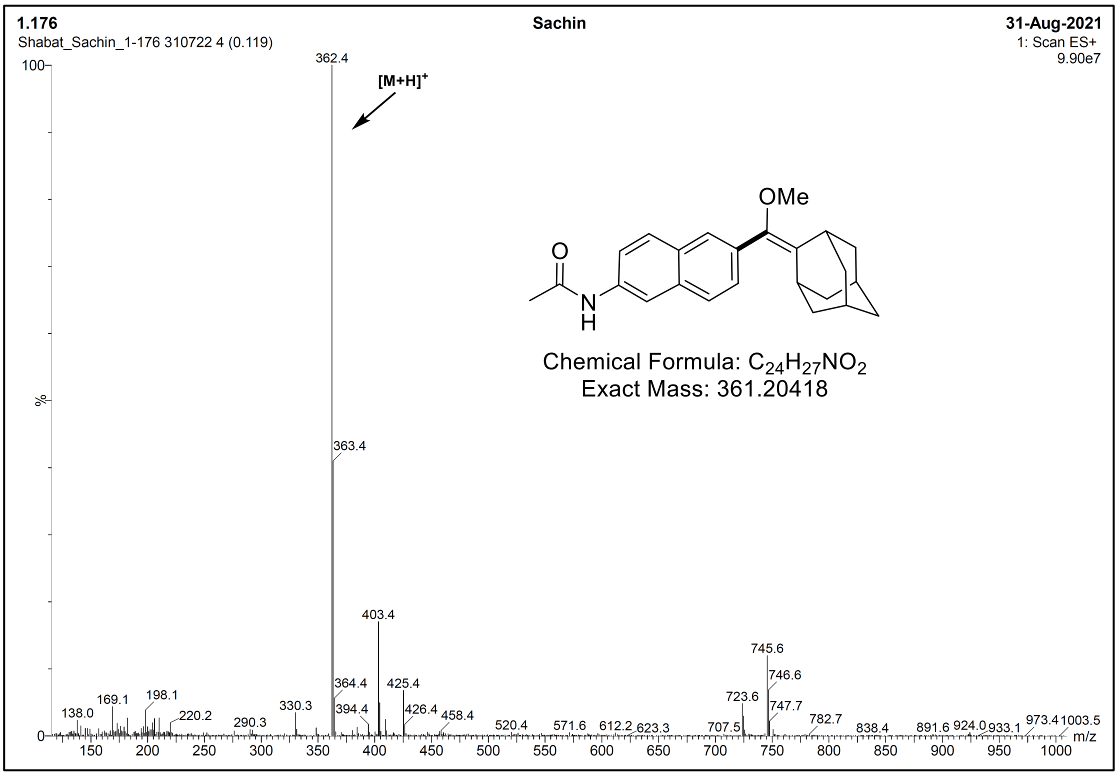

^{1}H-NMR and ^{13}C-NMR spectra of compound **9e:**

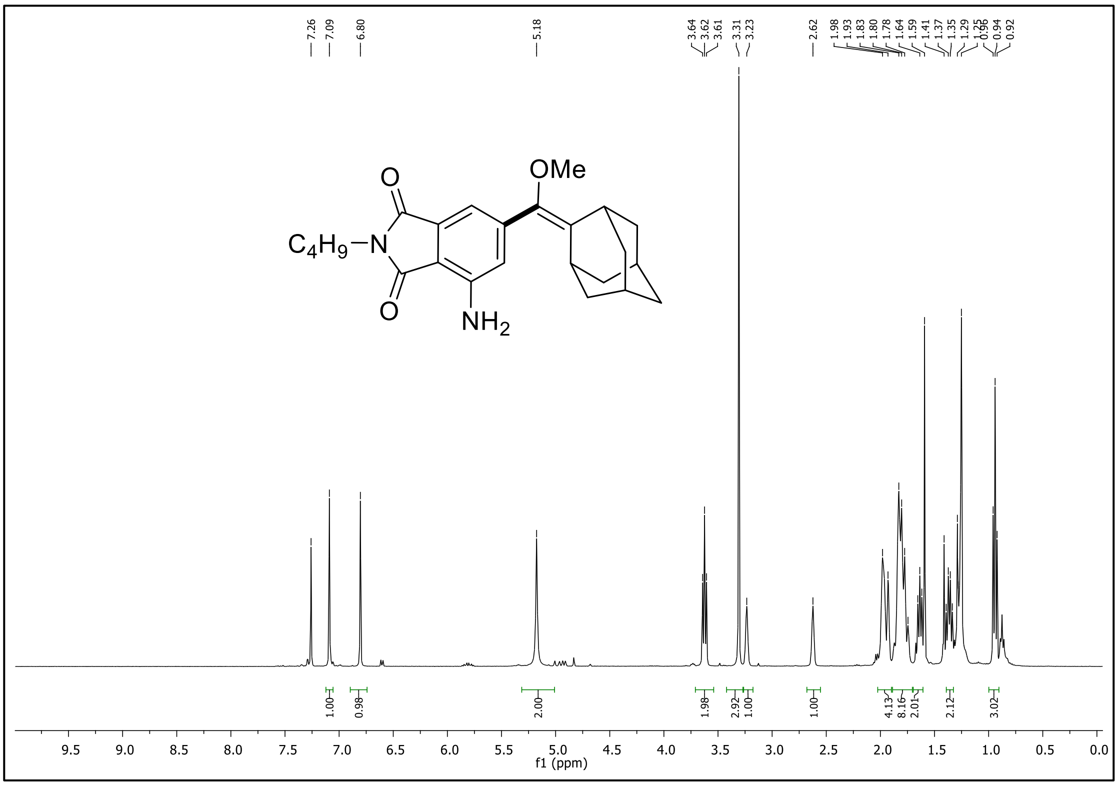

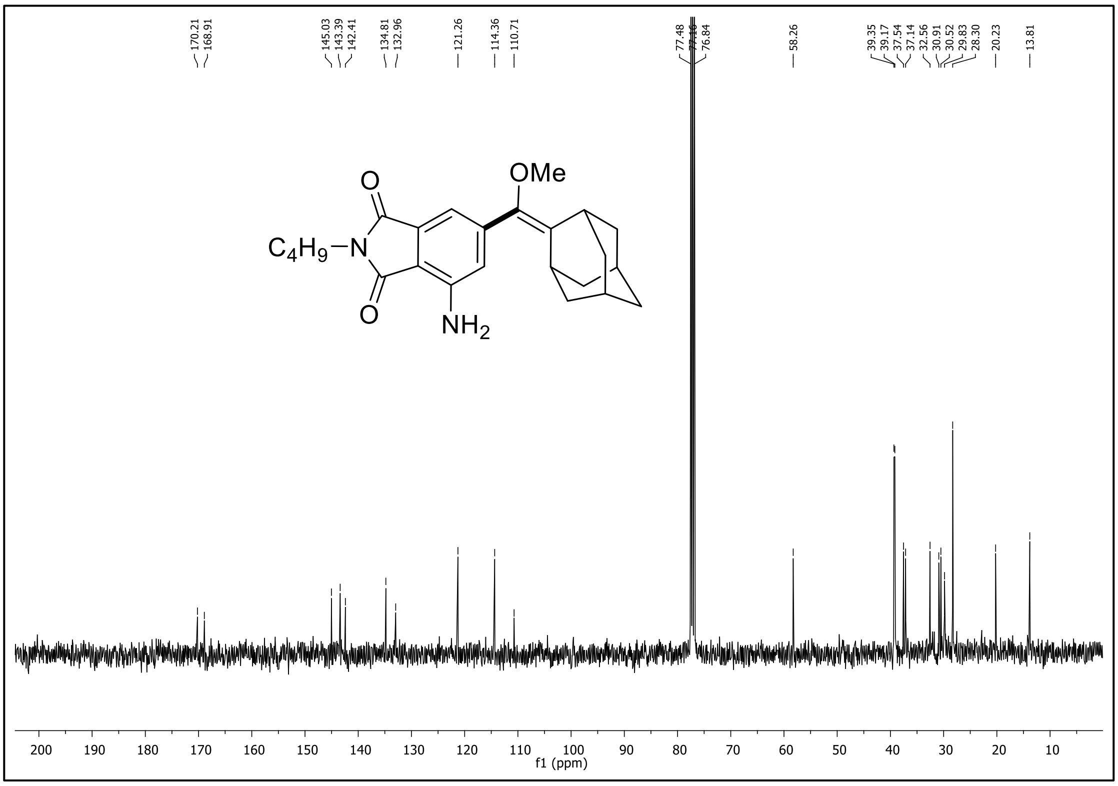

MS of compound **9e:**

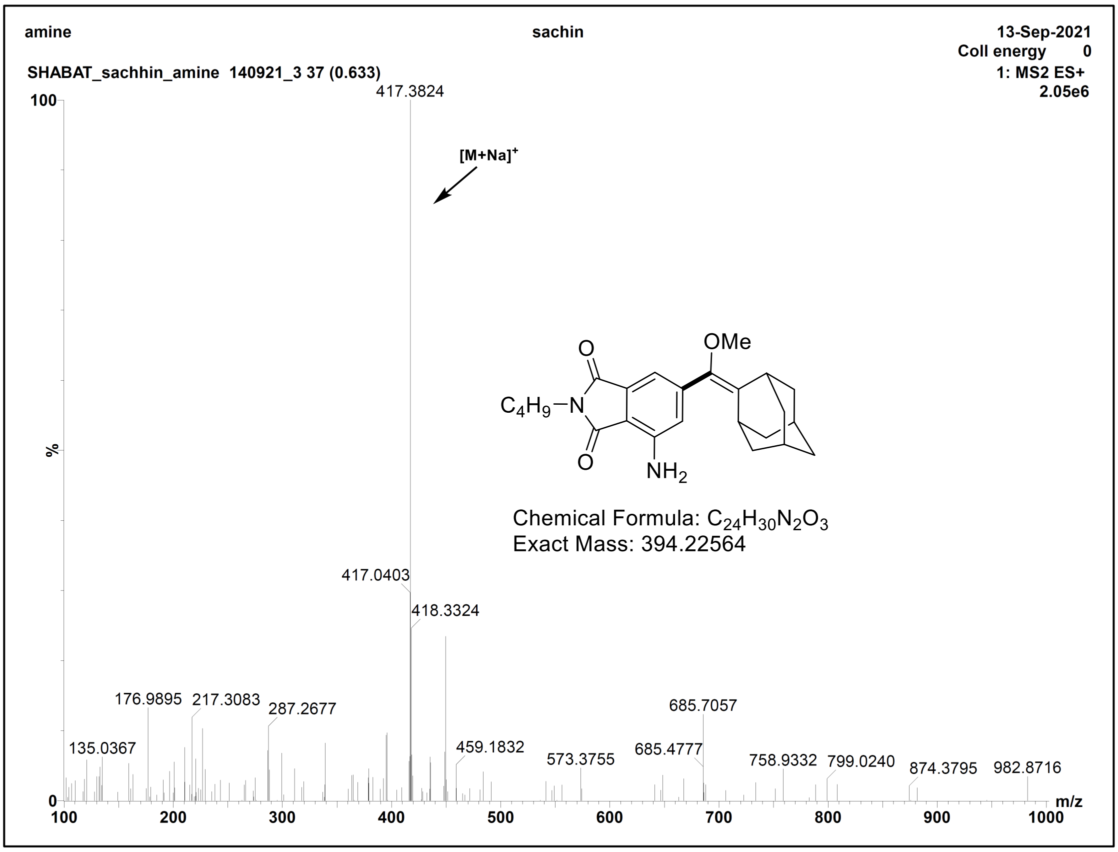

^{1}H-NMR and ^{13}C-NMR spectra of compound **9f:**

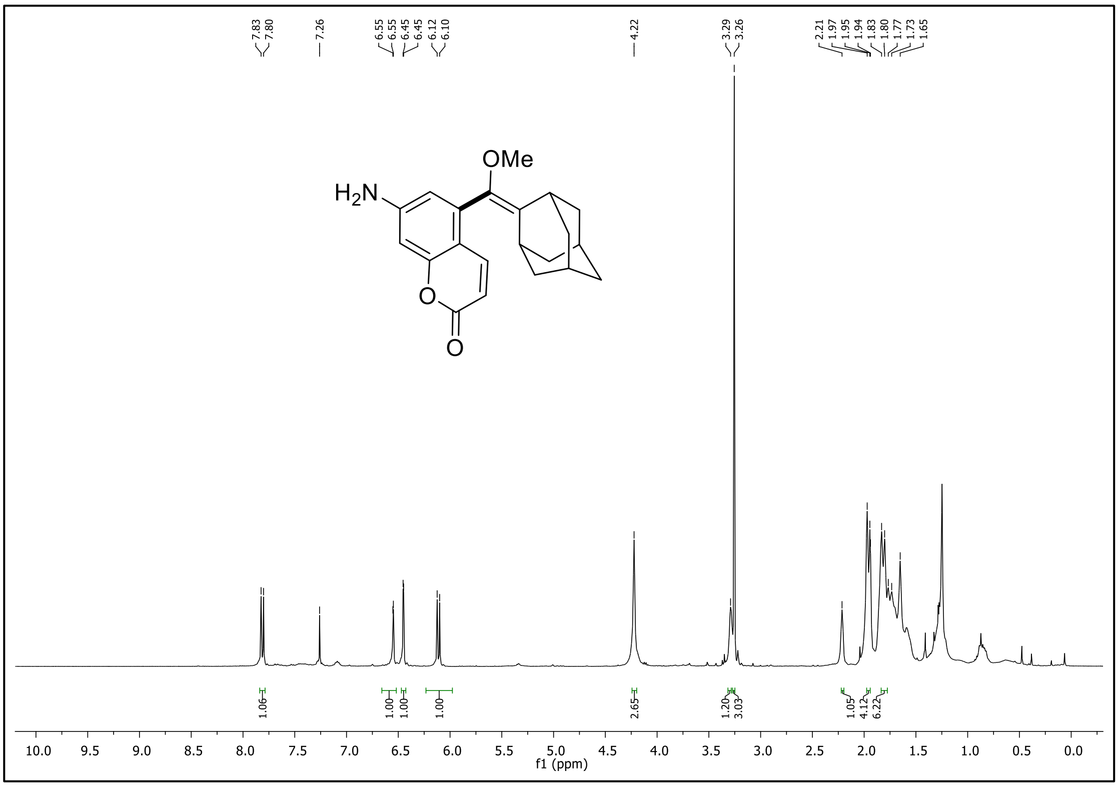

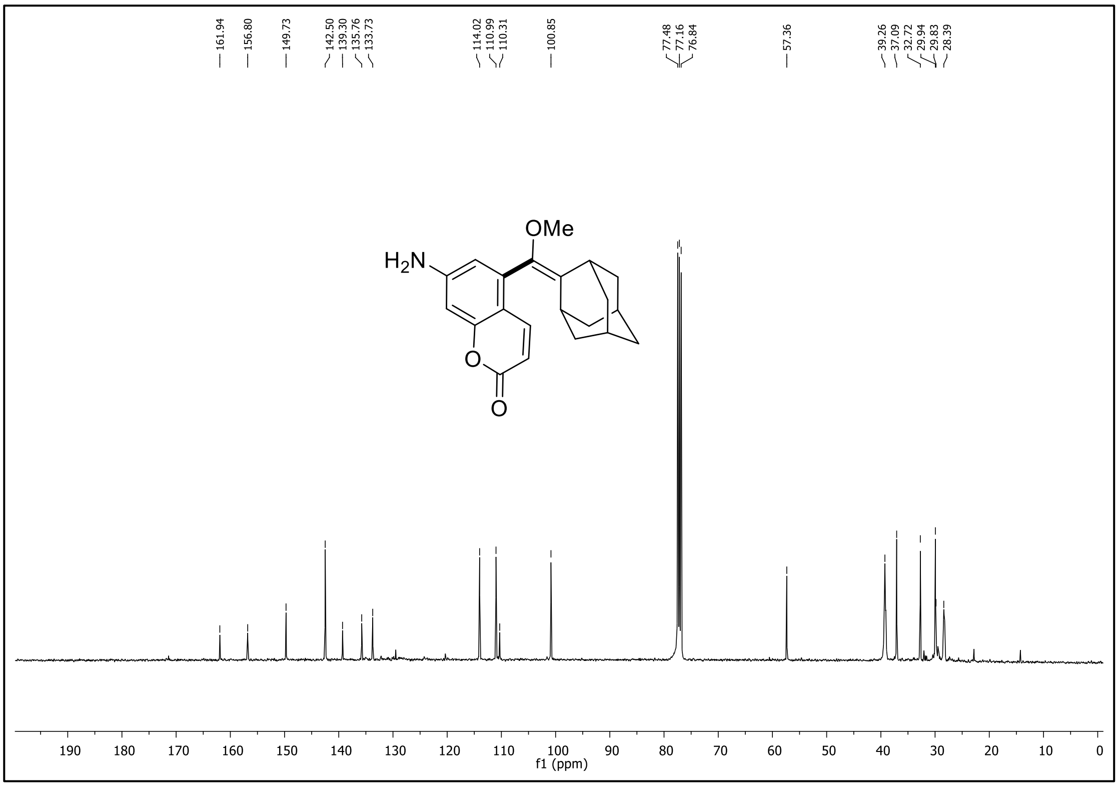

MS of compound **9f:**

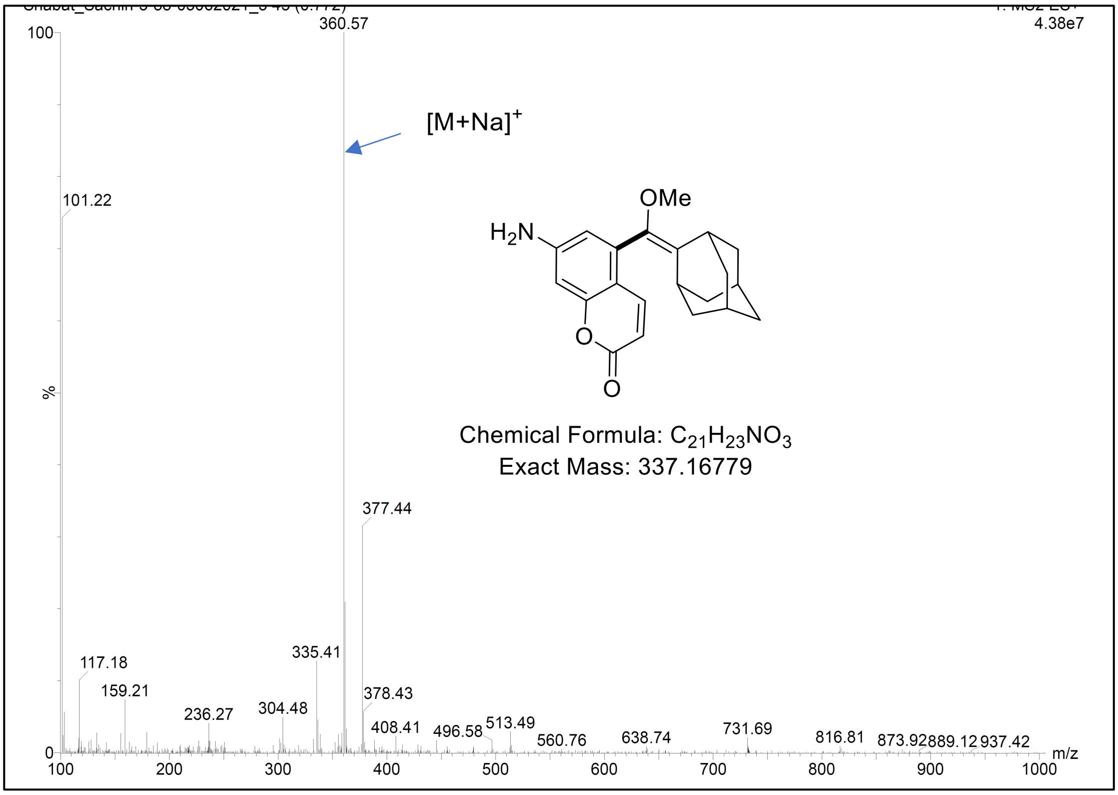

^{1}H-NMR and ^{13}C-NMR spectra of compound **9g:**

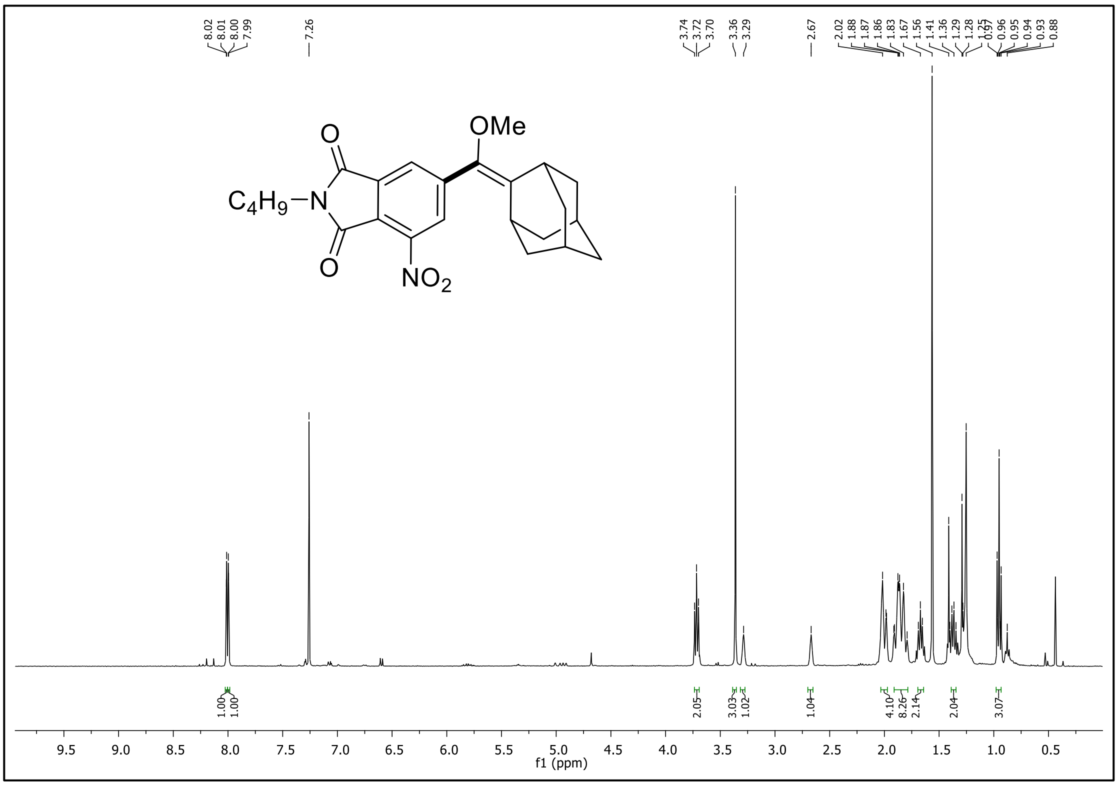

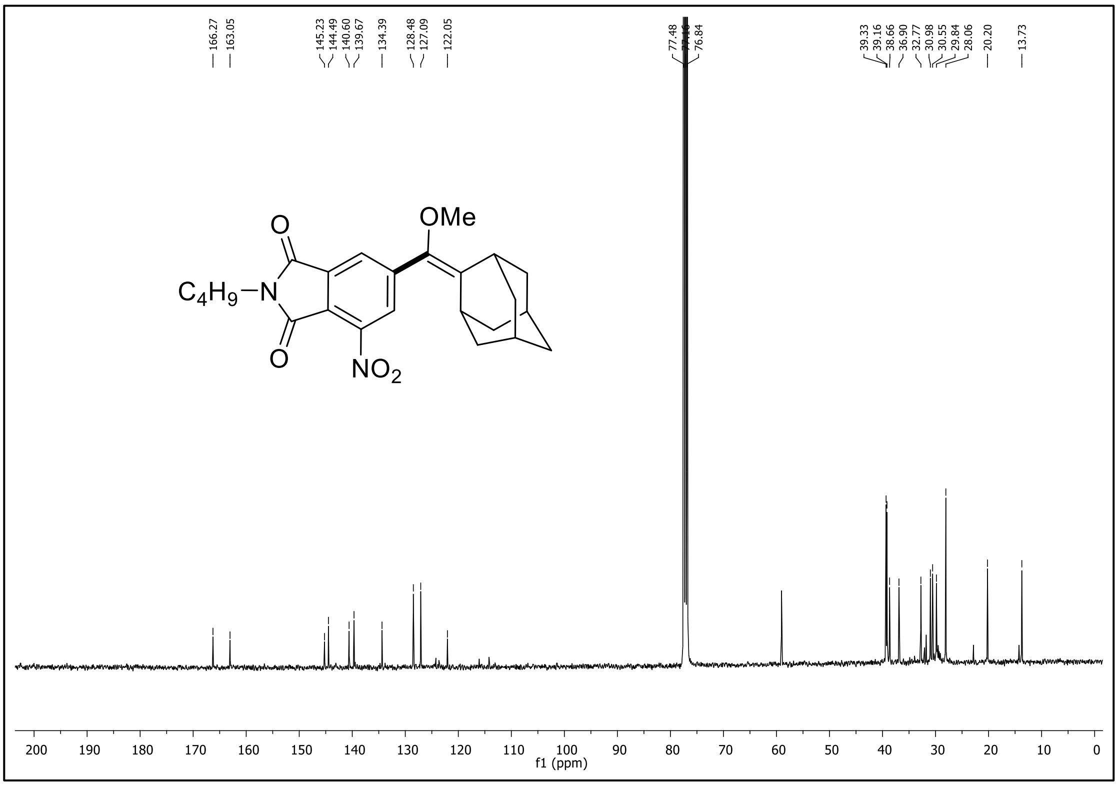

MS of compound **9g:**

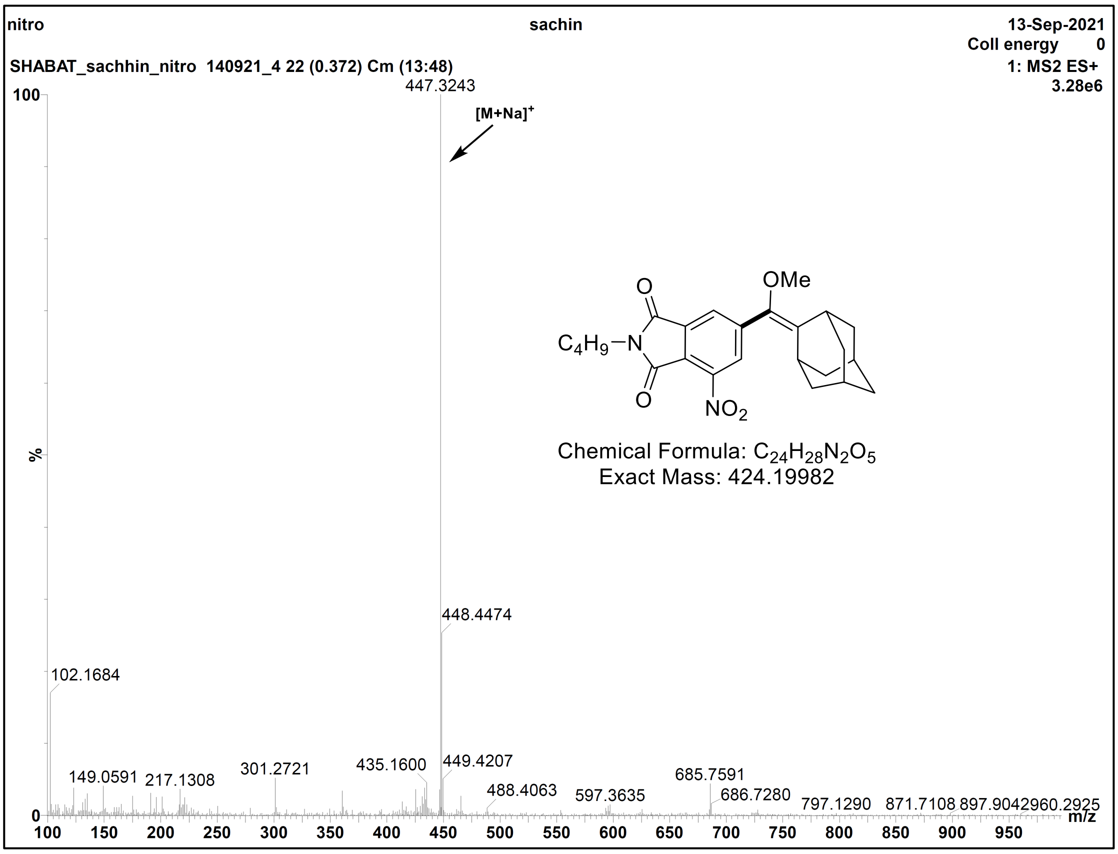

^{1}H-NMR and ^{13}C-NMR spectra of compound **9h:**

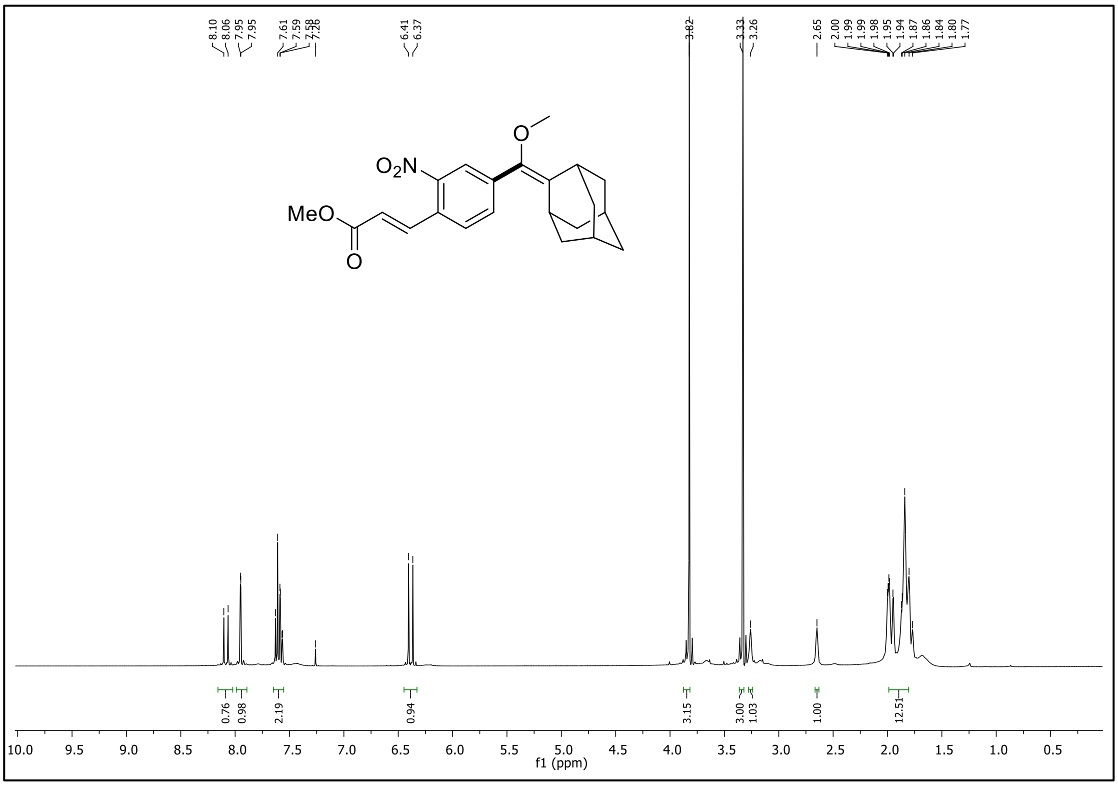

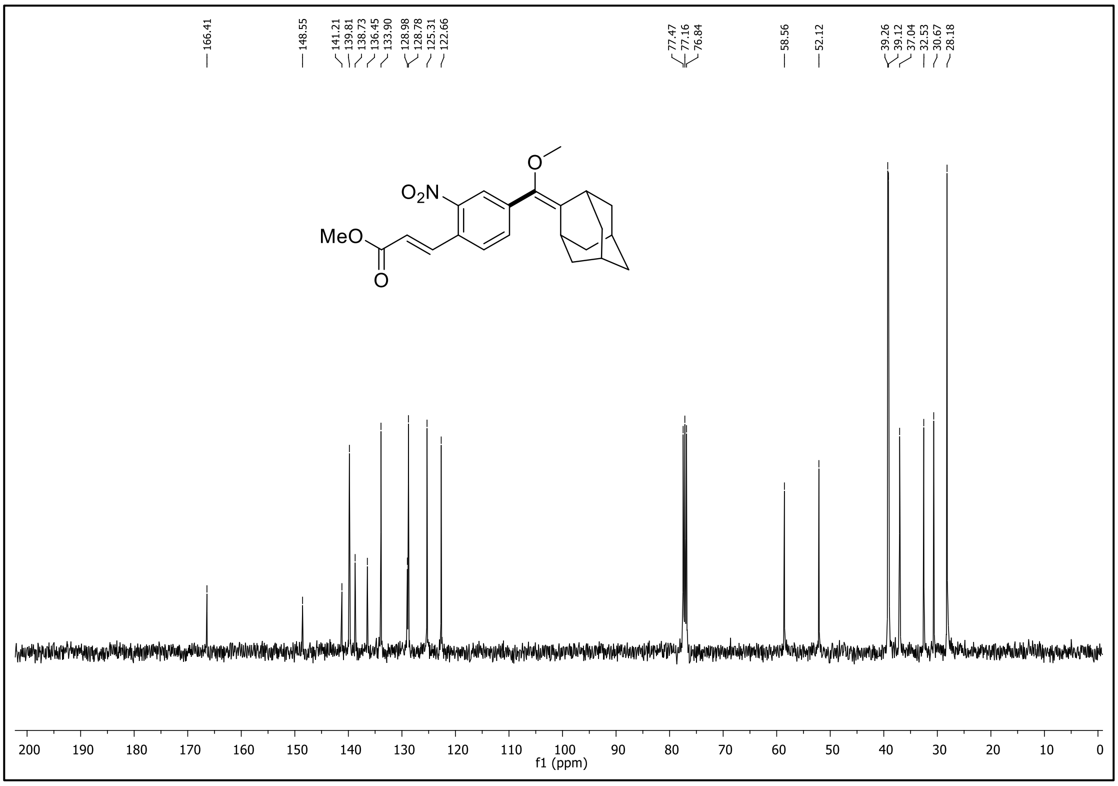

MS of compound **9h:**

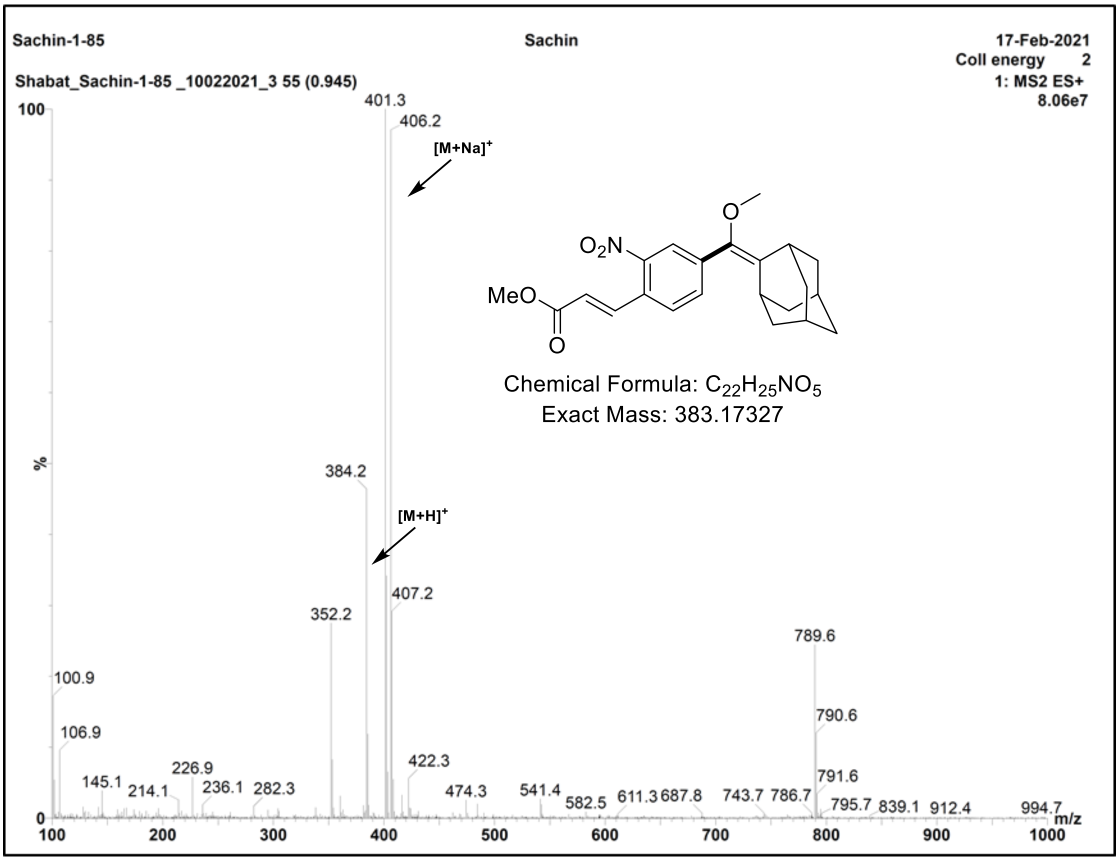

^{1}H-NMR and ^{13}C-NMR spectra of compound **9i:**

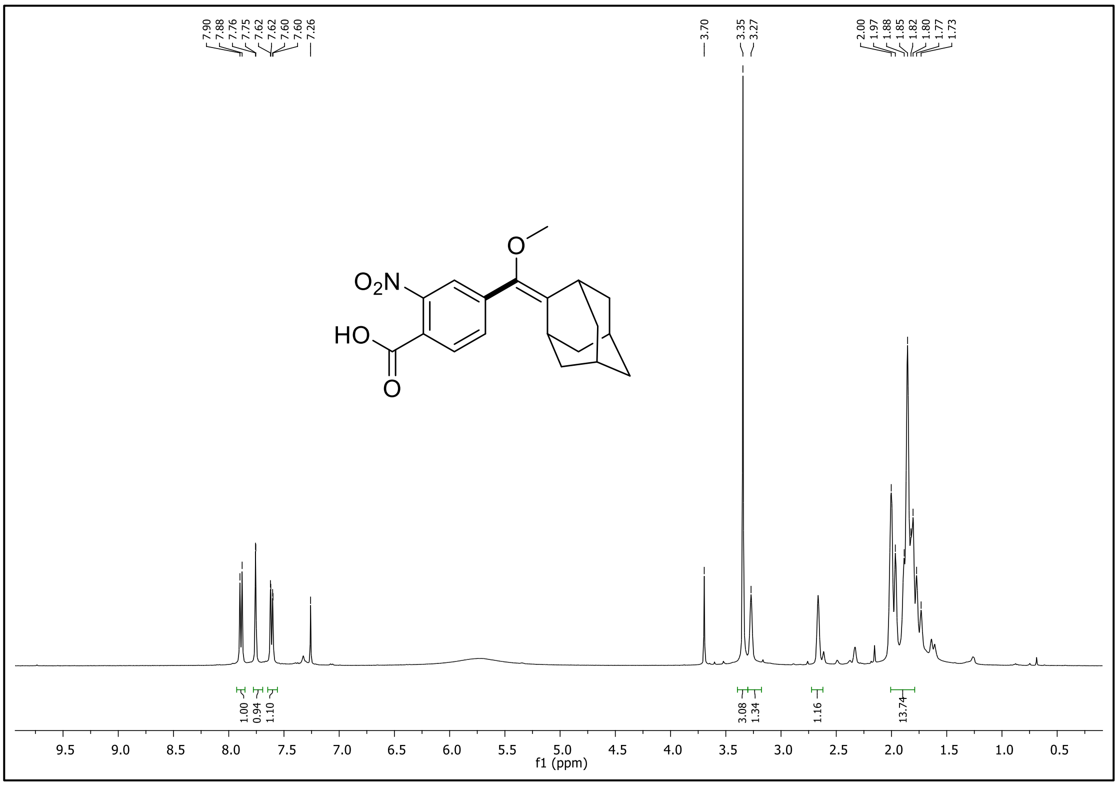

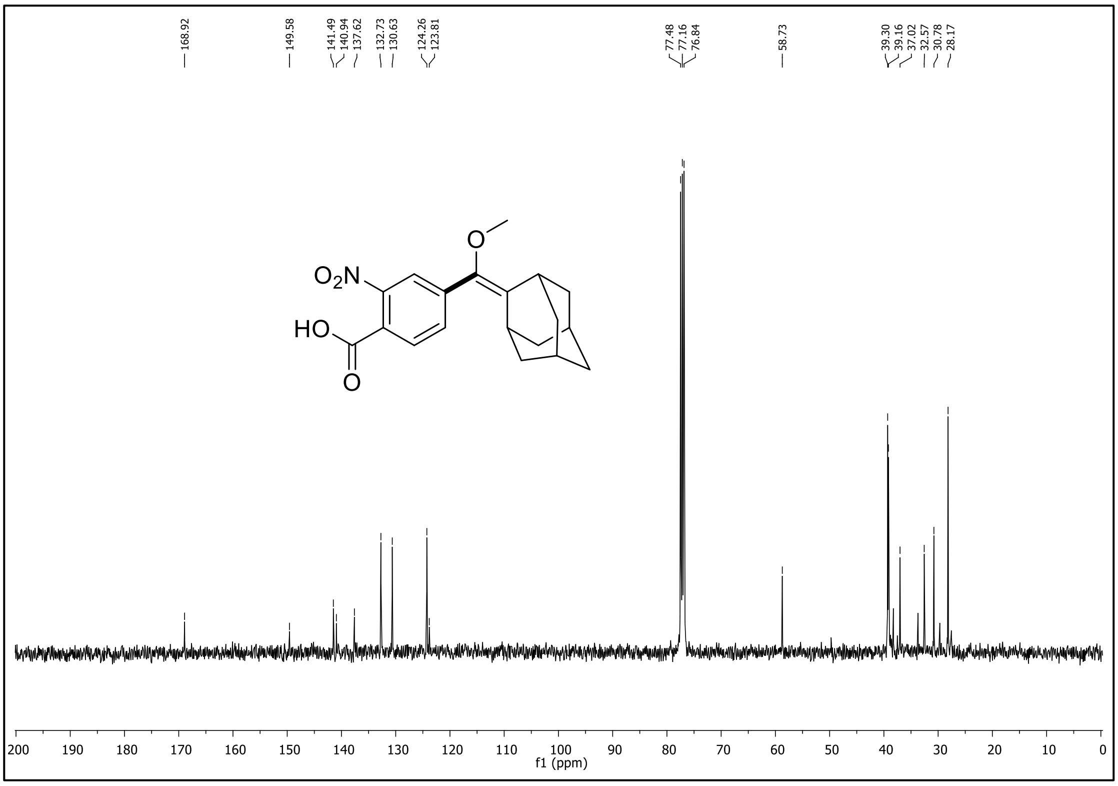

MS of compound **9i:**

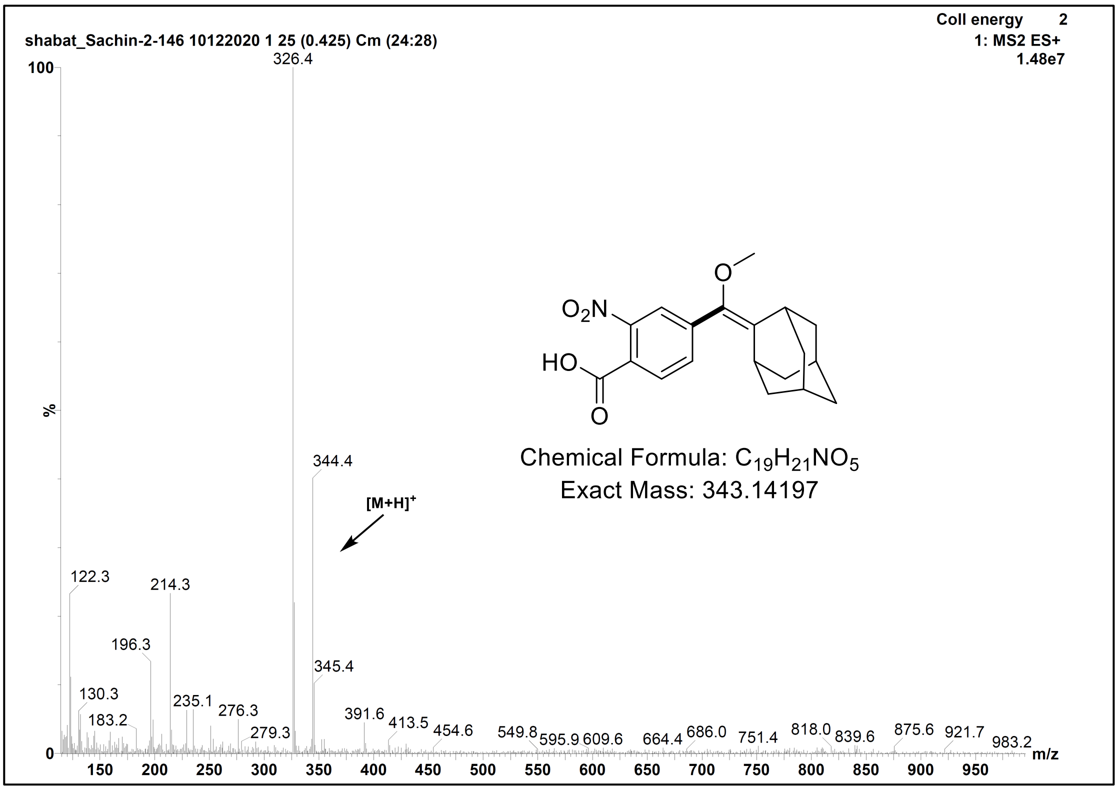

^{1}H-NMR and ^{13}C-NMR spectra of compound **9j:**

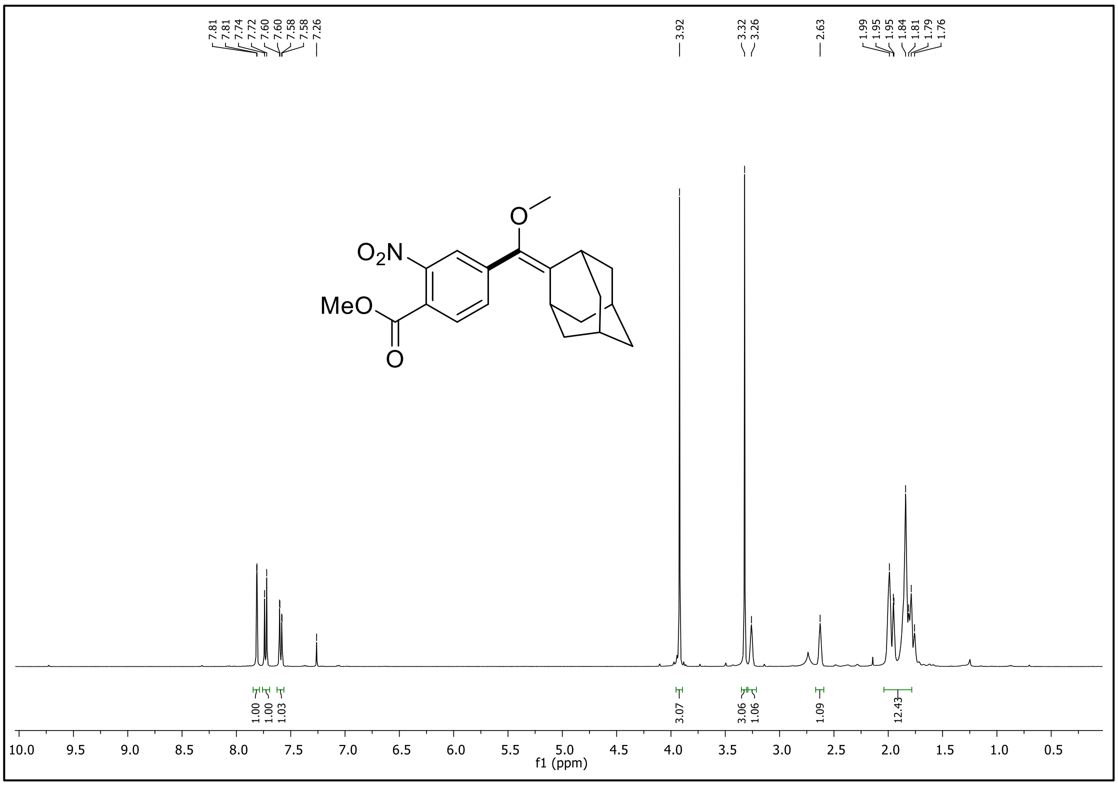

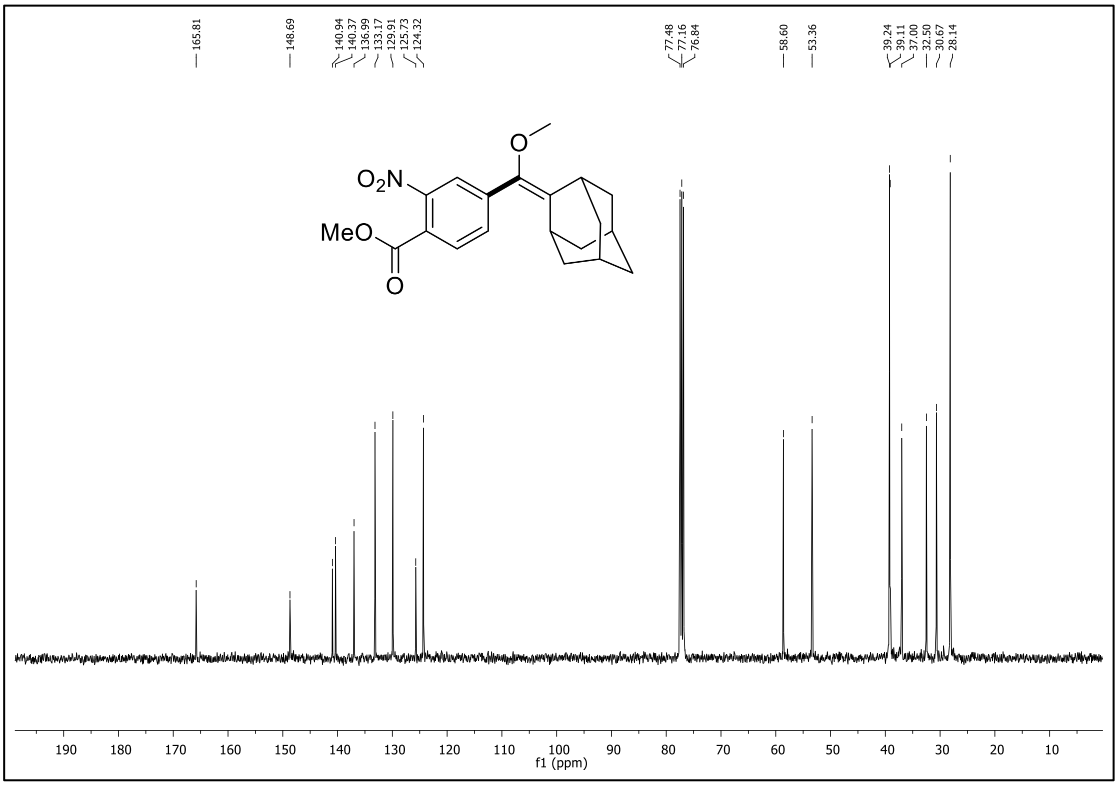

MS of compound **9j:**

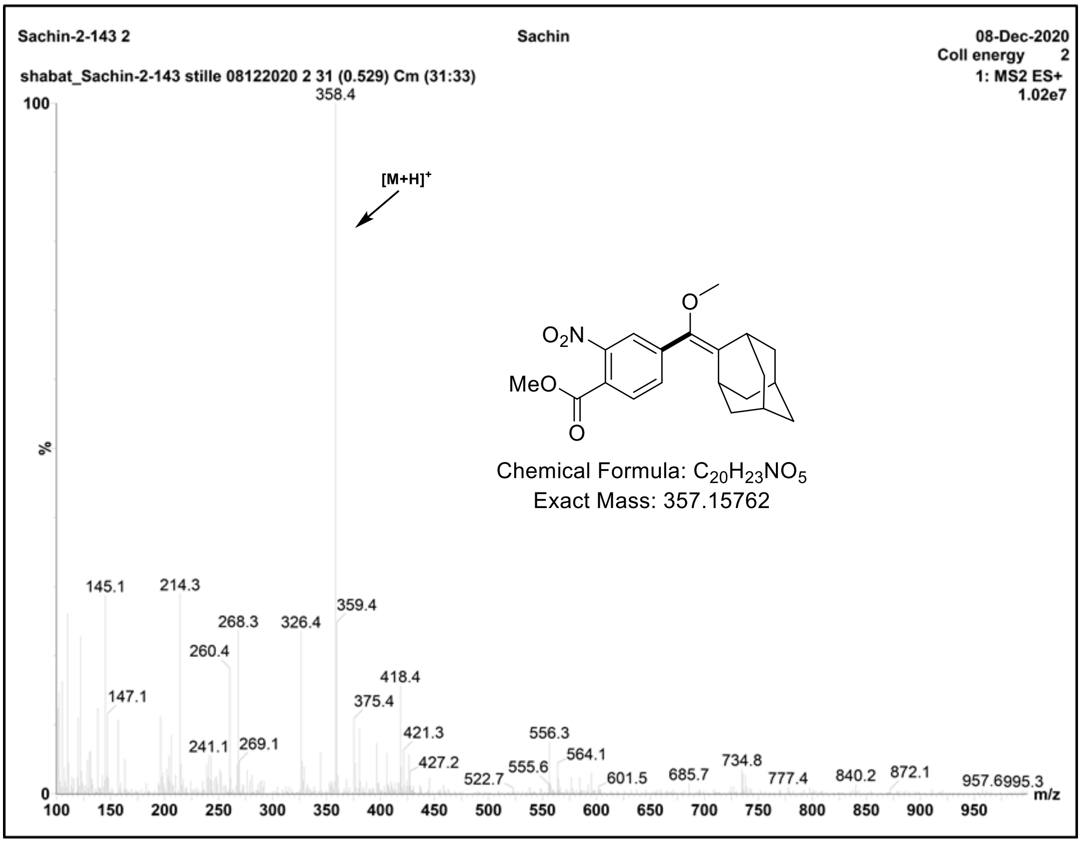

^{1}H-NMR and ^{13}C-NMR spectra of **9k**:

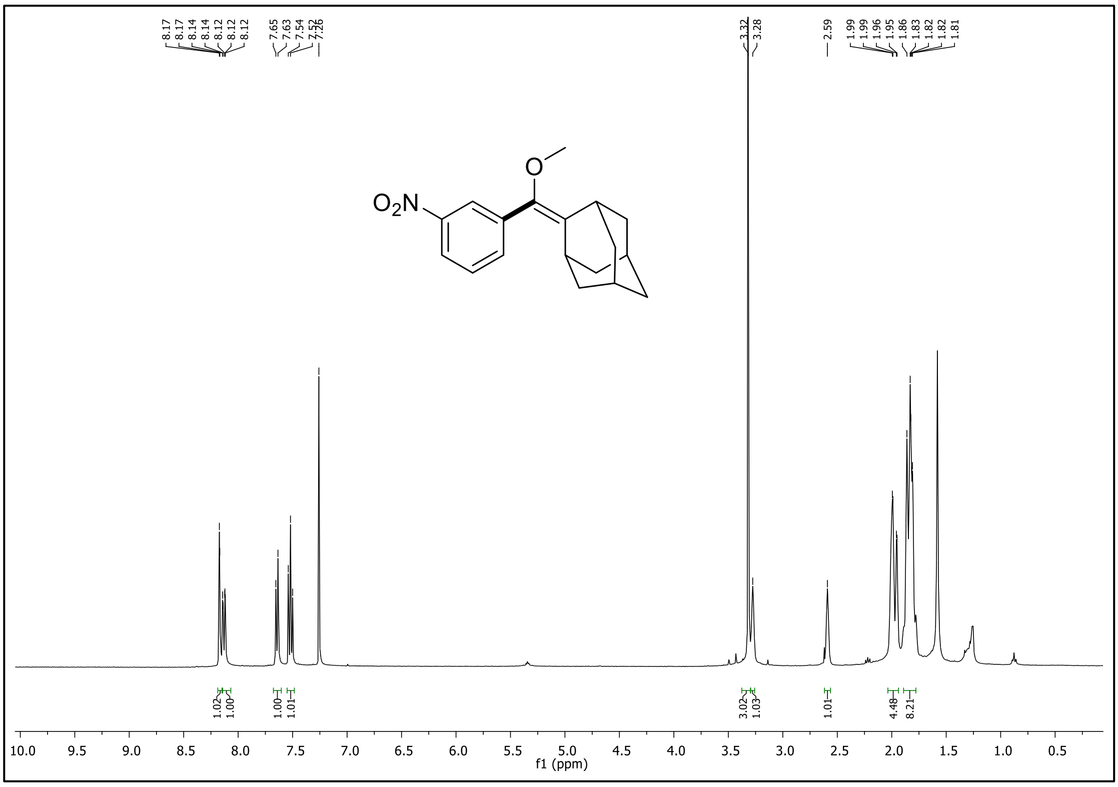

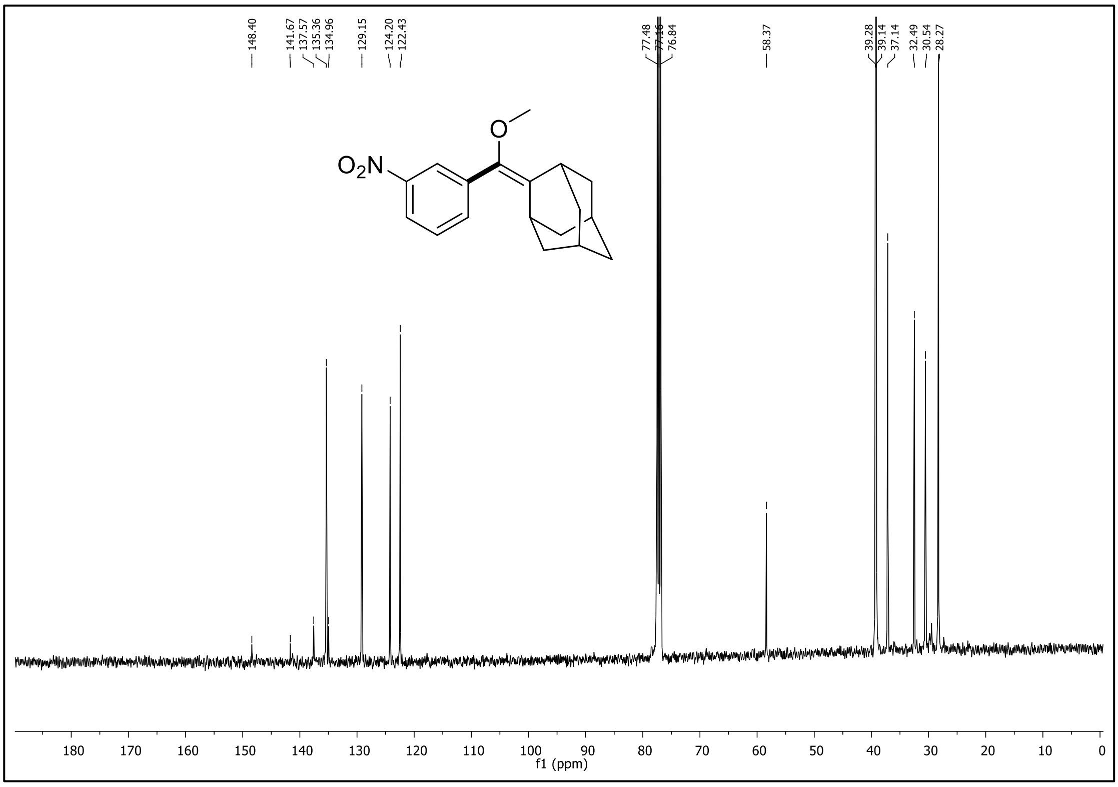

MS of **9k:**

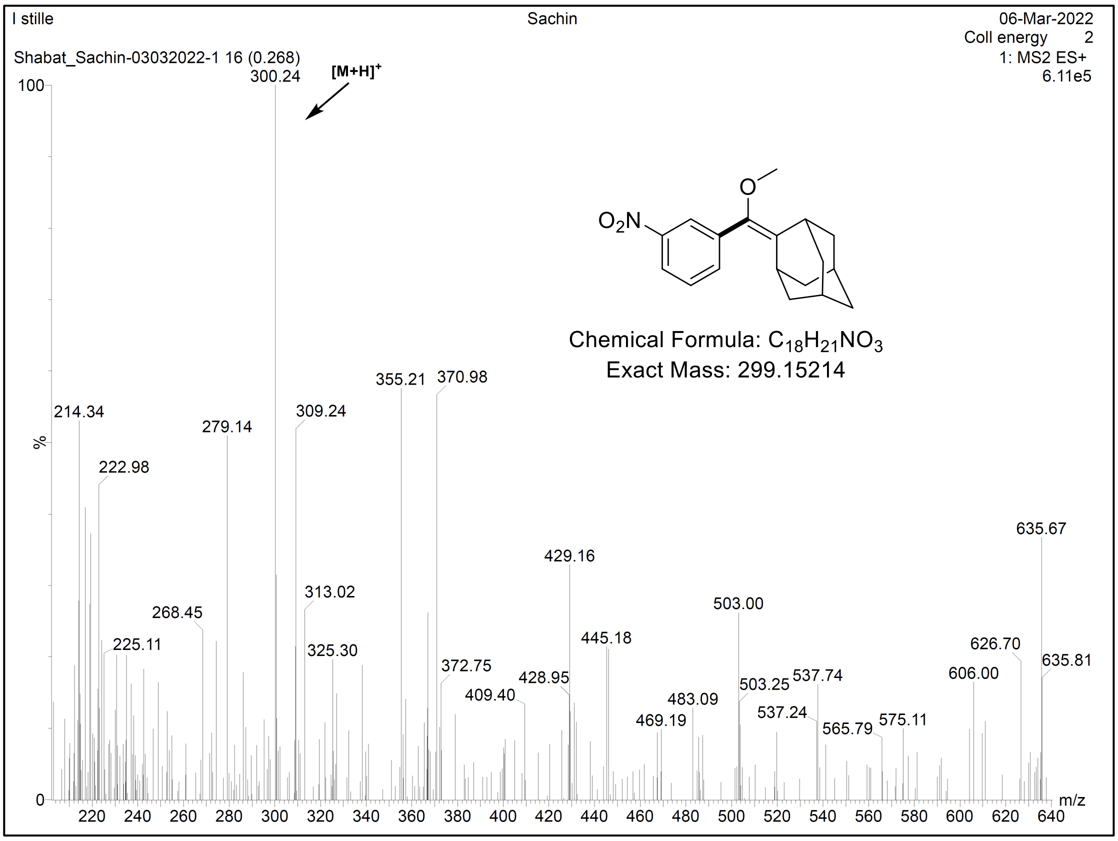

I stille
Sachin
06-Mar-2022
Coll energy 2
Shabat_Sachin-03032022-1 16 (0.268)
1: MS2 ES+
6.11e5
[M+H]+
300.24
214.34
222.98
225.11
268.45
279.14
309.24
313.02
325.30
355.21
370.98
372.75
409.40
428.95
429.16
445.18
469.19
483.09
503.00
503.25
537.24
537.74
565.79
575.11
606.00
626.70
635.67
635.81
O2N
O
Chemical Formula: C18H21NO3
Exact Mass: 299.15214
%
m/z

^{1}H-NMR and ^{13}C-NMR spectra of **7-amino coumarin dioxetane:**

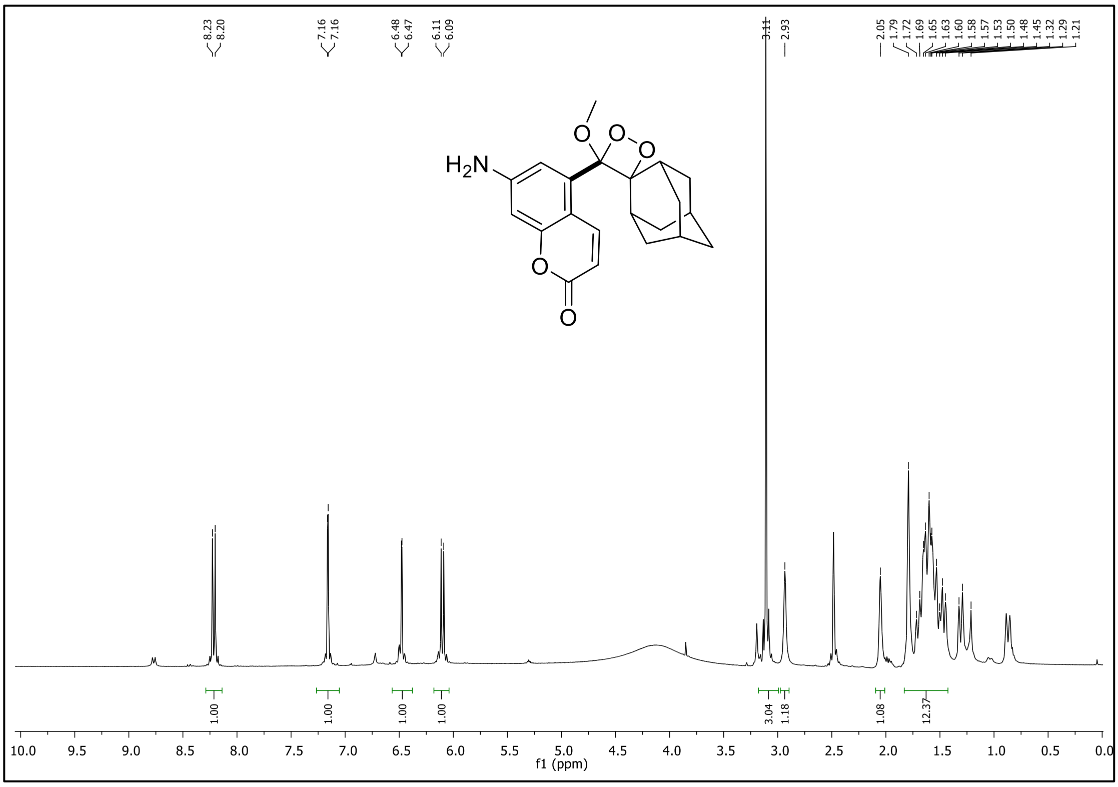

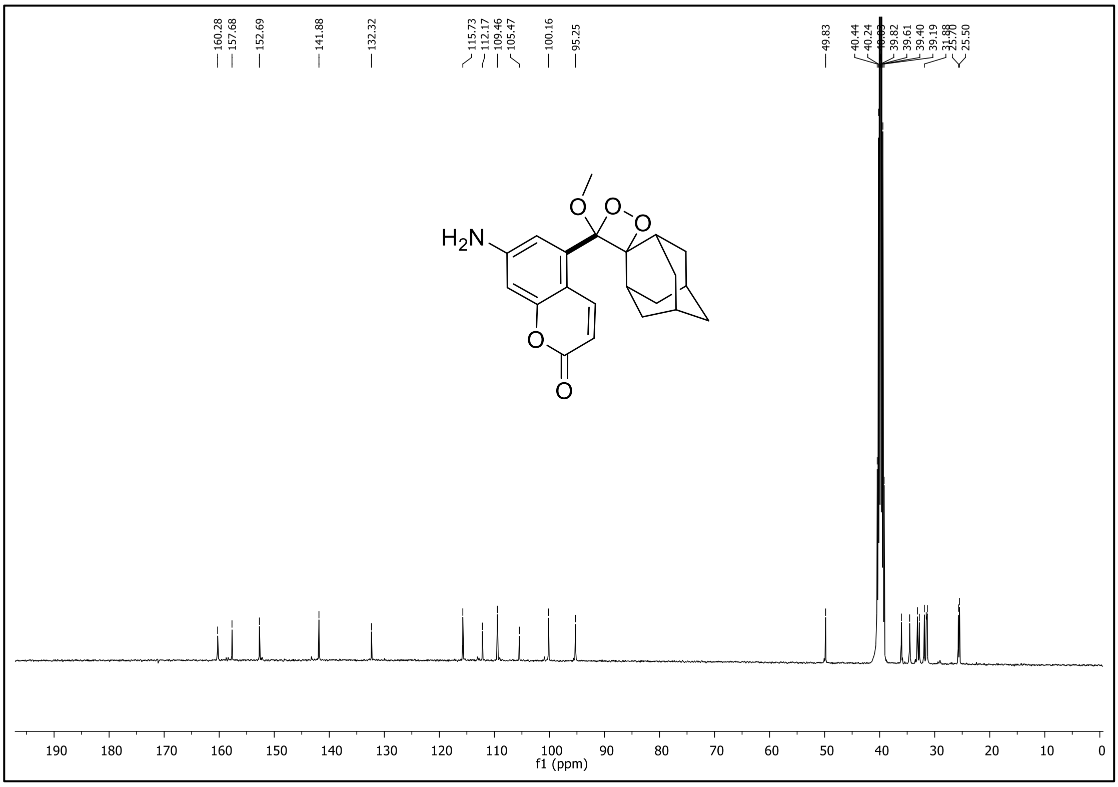

MS of compound **7-amino coumarin dioxetane:**

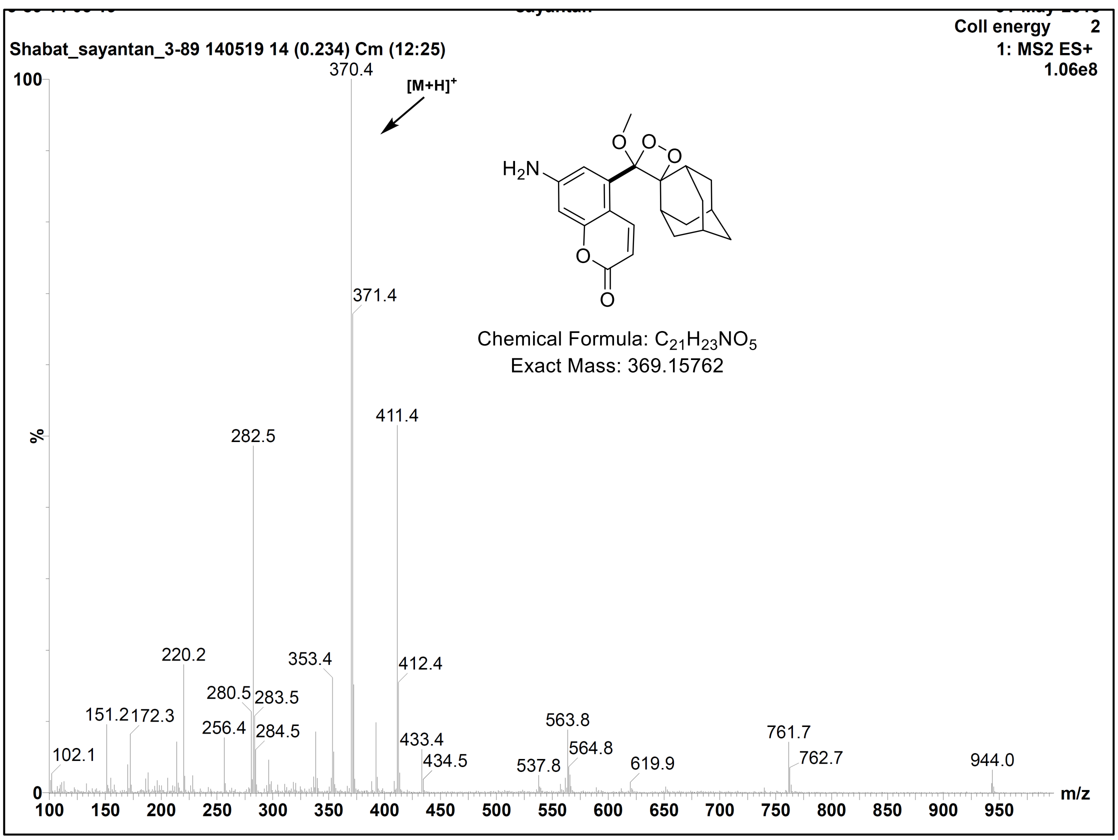

^{1}H-NMR and ^{13}C-NMR spectra of **TBSO-pyridine enol ether:**

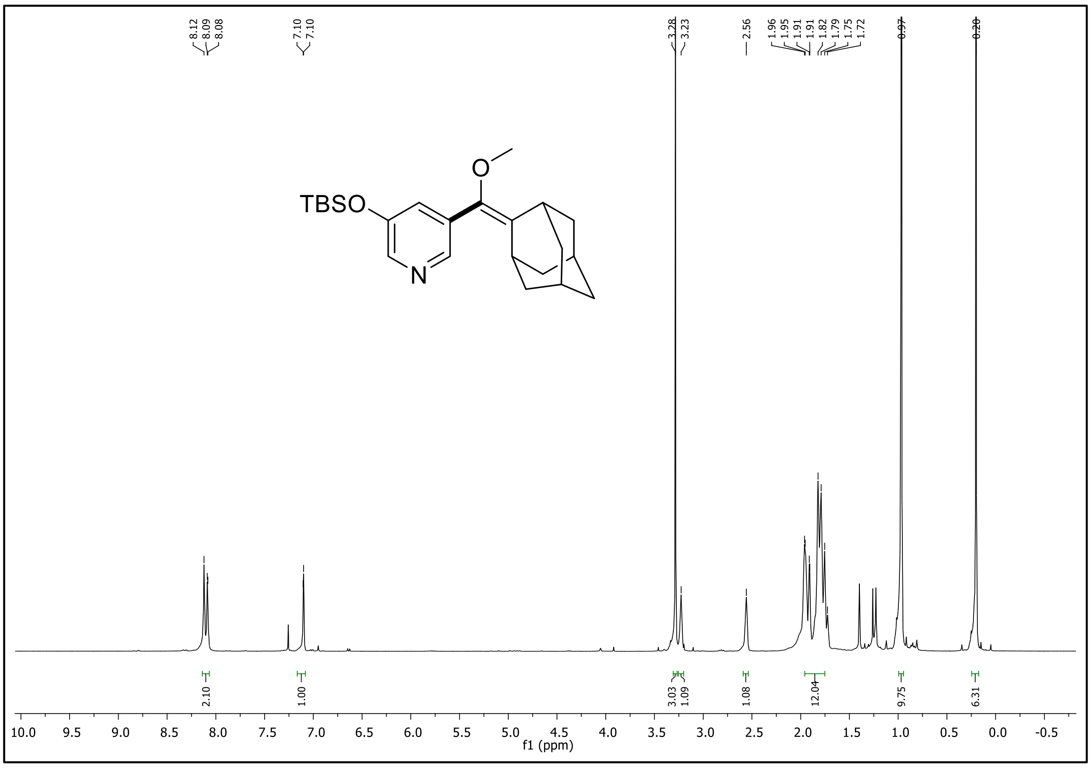

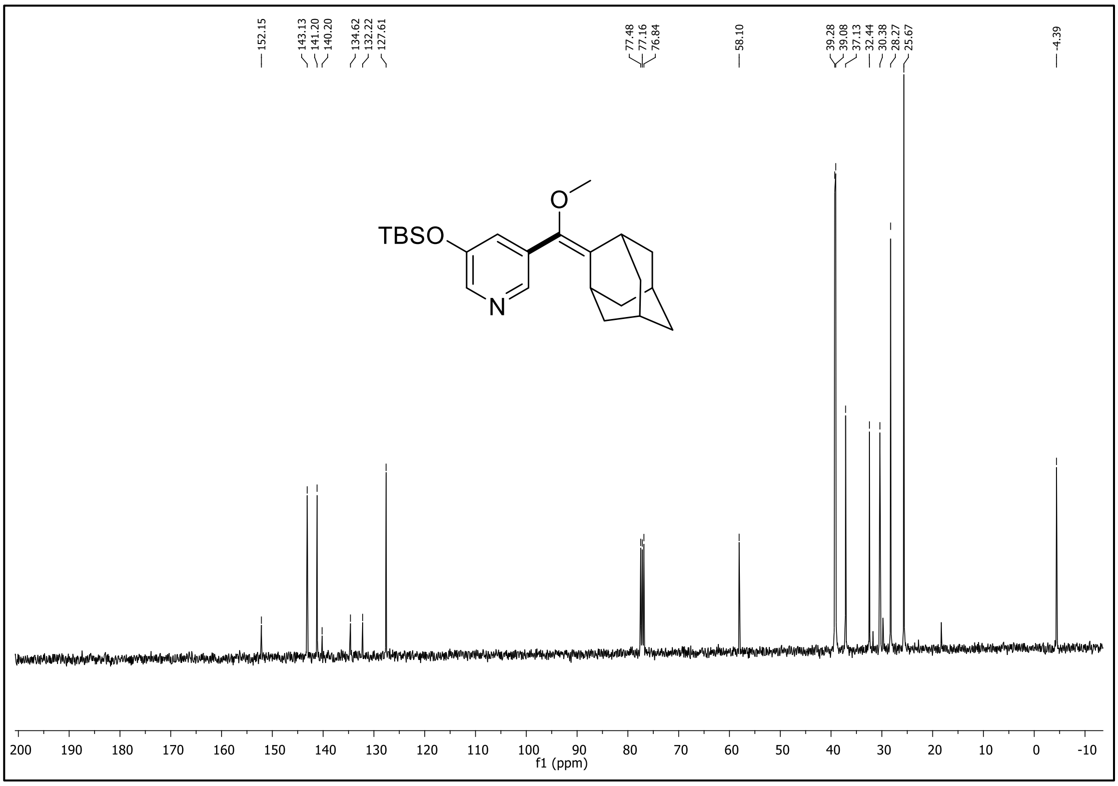

MS of **TBSO-pyridine enol ether:**

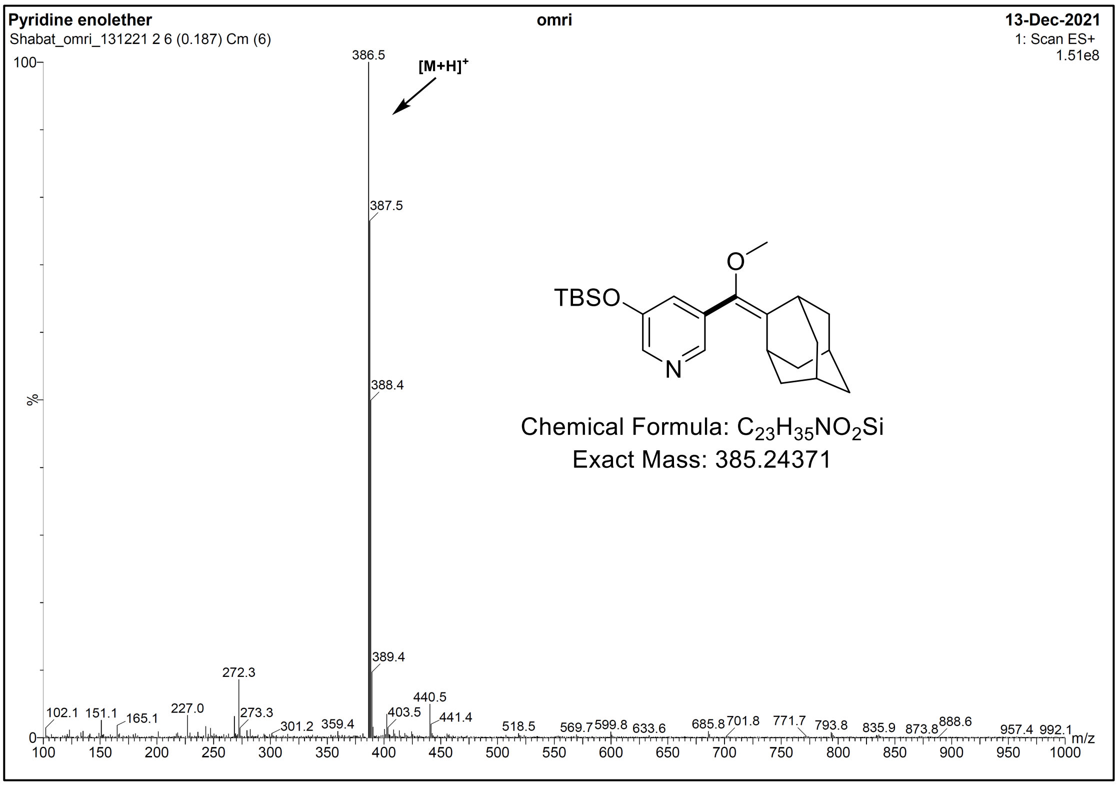

^{1}H-NMR and ^{13}C-NMR spectra of **Py-dioxetane:**

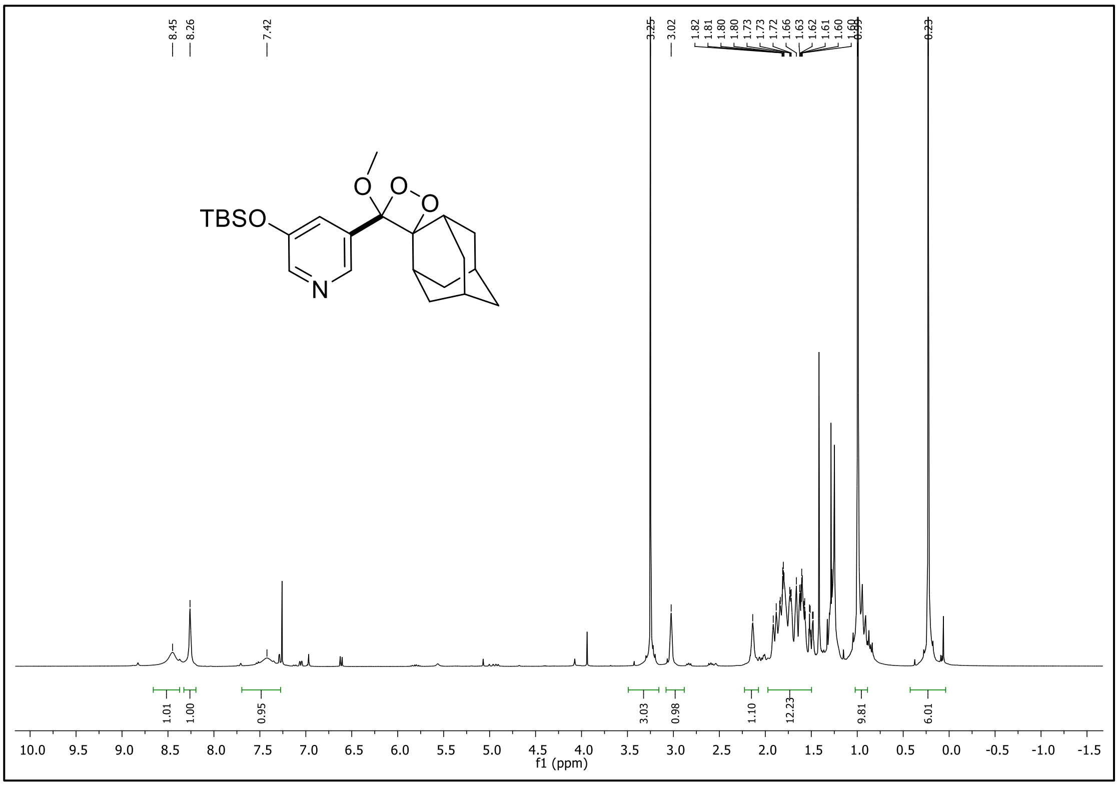

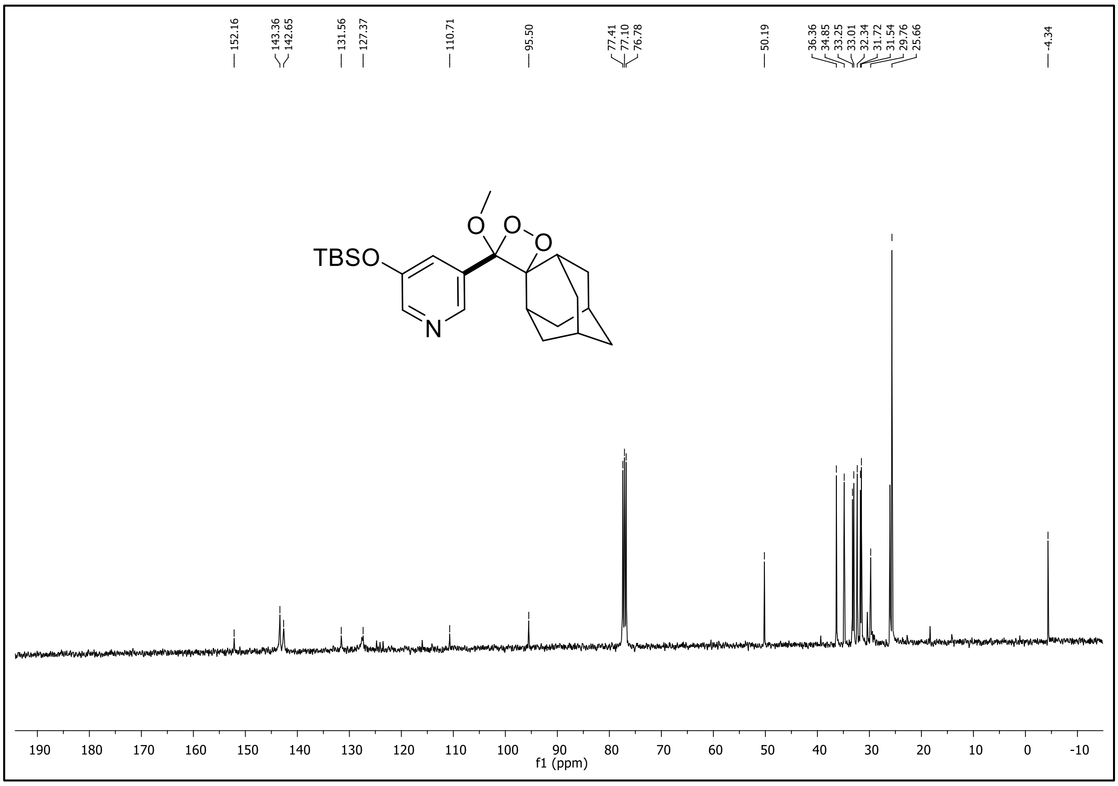

MS of **Py-dioxetane:**

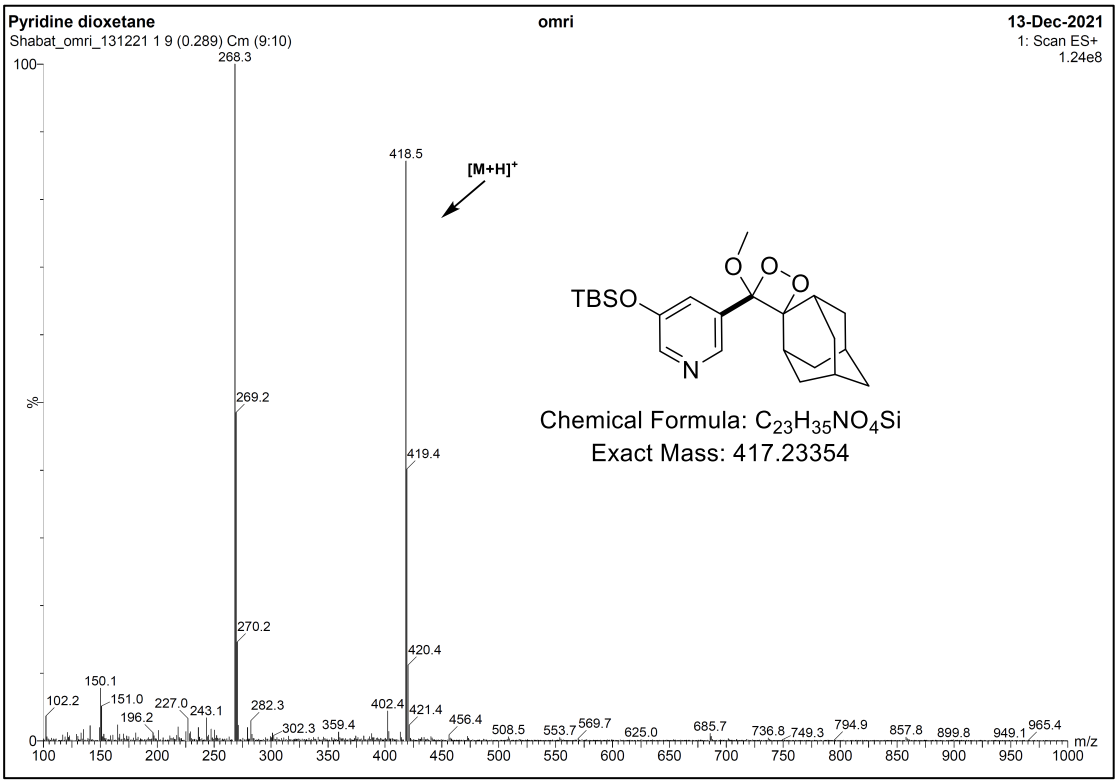

MS of **CyOH dioxetane:**

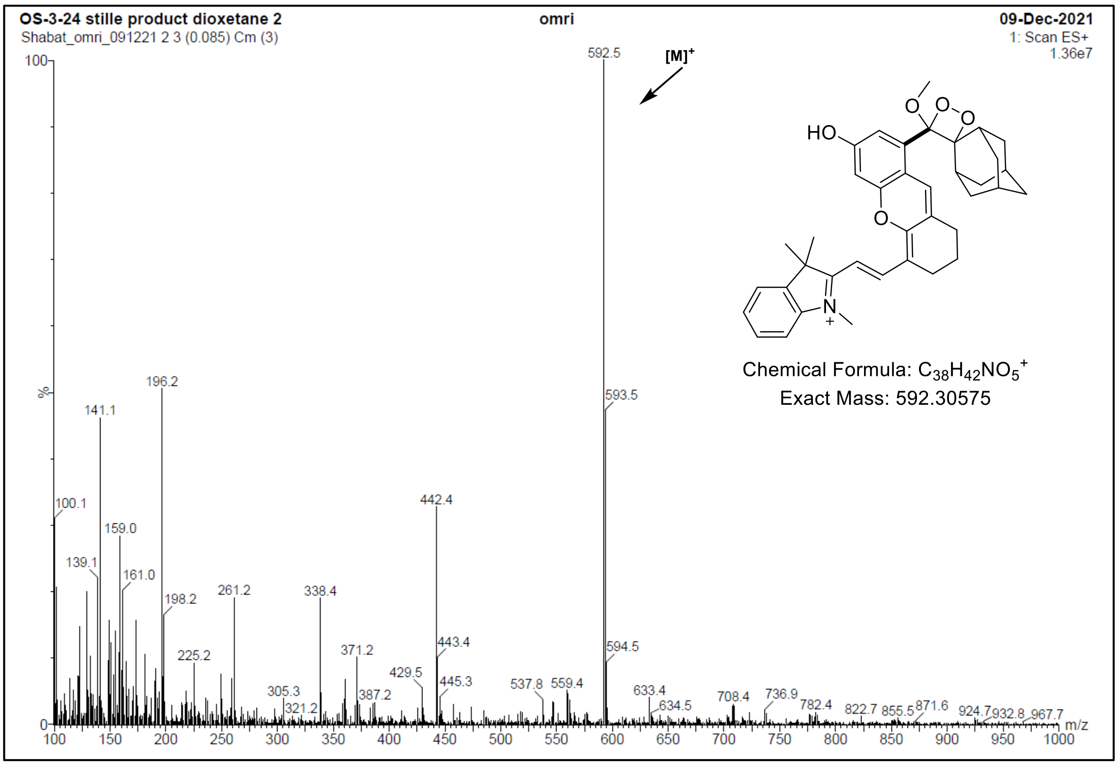

MS of compound **7w:**

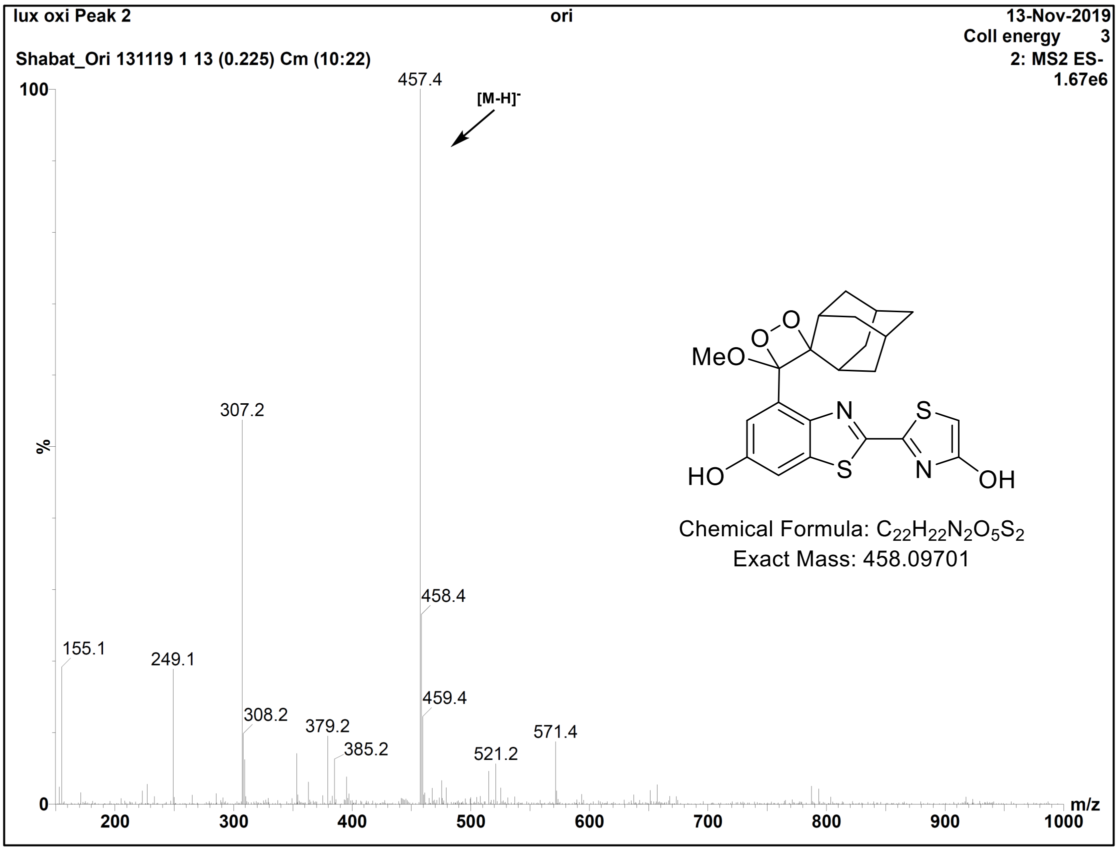

4. References

[1] G. Akyüz, F. Ş. Beriş, B. Kahveci, E. Menteşe, *J. Heterocyclic Chem.* **2019**, *56*, 3065-3072.

[2] J. M. Baccei, Y. Bravo, A. C-Y. Chen, J. Roppe, T. Schrader, Y. Xiong, U.S. Patent WO 2021071843, April 15, **2021**.

[3] M. Irie, Japan Patent JP07248570, **1995**.

[4] M. B. Dewal, U.S. Patent WO 2020142490 A1, July 9, 2020.

[5] K. Elumalai, W. K. Leong, *Tetrahedron Lett.* **2018**, *59*, 113-116.

[6] M. Kumar, S. Bagchi, A. Sharma, *New J. Chem.* **2015**, *39*, 8329-8336.

[7] F. L. Callonnec, E. Fouquet, F.-X. Felpin, *Org. Lett.*, **2011**, *13*, 2646-2649.

[8] T. Symeonidis, M. Chamilos, D. J. Hadjipavlou-Litina, M. Kallitsakis, K. E. Litinas, *Bioorg. Med. Chem. Lett.,* **2009**, *19*, 1139-1142.

[9] S. Ioka, T. Saitoh, S. Iwano, K. Suzuki, S. A. Maki, A. Miyawaki, M. Imoto, S. Nishiyama, *Chem. Eur. J.* **2016**, *22*, 9330-9337.

[10] V. Guilarte, M. P. Castroviejo, P. García-García, M. A. Fernández-Rodríguez, R. Sanz, *J. Org. Chem.* **2011**, *76*, 9, 3416-3437.

[11] R. C. Steinhardt, C. M. Rathbun, B. T. Krull, J. Yu, Y. Yang, B. D. Nguyen, J. Kwon, D. C. McCutcheon, K. A. Jones, F. Furche, J. A. Prescher, *Chembiochem.* **2017**, *18*, 96-100.

[12] S. Son, M. Won, O. Green, N. Hananya, A. Sharma, Y. Jeon, J. H. Kwak, J. L. Sessler, D. Shabat, J. S. Kim, *Angew. Chem. Int. Ed.* **2019**, *58*, 1739-1743.
